# CircRNF111 Protects Against Insulin Resistance and Lipid Deposition via Regulating miR-143-3p/IGF2R Axis in Metabolic Syndrome

**DOI:** 10.3389/fcell.2021.663148

**Published:** 2021-08-17

**Authors:** Xihua Lin, Ying Du, Weina Lu, Weiwei Gui, Shuiya Sun, Yiyi Zhu, Gangliang Wang, Daniel Turunen Eserberg, Fenping Zheng, Jiaqiang Zhou, Fang Wu, Hong Li

**Affiliations:** ^1^Department of Endocrinology, Sir Run Run Shaw Hospital, School of Medicine, Zhejiang University, Hangzhou, China; ^2^Key Laboratory of Biotherapy of Zhejiang Province, Biomedical Research Center, Sir Run Run Shaw Hospital, Zhejiang University, Hangzhou, China; ^3^School of Medicine, Zhejiang University, Hangzhou, China; ^4^Department of Endocrinology, Peking Union Medical College Hospital, Peking Union Medical College, Chinese Academy of Medical Sciences, Beijing, China; ^5^Department of Orthopaedics Surgery, Sir Run Run Shaw Hospital, School of Medicine, Zhejiang University, Hangzhou, China

**Keywords:** circRNF111, miR-143-3p, IGF2R, circular RNAs, insulin resistance, lipid deposition, metabolic syndrome

## Abstract

Abnormal expression of circRNAs (circular RNAs), a subclass of non-coding RNAs, has been documented in numerous human diseases. Herein, we explored whether circRNAs act as ceRNAs (competing endogenous RNAs) to modulate the pathological process-insulin resistance, as well as dyslipidemia of MetS (Metabolic Syndrome). The profile of circRNAs in serume of MetS and control samples was characterized by circRNA deep sequencing. We identified circRNF111 as a key downregulated circRNA involved in MetS. The decreased expression of circRNF111 in the serum samples of MetS was directly linked to excessive insulin resistance and dyslipidemia. Loss-of-function experiments showed that circRNF111 knockdown inhibited the glucose uptake and the Akt signaling pathway, meanwhile increased the deposition of triglycerides in adipogenic differentiated hADSCs (human adipose-derived stem cells). Mechanistically, circRNF111 sponged miR-143-3p and functioned via targeting miR-143-3p along with its downstream target gene IGF2R. The role along with the mechanism of circRNF111 sponging miR-143-3p in MetS was also explored in obese mice triggered by high-fat die. Therefore, our data suggest a protective role of the novel circRNA-circRNF111 in MetS progression. CircRNF111 inhibition enhances insulin resistance and lipid deposition in MetS through regulating miR-143-3p-IGF2R cascade. This provides a promising therapeutic approach for MetS.

## Introduction

Metabolic syndrome (MetS) is a group of predisposing factors, consisting of low high-density lipoprotein content, high glucose, hypertension, obesity along with high triglyceride contents (Rodríguez-Monforte et al., 2019). Patients with MetS have a relatively increased risk of about twofold of developing cardiovascular disease in a period of 5–10 years and at least 5-fold for developing type-2 diabetes, and thus an approximately 1.6-fold increased risk of mortality (Kaur, 2014). In 2005, the International Diabetes Federation estimated that one-quarter of the world’s population had MetS (Zimmet et al., 2005). The overall standardized MetS prevalence was about 24.2% (24.6% in men and 23.8% in women) in China in 2012 on the basis of CNNHS (China National Nutrition and Health Survey) statistics (Li Y. et al., 2018). A population-based pooled assessment involving 2416 studies consisting of 128.9 million children, adolescents, as well as adults from the Lancet showed that China has the largest number of obese and severely obese people in the world, to 1st rank for both men and women in 2014 (NCD Risk Factor Collaboration (NCD-RisC), 2016). Insulin resistance and obesity-induced dyslipidemia are recognized as the primary causes of most MetS cases (Gobato et al., 2014). A critical relationship of MetS with dyslipidemia involves insulin resistance development in peripheral tissues resulting in an elevated hepatic surge of fatty acids from intravascular lipolysis, dietary sources, as well as adipose tissue that is resistant to the antilipolytic influences of insulin (Klop et al., 2013; Aroor et al., 2019). The mechanism of insulin resistance still remains elusive, however. Hence, it is necessary to design improved approaches of averting insulin resistance, therefore enhancing MetS prognosis.

It is emerging increasingly obvious that alterations in miRNA (microRNA) expression levels critically participate in the modulation of disease processes, including MetS progression (Ramzan et al., 2019). Our previous studies have found that miR-143-3p expression in serum and urine samples of individuals with MetS was higher in contrast with those of non-MetS controls, and miR-143-3p overexpression could promote insulin resistance and lipid deposition in adipose tissues. This effect is mediated by the gene silencing influence of miR-143-3p on the 3′UTR of IGF2R (insulin-like growth factor 2 receptor), which consequently affects the classical insulin-induced intra-cellular IRS1-PI3K-Akt signaling pathway (Xihua et al., 2019). Recent research evidence documented that ceRNAs (competitive endogenous RNAs) sponge miRNA via their docking sites, and that alterations in the abundance of ceRNAs from individual genes regulates miRNAs (Tay et al., 2014). In addition to miRNA, RNA sequencing detects long RNAs comprising lncRNAs (long non-coding RNAs), circRNAs (circular RNAs), and mRNA (Cech and Steitz, 2014; Kristensen et al., 2019). Of interest, circRNAs either sponge miRNA or ceRNAs to modulate gene expression and competitively inhibit miRNA activity, and its abnormal expression is linked to important biological processes (Hansen et al., 2013; Kristensen et al., 2019). For example, circular RNA ciRS-7 has been shown to counteract miR-7 mediated repression of mRNA (Li R. C. et al., 2018).

CircRNAs, a novel group of non-coding RNA firstly identified by RNA-seq methods in 2012, which are single-stranded, and covalently closed loops formed by ‘back-splicing.’ They are abundantly expressed in a tissue-distinct, developmental stage-distinct as well as disease-distinct manner (Ivanov et al., 2015; Zhang et al., 2016; Kristensen et al., 2019). Mainly existing in eukaryotic cells’ cytoplasm, circRNAs can also be packaged into exosomes or extracellular vesicles and selectively released into bodily fluids. The biogenesis of circRNAs is now known to be regulated by multiple specific elements and factors (Eger et al., 2018). CircRNAs function as regulators of transcription and translation, which includes miRNA binding, protein binding, gene expression regulating, and acting as templates for translation (Zhou et al., 2020). Utilizing the high-throughput RNA sequencing technology, recent discoveries have illustrated that a great deal of endogenous circRNAs are extensively expressed both in human and rodents tissues. Many studies have reported their function in disease such as diabetes mellitus, neurodegenerative diseases, cancer, chronic inflammatory diseases, as well as cardiovascular diseases (Holdt et al., 2016; Hanan et al., 2017; Fang et al., 2018; Kristensen et al., 2018; Aufiero et al., 2019). However, the function of circRNAs in MetS and its core step—insulin resistance remains unclear. Hence, herein, we purposed to explore the functional circRNAs in insulin resistance progression.

Herein, we uncovered and characterized a circRNA (hsa_circ_0001982, derived from exon 2 of RNF111 gene, thereafter termed circRNF111) in MetS. We subsequently validated its biological regulatory function in obesity-induced insulin resistance and lipidosis through sponging of miR-143-3p, which downregulated IGF2R expression.

## Materials and Methods

### Cross-Sectional Study Subjects

We selected the study participants from a population-based cross-section survey conducted between March 2010 and May 2010 in Hangzhou, Zhejiang Province, China in the Caihe community, as documented previously (Xueyao et al., 2014). In brief, 624 participants (from the Han Chinese community, aged between 40 and 65 years old; mean age-56.40 ± 6.52 years) were enrolled in our study. Of which, 51.25% were male. Each of the study subject completed the population based cross-section survey and was assigned a unique number. After that, an electronic method was employed to generate random numbers, and we selected 40 individuals with MetS (56.82 ± 6.58 years; 57.5% male) and 40 healthy controls (56.33 ± 6.12 years; 45.0% male). Next, urine along with serum samples were obtained from the participants. The Ethics Committee of Sir Run Run Shaw Hospital approved this study. Besides, each participant gave an informed consent. Trained medical personnel conducted a face-to-face interview of the study subjects and filled a questionnaire involving the medical history, demographic data, health-linked data, lifestyle, and medical therapy. All baseline the anthropometric, as well as metabolic assessments were recorded with standardized approaches as previously documented (Xueyao et al., 2014; Xihua et al., 2019).

### Diagnosis of MetS

The diagnosis of MetS was on the basis of the criteria developed by JCDCG (Joint Committee for Developing Chinese Guidelines on Prevention and Treatment of Dyslipidemia in Adults) (Joint Committee for Developing Chinese guidelines on Prevention and Treatment of Dyslipidemia in Adults, 2007). The HOMA-IR (Homeostasis Model of Assessment-Insulin Resistance) has been used as a surrogate measure of insulin resistance for epidemiological studies in pediatric populations and it assumes that hepatic and peripheral insulin resistance are equal (da Silva et al., 2019). Determination of the HOMA-IR value was based on a previously documented formula: HOMA-IR = [{fasting plasma glucose (mmol/L × fasting insulin (mIU/L)}/22.5] (Emoto et al., 1999). We excluded patients with renal dysfunction or aberrant renal function at the time of enrollment. The healthy controls included individuals who did not meet any of the above criteria.

### Circular RNA Library Construction and Sequencing

The TriZol reagent (cat. no., Invitrogen, Carlsbad, CA, United States) was employed to isolate tRNA from serum samples of 4 clinical MetS and 4 control tissues as described by the manufacturer. The RNA concentration along with purity of all the samples were checked with a NanoDrop ND-1000 (NanoDrop, Wilmington, DE, United States). Afterward, an Agilent 2100 Bioanalyzer (Agilent Technologies) was employed to assess the RNA integrity, with a threshold of RIN > 7.0. Next, 5 μg of tRNA was subjected to digestion of ribosomal RNA by using a Ribo-Zero^TM^ rRNA Removal Kit (cat. no., Illumina Inc.) as described in the manufacturer manual. The remaining RNA was inoculated with RNase R (cat. no., Epicentre Inc., Madison, WI, United States) to digest linear RNAs, as well as enrich circRNAs. Following the ribosomal and linear RNA removal steps, divalent cations were employed to fragment the enriched circRNAs under high temperature. Subsequently, cDNAs were generated from the cleaved RNA fragments. Then, *Escherichia coli* DNA polymerase I, dUTP, and RNase H were employed to generate U-labeled second-strand DNA from the cDNA. After that, an A-base was introduced to the blunt ends of every strand to prepare them for ligation to the indexed adapters. Every adapter harbored a T-base overhang to allow ligation to the A-tailed fragmented DNA. We ligated dual- or single-index adapters to the fragments, and AMPure XP beads were employed to perform size selection, following heat-labile UDG enzyme digestion of the U-labeled second-stranded DNAs. The average insert size of the final cDNA library was 300 bp (±50 bp). Lastly, paired-end deep sequencing was run on an Illumina HiSeq 4000 (LC Bio, China) as described by the manufacturer. RNA libraries were sequenced by Shanghai Origingene Bio-Pharm Biotechnology Co., Ltd.

### Circular RNA Data Analysis

First, Cutadapt (Martin, 2011; Chen et al., 2014) was employed to filter out the reads with adaptor contamination, undetermined bases along with low quality bases. Next, FastQC^1^ (Brown et al., 2017) was employed to verify the sequence quality. TopHat2 (Kim et al., 2013) and STAR (Dobin et al., 2013) were employed to map reads to hg19 (the human genome). Afterward, CIRI2 (Gao et al., 2015) along with CIRCexplore (Dong et al., 2019) were employed to determine back splicing reads, and subsequently *de novo* assemble the mapped reads to circRNAs. The circRNAs uncovered by both algorithms were considered as valid candidates. All the samples produced unique circular RNAs. To explore circRNAs expression levels, we standardized the back-spliced reads (evidence for circRNA) via read length, as well as mapped read numbers [spliced reads/billion mapping, designated as SRPBM (Zheng et al., 2016)], which allows relative quantification of the back splicing from different RNA-seq data. circRNAs which were differentially expressed were identified using edgeR R package with criteria of | log2 (fold change)| > 1 along with *p* < 0.05.

### RT-PCR Assessment of circRNA/mRNA Expression

The TriZol reagent (cat. no., Invitrogen) was employed to isolate total cellular RNA. After that, cDNAs were generated from the RNAs with the reverse transcription reagents kit (cat. no., TaKaRa). Subsequently, the SYBR Green (cat. no., TaKaRa) was employed to perform qPCR and the reaction was run on the 7500 Real-Time PCR System (Applied Biosystems). For the circRNAs, we inoculated the total cellular RNAs with or without 3 U/μg of RNase R (cat. no., Epicentre, San Diego, CA, United States) for 20 min at 37°C. Afterward, the RNeasy MinElute Cleanup Kit (cat. no., Qiagen) was employed to purify the resulting RNA, and then amplification of the circRNA was performed using the specific divergent primers of the back-splice junction of circRNF111. Next, agarose gel electrophoresis of the PCR products was performed, followed by sequencing to confirm the amplicons. All the reactions were replicated thrice. GAPDH was employed as the normalization standard of mRNA and circRNF111 expression. The 2^–ΔΔ*Ct*^ approach was employed to determine relative expression. The primer sequences are indicated in the Supplementary Table 10.

### Serum and Urine miRNA Detection

The miRNeasy kit (cat. no., Qiagen) was employed to isolate total RNA from serum; manufacturer instructions were followed. For each reaction, 2–4 μl of tRNA isolated from 250 μl–1 ml of serum samples was used. The TriZol reagent (cat. no., Invitrogen) was employed to isolate tRNA from urine, and the Urine RNA Purification Kit (cat. no., Abnova) employed to purify the RNA; manufacturer instructions were adhered to. For each reaction, we used 2–4 μl of tRNA extracted from 5 to 10 ml of urine samples. The expression microRNAs were validated using the MiDETECT Track^TM^ miRNA qRT-PCR Start Kit (cat. no., RiboBio, Guangzhou, China) via poly(A) tailing-based RT-PCR as described by the manufacturer. On the basis of the microarray data, we selected U6 as the stable endogenous control miRNAs. The primer sets of the miRNAs for the qPCR reaction were synthesized by Guangzhou RiboBio Co. (Guangzhou, China).

### RNA Fluorescent *In Situ* Hybridization (FISH)

The FISH assay was carried out in HCs or rabbit tissues. Cy3-labeled circRNF111 probes along with 488-labeled locked nucleic acid miR-143-3p probes were designed and synthesized by RiboBio (Guangzhou, China). The Fluorescent *In Situ* Hybridization Kit (cat. no., RiboBio, Guangzhou, China) was utilized to monitor the signals of the probes as described by the manufacturer. The Nikon A1Si Laser Scanning Confocal Microscope (Nikon Instruments Inc., Japan) was employed to image the RNAs. In the *in vivo* FISH assay, the tissue sections were deparaffinised, followed by rehydration and permeabilization using 0.8% pepsin at 37°C for 30 min prior to hybridization. The primer and prober sequences are listed in Supplementary Table 10.

### Cell Culture and Adipogenic Differentiation of hADSCs Preadipocytes

The hADSCs (human adipose-derived mesenchymal stem cells) along with 293 HEK cells were supplied by the American Type Culture Collection (ATCC). These cells were inoculated in DMEM medium enriched with 10% FBS (cat. no., Bio-Rad), and streptomycin/penicillin (100 IU/mL) under 37°C, 5% CO_2_ and 95% conditions. After 2 days of growth (designated Day 0), a differentiation mixture [consisting of DMEM medium enriched with 10% FBS, insulin (10 μg/mL) (cat. no., TOCRIS, United States) 0.5 mM IBMX (cat. no., Sigma, United States), and 1 μM DEX (cat. no., Sigma, United States)] was introduced to the hADSCs to trigger differentiation into adipocytes. After 2 days, we changed the growth medium with DMEM medium enriched with 10% FBS and 10 μg/mL insulin and the cells were allowed to grow for an additional 2 days. Subsequently, the growth medium was changed after 24 h with DMEM medium enriched with 10% FBS until day 10. The 293HEK cells were employed to conduct the Dual-luciferase enzyme assays.

### Flow Cytometry Evaluation of Glucose Uptake

The activity of glucose uptake in differentiated haDSCa was assessed with 2-NBDG (cat. no., Invitrogen) (Chen et al., 2015). Concisely, the differentiated hADSCs were inoculated with agomirs along with antogomirs in 12-well culture pates for 48 h. Thereafter, the cells were rinsed using DPBS, then inoculated with 100 nM insulin suspended in glucose free-DMEM medium for 10 min. After that, 2-NBDG (60 μM) was introduced to the cell suspension and incubated for 1 h. Next, the cells were rinsed twice with ice-cold DPBS. After trypsinisation, the cells were suspended in DPBS; fluorescence activity was explored via flow cytometry according to manufacturer instructions. Fluorescence activity was determined at wavelengths of excitation and emission of 485 and 535 nm, respectively. The FACScalibur flow cytometer (Becton Dickinson, Franklin Lakes, NJ, United States) was employed to read 2-NBDG fluorescence intensity. We recorded data from 1,000 unique cell events. Blank controls (without 2-NBDG treatment) were employed to rule out false positives by being considered as the background fluorescence. The relative fluorescence intensities minus background fluorescence were employed for data analyses.

### Oil Red O Staining

In the oil red O staining, 10 days post the stimulation of differentiation of hADSCs, the cells were rinsed two times with the D-Hank’s solution. Next, the cells were fixed with 4% formaldehyde for 30 min, followed by rinsing three times in H_2_O. After that, oil red O staining of the cells was performed for 15 min using Oil Red O (cat. no., Sigma, United States). Subsequently, the cells were rinsed thrice using H_2_O, and a microscope (TE2000-E; Nikon, Japan) employed to image the lipid droplets.

### Western Blotting

Fifty microgram of the denatured proteins were fractionated on a 10% SDS-PAGE gels. Then, the fractionated proteins were transfer-embedded onto PVDF membranes (Millipore). After that, 5% skimmed milk was employed to block the membranes for 1 h at RT. Subsequently, the membranes were inoculated with the primary antibodies consisting of polyclonal anti-rabbit phosphorylated Akt, Akt (cat. no., Cell Signaling Technology, 1:1000), anti-mouse β-actin (cat. no., Sigma-Aldrich,1:5000), and polyclonal anti-rabbit IGFBP5 and IGF2R (cat. nos., BD Biosciences, 1:1000) and incubated overnight at 4°C. Thereafter, the membranes were inoculated with HRP-labeled goat corresponding secondary antibodies for 1 h at RT. Lastly, the chemiluminescent ECL assay kit (cat. no., Millipore) was employed to detect the protein bands.

### Pull-Down Assay With Biotinylated circRNF111 Probe

The Pierce Magnetic RNA-Protein Pull-Down Kit (cat. no., Thermo Scientific, 20164) was employed to perform the RNA-Protein pull-down assay. Sangon Biotechnology Company synthesized the Biotin labeled circRNF111 probe. In the assay, 100 pmol biotinylated labeled RNA that is complementary to circRNA111 junction sequence was inoculated with 50 μl Streptavidin Magnetic Beads lysates. Negative control RNA was employed as the contrast. The RNA-bound beads were then inoculated with hADSCs in 1× binding buffer and 50% glycerol for 1 h at 4°C while rotating. After that, we isolated complexes via washing thrice in ice-chilled 1× washing buffer. Subsequently, western blotting, Mass Spectrometry (BIOTREE, China), along with silver staining (Beyotime Biotechnology, China) were employed to detect the RNA-binding protein the pull down.

### CircRNAs *in vivo* Precipitation (circRIP)

The circRIP assay was conducted as documented previously (Han et al., 2017). A total of 1 × 10^7^ hADSCs were fixed with 1% formaldehyde for 10 min, and then lysed, followed by sonication. The probes were incubated at 65°C for 10 min to denature them. After that, the probes were incubated at room temperature (RT) for 2 h for hybridization, followed by addition of 200 μl streptavidin-coated magnetic beads. After centrifugation, we retained 50 μl of the supernatant as input, while the remaining supernatant was inoculated with a mixture of circRNF111-specific probes streptavidin dynabeads (M-280; Invitrogen) and incubated at 30°C overnight. Subsequently, the M-280 dynabeads-probes-circRNAs mixture was rinsed and inoculated with 200 μl of lysis buffer along with proteinase K for reversing the formaldehyde cross-linking. Lastly, the TriZol reagent was employed to extract RNA from the mixture and qRT-PCR analysis was done to explore the strength of binding after reverse transcription of the miRNAs. The probes are indicated in the Supplementary Table 10.

### RNA Immunoprecipitation

The Magna RNA Immunoprecipitation (RIP) Kit (cat. no., Millipore, Bedford, MA, United States) was employed to carry out the Ago-RIP assay in hADSCs cells as described by the manufacturer. About 1 × 10^7^ differentiated hADSCs were sedimented, followed by re-suspension in an equivalent pellet volume of the RIP Lysis Buffer enriched with protease inhibitor cocktail along with RNase inhibitors. Thereafter, 200 μl of the cell lysate was inoculated with antibody against Ago2 (10 μg) (cat. no., Millipore, Billerica, MA, United States) or the control rabbit IgG-coated beads and incubated overnight at 4°C while mixing by rotation. Subsequently, the reaction mixture was inoculated with proteinase K buffer, and the RNeasy MinElute Cleanup Kit (cat. no., Qiagen, Düsseldorf, Germany) employed to isolate the immunoprecipitated RNAs, followed by generation of cDNA using the Prime- Script RT Master Mix (cat. no., TaKaRa, Tokyo, Japan). RT-qPCR was employed to assess circRNF111 relative abundance, with GAPDH serving as the internal control.

### Serum miRNA Microarray

A microarray (Serum/Plasma Focus microRNA PCR Panel, V1.M, Exiqon) was employed to identify genome-wide circulating miRNA profiles in the serum samples from four individuals with MetS and four health individuals. The MetS patients whose samples were utilized in the microarray assay were randomly selected from the 40 MetS patients enrolled in the study. The TaqMan^®^ Array Human MicroRNA Cards B v3 (cat. no. 4444910; Thermo Fisher Scientific, Inc., Waltham, MA, United States) were utilized to achieve comprehensive coverage. This resulted in 377 unique assays distinct to human miRNAs. The analysis of the 8 RNA samples was performed by the Kangchen Biotechnology Co., Ltd. (Shanghai, China).

### Prediction of circRNF111 miRNA Targets

The miRNA docking sites of circRNF111 was predicted with miRanda^2^, TargetScan^3^, along with circBank^4^ (Das, 2012; Agarwal et al., 2015; Liu et al., 2019) bioinformatics data resources. RNAhybrid^5^ web resource (Krüger and Rehmsmeier, 2006) was employed as a tool for predicting miRNA docking sites between circRNF111 and miR-143-3p and to measure the binding accessibility and energy.

### Adenovirus Knockdown

For recombinant adenovirus sh-circRNF111 construction, oligos with the circRNF111 target sequences were employed to clone shRNA-coding sequences into the pDC311-U6-MCMV-EGFP vector (cat. no., Hanbio Co. Ltd., Shanghai, China). For construction of the miR-143-3p sponge adenovirus, self-complementary DNA oligos containing miR-143-3p sequence were synthesized chemically, including the overhangs from the 5′ BamH1- and the 3′ *Eco*RI-restriction site. The annealed oligos were directionally propagated into the BamH1/*Eco*RI-digested pDC311-U6-MCMV-EGFP vector. The pDC311-sh-circRNF111 or pDC311-miR-143-3p-sponge along with pBHGlox_E1, 3Cre (Ng et al., 1999, 2000) were co-inserted into HEK293 cells via transfection with the LipoFiter^TM^ liposome transfection reagent (cat. no., Hanbio, Shanghai, China) to create the recombinant adenoviruses (sh-circRNF111). Adenoviruses containing green fluorescent protein (sh-control) were employed as the control. Then, sh-circRNF111 or pDC311-miR-143-3p-sponge along with sh-control were inoculated into HEK293 cells. The cloned recombinant adenoviruses in the HEK293 cells were purified and the titer of virus was assessed by plaque assays. The stock solutions of sh-circRNF111 or pDC311- miR-143-3p-sponge and sh-control were made with 1 × 10^11^ plaque formation unit (PFU)/ml.

### Agomirs/Antagomirs Treatment, RNA Interference and IGF2R Overexpression

Agomir-143-3p, antagomir-143-3p, agomir-NC along with antagomir-NC were synthesized by RiboBio (RiboBio, Guangzhou, China) to suppress or trigger miR-143-3p expression, respectively (Krützfeldt et al., 2005; Hu et al., 2014). When differentiated hADSCs attained about 30–40% confluence, either 100nM agomir-143-3p or 200 nM antagomir-143-3p, and the respective controls, were inoculated. After 48 h or 72 h, and re-inoculated every 2 days throughout the stimulation of differentiation. CircRNA expression was repressed by siRNA-mediated silencing. Four different siRNAs were designed and evaluated for circRNF111 (Ribobio), siRNA sequences are listed in Supplementary Table 10. The IGF2R-pcDNA3.1 overexpression plasmid (oe-IGF2R) and GFP-pcDNA3.1 (oe-Vector) control vectors were conducted by Shanghai GeneChem Co., Ltd. (Shanghai, China). Cells transfection with plasmids was achieved using Lipofectamine 3000 transfection reagent (Thermo Fisher). Lipofectamine RNAiMAX transfection reagent (Thermo Fisher) was used for the siRNAs, agomir-143-3p, as well as antagomir-143-3p.

### Dual-Luciferase Reporter Assays

*Homo sapiens* IGF2R (Gene ID: 3482) 3′UTRs harboring predicted the binding sites of miR-143-3p and its mutated forms were amplified using PCR and propagated into the *Xho*I/*Hin*dIII site of the pGL4-basic vector (cat. no., Promega) to create the IGF2R-3′UTR-WT, as well as IGF2R-3′UTR-Mut vectors. The luciferase coding sequence was fused to the 3′UTRs of IGF2R. DNA sequencing was employed to confirm all the PCR products. 5 × 10^4^ 293HEK cells were planted in 24-well plates 24 h prior to transfection. Afterward, 200 ng of construct plasmids (IGF2R-3′UTR-WT and IGF2R-3′UTR-Mut reporter plasmid) along with 100 ng of the *Renilla* luciferase plasmid (pRL-TK serving as an internal standard) expressing *Renilla* luciferase constitutively were co-inserted via co-transfection with Lipofectamine 3000 (cat. no., Invitrogen). 100 nM of agomir-143-3p or 20 nM si-circRNF111 was administered after 6–8 h of culture medium replacement. After the elapse of 48 h following transfection, we harvested the cells and employed the Dual-Luciferase enzyme Reporter Assay System (cat. no., Promega) to assess the luciferase enzyme activity. In all the experiments, cells were inserted with the same plasmids in quadruplicate via transfection, and Luciferase enzyme activities were standardized to the co-inserted pRL-TK plasmid (mean ± S.D.). Primer sets are given in Supplementary Table 10.

### Animals and Animal Treatments

The guidelines of the Animal Care Committee of Zhejiang University were followed in all protocols involving animals. The study mice (male C57BL/6 aged 8 weeks) were commercially provided by Slack Experimental Animal Center of the Chinese Academy of Sciences (Shanghai, China). Afterward, the mice were kept in a distinct pathogen-free animal house. A group of 25 mice were put under a cycle of 12-h lighting-darkness. The study mice were given 1 week to acclimatize under a regular chow diet. After that, the weight of the mice was determined and then they were randomly grouped into two study groups, i.e., 5 in the NCD group (under the normal chow diet consisting of 9.9% fat, 26.18 protein along with 63.92 carbohydrate) and 20 in the HFD group (under high fat diet consisting of the NJ research diets, 45% of fat, 20% protein, and 35% carbohydrate). The mice were fed with the specified diets for 12 weeks under controlled light and temperature conditions. In addition, they freely accessed water. At week 8 of feeding, the mice in both groups were fasted overnight, and then we performed an intraperitoneal glucose tolerance test (GTT). At week of feeding, the mice were fasted for 4 h, and then we performed an intraperitoneal insulin tolerance test (ITT). At week 12 of feeding, mice were sacrificed via cervical dislocation after exsanguination and their body weights were measured. For the other 15 HFD-triggered obese mice, sh-control adenovirus (*n* = 5), sh-circRNF111 adenovirus (*n* = 5), sh-circRNF111 adenovirus combined with miR-143-3p sponge adenovirus (*n* = 5) were inoculated twice weekly through tail vein for another 2 weeks (1 × 10^11^ vg/ml), and then GTT along with ITT were conducted. After 16 weeks, plasma and tissue samples were promptly frozen in liquid nitrogen and kept at −80°C for histological and biochemical analysis. Liver, skeletal muscle, iWAT (inguinal white adipose tissues) along with eWAT (epididymal white adipose tissues) were fixed with 4% formaldehyde, and then embedded in an OCT compound. After that, the tissues were sliced into 4 μm thick slices as per the standard protocol followed by H&E staining of the slices and assessment with a light microscope.

### GTT and ITT

In the GTT assessment, we fasted the mice overnight, followed intraperitoneal administration of 1.5 g/Kg of glucose. After that, One Touch Ultra glucose strips (LifeScan, Malvern, PA, United States) were employed to determine the blood glucose content at specified time points, i.e., 0, 15, 30, 60, and 120 min. In the ITT test, we fasted the mice for 4 h, followed by intraperitoneal administration of 0.5 U/kg insulin (cat. no., Eli Lilly Company, United States). Subsequently, blood glucose content was determined with the above approach.

### Statistical Analysis

In cross-sectional analysis, we tested all the continuous variables for a normal distribution. The normally distributed variables are given as mean ± S.D. Variables exhibiting a skewed distribution are given as the median (interquartile range, 25–75%) and were transformed logarithmically to attain normal distribution prior to analysis. Categorical variables are given as frequencies along with percentages. Differences in the baseline features between study subjects with and without Mets were analyzed by using *t*-test for continuous variables and by using Chi-square test for categorical variables. Spearman correlations between serum or urine circRNF111 and metabolic parameters before and after adjustment for age, smoking, gender, as well as drinking status were determined by using Spearman correlation in the cross-sectional analysis. The Chi-square was employed to analyze the incidence of MetS at different levels of serum or urine circRNF111. Logistic regression along with multiple stepwise regression analyses considering a serum or urine circRNF111 contents as a dependent variable after adjustment for drinking status, gender, smoking status, and age, were carried out in the prospective analysis to explore the effects of variables on MetS risk, HOMA-IR, as well as other metabolic subgroups (low HDL-c, leveled BP, hyperglycemia, central obesity, and hypertriglyceridemia) linked to serum and urine circRNF111 contents. Statistical analyses were implemented in SPSS 22.0 (IBM, United States), as well as GraphPad Prism 5 for Microsoft Windows. For animal or cell experiments, independent two-sided Student’s *t*-test and one-way ANOVA were employed to analyze two or multiple groups, respectively. Each experiment was replicated thrice. Data are given as the mean ± SEM (*in vivo* studies) or mean ± S.D. (*in vitro* studies). Results are representative of three independent experiments. *P* < 0.05 signified statistical significance.

## Results

### Profiling of circRNAs in Serum of MetS and Control Samples

**FIGURE 1:**
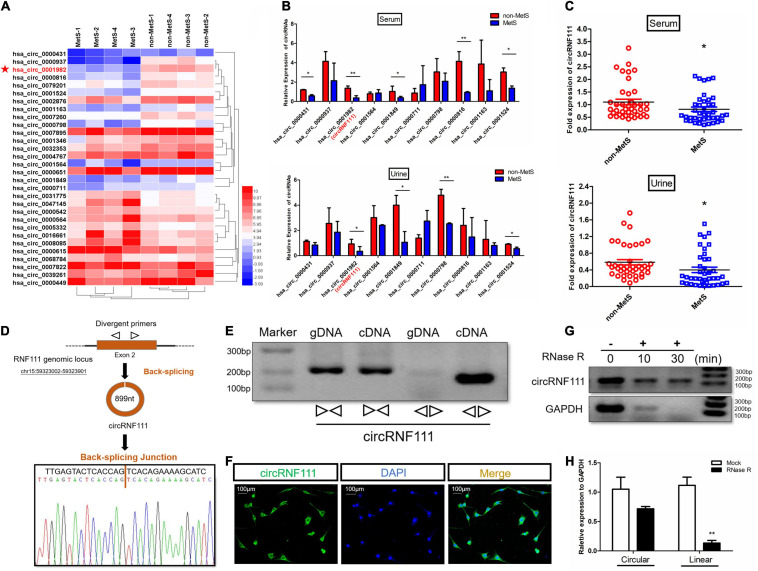
CircRNF111 validation and expression in MetS human serum and urine samples. **(A)** Heat map illustrating all the circRNAs differentially expressed between MetS (*n* = 4) and non-MetS (*n* = 4) human serum samples. **(B)** The expression of 10 candidate circRNAs in individuals with MetS in contrast with healthy individuals in human serum, as well as human urine samples. *n* = 3 (three separate experiments). *p < 0.05. **(C)** CircRNF111 expression was lower in Individuals with MetS (*n* = 40) in contrast with healthy individuals (*n* = 40) in human serum, as well as human urine samples. *p < 0.05. **(D)** Scheme illustrating RNF111 exons 2 circularization to produce circRNF111 (black arrow). CircRNF111 presence was verified using RT-PCR, and subsequent Sanger sequencing. Black arrow designates head-to-tail circRNF111 splicing sites. **(E)** CircRNF111 presence was confirmed in hADSCs via RT-PCR. CircRNF111 was amplified from cDNA with divergent primers, but not from gDNA. **(F)** RNA FISH illustrating the dominant circRNF111 localization in the cytoplasm. Cy-3 was employed to label circRNF111 probes. DAPI staining of the nuclei was carried out. Scale bar, 100 μm. **(G)** After total RNA samples were treated with or without RNase R for the three time-points in hADSCs, PCR was performed to detect circRNF111 and GAPDH, indicating that circRNF111 was more resistant to RNase R digestion than GAPDH. **(H)** RT-qPCR investigation of above circRNF111 and RNF111 mRNA expressions in hADSCs inoculated with or without RNase R. CircRNF111 and RNF111 mRNA relative levels were normalized to the values determined in mock treatment. **p < 0.01.

Deep RNA sequencing of ribosomal RNA-digested total RNA from four MetS serum and four non-MetS control serum samples was employed to characterize the cirRNA mRNAs. The Illumina HiSeq procedure was employed to prepare a library deep sequenced on the Illumina HiSeq 2000 platform to generate a depth averaging 30 M aligned reads per sample.TopHat2 (Kim et al., 2013) along with STAR (Dobin et al., 2013) were employed to align the sequence reads to the hg19/GRCh37 human genome. Overall, 4791 circRNAs were uncovered and a significant number of the identified circRNAs were supported by more than 10 reads as indicated in the Supplementary Figure 1A. RefSeq data resource (O’Leary et al., 2016; Haft et al., 2018) was employed to annotate the identified candidates, and more than 80% of the circRNAs constituted protein-coding exons, while smaller proportions aligned with introns, lncRNAs and antisense regions as illustrated in the Supplementary Figure 1B. Most of circRNAs were less than 1,500 nucleotides (nt) long, with a median length of about 500 nt as exhibited in the Supplementary Figure 1C. The chromosome distribution of the discovered circRNAs exhibited no remarkable difference between MetS serum and control samples as illustrated in Supplementary Figures 1D,E. We further investigated circRNAs’ number in their host genes and the results illustrated that one gene could generate numerous circRNAs as indicated in Supplementary Figure 1F. Analysis of circRNAs abundance within one gene locus demonstrated that there is usually one dominantly expressed circRNA isoform from one gene locus (Supplementary Figure 1G). MetS serum and control samples showed differential expression patterns of circRNAs and we identified the 30 most differentially expressed circRNAs (Figure 1A, Supplementary Figure 1H, and Supplementary Table 1). To verify the RNA deep sequencing data, RT-qPCR was carried out to confirm 10 downregulated circRNAs of hsa_circ_0000431, hsa_circ_0000937, hsa_circ_0001982, hsa_circ_0001564, hsa_circ_0001849, hsa_circ_0000711, hsa_circ_0000798, hsa_circ_0000816, hsa_circ_0001163 and hsa_circ_0001524 both in MetS serum and urine samples (Figure 1B). In the subsequent step, we identified and characterized one potential protective function of hsa_circ_0001982, which is produced by the circularization of exon 2 of RNF111 gene, thereafter termed circRNF111 (Supplementary Figure 1I).

### CircRNF111 Is Downregulated in MetS Serum and Urine Samples and Is Localized in the Cytoplasm

We further validated the expression of circRNF111 from another individual serum, as well as urine samples from 40 MetS study subjects and 40 non-MetS control participants via RT-qPCR. The data illustrated that circRNF111 expression was downregulated in the Individuals with MetS in contrast with healthy individuals in serum, as well as urine samples (*p* < 0.05) (Figure 1C). Given the different circRNF111 expression in Individuals with MetS and non-MetS controls, we speculated that circRNF111 might be a target that participates in MetS. Hence, a set of investigations were conducted as in the previously described methodology (Memczak et al., 2013; Jeck and Sharpless, 2014) to explore whether circRNF111 is a circular transcript, but not linear transcript. Sanger sequencing was used to verify the head-to-tail splicing in the RT-qPCR products of circRNF111 determined by its expected size (Figure 1D). Convergent along with divergent primers were designed to amplify RNF111 mRNA and circRNF111, respectively, using cDNA and not genomic DNA (gDNA) as indicated in Figure 1E. RNA FISH data illustrated the abundant expression of cytoplasmic circRNF111 in hADSCs preadipocytes (Figure 1F). Besides, RNase R was additionally employed to confirm circRNF111 circular form, given that RNase R digests only linear RNA and not circRNAs over very short time (Xiao and Wilusz, 2019). The PCR along with qRT-PCR data validated that circRNF111 was highly resistant to RNase R denaturation in contrast with its linear form as indicated in Figures 1G,H. After total RNA samples were treated with or without RNase R for the three time-points of 0,10, and 30 min in hADSCs, PCR was performed to detect circRNF111 and GAPDH, the results indicating that circRNF111 was more resistant to RNase R digestion than GAPDH. Hence, these data suggest that circRNF111 is a circularization of single-stranded RNA molecules that is formed from RNF111 via a back-splicing event of one or two exons.

### CircRNF111 Regulates Insulin Sensitivity and Lipid Deposition *in vitro*

**FIGURE 2:**
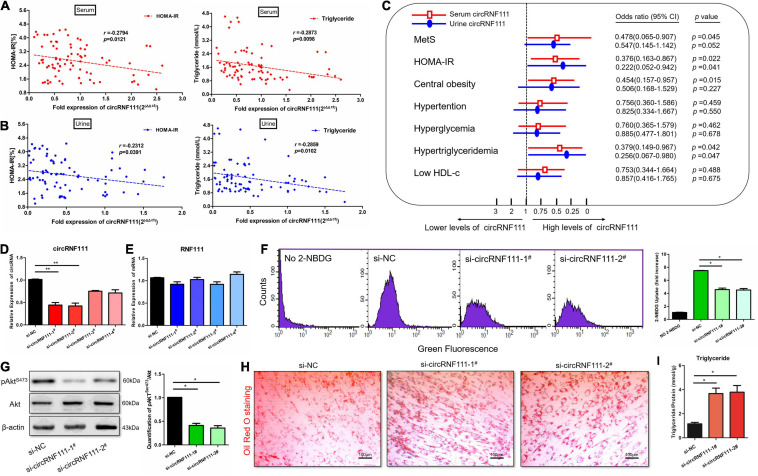
Knockdown of circRNF111 impairs insulin sensitivity and accelerates lipid accumulation *in vitro*. **(A)** Scatterplot along with Pearson correlation of human serum circRNF111 contents with HOMA-IR (homeostasis model assessment of insulin resistance) and the content of triglycerides were performed on MetS and non-MetS samples; *n* = 80, *p* < 0.05. **(B)** Pearson correlation of human urine circRNF111 contents with HOMA-IR and triglycerides were performed in MetS and non-MetS samples; *n* = 80, *p* < 0.05. **(C)** Multiple stepwise logistic regression of HOMA-IR, MetS, as well as other metabolic subgroups (hypertriglyceridemia, central obesity, hyperglycemia, low HDL-c, and hypertension) linked to human urine and serum circRNF111 contents. *n* = 80, *p* < 0.05. **(D,E)** RT-qPCR evaluation of circRNF111 and RNF111 mRNA contents in differentiated hADSCs transfects of four circRNF111 siRNA or negative control siRNA at a final level of 20 nM at 48 h. GAPDH served as the normalization standard. **p < 0.01. **(F)** Flow cytometry images illustrating glucose uptake in differentiated hADSCs preadipocytes at wavelengths of excitation and emission of 485 and 535 nm, respectively. Right panels are the stoichiometric results, *p < 0.05. **(G)** Western blotting assessment of pAkt^*Ser*473^ in differentiated hADSCs pre-adipocytes inoculated with circRNF111 siRNA or negative control siRNA, β-actin served as the loading standard. Right panels designate the stoichiometric data of the pAkt^*Ser*473^ along with of Akt (pAkt^*Ser*473^/Akt) protein expression *p < 0.05. **(H)** Images illustrating Oil Red O staining in differentiated hADSCs inoculated with circRNF111 siRNA or negative control siRNA, scale bar, 100 μm. **(I)** Histogram illustrating triglyceride assay of the contents of intracellular triglycerides after treatment with circRNF111 siRNA or negative control siRNA, *p < 0.05.

Associations between circRNF111 content and factors linked to lipid profiles, adiposity, hepatic enzyme function, and insulin resistance were investigated from the above serum, as well as urine samples from 40 individuals with MetS and 40 non-MetS healthy subjects. Clinical features of the subjects included in the verification study are indicated in the Supplementary Table 2. Pearson correlation assessment of urine and serum circRNF111 contents with metabolic risk were carried out as indicated in Supplementary Tables 3, 4. The results revealed that serum and urine circRNF111 contents were negatively linked to HOMA-IR (homeostatic model assessment of insulin resistance) and the content of triglyceride. The relevant indices of serum along with urine circRNF111 with HOMA-IR were: *r* = −0.2794 and *r* = −0.2312, respectively, both *p* < 0.05. The relevant indices of serum along with urine circRNF111 with triglyceride (TG) were: *r* = −0.2873 and *r* = −0.2859, respectively, both *p* < 0.05 (Figures 2A,B). Elevated urine and serum circRNF111 contents were coupled by a decreasing pattern in the HOMA-IR, as well as the content of triglyceride.

Subsequently, multiple stepwise regression assessment using urine or serum circRNF111 content as an dependent variable after adjusting for gender, smoking status, age, and drinking status illustrated that serum circRNF111 contents were linked independently to the TG, HOMA-IR and visceral fat area (VFA), for standardized β = −0.582, −0.286, and −0.275, respectively, and that urine circRNF111 contents were independently linked to HOMA-IR, Body Fat (%) and TG, for standardized β = −0.525, −0.308, and −0.107, respectively; all *p* < 0.05 as indicated in the Supplementary Tables 5, 6. After adjusting for confounding factors, circRNF111 was illustrated as an independent protective factor of insulin resistance; OR = 0.376, 95% CI = 0.163–0.867 for serum circRNF111 content, and OR = 0.222, 95% CI = 0.052–0.942 for urine circRNF111 content, both *p* < 0.05. CircRNF111 was also determined to be an independent protective factor for hypertriglyceridemia; OR = 0.379, 95% CI = 0.149–0.967 for serum circRNF111 content, and OR = 0.256, 95% CI = 0.067–0.980 for urine circRNF111 content, both *p* < 0.05 (Figure 2C). The results illustrated that circRNF111 reduction may induce insulin resistance and lipid aggregation.

Furthermore, to validate these data, hADSCs were induced to differentiate into mature adipocytes using an adipogenic induction cocktail methods (Hu et al., 2018). We then transfected differentiated hADSCs with four circRNF111 small-interfering RNA, two of which effectively knocked down the expression of circRNF111 by qRT-PCR assay (Figure 2D). Silencing of circRNF111 expression had no impact on RNF111 mRNA content (Figure 2E). We then explored the influence of circRNF111 inhibition on insulin signaling along with glucose uptake in differentiated hADSCs. A fluorescent 2-NBDG screening approach (i.e., flow cytometry) was employed to assay for glucose uptake in which differentiated hADSCs were inoculated with siRNAs in the presence of 60 μM 2-NBDG for 1 h. As shown in Figure 2F, knockdown of circRNF111 remarkably reduced 2-NBDG uptake in differentiated hADSCs, *p* < 0.05 (Figure 2F). Besides, we examined the effects of circRNF111 inhibition exerted on Akt activity by analyzing phospho-Akt expression in differentiated hADSCs via Western blotting. Congruent with our glucose uptake assay data, knockdown of circRNF111 decreased Akt phosphorylation in differentiated hADSCs as indicated in Figure 2G. The Oil Red O staining assay was performed on day 10 after differentiation of hADSCs. The results showed that down-regulation of circRNF111 could generate excess lipid droplet deposition in differentiated hADSCs Figure 2H. Moreover, intracellular triglyceride contents were explored with triglyceride assays which verified the lipid aggregation after circRNF111 inhibition (Figure 2I). Together, these data demonstrate that silencing of circRNF111 impairs insulin sensitivity and accelerates lipid accumulation.

### CircRNF111 Serves as Sponge for miR-143-3p

**FIGURE 3:**
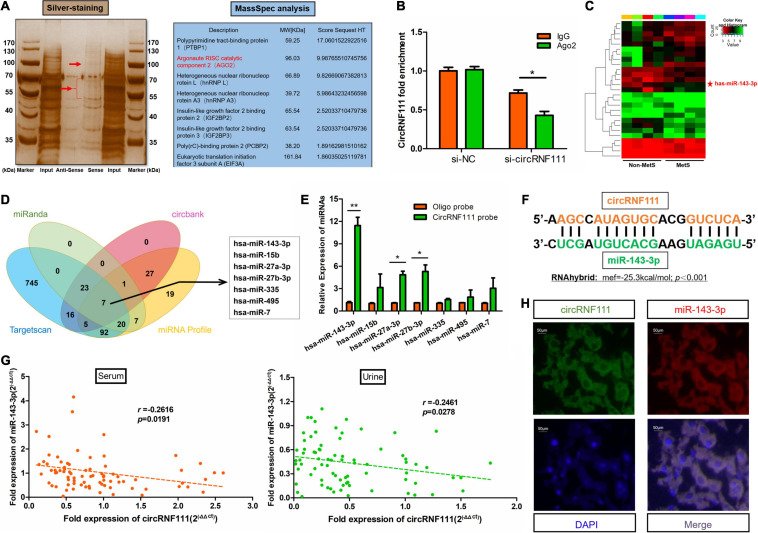
CircRNF111 sponges miR-143-3p. **(A)** RNA pull-down assay with a biotinylated circRNF111 probe followed by silver staining was shown. Subsequent mass spectrometry (MS) analysis revealed several RBPs including AGO2 were captured by biotinylated circRNF111 in hADSCs. **(B)** AGO2 RNA immunoprecipitation (RIP) assay was done to explore circRNF111 contents in hADSCs inoculated with si-circRNF111. Data are given as the mean ± SD of 3 experiments. *p < 0.05. **(C)** miRNA microarray of the miRNA-mediated differential dysregulation of miRNAs in MetS and non-MetS serum samples (*n* = 4). **(D)** Scheme illustrating overlapping of the target miRNAs of circRNF111 predicted by miRanda, circBank, TargetScan, as well as miRNAs upregulated in MetS determined by microarray analysis. **(E)** RT-qPCR evaluation of circRIP assay of the lysates prepared from hADSCs. Comparison of circRNF111 contents normalized to the input contents. Results are given as the mean ± SD of 3 experiments. *p < 0.05 versus control (lac Z) probe. **(F)** RNAhybrid was used as a tool for predicting miRNA binding sites between circRNF111a and miR-143-3p and to measure the binding accessibility and energy, *p* < 0.001. **(G)** Scatterplot along with the Spearman correlation of circRNF111 contents with miR-143-3p were performed in MetS and non-MetS serum, as well as urine samples; *n* = 80, both *p* < 0.05. **(H)** Images illustrating RNA FISH evaluation of co-localization of circRNF111 along with miR-143-3p in the differentiated hADSCs mature adipocytes. Alexa fluor 488 was employed to label miR-143-3p probes, while Cy3 was employed to label circRNF111 probes. DAPI staining of the nuclei was conducted. Scale bar, 50 μm.

Recent research evidence documented that numerous abundant circRNAs can function as miRNA sponges (Hansen et al., 2013). Given that circRNF111 is predominantly a cytoplasm-localized and exhibited stability, we further explored whether circRNF111 could act as ceRNA capable of sponging miRNAs in MetS. An RNA-Protein pull-down assay with a biotinylated circRNF111 probe followed by mass spectrometry (MS) analysis were carried out. Several RNA-binding proteins as well as AGO2 (argonaute RISC catalytic component 2) were captured by biotinylated circRNF111 in hADSCs (Figure 3A and Supplementary Table 7). Next, RIP was carried out and the data illustrated that endogenous circRNF111 pulled down from AGO2 antibodies were remarkably increased in contrast with circRNF111-depleted cells (Figure 3B).

Comparative expression of circulating miRNAs between individuals with MetS and healthy individuals was screened using microarray as reported in our previous work (Xihua et al., 2019) (Supplementary Table 8). In all, 27 miRNAs were determined to be differentially expressed in the serum of individuals with MetS in contrast with healthy individuals through microarray analysis with a fold change > 2 (*p* < 0.05) as illustrated in Figure 3C. Three data resources (miRanda, circBank, and TargetScan) were employed to predict the possible target miRNAs (Das, 2012). Finally, seven miRNAs were screened out from the overlap between these data resources and miRNA microarray (Figure 3D and Supplementary Table 9). The quantities of the seven candidate miRNAs were assessed via circRNAs *in vivo* precipitation assay (circRIP) (Han et al., 2017). As shown in Figure 3E, miR-143-3p were remarkably increased by biotinylated circRNF111 probe, *p* < 0.01. RNA hybrid (Krüger and Rehmsmeier, 2006) was employed to predict the binding sites between circRNF111 and miR-143-3p and to quantitatively measure the binding energy (Figure 3F). Next, the relationship of miR-143-3p with circRNF111 was assessed using Spearman correlation analyses. The level of miR-143-3p exhibited some degree of negative relationship with the level of circRNF111 both in serum samples (*r* = −0.2616, *p* = 0.0191) and in urine samples (*r* = −0.2461, *p* = 0.0278) (Figure 3G). Furthermore, the RNA FISH assay revealed that circRNF111 along with miR-143-3p were co-localized in the cytoplasm of the differentiated hADSCs mature adipocytes as indicated in Figure 3H, illustrating that miR-143-3p can be sponged by circRNF111. Studies have revealed that miR-143 was involved in the regulation of several important biochemical processes (Surgucheva et al., 2013).

### CircRNF111 Participates in MetS by Targeting miR-143-3p Expression

**FIGURE 4:**
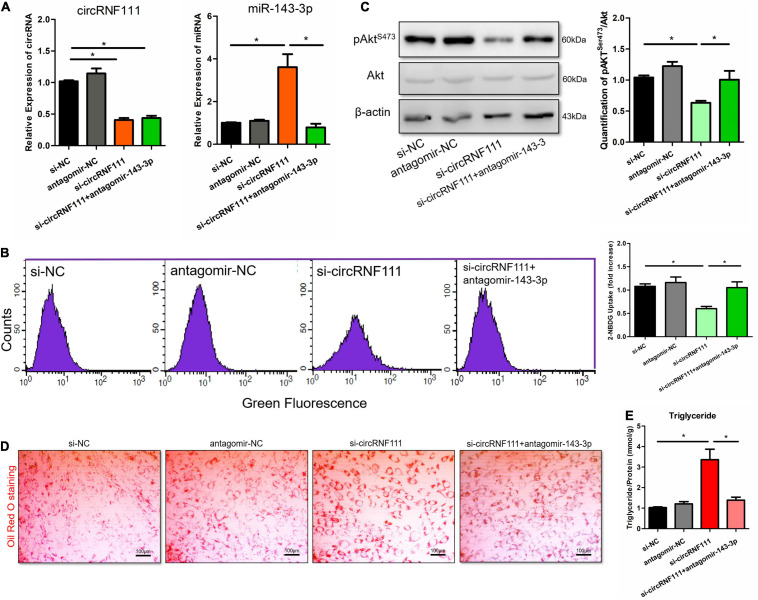
Silencing miR-143-3p expression reverts the circRNF111 function in differentiated hADSCs. **(A)** Differentiated hADSCs were co-transfected with si-circRNF111 (20 nM) and antagomir-143-3p (200 nM) in contrast with the cells transfected with si-circRNF111 alone. After 48 h of transfection, circRNF111 and miR-143-3p contents were measured by RT-qPCR and normalized to GAPDH or U6 level. *p < 0.05. **(B)** Flow cytometry evaluation via a fluorescent 2-NBDG approach illustrating that diminished expression of both circRNF111 and miR-143-3p led to increased glucose uptake in contrast with the inhibition of circRNF111 alone. Fluorescence was determined at wavelengths of excitation and emission of 485 and 535 nm, respectively. Right panels are the stoichiometric results, *p < 0.05. **(C)** Western blotting evaluation of pAkt^*Ser*473^ and Akt in differentiated hADSCs co-transfected with si-circRNF111 and antagomir-143-3p in contrast with the cells transfected with si-circRNF111 alone, β-actin served as the loading standard. Right panels designate the stoichiometric data of the pAkt^*Ser*473^ along with Akt (pAkt^*Ser*473^/Akt) protein expression *p < 0.05. **(D)** Images illustrating Oil Red O staining in differentiated hADSCs inoculated with circRNF111 and miR-143-3p expression inhibitors in contrast with the cells inoculated with circRNF111 inhibitor, scale bar, 100 μm. **(E)** Histogram illustrating intracellular triglyceride contents assessed via triglyceride assay after co-transfected with si-circRNF111 and antagomir-143-3p in contrast with the cells inserted with si-circRNF111 alone, *p < 0.05.

To assess if circRNF111 affected the biological role in MetS by sponging miR-143-3p, we co-inserted differentiated hADSCs with si-circRNF111 and miR-143-3p antagomir (antagomir-143-3p) via co-transfection (Figure 4A). Antagomirs are referred to as blockmirs or anti-miRs, they are a group of chemically engineered oligos that repress other molecules from docking to a desired site on an RNA molecule (Krützfeldt et al., 2005). Therefore, antagomirs are employed to silence endogenous miRNAs[]. As illustrated in Figure 4B, the silencing of circRNF111 expression reduced the fluorescent 2-NBDG glucose uptake in differentiated hADSCs whereas this reduction was remarkably rescued by the repression of the expression of both miR-143-3p and circRNF111. The data illustrated that the phosphorylation of Akt in differentiated hADSCs increased in the cells co-inserted with si-circRNF111 and antagomir-143-3p as in contrast with the cells inserted with si-circRNF111 alone, *p* < 0.05 (Figure 4C). The Oil Red O staining analysis illustrated that additional deposition of lipid droplets in differentiated hADSCs escalated in the cells with circRNF111 and miR-143-3p expression repressors in contrast with the circRNF111-depletion cells inoculated with inhibitor (Figure 4D). Moreover, triglyceride content analysis confirmed that intracellular triglyceride contents were decreased in the cells co-inserted with si-circRNF111 and antagomir-143-3p as in contrast with the cells inserted with si-circRNF111 alone, *p* < 0.05 as illustrated in Figure 4E. Taken together, these results suggest that circRNF111 alleviates insulin sensitivity and lipid deposition by targeting miR-143-3p *in vitro*.

#### CircRNF111 Protects Against Insulin Resistance and Lipid Deposition via IGF2R

**FIGURE 5:**
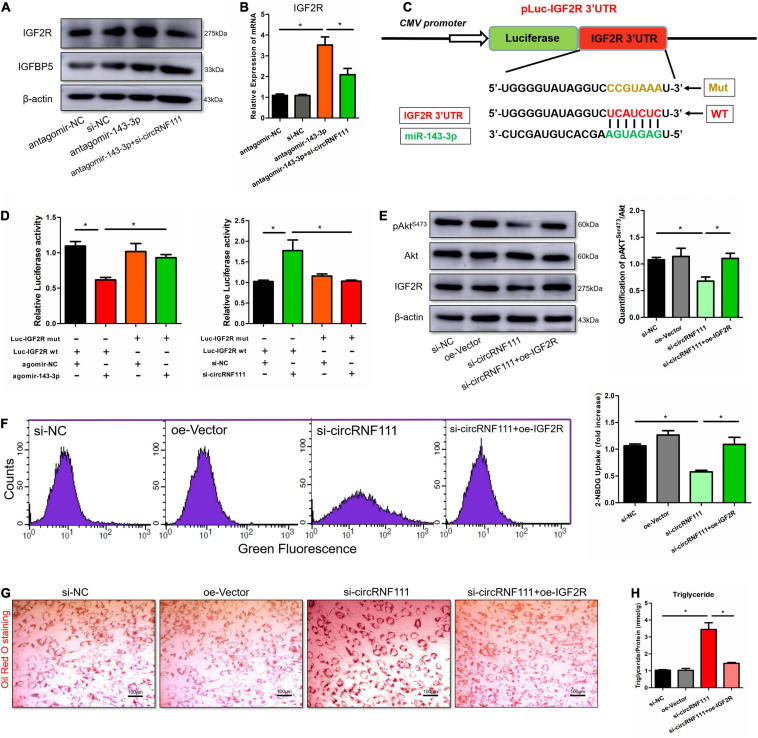
Overexpression of IGF2R expression reverses the circRNF111 function in differentiated hADSCs. **(A)** Western blotting assessment of IGF2R along with IGFBP5 in hADSCs preadipocytes co-transfected with antagomir-143-3p and si-circRNF111 in contrast with the cells inserted with antagomir-143-3p alone, β-actin was employed as a loading standard. **(B)** RT-qPCR evaluation of IGF2R mRNA contents in hADSCs co-transfects of antagomir-143-3p and si-circRNF111 in contrast with the cell transfects of antagomir-143-3p alone, relative IGF2R mRNA levels were normalized to GAPDH. Results are given as the mean ± SD for three experiments. *p < 0.05. **(C)** Scheme exhibiting the complementary sequence between IGF2R and miR-143-3p. IGF2R 3′UTR mutated nucleotides are indicated in red letters. **(D)** HEK293 cells were co-transfected with agomir-143-3p or si-circRNF111 and a luciferase reporter construct harboring mutated (Mut) or wild-type (WT) IGF2R 3′UTR. The luciferase enzyme activities in the cells were explored, results are shown as the mean ± SD for 3 experiments. *p < 0.05. **(E)** Representative western blotting assays of pAkt^*Ser*473^, Akt and IGF2R in differentiated hADSCs adipocytes co-transfected with si-circRNF111 and IGF2R overexpression plasmid in contrast with the cells transfected with si-circRNF111 alone, β-actin served as the loading standard. Right panels exhibit the stoichiometric results of the pAkt^*Ser*473^ along with Akt (pAkt^*Ser*473^/Akt) protein contents, *p < 0.05. **(F)** Overexpression of IGF2R in si-circRNF111 treated cells resulted in an increased glucose uptake via a fluorescent 2-NBDG screening approach by flow cytometry than those observed with the inhibition of circRNF111 alone. Fluorescence was determined at wavelengths of excitation and emission of 485 and 535 nm, respectively. Right panels are the stoichiometric results, *p < 0.05. **(G)** Images illustrating Oil Red O staining in differentiated hADSCs inoculated with si-circRNF111 and oe-IGF2R plasmid in contrast with the cells inoculated with circRNF111 inhibitor alone, scale bar, 100 μm. **(H)** Histogram exhibiting intracellular triglyceride contents evaluated with the triglyceride assay after co-transfected with si-circRNF111 and oe-IGF2R plasmid in contrast with the cells inserted with si-circRNF111 alone, *p < 0.05.

In our previous work, we have defined miR-143-3p as an integrator of insulin signaling pathways in both MetS patients and mouse models of obesity. MiR-143-3p silencing participates in the development of obesity-mediated insulin resistance, glucose tolerance and lipid accumulation and characterizes the miR-143-3p-IGF2R cascade as a possible target of treating obesity-linked metabolic diseases (Xihua et al., 2019). Therefore, we next evaluated the relationship between circRNF111 and IGF2R *in vitro*. Downregulation of miR-143-3p using antagomir-143-3p induced the protein levels of IGF2R, this corresponded to an increase in the content of IGF2R mRNA, and inhibition of circRNF111 reversed these effects (Figures 5A,B). Moreover, we developed reporter constructs where the luciferase encoding sequence was incorporated to the 3′UTRs of IGF2R, a known miR-143-3p target sites (IGF2R 3′UTR-WT), as well as the mutated forms of the IGF2R 3′UTR, where the miR-143-3p seed binding sites was obliterated (IGF2R 3′UTR-Mut) (Figure 5C). Consistent with our previous studies, we documented reduced luciferase enzyme activity of the IGF2R 3′UTR-WT but not IGF2R 3′UTR-Mut in the presence of miR-143-3p using agomir-143-3p. As expected, circRNF111 inhibition enhanced luciferase enzyme activity of the IGF2R 3′UTR-WT and not IGF2R 3′UTR-Mut (Figure 5D).

To further investigate whether circRNF111 protects against insulin resistance and lipid deposition by targeting IGF2R, an IGF2R overexpression plasmid (oe-IGF2R) and control vector (oe-Vector) was constructed and transfected into si-circRNF111 treated hADSCs. The western blotting data illustrated that oe-IGF2R was able to partially revert the loss of the expression of the phosphorylation of Akt and IGF2R in hADSCs which was caused by the circRNF111 knockdown (Figure 5E). This effect was further confirmed via the fluorescent 2-NBDG glucose uptake assay and the Oil Red O staining assay. Functionally, the glucose uptake ability was obviously ameliorated by oe-IGF2R in si-circRNF111 treated cells (Figure 5F). The extra-deposition of lipid droplets in differentiated hADSCs increased after circRNF111 inhibition and reversed by oe-IGF2R transfection (Figure 5G). Additionally, the triglyceride content analysis confirmed that intracellular triglyceride contents were reduced in the cells co-transfected with si-circRNF111 and oe-IGF2R as in contrast with the cells transfected with si-circRNF111 alone (Figure 5H). Altogether, these findings suggest that knockdown of circRNF111 impairs insulin sensitivity and accelerates lipid accumulation of differentiated hADSCs mainly by targeting IGF2R as miR-143-3p sponge.

### CircRNF111 Functions as miR-143-3p Sponge to Protect Against Insulin Resistance and Lipid Deposition *in vivo*

**FIGURE 6:**
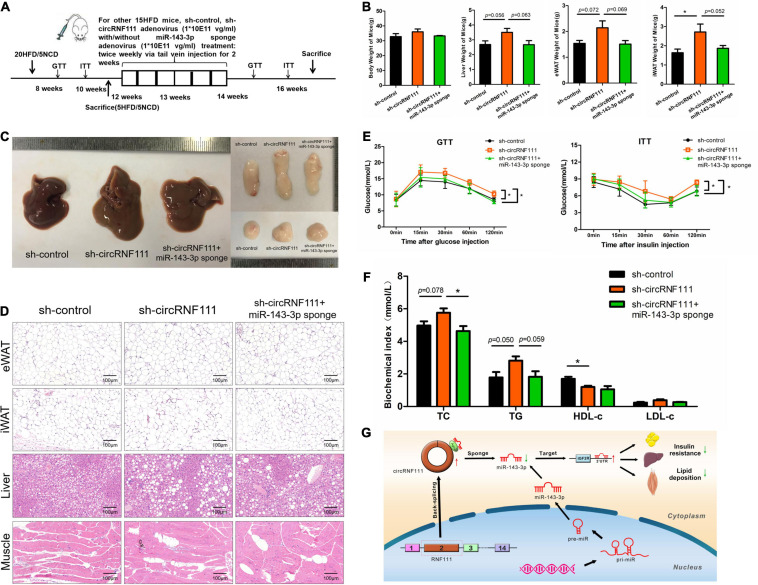
CircRNF111 function as miR-143-3p sponge *in vivo*. **(A)** The schematic diagram of mice treatment process via injection through the tail vein injection of sh-control adenovirus (*n* = 5), sh-circRNF111 adenovirus (*n* = 5), sh-circRNF111 adenovirus combine with miR-143-3p sponge adenovirus (*n* = 5) of the 12 weeks’ HFD-triggered obese mice twice weekly during 2 weeks. **(B)** After 16 weeks, liver tissues, eWAT (epididymal white adipose tissue) and iWAT (inguinal white adipose tissue) of mice were dissected. Body weight, eWAT weight, liver weight along with iWAT weight of obese mice was injected with sh-control adenovirus (*n* = 5), sh-circRNF111 adenovirus (*n* = 5), sh-circRNF111 adenovirus combine with miR-143-3p sponge adenovirus (*n* = 5) were compared. *p < 0.05. **(C)** After 16 weeks, liver, eWAT, and iWAT of mice were dissected and photographed. The representative photographs were shown. **(D)** Hematoxylin/Eosin representative staining of adipocytes in iWAT, eWAT, liver, and muscle. **(E)** Intraperitoneal GTT (glucose tolerance test) results and Intraperitoneal ITT (insulin tolerance test) results. Blood glucose was assessed at the specified times between obese mice which injected with sh-control adenovirus (*n* = 5), sh-circRNF111 adenovirus (*n* = 5), sh-circRNF111 adenovirus combine with miR-143-3p sponge adenovirus (*n* = 5) were compared., *p < 0.05. **(F)** Plasma TC, LDL-c, TG, and HDL-c contents were detected after injection of sh-control adenovirus (*n* = 5), sh-circRNF111 adenovirus (*n* = 5), sh-circRNF111 adenovirus combine with miR-143-3p sponge adenovirus. *p < 0.05. **(G)** Scheme illustrating circRNF111/miR-143-3p/IGF2R axis. Our data illustrate a vital role of the novel circRNA-circRNF111 in the progress of MetS. CircRNF111 inhibition enhances insulin resistance and lipid deposition in MetS through regulating miR-143-3p-IGF2R cascade. It offers a prospective effective treatment approach of MetS progression.

To explore the effects of circRNF111 and miR-143-3p *in vivo*, sh-circRNF111 recombinant adenovirus and miR-143-3p sponge adenovirus were constructed and packaged. As illustrated in the schematic diagram, sh-circRNF111 adenovirus with or without miR-143-3p sponge adenovirus was administered into the tail vein of the high-fat diet (HFD)-triggered obese mice twice weekly for 2 weeks (Figure 6A and Supplementary Figure 2). Obese mice administered with sh-circRNF111 consistently exhibited about a 50% decrease in circRNF111 contents and nearly a two-fold increase in miR-143-3p contents in contrast with the sh-control group or sh-circRNF111 + miR-143-3p sponge group in eWAT, iWAT, liver tissues, skeletal muscle tissues and serum samples (*p* < 0.05) (Supplementary Figure 3). There was a tendency to gain weight of eWAT, iWAT, and liver tissues in the circRNF111 knockdown group mice and the increasing tendency was partly reversed by miR-143-3p sponge treatment, but there were no dramatic changes in the body weight of the mice (Figures 6B,C). Supporting this result, Hematoxylin/Eosin staining of eWAT, iWAT, liver and skeletal muscle tissues revealed that obese mice co-injected with sh-circRNF111 adenovirus and miR-143-3p sponge adenovirus had smaller adipocytes in eWAT and iWAT, as well as reduced heterotopic lipid accumulation in liver and skeletal muscle tissues in contrast with sh-circRNF111 adenovirus injected mice alone (Figure 6D).

Furthermore, HFD-fed obese mice inoculated with sh-circRNF111 adenovirus had a remarkably decreased glucose tolerance along with insulin sensitivity tin contrast with the sh-control group or sh-circRNF111 + miR-143-3p sponge group (Figure 6E). Besides, we assessed the plasma TC, TG, HDL-c, as well as LDL-c contents to explore the influence of circRNF111 sponging miR-143-3p in dyslipidemia triggered by obesity in mice. The results showed that the plasma TC and TG contents were increased after sh-circRNF111 adenovirus injection and were reduced with co-injection of miR-143-3p sponge adenovirus. Knockdown of miR-143-3p also reversed the HDL-c level-decrease induced by circRNF111 inhibition (Figure 6F). Taken together, our results revealed that knockdown of circRNF111 impairs insulin sensitivity and accelerates ectopic lipid deposition of obesity-induced metabolic syndrome via sponging miR-143-3p (Figure 6G).

## Discussion

CircRNAs are generated via a non-canonical splicing in eukaryotes with tissue distinct and developmental stage distinct expression trends. CircRNAs have been documented to exist in stable forms in body fluids, e.g., urine, serum, plasma, as well as exosomes (Li et al., 2015; Dou et al., 2016), as indicated by the observation of circCNOT2 in the plasma of individuals with breast (Smid et al., 2019), as well as the presence of circKLDHC10 in serum exosomes obtained from individuals with colorectal cancer (Li et al., 2015). Investigations have documented that circRNAs are promising diagnostic, prognostic, as well as predictive biomarkers for various cancers and diseases. Current investigations have found that circRNAs participate in the pathological processes of cardiovascular, digestive system, nervous, skin, and musculoskeletal systems (Kristensen et al., 2019). There is no systematical investigation of any circRNAs in obesity or obesity associated metabolic disease, such as Metabolic Syndrome (MetS), dyslipidemia, hyperglycemia, fatty liver disease, lipid ectopic deposition or insulin resistance, however. Herein, we first exhibited the profiling of circRNAs in serum of MetS and control samples by circRNA deep sequencing and first determined circRNF111 as a core downregulated circRNA that participates in MetS. Besides, we employed loss-of-function methods to illustrate the involvement of circRNF111 in protecting the promotion of insulin sensitivity and retarding lipid deposition. Hence, elucidation of the cellular mechanisms of circRNF111 in MetS may unearth a novel strategy for treating MetS.

CircRNF111 is formed via the back-splicing of the exons 2 of the RNF111 gene. RNF111, ring finger protein 111, also known as Arkadia, is a novel SUMO-targeted ubiquitin ligase that activates the transforming growth factor β pathway involved in poly-sumoylated degradation (Sriramachandran et al., 2019). RNF111 in humans has numerous SUMO interaction motifs for recognizing substrates harboring poly-SUMO chains, particularly selecting substrates harboring SUMO1-capped SUMO2/3 hybrid conjugates and targets them for proteasomal degradation (Sun et al., 2014). RNF111 expression was remarkably high in the cytoplasm but dramatically low in nucleus of cholangiocytes, as well as hepatocytes. The RNF111 protein contents and the mRNA contents were remarkably different in fibrotic liver samples in contrast with in non-fibrotic liver samples, indicating that RNF111 has a core role in the pathogenesis and progress of hepatic fibrosis (Hou et al., 2018). However, its role in the modulation of glucose, as well as lipid metabolism in health or disease remain unclear. Herein, circRNF111 inhibition had no impact on RNF111 expression, illustrative of the independent role of circRNF111 in the progress of MetS.

Only more recently, when circRNAs have become the center of functional studies, has the mechanisms behind their turnover begun to be unraveled. To date, the biological roles have only been studies for a small proportion of identified circRNAs, one of which have been opined to act as miRNA sponges in the cytoplasm (Zheng et al., 2016). The overall pathophysiological contributions of circRNAs to glucose and lipid metabolism remain largely unknown. Fang et al. (2018) have screened for circRNAs and validated circANKRD36 as an inflammation regulator in patients with type 2 diabetes. Recently, Stoll et al. (2018) employed microarray profiling and determined thousands circRNAs in human pancreatic islets, in which circHIPK3 along with ciRS-7/CDR1as were confirmed to be dysregulated in type 2 diabetes mouse models. They investigated if circHIPK3 and ciRS-7/CDR1as promote insulin secretion, as well as improve β cell function via sponging miR-124-3p, miR-338-3p (circHIPK3) along with miR-7 (ciRS-7/CDR1as) in diabetes mellitus (Xu et al., 2015; Stoll et al., 2018). CircARF3 (ADP-ribosylation factor 3), serves as an endogenous miR-103 sponge to abolish adipose inflammation via enhancing mitophagy (Zhang et al., 2019). Herein, circRNF111 contained a conserved miR-143-3p target site that was verified by bioinformatics approaches, RNA pull-down, circRIP, luciferase along with FISH assessment. Besides, we revealed that circRNF111 promotes the glucose and lipid metabolism of human adipogenic-derived stem cells by reverting miR-143-3p expression to inhibit the miR-143-3p-mediated suppression of the insulin pathway gene-IGF2R. Hence, we proposed a mechanism wherein circRNF111 acts as an miR-143-3p sponge to repress insulin resistance, enhance glucose uptake and repress ectopic lipid accumulation, therefore, delaying the progress of MetS.

In one of our previous studies, the remarkable hyperexpression of miR-143-3p were reported in serum samples of individuals with MetS in contrast with healthy individuals via microarray assessments and miR-143-3p contents were remarkably linked to the risk of insulin resistance after adjusting for confounding factors. Our data illustrated the repression of miR-143-3p protects against insulin resistance in MetS as well as obesity mice models by activating the insulin signaling pathway. Furthermore, miR-143-3p has been established to target IGF2R along with IGFBP5 thus possibly participates in insulin resistance reported in MetS (Xihua et al., 2019). In our present work, we found that circRNF111 was remarkably downregulated in the serum, as well as urine samples of individuals with MetS and circRNF111 expression was negatively linked to miR-143-3p levels. After adjusting for confounding factors, elevated circRNF111 was an independent protective factor of insulin resistance and lipid deposition. The powerful protective influence along with the mechanism of circRNF111 sponging miR-143-3p in MetS was also testified in HFD-fed obese mice. Nevertheless, our study could be improved by using circRNF111 knockout mice or transgenic mice rather than using the tail vein of sh-circRNF111 adenovirus to downregulate circRNF111 expression. Furthermore, to limit the potential off-target risk of anti-circRNF111, there is a need to develop a safe and reliable method to weaken the circRNF111 effect by precise targeted therapy.

Notably, circRNAs research had become a research hotspot in the scientific community and researchers had attempted to link circRNAs with the initiation and progression of various diseases. Therefore, targeting dysfunctional circRNA-miRNA-mRNA regulatory axis fulfill a critical therapeutic promise. Here, we have identified and replenished the circRNF111-miR-143-3p-IGF2R axis as a promising therapeutic target in the alleviation of insulin resistance and lipid deposition of MetS.

## Data Availability Statement

All datasets presented in this study are included in the article/Supplementary Material or can be accessed online at: https://github.com/linxihua2020/linxihua.

## Ethics Statement

The studies involving human participants were reviewed and approved by the Ethics Committee of Sir Run Run Shaw Hospital, Hangzhou, China. The patients/participants provided their written informed consent to participate in this study. The animal study was reviewed and approved by the Ethics Committee of Sir Run Run Shaw Hospital, Hangzhou, China.

## Author Contributions

HL, FW, JZ, FZ, and XL conceived of and designed the research. XL led the experiments. XL and YD led the data analysis. WL, YZ, WG, SS, and GW performed the experiments. HL, FW, GW, and FZ provided reagents. XL and DE wrote the manuscript with input from all authors. HL and FW supervised the whole project.

## Conflict of Interest

The authors declare that the research was conducted in the absence of any commercial or financial relationships that could be construed as a potential conflict of interest.

## Publisher’s Note

All claims expressed in this article are solely those of the authors and do not necessarily represent those of their affiliated organizations, or those of the publisher, the editors and the reviewers. Any product that may be evaluated in this article, or claim that may be made by its manufacturer, is not guaranteed or endorsed by the publisher.

## Footnotes

1
http://www.bioinformatics.babraham.ac.uk/projects/fastqc/

2
http://mirdb.org/

3
http://www.targetscan.org/

4
http://www.circbank.cn

5
https://bibiserv.cebitec.uni-bielefeld.de/rnahybrid/
