# Supplementary material for: CircRNF111 Protects Against Insulin Resistance and Lipid Deposition via Regulating miR-143-3p/IGF2R Axis in Metabolic Syndrome
Source: Front Cell Dev Biol. 2021 Aug 17;9:663148. doi: 10.3389/fcell.2021.663148 (PMC8415985; doi:10.3389/fcell.2021.663148)
Supplement: Supplementary file 1 [file Data_Sheet_1.ZIP › Supplemental File Sets/STARíεMETHODS.docx]

**STAR☆METHODS**

**KEY RESOURCES TABLE**

| REAGENT or RESOURCE | SOURCE | IDENTIFIER |
| --- | --- | --- |
| Antibodies | | |
| Anti-Akt (40D4 ) | Cell Signaling Technology | Cat# 2920 |
| Anti- phosphorylated Akt (D9E) | Cell Signaling Technology | Cat# 4060 |
| Anti-IGF2R (D3V8C) | Cell Signaling Technology | Cat# 14364 |
| Anti-IGFBP5 | Proteintech Group, Inc | Cat# 55205-1-AP |
| Anti-β-actin (13E5) | Cell Signaling Technology | Cat# 4970 |
| Anti-Ago2 | Millipore | Cat# 03-110 |
| Chemicals, Peptides, and Recombinant Proteins | | |
| Insulin for ITT | Eli Lilly Company | Cat# J20080093 |
| Insulin for cell differentiation | TOCRIS | Cat# 3435 |
| 3-isobutyl-1-methylxanthine | Sigma | Cat# I-7018 |
| Dexamethasone | Sigma | Cat# D-4902 |
| 2-[N-(7-nitrobenz-2-oxa-1,3-diaz-ol-4-yl) amino]-2-deoxy-d-glucose | Invitrogen | Cat# N13195 |
| Oil Red O | Sigma | Cat# O1391 |
| TRIzol Reagent | Invitrogen | Cat# 15596018 |
| TRIzol LS Reagent | Invitrogen | Cat# 10296028 |
| RNase R | Epicentre | Cat# RNR07250 |
| DAPI | Thermo Fisher Scientific | Cat# 62248 |
| Oligonucleotides | | |
| Agomir-NC | RiboBio | Cat# miR04101 |
| Agomir-143-3p | RiboBio | Cat# miR40000210 |
| Antagomir-NC | RiboBio | Cat# miR03101 |
| Antagomir-143-3p | RiboBio | Cat# miR30000256 |
| CircRNF111 siRNAs | RiboBio | Cat# siB190717031545 |
| NC siRNAs | RiboBio | Cat# siN0000001-1-5 |
| Cy3-labeled circRNF111 probes | RiboBio | NA |
| 488-labeled locked nucleic acid miR-143-3p probes | RiboBio | NA |
| Biotin-labeled circRNF111 probe | Sangon Biotech | NA |
| Experimental Models | | |
| Male C57BL/6 mice | Slack Experimental Animal Center of the Chinese Academy of Sciences | NA |
| High-fat diet | Research Diets | Cat# M10160 |
| Critical Commercial Assays | | |
| One Touch Ultra glucose strips | LifeScan | Cat# LS021-098 |
| Reverse Transcription Reagents Kit | TaKaRa | Cat# RR037A |
| SYBR Green | TaKaRa | Cat# DRR041A |
| RNeasy MinElute Cleanup Kit | Qiagen | Cat# 74204 |
| MiRNeasy Mini kit | Qiagen | Cat# 217004 |
| Urine RNA Purification Kit | Abnova | Cat# KA4427 |
| A MiDETECT TrackTM miRNA qRT-PCR Starter Kit | RiboBio | Cat# R10048.3 |
| Pierce Magnetic RNA-Protein Pull-Down Kit | Thermo Scientific | Cat# 20164 |
| Silver Staining Kit | Beyotime Biotechnology | Cat# P0017S |
| Streptavidin Dynabeads | Invitrogen | Cat# M-280 |
| Magna RIP RNA-Binding Protein Immunoprecipitation Kit | Millipore | Cat#17-700 |
| Fluorescent in Situ Hybridization Kit | RiboBio | Cat# C10910 |
| LipoFiterTM Transfection Reagent | Hanbio Co. Ltd | Cat# HB-LF-1000 |
| Lipofectamine3000 Transfection Reagent | ThermoFisher | Cat# L3000015 |
| Lipofectamine RNAiMAX Transfection Reagent | ThermoFisher | Cat# 13778100 |
| Dual-Luciferase Reporter Assay System | Promega | Cat# E1910 |
| Chemiluminescent ECL assay kit | Millipore | Cat# WBKLS0500 |
| Immun-Blot PVDF membranes | Millipore | Cat# IPVH00010 |
| Recombinant DNA and Adenovirus | | |
| sh-circRNF111 adenovirus | Hanbio Co. Ltd | NA |
| miR-143-3p sponge adenovirus | Hanbio Co. Ltd | NA |
| pDC311-U6-MCMV-EGFP vector | Hanbio Co. Ltd | NA |
| IGF2R/GFP-pcDNA3.1 plasmid | GeneChem Co. Ltd | NA |
| pGL4-basic vector | Promega | Cat# E665A |
| pRL-TK vector | Promega | Cat# E2241 |
| Software and Algorithms | | |
| FACScalibu | Becton Dickinson, Franklin Lakes, USA | NA |
| Microscope | TE2000-E; Nikon, Japan | NA |
| 7500 real-time PCR detection system | Applied Biosystems | NA |
| Mass Spectrometry | BIOTREE | NA |
| NanoDrop | NanoDrop | Cat# ND-1000 |
| Serum/Plasma Focus microRNA PCR Panel | Exiqon | (Xihua et al., 2019) |
| TaqMan® Array Human MicroRNA Cards B v3 | Thermo Fisher Scientific | Cat# 4444910 |
| Agilent 2100 Bioanalyzer | Agilent Technologies | NA |
| Ribo-Zero™ rRNA Removal Kit | Illumina | NA |
| Illumina HiSeq 4000 | LC Bio | NA |
| MiRanda | (Agarwal et al., 2015) | [http://www.microrna.org](http://www.microrna.org/) |
| TargetScan | (Das, 2012) | [http://www.targetscan.org/](http://www.targetscan.org/)) |
| CircBank | (Liu et al., 2019) | [http://www.circbank.cn](http://www.circbank.cn/) |
| RNAhybrid | (Krüger and Rehmsmeier, 2006) | <https://bibiserv.cebitec.uni-bielefeld.de/rnahybrid/> |
| Cutadapt | (Martin, 2011; Chen et al., 2014) |  |
| FastQC | (Brown et al., 2017) | <http://www.bioinformatics.babraham.ac.uk/projects/fastqc/> |
| TopHat2 | (Kim et al., 2013) | NA |
| STAR | (Dobin et al., 2013) | NA |
| CIRI2 | (Gao et al., 2015) | NA |
| CIRCexplore | (Dong et al., 2019) | NA |
| SRPBM | (Zheng et al., 2016) | NA |
| Other | | |
| DNA sequencing | Tsingke | NA |
| RNA libraries sequencing | Origingene Bio-pharm Biotechnology | NA |
| SPSS 22.0 | IBM | NA |
| GraphPad Prism 5 | GraphPad | NA |

**LEAD CONTACT AND MATERIALS AVAILABILITY**

Further information and requests for resources and reagents should be directed to and will be fulfilled by the Lead Contact, Xihua Lin ([linxihua@zju.edu.cn](mailto:linxihua@zju.edu.cn)). This study did not generate new unique reagents.

**EXPERIMENTAL MODEL AND SUBJECT DETAILS**

**Cross-sectional study subjects**

Subjects were selected from a population based cross-sectional survey, which was conducted from March to May 2010 in the Caihe community of Hangzhou, Zhejiang province, China, as previous reported(Xueyao et al., 2014). Briefly, a total of 624 eligible Han Chinese participants (age, 40‑65 years) were recruited. Participants were 56.40±6.52 years and 51.25% were male. All patients completed a population‑based cross‑sectional survey and were assigned a number. Random numbers were generated electronically and 40 patients with MetS (56.82±6.58 years; 57.5% male) and 40 control subjects (56.33±6.12 years; 45.0% male) were selected. The serum and urine samples were collected. The study protocol was approved by the Ethics Committee of Sir Run Run Shaw Hospital and all patients provided informed consents. Participants were interviewed face‑to‑face by trained medical staff, completing a questionnaire regarding demographic data, life style, present and past illness, medical therapy and other health‑associated information. All baseline anthropometric and metabolic measurements were referred to recorded using standardized methods as previously reported(Xueyao et al., 2014; Xihua et al., 2019).

**Diagnosis of MetS**

MetS was diagnosed according to criteria established by the Joint Committee for Developing Chinese Guidelines on Prevention and Treatment of Dyslipidemia in Adults (JCDCG)(Joint Committee for Developing Chinese guidelines on Prevention and Treatment of Dyslipidemia in Adults, 2007). Individuals with≥3 of the following abnor­malities were considered to have MetS: Central obesity [waist circumference (WC), >90 cm for men and >85 cm for women]; hypertriglyceridemia (≥1.70 mmol/l); high density lipopro­tein‑cholesterol (HDL‑c; <1.04 mmol/l); blood pressure (BP; ≥130/85 mmHg or ongoing treatment for hypertension); and hyperglycemia [fasting plasma glucose (FPG)≥6.1 mmol/l or 2 h postprandial glucose≥7.8 mmol/l]. The Homeostasis Model of Assessment-Insulin Resistance (HOMA-IR) value was calculated using a previously established formula: HOMA-IR=[fasting insulin(mIU/L)×fasting plasma glucose (mmol/L)/22.5](Emoto et al., 1999).Patients suffering from abnormal renal function or renal dysfunction at the time of recruitment were excluded. Patients fulfilling none of the above criteria were selected as healthy controls.

**Mice**

All animal care procedures and methods were performed in accordance with the guidelines of the Animal Care Committee of Zhejiang University. All animals were maintained in a specific pathogen-free facility. Male C57BL/6 mice (aged 8 weeks) were purchased from Slack Experimental Animal Center of the Chinese Academy of Sciences (Shanghai, China). A cohort of 25 mice were housed and maintained on a 12h light-dark cycle. After 1 week of habituation and maintenance on a regular chow diet, mice were weighed and divided into two groups: 5 into a normal-chow diet group (NCD group; carbohydrate, 63.92%; protein, 26.18%; fat, 9.9%) and 20 into a high-fat diet group (HFD group, 35% carbohydrate, 20% protein and 45% fat; Research Diets, NJ) for 12 weeks. Mice were provided free access to water and either an NCD or HFD under controlled conditions of light and temperature. After 8 weeks of maintenance on either an NCD or HFD, an intraperitoneal glucose tolerance test (GTT) was performed after an overnight fast of the animals. After 10 weeks, an intraperitoneal insulin tolerance test (ITT) was performed after a 4h fast. After 12 weeks of, mice were sacrificed by cervical dislocation after exsanguination and their body weights were measured. For the other 15 HFD-induced obese mice, sh-control adenovirus (n = 5), sh-circRNF111 adenovirus (n = 5), sh-circRNF111 adenovirus combine with miR-143-3p sponge adenovirus (n = 5) were administered twice weekly via tail vein injection for another two weeks (1*10E11 vg/ml), the GTT and ITT were performed again as mentioned above. After 16 weeks of, plasma and tissue samples were frozen promptly in liquid nitrogen and stored at -80°C for histological and biochemical analysis. Liver, skeletal muscle, inguinal white adipose tissues (iWAT) and epididymal white adipose tissues (eWAT) were fixed in 4% formaldehyde, embedded in an OCT compound, and sectioned (thickness, 4μm) according to a standard protocol. The sections were stained with hematoxylin and eosin (H&E) and examined under light microscopy.

**METHOD DETAILS**

**GTT and ITT**

For the GTT, mice were fasted overnight and then injected intraperitoneally with 1.5g/kg glucose. Blood glucose levels were measured from tail blood using One Touch Ultra glucose strips (LifeScan, PA, USA) at predetermined time points (0min, 15min,30min,60min and 120min). For the ITT, mice were fasted for 4h and injected intraperitoneally with 0.5U/kg insulin (Eli Lilly Company, USA), and blood glucose levels were measured at predetermined time points using the above methodology.

**Cell culture and adipogenic differentiation of hADSCs preadipocytes**

Human adipose-derived mesenchymal stem cells (hADSCs) and 293HEK cells were purchased from the American Type Culture Collection (ATCC). They were cultured in Dulbecco's modified Eagle's medium (DMEM) containing 10% fetal bovine serum (FBS, Bio-rad) and 100IU/mL penicillin/streptomycin at 37°C, 5% CO_2_ and 95% humidity. Two days post-confluence, hADSCs (designated as Day 0) were induced to differentiate into adipocytes via addition of differentiation mixture with DMEM containing 10% FBS, 10μg/mL insulin (TOCRIS, USA), 0.5mM 3-isobutyl-1-methylxanthine (IBMX, Sigma, USA) and 1μM dexamethasone (Sigma, USA). Two days later, culture medium was changed to DMEM supplemented with 10% FBS and 10μg/mL insulin for two days. Medium was subsequently replaced every other day with DMEM containing 10% FBS for different periods until day ten. 293HEK cells were used to perform Dual-luciferase reporter assays.

**Glucose Uptake Measurement by Flow Cytometry**

Glucose uptake activity was measured using a fluorescent D-glucose analogue 2-[N-(7-nitrobenz-2-oxa-1,3-diaz-ol-4-yl) amino]-2-deoxy-d-glucose (2-NBDG, Invitrogen) in differentiated hADSCs. Briefly, differentiated hADSCs in 12-well plates were treated with agomirs and antagomirs for 48h. Cells were then washed with Dulbecco’s phosphate buffered saline (DPBS) and incubated with 100nM insulin in glucose-free DMEM for 10 min. 60μM 2-NBDG was added to the medium for another 1 h. The medium was then washed twice with cold DPBS to remove free 2-NBDG. Cells in each well were suspended with DPBS after trypsinisation; fluorescence activity was subsequently determined by flow cytometry according to manufacturer instructions. Fluorescence activity was monitored at excitation and emission wavelengths of 485nm and 535nm, respectively. The fluorescence intensity of 2-NBDG was recorded in the FL1 channel using a FACScalibur (Becton Dickinson, Franklin Lakes, U.S.A.) flow cytometer. Data from 1000 unique cell events were collected. To rule out false-positives, treatment in the absence of 2-NBDG was measured and considered as background readings. Relative fluorescence intensities minus background levels were used for data analysis.

**Oil red O staining**

Ten days after induction of hADSCs differentiation, cells were washed twice in D-Hank’s solution, fixed in 4% formaldehyde for 30 min, and washed thrice with water. Then, cells were stained with Oil Red O (Sigma, USA) for 15 min. Following three washes in water, lipid droplets were observed and photographed under a microscope (TE2000-E; Nikon, Japan).

**RNA extraction, RNase R treatment and circRNA/mRNA quantitative real-time PCR**

Total RNA was isolated from treated cells with the Trizol reagent (Invitrogen) and reverse-transcribed using a reverse transcription reagents kit (TaKaRa) to generate a cDNA template. Real-time quantitative PCR was performed using a PCR kit containing SYBR Green (TaKaRa) and a 7500 real-time PCR detection system (Applied Biosystems). For circRNA, total RNAs were incubated with or without 3 U/μg of RNase R (Epicentre, San Diego, CA, USA) at 37℃ for 20 minutes, and the resulting RNA was subsequently purified using the RNeasy MinElute Cleanup Kit (Qiagen). The specific divergent primers for the back-splice junction of circRNF111 were used to amplify circRNA. The amplification products were detected by agarose gel electrophoresis and sequencing. All reactions were run in triplicate and relative circRNF111 or mRNA expression was normalized to that of GAPDH as an internal control for quantification of individual mRNA species and calculated using the formula 2^−ΔΔCt^. Primer sets are listed in Supplementary Table10.

**Serum and urine miRNA detection**

Total RNA was extracted from serum using an miRNeasy kit (Qiagen); manufacturer instructions were followed. For each reaction, 2-4 µl of total RNA isolated from 250µl-1ml of serum samples was used. Total RNA was extracted from urine using Trizol LS reagent (Invitrogen) and purified using a Urine RNA Purification Kit (Abnova); manufacturer instructions were adhered to. For each reaction, 2-4 µl of total RNA isolated from 5-10ml of urine samples was used. A MiDETECT Track^TM^ miRNA qRT-PCR Start Kit (RiboBio, Guangzhou, China) was used for validating expression of microRNAs by poly(A) tailing-based RT-PCR, adhering to manufacturer directions. According to microarray results, U6 was selected as stable endogenous control miRNAs. miRNA qPCR Primer sets were purchased from Guangzhou RiboBio Co. (Guangzhou, China).

**Pull-down assay with biotinylated circRNF111 probe**

For RNA-Protein pull-down assay, the Pierce Magnetic RNA-Protein Pull-Down Kit (Thermo Scientific, 20164) was used. Biotin-labeled circRNF111 probe was synthesized by Sangon Biotech. 100 pmol of biotinylated labeled RNA complementary to the junction sequence of circRNA111 was bound to 50ul Streptavidin Magnetic Beads lysates. Negative RNA control was used as a contrast. The hADSCs lysate was then incubated with RNA-bound beads in 1× binding buffer and 50% glycerol for 1h at 4°C with rotation. Next, complexes were isolated after three washes with ice-cold 1× washing buffer. RNA-binding-protein was detected in the pulldown by western blot analysis, silver staining (Beyotime Biotechnology, China) and Mass Spectrometry (BIOTREE, China).

**CircRNAs in vivo precipitation (circRIP)**

The circRIP assay was performed as described in the manuscript by Han D, et al(Han et al., 2017). A total of 1×10^7^ hADSCs were fixed by 1% formaldehyde for 10 minutes, lysed, and sonicated. The probes were denatured at 65 °C for 10 min and hybridized at room temperature for 2 h before adding 200 µl streptavidin-coated magnetic beads. After centrifugation, 50µl of the supernatant was retained as input and the remaining part was incubated with circRNF111-specific probes streptavidin dynabeads (M-280; Invitrogen) mixture overnight at 30 °C. On the next day, an M-280 dynabeads-probes-circRNAs mixture was washed and incubated with 200µl of lysis buffer and proteinase K to reverse the formaldehyde cross-linking. Finally, the mixture was added with Trizol for RNA extraction and qRT-PCR were used to analyze binding strength after reverse transcribing the miRNAs.The probes are shown in Supplementary Table S10.

**RNA immunoprecipitation**

The Ago-RIP assay was conducted in hADSCs cells using the Magna RIP RNA-Binding Protein Immunoprecipitation Kit (Millipore, Bedford, MA, USA) according to the manufacturer’s instructions. Approximately 1×10^7^ differentiated hADSCs were sedimented and resuspended with an equal pellet volume of RIP Lysis Buffer plus a protease inhibitor cocktail and RNase inhibitors. Cell lysates (200 µl) were incubated with 10 µg of antibody against Ago2 (Millipore, Billerica, MA, USA) or control rabbit IgG-coated beads and mixed by rotation at 4 °C overnight. After treating with proteinase K buffer, the immunoprecipitated RNAs were extracted by RNeasy MinElute Cleanup Kit (Qiagen, Düesseldorf, Germany) and reverse transcribed using Prime- Script RT Master Mix (TaKaRa, Tokyo, Japan). The abundance of circRNF111 or GAPDH was detected by RT–qPCR assay.

**RNA fluorescent in situ hybridization (FISH)**

The FISH assay was performed in HCs or rabbit tissues. Cy3-labeled circRNF111 probes and 488-labeled locked nucleic acid miR-143-3p probes were designed and synthesized by RiboBio (Guangzhou, China). The signals of the probes were detected by Fluorescent in Situ Hybridization Kit (RiboBio, Guangzhou, China) according to the manufacturer’s instructions. The images were acquired on Nikon A1Si Laser Scanning Confocal Microscope (Nikon Instruments Inc, Japan). For in vivo FISH, tissue sections were deparaffinised, rehydrated, and permeabilized by 0.8% pepsin treatment at 37°C for 30 min before hybridization. The primer and prober sequences are listed in Supplementary Table S10.

**Adenovirus knockdown**

For recombinant adenovirus sh-circRNF111 construction, oligonucleotides with the circRNF111 targeting sequences were used for the cloning of small hairpin RNA (shRNA)-encoding sequences into pDC311-U6-MCMV-EGFP vector (purchased from Hanbio Co. Ltd, Shanghai, China). For miR-143-3p sponge adenovirus construction, self-complementary DNA oligonucleotides encompassing the sequence of the miRNAs miR-143-3p were chemically synthesized, including overhang sequences from a 5’ BamH1- and a 3’ EcoRI-restriction site. Annealed oligonucleotides were directionally cloned into the BamH1/EcoRI-digested pDC311-U6-MCMV-EGFP vector. The pDC311-sh-circRNF111 or pDC311-miR-143-3p-sponge and pBHGlox_E1,3Cre(Ng et al., 1999) were co-transfected into HEK293 cells by using LipoFiter^TM^ transfection reagent (Hanbio, Shanghai, China) to generate the recombinant adenoviruses (sh-circRNF111). Adenoviruses harboring green fluorescent protein (sh-control) were used as a control. Then, sh-circRNF111 or pDC311-miR-143-3p-sponge and sh-control were propagated in HEK293 cells. The Propagated recombinant adenoviruses in the HEK293 cells were purified and the titer of virus was measured by plaque assays. The stock solutions of sh-circRNF111 or pDC311- miR-143-3p-sponge and sh-control were made with 1×10^11^ plaque formation unit (PFU)/ml.

**Agomirs/antagomirs treatment, RNA interference and IGF2R overexpression**

The cholesterol-conjugated 2′-*O*-methyl-modified mimic (agomir-143-3p), agomir-NC, antagomir-143-3p and antagomir-NC were synthesized by RiboBio (RiboBio, Guangzhou, China) to inhibit or induce miR-143-3p expression, respectively(Krützfeldt et al., 2005). When differentiated hADSCs were approximately 30–40% confluent, either agomir-143-3p (100nM) or antagomir-143-3p (200nM), as well as matched controls, were treated. Cells were subsequently harvested after 48h or 72h and re-treated every two days throughout the process of induction of differentiation. CircRNA expression was suppressed by siRNA-mediated knockdown. Four different siRNAs were designed and tested for circRNF111 (Ribobio), siRNA sequences are shown in Supplementary Table S10. The IGF2R-pcDNA3.1 overexpression plasmid(oe-IGF2R) and GFP-pcDNA3.1(oe-Vector) control vectors were conducted by Shanghai GeneChem Co., Ltd. (Shanghai, China). Cells transfection with plasmids was achieved using Lipofectamine 3000 transfection reagent (ThermoFisher). Lipofectamine RNAiMAX transfection reagent (ThermoFisher) was used for the siRNAs, agomir-143-3p and antagomir-143-3p.

**Dual-luciferase reporter assays**

*Homo sapiens* IGF2R (Gene ID: 3482) 3'untranslated regions (3'UTRs) containing predicted miR-143-3p binding sites and its mutated forms were amplified via PCR and cloned into the XhoI/HindIII site of a pGL4-basic vector (Promega) to generate the IGF2R-3'UTR-WT and IGF2R-3'UTR-Mut vectors. The luciferase coding sequence was fused to the 3′UTRs of IGF2R. All PCR products were verified by DNA sequencing. 293HEK cells (5×10^4^) were seeded in 24-well plates 24h before transfection. The following day, 200ng of construct plasmids (IGF2R-3'UTR-WT and IGF2R-3'UTR-Mut reporter plasmid) along with 100ng of the *Renilla* luciferase plasmid (pRL-TK, considered as an internal control) constitutively expressing *Renilla* luciferase were co-transfected using Lipofectamine 3000 (Invitrogen). 100nM of agomir-143-3p or 20nM si-circRNF111 was administered after 6-8h of culture medium replacement. Cells were harvested 48h post-transfection and assayed for luciferase activity using the Dual-Luciferase Reporter Assay System (Promega). For each experimental trial, cells were transfected with the same plasmids in quadruplicate, and Luciferase activities were normalized to the co-transfected pRL-TK plasmid (mean ± S.D.). Primer sets are listed in Supplementary Table10.

**Serum miRNA microarray**

Genome‑wide circulating miRNA profiles were detected using a microarray (Serum/Plasma Focus microRNA PCR Panel, V1.M, Exiqon) in serum samples from 4 patients with MetS and 4 controls. Patients for the microarray analysis were selected at random out of the 40 patients previously identified per group. Comprehensive coverage was ensured using the TaqMan® Array Human MicroRNA Cards B v3 (cat. no. 4444910; Thermo Fisher Scientific, Inc., Waltham, MA, USA), resulting in a total of 377 unique assays specific for human miRNAs. A total of 8 RNA samples were analyzed by Kangchen BioTech Co., Ltd. (Shanghai, China) according to manufacturers' instructions.

**Circular RNA library construction and sequencing**

Total RNA from serum samples of 4 clinical MetS and 4 control tissues was isolated and purified using Trizol reagent (Invitrogen, Carlsbad, CA, USA) following the manufacturer's protocol. The RNA concentration and purity of each sample was quantified using NanoDrop ND-1000 (NanoDrop, Wilmington, DE, USA). The RNA integrity was assessed using an Agilent 2100 Bioanalyzer (Agilent Technologies) with a threshold of RIN>7.0. Approximately 5μg of total RNA was subjected to ribosomal RNA depletion using a Ribo-Zero™ rRNA Removal Kit (Illumina Inc.) according to the manufacturer’s instructions. The remaining RNA was treated with RNase R (Epicentre Inc., Madison, WI, USA) to remove linear RNAs and enrich circRNAs. Following the ribosomal and linear RNA removal steps, the enriched circRNAs were fragmented using divalent cations under high temperature. The cleaved RNA fragments were reverse transcribed, and the resulting cDNA was used to synthesize U-labeled second-stranded DNA with E. coli DNA polymerase I, RNase H and dUTP. An A-base was then added to the blunt ends of each strand to prepare them for ligation to the indexed adapters. Each adapter contained a T-base overhang to allow ligation to the A-tailed fragmented DNA. Single- or dual-index adapters were ligated to the fragments, and size selection was performed using AMPure XP beads. Following heat-labile UDG enzyme treatment of the U-labeled second-stranded DNAs. The average insert size for the final cDNA library was 300 bp (±50 bp). Finally, we performed paired-end sequencing on an Illumina HiSeq 4000 (LC Bio, China) following the vendor's recommended protocol. RNA libraries were sequenced by Shanghai Origingene Bio-pharm Biotechnology Co.,Ltd.

**Circular RNA data analysis**

First, Cutadapt(Martin, 2011) was used to remove the reads that contained adaptor contamination, low quality bases and undetermined bases. Sequence quality was then verified using FastQC. TopHat2(Kim et al., 2013) and STAR(Dobin et al., 2013) were used to map reads to hg19 (the human genome). CIRI2(Gao et al., 2015) and CIRCexplore(Dong et al., 2019) were used for identification of back splicing reads, and subsequent de novo assembly of the mapped reads to circRNAs. Only circRNAs identified by both algorithms were treated as valid candidates. All samples generated unique circular RNAs. To evaluate expression levels of circRNAs, we normalized the back-spliced reads (evidence for circRNA) by read length and number of mapped reads (spliced reads per billion mapping, denoted as SRPBM(Zheng et al., 2016)), which permits quantitative comparison of back splicing from different RNA-seq data. The differentially expressed circRNAs were selected using R package-edgeR with the significance threshold set as |log2 (fold change)| >1 and p<0.05.

**QUANTIFICATION AND STATISTICAL ANALYSIS**

For the cross-sectional study, all continuous variables were tested for a normal distribution. Normally distributed variables were expressed as mean±S.D.. Variables with a skewed distribution were presented with a median (interquartile range, 25-75%) and logarithmically transformed to achieve a normal distribution before analysis. Categorical variables were presented as frequencies and percentages. Differences of baseline characteristics between participants with and without Mets were analyzed via t-test for continuous variables and via Chi-square test for categorical variables. Spearman correlations between serum or urine circRNF111 and metabolic parameters before and after adjustment for age, gender, smoking and drinking status were obtained via Spearman correlation analysis in the cross-sectional study. Incidence of MetS at different levels of serum or urine circRNF111 was analyzed by the Chi-square. Logistic regression and multiple stepwise regression analyses considering a serum or urine circRNF111 levels as a dependent variable after adjustment for gender, age, smoking and drinking were conducted in the prospective study to evaluate what influences variables exert on MetS risk, HOMA-IR and other metabolic subgroups (central obesity, leveled BP, hyperglycemia, hypertriglyceridemia, and low HDL-c) associated with serum and urine circRNF111 levels. SPSS 22.0 (IBM, USA) was used for statistical analyses. All statistical analyses were performed using the software GraphPad Prism 5 for Microsoft Windows. For animal or cell experiments, independent two-sided Student's t-test and one-way ANOVA were used to analyze two or multiple groups, respectively. Each experiment was performed in triplicate. Data are presented as the mean±SEM (*in vivo* studies) or mean±S.D. (*in vitro* studies). Results are representative of three independent experiments. A significant difference was defined as p<0.05.

**DATA AND CODE AVAILABILITY**

The circRNA deep sequencing data and other supplementary data reported in this paper can be accessed here: <https://github.com/linxihua2020/linxihua>.

**REFERENCES**

Agarwal, V., Bell, G. W., Nam, J. W., and Bartel, D. P. (2015). Predicting effective microRNA target sites in mammalian mRNAs. *Elife* 4 . doi: 10.7554/eLife.05005

Brown, J., Pirrung, M., and McCue, L. A. (2017). FQC Dashboard: integrates FastQC results into a web-based, interactive, and extensible FASTQ quality control tool. *Bioinformatics* . doi: 10.1093/bioinformatics/btx373

Chen, C., Khaleel, S. S., Huang, H., and Wu, C. H. (2014). Software for pre-processing Illumina next-generation sequencing short read sequences. *Source Code Biol Med* 9, 8. doi: 10.1186/1751-0473-9-8

Joint Committee for Developing Chinese guidelines on Prevention and Treatment of Dyslipidemia in Adults (2007). [Chinese guidelines on prevention and treatment of dyslipidemia in adults]. *Zhonghua Xin Xue Guan Bing Za Zhi* 35, 390-419.

Das, N. (2012). MicroRNA Targets - How to predict. *Bioinformation* 8, 841-845. doi: 10.6026/97320630008841

Dobin, A., Davis, C. A., Schlesinger, F., Drenkow, J., Zaleski, C., Jha, S., et al. (2013). STAR: ultrafast universal RNA-seq aligner. *Bioinformatics* 29, 15-21. doi: 10.1093/bioinformatics/bts635

Dong, R., Ma, X. K., Chen, L. L., and Yang, L. (2019). Genome-Wide Annotation of circRNAs and Their Alternative Back-Splicing/Splicing with CIRCexplorer Pipeline. *Methods Mol. Biol.* 1870, 137-149. doi: 10.1007/978-1-4939-8808-2_10

Emoto, M., Nishizawa, Y., Maekawa, K., Hiura, Y., Kanda, H., Kawagishi, T., et al. (1999). Homeostasis model assessment as a clinical index of insulin resistance in type 2 diabetic patients treated with sulfonylureas. *Diabetes Care* 22, 818-822. doi: 10.2337/diacare.22.5.818

Gao, Y., Wang, J., and Zhao, F. (2015). CIRI: an efficient and unbiased algorithm for de novo circular RNA identification. *Genome Biol.* 16, 4. doi: 10.1186/s13059-014-0571-3

Han, D., Li, J., Wang, H., Su, X., Hou, J., Gu, Y., et al. (2017). Circular RNA circMTO1 acts as the sponge of microRNA-9 to suppress hepatocellular carcinoma progression. *Hepatology* 66, 1151-1164. doi: 10.1002/hep.29270

Kim, D., Pertea, G., Trapnell, C., Pimentel, H., Kelley, R., and Salzberg, S. L. (2013). TopHat2: accurate alignment of transcriptomes in the presence of insertions, deletions and gene fusions. *Genome Biol.* 14, R36. doi: 10.1186/gb-2013-14-4-r36

Krüger, J., and Rehmsmeier, M. (2006). RNAhybrid: microRNA target prediction easy, fast and flexible. *Nucleic Acids Res.* 34, W451-454. doi: 10.1093/nar/gkl243

Krützfeldt, J., Rajewsky, N., Braich, R., Rajeev, K. G., Tuschl, T., Manoharan, M., et al. (2005). Silencing of microRNAs in vivo with 'antagomirs'. *Nature* 438, 685-689. doi: 10.1038/nature04303

Liu, M., Wang, Q., Shen, J., Yang, B. B., and Ding, X. (2019). Circbank: a comprehensive database for circRNA with standard nomenclature. *RNA Biol* 16, 899-905. doi: 10.1080/15476286.2019.1600395

Martin, M. (2011). Cutadapt removes adapter sequences from high-throughput sequencing reads. 17 .

Ng, P., Parks, R. J., Cummings, D. T., Evelegh, C. M., Sankar, U., and Graham, F. L. (1999). A high-efficiency Cre/loxP-based system for construction of adenoviral vectors. *Hum. Gene Ther.* 10, 2667-2672. doi: 10.1089/10430349950016708

Xihua, L., Shengjie, T., Weiwei, G., Matro, E., Tingting, T., Lin, L., et al. (2019). Circulating miR-143-3p inhibition protects against insulin resistance in Metabolic Syndrome via targeting of the insulin-like growth factor 2 receptor. *Transl Res* 205, 33-43. doi: 10.1016/j.trsl.2018.09.006

Xueyao, Y., Saifei, Z., Dan, Y., Qianqian, P., Xuehong, D., Jiaqiang, Z., et al. (2014). Circulating fractalkine levels predict the development of the metabolic syndrome. *Int J Endocrinol* 2014, 715148. doi: 10.1155/2014/715148

Zheng, Q., Bao, C., Guo, W., Li, S., Chen, J., Chen, B., et al. (2016). Circular RNA profiling reveals an abundant circHIPK3 that regulates cell growth by sponging multiple miRNAs. *Nat Commun* 7, 11215. doi: 10.1038/ncomms11215
