# Supplementary material for: CircRNF111 Protects Against Insulin Resistance and Lipid Deposition via Regulating miR-143-3p/IGF2R Axis in Metabolic Syndrome
Source: Front Cell Dev Biol. 2021 Aug 17;9:663148. doi: 10.3389/fcell.2021.663148 (PMC8415985; doi:10.3389/fcell.2021.663148)
Supplement: Supplementary file 1 [file Data_Sheet_1.ZIP › Supplemental File Sets/Supplementary Figures and Tables.docx]

Supplementary Information for

**CircRNF111 Protects against Insulin Resistance and Lipid Deposition via Regulating miR-143-3p/IGF2R Axis in Metabolic Syndrome**

^*^Corresponding authors at: Department of Endocrinology, Sir Run Run Shaw Hospital, School of Medicine, Zhejiang University, Hangzhou, China.

3 East Qingchun Road, Hangzhou, Zhejiang, 310016, China.

E-mail addresses: [srrshnfm@zju.edu.cn](#mailto:srrshnfm@zju.edu.cn)(Hong Li) ；[zw5100637@zju.edu.cn](mailto:zw5100637@zju.edu.cn) (Fang Wu)

**This file includes:**

Figures S1 to S3

Tables S1 to S10


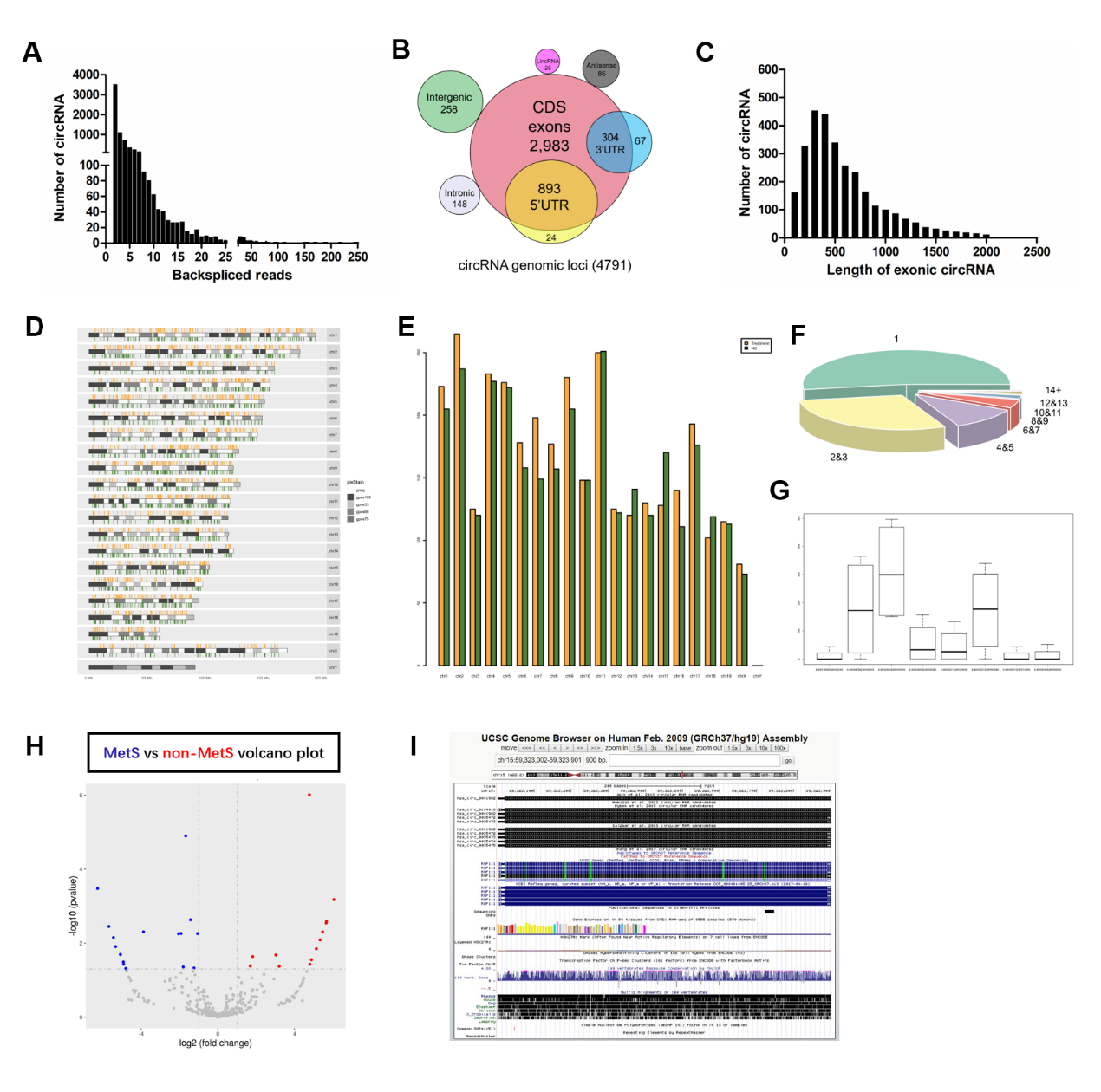


**Fig S1. Profiling of circRNAs in serum of MetS and control samples by circRNA deep sequencing.** **(A)** Each sample was sequenced on an Illumina HiSeq yielding at least 30 million reads. **(B)** A total of 4791 circRNAs were identified by both CIRI2 and CIRCexplorer. **(C)** The length of most exonic circRNAs was less than 1,500 nucleotides (nt), and the median length was ~500 nt. **(D，E)** Schematic illustration showing the chromosome distribution of the identified circRNAs. **(F)** Analysis of the number of circRNAs in their host genes indicated that one gene could produce multiple circRNAs. **(G)** The abundance of the circRNAs within one gene locus. **(H)** The volcano plot showed differential expression patterns of circRNAs of MetS serum and control samples. **(I)** Hsa_circ_0001982 is formed by the circularization of exon 2 of RNF111 gene, mapped to the human reference genome (GRCh37/hg19) assembly.


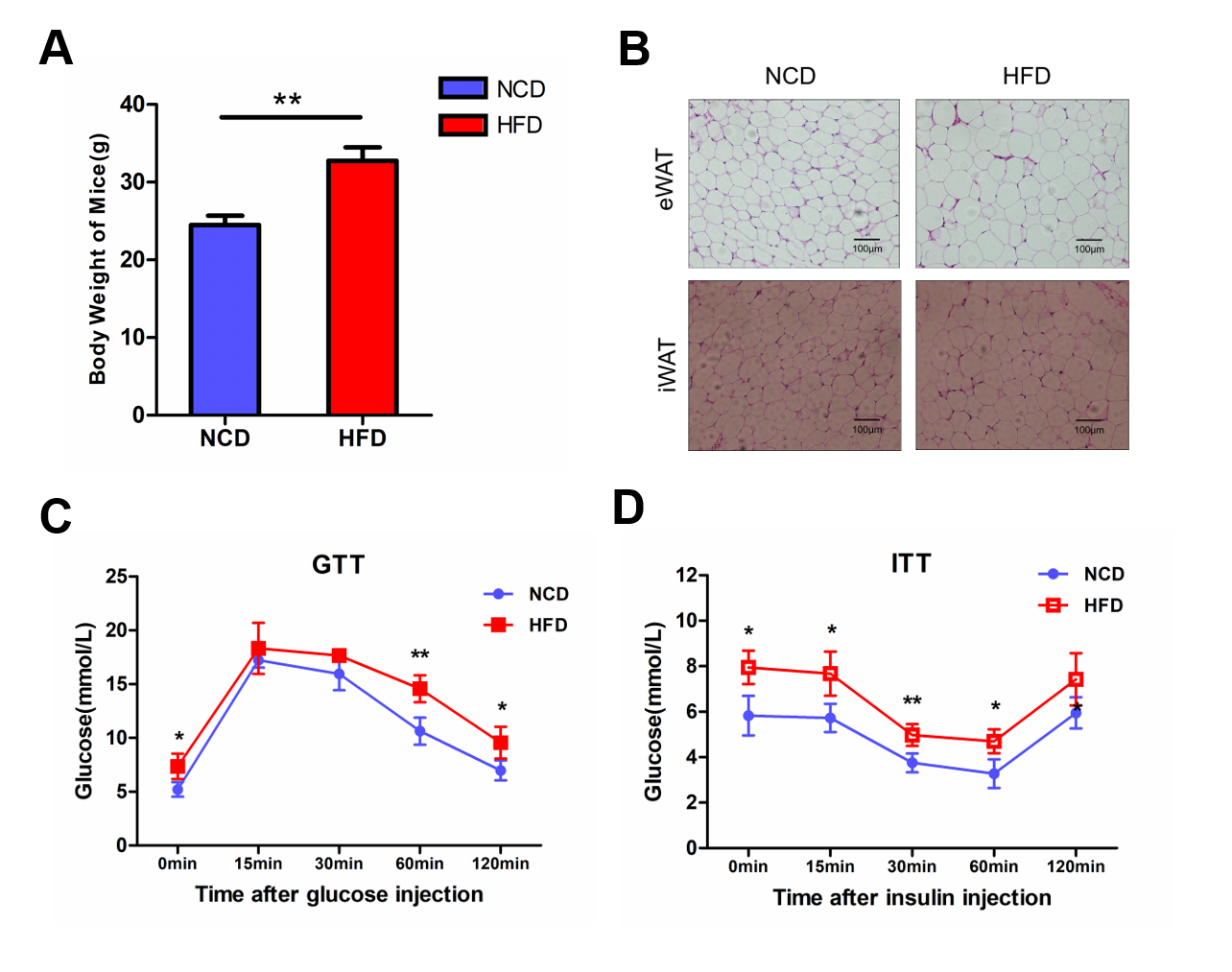


**Fig S2. High-fat diet-induced obesity and insulin resistance in C57BL/6 mice.** **(A)** Body weight of mice fed normal-chow-diet (NCD) and a high-fat diet (HFD) for 12 weeks (NCD, n = 5; HFD, n = 5). **(B)** Representative sections stained with Hematoxylin/Eosin of adipocytes in eWAT and iWAT (NCD, n = 5; HFD, n = 5). **(C)** Intraperitoneal glucose tolerance test. Mice were fasted overnight and then injected intraperitoneally with 1.5g/kg glucose, and blood glucose were measured at the indicated times (NCD, n = 5; HFD, n = 5). Data represent the means ± SEM, *p<0.05,**p<0.01. **(D)** Intraperitoneal insulin tolerance test. Mice were fasted 4h and then injected intraperitoneally with 0.5U/kg insulin, and blood glucose were measured at the indicated times (NCD, n = 5; HFD, n = 5). Data represent the means ± SEM, *p<0.05,**p<0.01.


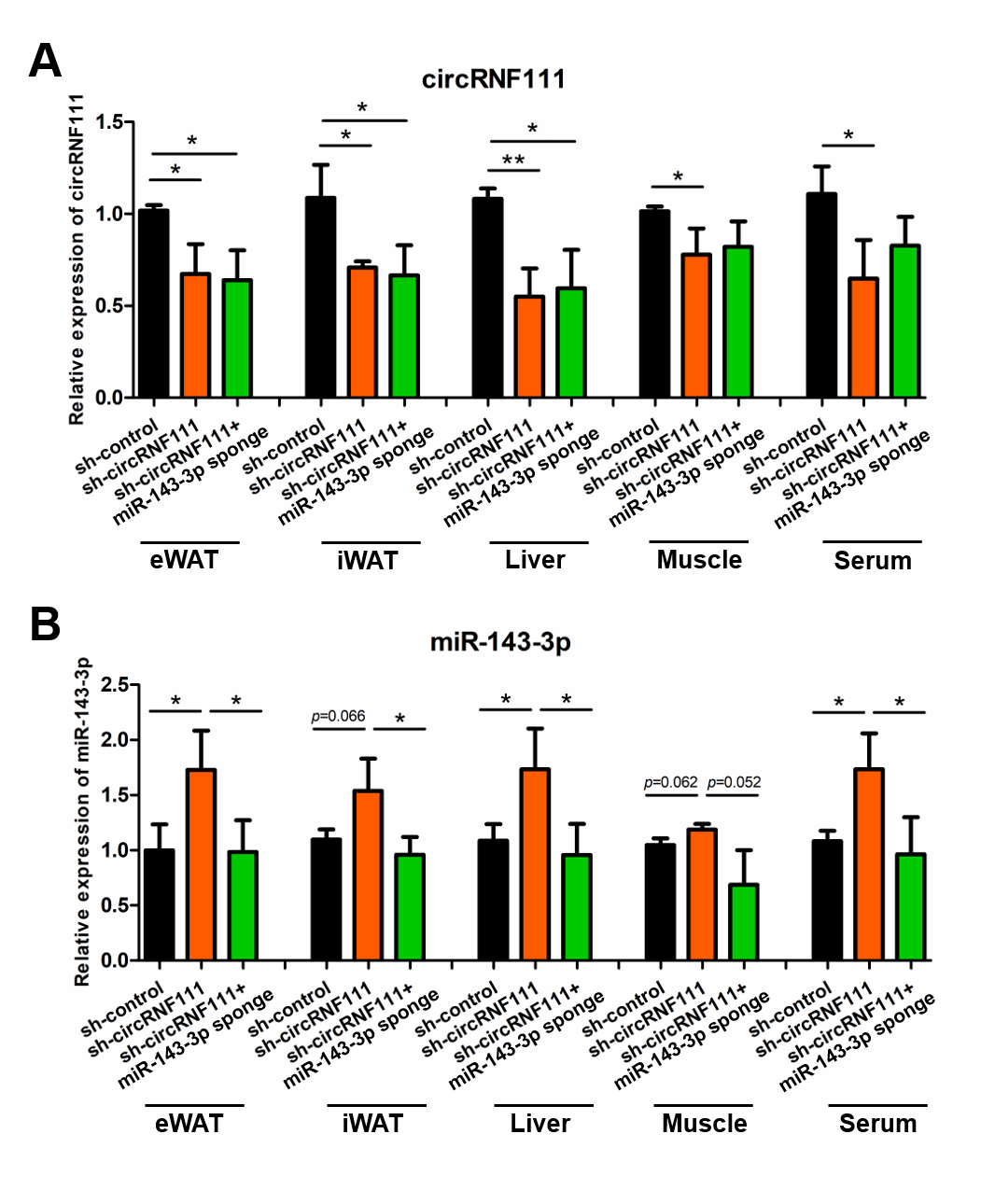


**Fig S3. CircRNF111 and miR-143-3p expression of eWAT, iWAT, liver, muscle and serum of adenovirus treated obesity mice.** **(A)** CircRNF111 expression of eWAT, iWAT, liver, muscle and serum of obese mice was injected with sh-control adenovirus (n = 5), sh-circRNF111 adenovirus (n = 5), sh-circRNF111 adenovirus combine with miR-143-3p sponge adenovirus (n = 5) were compared. *p<0.05,**p<0.01. **(B)** miR-143-3p expression of eWAT, iWAT, liver, muscle and serum of obese mice was injected with sh-control adenovirus (n = 5), sh-circRNF111 adenovirus (n = 5), sh-circRNF111 adenovirus combine with miR-143-3p sponge adenovirus (n = 5) were compared. *p<0.05,**p<0.01.

**Table S1. The circRNAs differentially expressing between MetS and non-MetS with fold change >2 and p<0.05**

| ID | log2Fold Change (MetS/Control) | p value | direction | significant |
| --- | --- | --- | --- | --- |
| 5:32955852-32968771 | -7.2095933 | 0.00013432 | down | yes |
| 17:57406833-57408495 | -5.7256104 | 0.0001382 | down | yes |
| 15:59323002-59323901 | -5.2760784 | 0.0001862 | down | yes |
| 6:134115219-134137417 | 4.06243391 | 0.00040484 | up | yes |
| 1:146682575-146701846 | -4.0531467 | 0.00111472 | down | yes |
| 6:134062063-134066465 | -3.7749837 | 0.00122753 | down | yes |
| 15:27553688-27571654 | -3.4219483 | 0.00296286 | down | yes |
| 17:50036940-50055627 | -3.85047 | 0.00300782 | down | yes |
| 11:117290397-117291036 | 3.52357915 | 0.00303382 | up | yes |
| 12:83574631-83575136 | 4.56983277 | 0.00524299 | up | yes |
| 10:98968693-98969124 | 2.04618052 | 0.0061645 | up | yes |
| 6:99435215-99456580 | -3.8014826 | 0.00705903 | down | yes |
| 14:118654149-118669107 | 2.86567306 | 0.00779452 | up | yes |
| 15:6836915-6842917 | -3.4977975 | 0.00811642 | down | yes |
| 6:99429209-99435345 | -3.3472394 | 0.00862442 | down | yes |
| 9:100834417-100855904 | -3.6844846 | 0.00873229 | down | yes |
| 4:88121269-88129052 | -3.3103606 | 0.00929394 | down | yes |
| 14:48168196-48179276 | -3.5440215 | 0.00954736 | down | yes |
| 1:191568290-191570704 | -3.2840934 | 0.01020717 | down | yes |
| 1:39973926-39980650 | 3.91773309 | 0.01077985 | up | yes |
| 17:57406833-57420524 | -2.8955174 | 0.01078439 | down | yes |
| 2:173688146-173695999 | -2.7507601 | 0.0134045 | down | yes |
| 12:69302323-69303962 | 2.2484411 | 0.01342013 | up | yes |
| 3:95991443-95992278 | -2.7923131 | 0.01370568 | down | yes |
| 8:56564557-56569410 | -2.7154978 | 0.0139525 | down | yes |
| 2:119076935-119086650 | -2.8798374 | 0.01416384 | down | yes |
| 9:64212583-64214609 | 3.54082201 | 0.01564169 | up | yes |
| 16:91782160-91793767 | -2.6103884 | 0.01569604 | down | yes |
| 17:74225431-74245087 | -4.0256687 | 0.01604729 | down | yes |
| 18:82955523-82957449 | 2.1840816 | 0.01628771 | up | yes |
| 17:39843774-39843992 | -2.3340353 | 0.01870983 | down | yes |
| 2:156177632-156178952 | -2.6963238 | 0.01990161 | down | yes |
| 1:165604609-165608604 | -2.298018 | 0.02001182 | down | yes |
| 4:63461281-63472868 | 2.33188823 | 0.0213266 | up | yes |
| 15:98951542-98953500 | -2.5316566 | 0.02133504 | down | yes |
| 9:119284545-119294523 | -2.8672356 | 0.02267481 | down | yes |
| 1:181956393-181961919 | -2.4410486 | 0.0227498 | down | yes |
| 17:79650301-79659473 | -2.7149079 | 0.02489881 | down | yes |
| 2:70768861-70786995 | -2.336148 | 0.02519941 | down | yes |
| 9:115266093-115280519 | 3.20707142 | 0.02528824 | up | yes |
| 3:98070859-98072922 | -3.0206565 | 0.02539876 | down | yes |
| 15:33249925-33302547 | -2.8597513 | 0.02762357 | down | yes |
| 7:118193449-118196035 | -2.4169485 | 0.02873991 | down | yes |
| 15:38493473-38498841 | 3.03998404 | 0.02895611 | up | yes |
| X:142832500-142834393 | 2.08199876 | 0.02926914 | up | yes |
| 18:5641381-5705243 | 2.07557749 | 0.03222577 | up | yes |
| 11:74656422-74657339 | 1.90831565 | 0.03320324 | up | yes |
| 9:82945757-82959851 | -2.4219052 | 0.03337239 | down | yes |
| 4:9529885-9532065 | -2.2898914 | 0.03571296 | down | yes |
| 15:12889618-12905701 | -2.1584349 | 0.03659215 | down | yes |
| 10:34583897-34591970 | 1.58844426 | 0.03682162 | up | yes |
| 19:4784723-4788042 | -2.5143186 | 0.0371715 | down | yes |
| 2:69914413-69922933 | -2.8364955 | 0.03729233 | down | yes |
| 18:61157342-61157761 | 2.12444385 | 0.03814177 | up | yes |
| X:151376747-151399803 | 3.08890248 | 0.03816561 | up | yes |
| 7:110052710-110054116 | 3.01222312 | 0.03835631 | up | yes |
| 18:36638204-36638708 | -2.181505 | 0.03909558 | down | yes |
| 11:97229829-97238258 | 2.89470144 | 0.03929864 | up | yes |
| 7:98243948-98266247 | 2.14474309 | 0.03967021 | up | yes |
| 1:33512015-33533363 | -2.7392927 | 0.03997965 | down | yes |
| 5:115884865-115886807 | -2.1872198 | 0.04022615 | down | yes |
| 4:152206763-152223162 | 2.0526393 | 0.04027099 | up | yes |
| 9:83952523-83991822 | -2.0173228 | 0.04080457 | down | yes |
| 2:128668924-128684663 | 1.70272444 | 0.041213 | up | yes |
| 6:119002576-119057515 | -2.1089966 | 0.04141039 | down | yes |
| 19:45630864-45640521 | -2.5100824 | 0.0422463 | down | yes |
| 19:40573770-40580076 | -2.0894124 | 0.04274839 | down | yes |
| 7:126551974-126552338 | -2.1791208 | 0.04383696 | down | yes |
| 14:57856609-57865641 | -2.8769173 | 0.04408098 | down | yes |
| 2:27922016-27929541 | -2.1044171 | 0.04411623 | down | yes |
| 5:36571295-36572037 | -2.3004161 | 0.04427124 | down | yes |
| 5:96128942-96134132 | 1.90239975 | 0.04490208 | up | yes |
| 5:114102382-114103826 | 2.00810182 | 0.0450386 | up | yes |
| 11:96770638-96773222 | -2.448017 | 0.04527876 | down | yes |
| 14:52181195-52181536 | -2.309055 | 0.04536368 | down | yes |
| 6:113408904-113409340 | 1.95850887 | 0.04637736 | up | yes |
| 10:116517316-116549175 | -2.4859603 | 0.04698215 | down | yes |
| 2:131511983-131516443 | 1.76079796 | 0.0470835 | up | yes |
| 9:21772744-21774752 | 1.91782781 | 0.0474196 | up | yes |
| 13:103884142-103889285 | 2.26421757 | 0.04906542 | up | yes |
| 13:103140162-103171556 | -2.104443 | 0.04925581 | down | yes |
| 4:59514273-59524476 | -2.234238 | 0.04941287 | down | yes |
| 3:116647781-116651499 | 2.13070195 | 0.04979502 | up | yes |
| 7:111989015-112004131 | -1.9814785 | 0.04990831 | down | yes |

**Table S2. Clinical characteristics of the participants included in the validation study**

| Characteristic | Total | non-MetS | MetS | p value |
| --- | --- | --- | --- | --- |
| N | 80 | 40 | 40 |  |
| Serum circRNF111 | 0.96±0.65 | 1.10±0.71 | 0.82±0.56 | 0.0219 |
| Urine circRNF111 | 0.49±0.41 | 0.58±0.40 | 0.40±0.41 | 0.0109 |
| Age (years) | 56.40±6.52 | 56.33±6.12 | 56.82±6.58 | 0.782 |
| Male, n(%) | 41(51.25) | 18(45.00) | 23(57.50) | 0.532 |
| Current smoker, n(%) | 34(42.5) | 8(10) | 26(32.5) | <0.001 |
| Alcohol drinker, n(%) | 25(31.25) | 13(16.25) | 12(15) | 0.117 |
| BMI (kg/m^2^) | 23.65±3.12 | 21.87±2.26 | 25.32±2.53 | <0.001 |
| WC (cm) | 80.56(68.13-91.10) | 72.86(68.13-83.25) | 86.66(83.23-91.10) | <0.001 |
| WHR | 0.89(0.80-0.98) | 0.84(0.80-0.88) | 0.93(0.89-0.98) | <0.001 |
| Body fat (%) | 28.70±6.48 | 26.24±5.66 | 30.89±6.40 | 0.001 |
| SBP (mmHg) | 123.45(110.10-141.52) | 116.68(110.10-123.30) | 124.82(118.82-141.52) | <0.001 |
| DBP (mmHg) | 81.17±9.20 | 78.35±7.86 | 84.30±8.72 | <0.001 |
| HbA1c (%) | 5.66(5.30-6.20) | 5.50(5.13-5.80) | 5.72(5.40-6.20) | 0.011 |
| ALT (U/l) | 18.10(14.00-26.10) | 17.60(10.90-25.40) | 29.15(16.90-33.30) | 0.001 |
| AST (U/l) | 19.00(16.00-23.00) | 19.82(14.70-24.80) | 23.53(17.50-29.60) | 0.164 |
| FPG (mmol/l) | 5.25(4.60-5.54) | 6.72(6.40-7.43) | 7.44(6.83-7.87) | 0.002 |
| 2 h postprandial glucose (mmol/l) | 6.05(4.82-7.12) | 7.59(6.4-8.6) | 10.19(7.62-15.44) | <0.001 |
| FINS (μU/ml) | 10.68(8.18-13.88) | 11.50±3.20 | 18.27±4.10 | <0.001 |
| 2 h INS (μU/ml) | 57.35(37.32-87.90) | 54.42(36.30-83.60) | 81.70(47.70-155.25) | <0.001 |
| HOMA-IR | 2.78(1.72-3.14) | 2.12(1.67-2.88) | 3.53(2.54-5.30) | <0.001 |
| TC (mmol/l) | 5.55±1.02 | 5.35±0.86 | 5.77±1.10 | 0.298 |
| LDL-c (mmol/l) | 2.24(2.00-2.60) | 2.32(1.91-2.74) | 2.30(1.70-2.89) | 0.801 |
| HDL-c (mmol/l) | 1.30(1.01-1.66) | 1.64(1.32-1.96) | 1.08(0.85-1.35) | <0.001 |
| TG (mmol/l) | 1.58(0.88-2.22) | 1.04(0.60-1.45) | 3.25(1.84-3.29) | <0.001 |
| SFA (cm^2^) | 152.74(122.50-192.45) | 150.69(105.70-205.10) | 170.45(122.28-228.26) | <0.001 |
| VFA (cm^2^) | 70.60(47.16-122.20) | 55.48(23.23-87.58) | 118.25(72.25-164.38) | <0.001 |

Data are presented as the mean ± standard deviation, the median with 25-75% interquartile range or n(%). BMI, body mass index; WC, waist circumference; WHR, waist-to-hip ratio; SBP, systolic blood pressure; DBP, diastolic blood pressure; HbA1c, hemoglobin A1c; ALT, alanine transaminase; AST, aspartate transaminase; FPG, fasting plasma glucose; FINS, fasting serum insulin levels; 2h INS, 2h postprandial insulin levels; HOMA-IR, homeostasis model assessment-insulin resistance; TC, total cholesterol; LDL-c, low-density lipoprotein-cholesterol; HDL-c high density lipoprotein-cholesterol; TG, triglyceride; SFA, abdominal subcutaneous fat area; VFA, visceral fat area.

**Table S3****. Spearman correlation analyses of serum circRNF111 with metabolic risk**

|  | Unadjusted | | Adjusted  (age, gender, smoking drinking) | |
| --- | --- | --- | --- | --- |
|  | r | p value | r | p value |
| BMI (kg/m^2^) | -0.232 | 0.024 | -0.255 | 0.020 |
| WC (cm) | -0.187 | 0.062 | -0.221 | 0.032 |
| WHR | -0.167 | 0.097 | -0.161 | 0.146 |
| Fat% (%) | -0.302 | 0.009 | -0.309 | 0.008 |
| SBP (mmHg) | 0.071 | 0.382 | 0.085 | 0.414 |
| DBP (mmHg) | -0.081 | 0.393 | -0.064 | 0.570 |
| HbA1c (%) | -0.252 | 0.012 | -0.234 | 0.034 |
| FPG (mmol/L) | -0.125 | 0.215 | -0.109 | 0.328 |
| 2h PG (mmol/L) | -0.137 | 0.174 | -0.112 | 0.041 |
| FINS (μU/ml) | -0.313 | 0.006 | -0.275 | 0.056 |
| 2h INS (μU/ml) | -0.272 | 0.007 | -0.318 | 0.003 |
| HOMA-IR | -0.287 | 0.013 | -0.279 | 0.012 |
| TC (mmol/L) | -0.249 | 0.025 | -0.271 | 0.018 |
| LDL-c (mmol/L) | -0.085 | 0.405 | -0.111 | 0.316 |
| HDL-c (mmol/L) | 0.244 | 0.022 | 0.220 | 0.006 |
| TG (mmol/L) | -0.305 | 0.002 | -0.287 | 0.010 |
| SFA (cm^2^) | -0.185 | 0.091 | -0.126 | 0.060 |
| VFA (cm^2^) | -0.352 | ＜0.001 | -0.227 | 0.008 |

**Table S4. Spearman correlation analyses of urine circRNF111 with metabolic risk**

|  | Unadjusted | | Adjusted  (age, gender, smoking drinking) | |
| --- | --- | --- | --- | --- |
|  | r | p value | r | p value |
| BMI (kg/m^2^) | -0.282 | 0.006 | -0.308 | 0.002 |
| WC (cm) | -0.172 | 0.086 | -0.208 | 0.018 |
| WHR | -0.124 | 0.221 | -0.191 | 0.066 |
| Fat% (%) | -0.262 | 0.002 | -0.239 | ＜0.001 |
| SBP (mmHg) | 0.171 | 0.182 | 0.185 | 0.114 |
| DBP (mmHg) | 0.044 | 0.633 | 0.094 | 0.366 |
| HbA1c (%) | -0.176 | 0.082 | -0.134 | 0.094 |
| FPG (mmol/L) | -0.225 | 0.015 | -0.209 | 0.026 |
| 2h PG (mmol/L) | -0.217 | 0.028 | -0.206 | 0.038 |
| FINS (μU/ml) | -0.214 | 0.032 | -0.278 | 0.016 |
| 2h INS (μU/ml) | -0.218 | 0.029 | -0.225 | 0.028 |
| HOMA-IR | -0.286 | 0.011 | -0.231 | 0.039 |
| TC (mmol/L) | -0.190 | 0.065 | -0.171 | 0.082 |
| LDL-c (mmol/L) | -0.142 | 0.115 | -0.116 | 0.266 |
| HDL-c (mmol/L) | 0.224 | 0.014 | 0.281 | 0.008 |
| TG (mmol/L) | -0.295 | ＜0.001 | -0.286 | 0.010 |
| SFA (cm^2^) | -0.225 | 0.024 | -0.220 | 0.035 |
| VFA (cm^2^) | -0.204 | 0.041 | -0.279 | 0.006 |

**Table S5. Multiple stepwise regression analyses of independent factors associated with serum circRNF111 levels**

| Independent variables | *β* | SE | Standard *β* | p value |
| --- | --- | --- | --- | --- |
| TG | -0.328 | 0.008 | -0.582 | <0.001 |
| HOMA-IR | -0.016 | 0.025 | -0.286 | 0.002 |
| VFA | -0.019 | 0.016 | -0.275 | 0.020 |

**Table S6. Multiple stepwise regression analyses of independent factors associated with urine circRNF111 levels**

| Independent variables | *β* | SE | Standard *β* | p value |
| --- | --- | --- | --- | --- |
| HOMA-IR | -0.245 | 0.026 | -0.525 | <0.001 |
| Fat% | -0.140 | 0.019 | -0.308 | <0.001 |
| TG | -0.013 | 0.006 | -0.107 | 0.030 |

**Table S7. RNA pull-down assay with a biotinylated circRNF111 probe followed by mass spectrometry (MS) analysis**

| Anti-sense（control probe） | Sense（circRNF111 probe） |
| --- | --- |
| 130kDa Protein 4.1B MEF cell isoform OS=Homo sapiens GN=Epb41l3 PE=1 SV=1 | 16 kDa protein OS=Homo sapiens GN=p16 PE=4 SV=1 |
| 17beta-hydroxysteroid dehydrogenase type 10/short chain L-3-hydroxyacyl-CoA dehydrogenase OS=Homo sapiens GN=Hsd17b10 PE=1 SV=1 | 17beta-hydroxysteroid dehydrogenase type 10/short chain L-3-hydroxyacyl-CoA dehydrogenase OS=Homo sapiens GN=Hsd17b10 PE=1 SV=1 |
| 2900073G15Rik protein (Fragment) OS=Homo sapiens GN=Myl12a PE=2 SV=1 | 2900073G15Rik protein (Fragment) OS=Homo sapiens GN=Myl12a PE=2 SV=1 |
| 3-hydroxyacyl-CoA dehydrogenase type-2 OS=Homo sapiens GN=Hsd17b10 PE=1 SV=1 | 3-hydroxyacyl-CoA dehydrogenase type-2 OS=Homo sapiens GN=Hsd17b10 PE=1 SV=1 |
| 3-hydroxyacyl-CoA dehydrogenase type-2 OS=Homo sapiens GN=Hsd17b10 PE=1 SV=4 | 3-hydroxyacyl-CoA dehydrogenase type-2 OS=Homo sapiens GN=Hsd17b10 PE=1 SV=4 |
| 4.1G protein (Fragment) OS=Homo sapiens GN=Epb41l2 PE=2 SV=1 | 40S ribosomal protein S10 OS=Homo sapiens GN=Rps10 PE=1 SV=1 |
| 40S ribosomal protein S10 OS=Homo sapiens GN=Rps10 PE=1 SV=1 | 40S ribosomal protein S11 (Fragment) OS=Homo sapiens GN=Rps11 PE=1 SV=1 |
| 40S ribosomal protein S11 (Fragment) OS=Homo sapiens GN=Rps11 PE=1 SV=1 | 40S ribosomal protein S11 OS=Homo sapiens GN=Rps11 PE=1 SV=1 |
| 40S ribosomal protein S11 OS=Homo sapiens GN=Rps11 PE=1 SV=1 | 40S ribosomal protein S11 OS=Homo sapiens GN=Rps11 PE=1 SV=3 |
| 40S ribosomal protein S11 OS=Homo sapiens GN=Rps11 PE=1 SV=3 | 40S ribosomal protein S13 OS=Homo sapiens GN=Rps13 PE=1 SV=1 |
| 40S ribosomal protein S13 OS=Homo sapiens GN=Rps13 PE=1 SV=1 | 40S ribosomal protein S13 OS=Homo sapiens GN=Rps13 PE=1 SV=2 |
| 40S ribosomal protein S13 OS=Homo sapiens GN=Rps13 PE=1 SV=2 | 40S ribosomal protein S14 (Fragment) OS=Homo sapiens GN=Rps14 PE=1 SV=1 |
| 40S ribosomal protein S14 (Fragment) OS=Homo sapiens GN=Rps14 PE=1 SV=1 | 40S ribosomal protein S14 OS=Homo sapiens GN=Rps14 PE=1 SV=3 |
| 40S ribosomal protein S14 OS=Homo sapiens GN=Rps14 PE=1 SV=3 | 40S ribosomal protein S15a (Fragment) OS=Homo sapiens GN=Rps15a PE=1 SV=1 |
| 40S ribosomal protein S15a (Fragment) OS=Homo sapiens GN=Rps15a PE=1 SV=1 | 40S ribosomal protein S15a (Fragment) OS=Homo sapiens GN=Rps15a PE=1 SV=2 |
| 40S ribosomal protein S15a (Fragment) OS=Homo sapiens GN=Rps15a PE=1 SV=2 | 40S ribosomal protein S15a OS=Homo sapiens GN=Rps15a PE=1 SV=2 |
| 40S ribosomal protein S15a OS=Homo sapiens GN=Rps15a PE=1 SV=2 | 40S ribosomal protein S16 OS=Homo sapiens GN=Rps16 PE=1 SV=4 |
| 40S ribosomal protein S16 OS=Homo sapiens GN=Rps16 PE=1 SV=4 | 40S ribosomal protein S17 OS=Homo sapiens GN=Rps17 PE=1 SV=2 |
| 40S ribosomal protein S17 OS=Homo sapiens GN=Rps17 PE=1 SV=2 | 40S ribosomal protein S18 OS=Homo sapiens GN=Rps18 PE=3 SV=1 |
| 40S ribosomal protein S18 OS=Homo sapiens GN=Rps18 PE=3 SV=1 | 40S ribosomal protein S19 (Fragment) OS=Homo sapiens GN=Rps19 PE=1 SV=2 |
| 40S ribosomal protein S19 (Fragment) OS=Homo sapiens GN=Rps19 PE=1 SV=2 | 40S ribosomal protein S19 (Fragment) OS=Homo sapiens GN=Rps19 PE=1 SV=8 |
| 40S ribosomal protein S19 (Fragment) OS=Homo sapiens GN=Rps19 PE=1 SV=8 | 40S ribosomal protein S19 OS=Homo sapiens GN=Rps19 PE=1 SV=1 |
| 40S ribosomal protein S19 OS=Homo sapiens GN=Rps19 PE=1 SV=1 | 40S ribosomal protein S19 OS=Homo sapiens GN=Rps19 PE=1 SV=3 |
| 40S ribosomal protein S19 OS=Homo sapiens GN=Rps19 PE=1 SV=3 | 40S ribosomal protein S2 (Fragment) OS=Homo sapiens GN=Rps2 PE=1 SV=1 |
| 40S ribosomal protein S2 (Fragment) OS=Homo sapiens GN=Rps2 PE=1 SV=1 | 40S ribosomal protein S2 OS=Homo sapiens GN=Rps2 PE=1 SV=1 |
| 40S ribosomal protein S2 OS=Homo sapiens GN=Rps2 PE=1 SV=1 | 40S ribosomal protein S2 OS=Homo sapiens GN=Rps2 PE=1 SV=3 |
| 40S ribosomal protein S2 OS=Homo sapiens GN=Rps2 PE=1 SV=3 | 40S ribosomal protein S23 OS=Homo sapiens GN=Rps23 PE=1 SV=3 |
| 40S ribosomal protein S23 OS=Homo sapiens GN=Rps23 PE=1 SV=3 | 40S ribosomal protein S24 OS=Homo sapiens GN=Rps24 PE=1 SV=1 |
| 40S ribosomal protein S24 OS=Homo sapiens GN=Rps24 PE=1 SV=1 | 40S ribosomal protein S24 OS=Homo sapiens GN=Rps24 PE=2 SV=1 |
| 40S ribosomal protein S24 OS=Homo sapiens GN=Rps24 PE=2 SV=1 | 40S ribosomal protein S25 OS=Homo sapiens GN=Rps25 PE=1 SV=1 |
| 40S ribosomal protein S25 OS=Homo sapiens GN=Rps25 PE=1 SV=1 | 40S ribosomal protein S26 OS=Homo sapiens GN=Rps26 PE=1 SV=3 |
| 40S ribosomal protein S26 OS=Homo sapiens GN=Rps26 PE=1 SV=3 | 40S ribosomal protein S27 (Fragment) OS=Homo sapiens GN=Rps27 PE=1 SV=1 |
| 40S ribosomal protein S29 OS=Homo sapiens GN=Rps29 PE=3 SV=2 | 40S ribosomal protein S27 OS=Homo sapiens GN=Rps27 PE=1 SV=3 |
| 40S ribosomal protein S3 OS=Homo sapiens GN=Rps3 PE=1 SV=1 | 40S ribosomal protein S27 OS=Homo sapiens GN=Rps27 PE=3 SV=1 |
| 40S ribosomal protein S30 (Fragment) OS=Homo sapiens GN=fau PE=3 SV=1 | 40S ribosomal protein S27-like OS=Homo sapiens GN=Rps27l PE=1 SV=3 |
| 40S ribosomal protein S30 OS=Homo sapiens GN=Fau PE=1 SV=1 | 40S ribosomal protein S3 OS=Homo sapiens GN=Rps3 PE=1 SV=1 |
| 40S ribosomal protein S30 OS=Homo sapiens PE=2 SV=1 | 40S ribosomal protein S30 (Fragment) OS=Homo sapiens GN=fau PE=3 SV=1 |
| 40S ribosomal protein S3a OS=Homo sapiens GN=Rps3a PE=1 SV=3 | 40S ribosomal protein S30 OS=Homo sapiens GN=Fau PE=1 SV=1 |
| 40S ribosomal protein S3a OS=Homo sapiens GN=Rps3a1 PE=2 SV=1 | 40S ribosomal protein S30 OS=Homo sapiens PE=2 SV=1 |
| 40S ribosomal protein S4 OS=Homo sapiens GN=Gm15013 PE=3 SV=1 | 40S ribosomal protein S3a OS=Homo sapiens GN=Rps3a PE=1 SV=3 |
| 40S ribosomal protein S4 OS=Homo sapiens GN=Rps4l PE=2 SV=1 | 40S ribosomal protein S3a OS=Homo sapiens GN=Rps3a1 PE=2 SV=1 |
| 40S ribosomal protein S4 OS=Homo sapiens GN=Rps4x PE=1 SV=1 | 40S ribosomal protein S4 OS=Homo sapiens GN=Gm15013 PE=3 SV=1 |
| 40S ribosomal protein S4 OS=Homo sapiens GN=Rps4x PE=2 SV=1 | 40S ribosomal protein S4 OS=Homo sapiens GN=Rps4l PE=2 SV=1 |
| 40S ribosomal protein S5 (Fragment) OS=Homo sapiens GN=Rps5 PE=1 SV=1 | 40S ribosomal protein S4 OS=Homo sapiens GN=Rps4x PE=1 SV=1 |
| 40S ribosomal protein S5 OS=Homo sapiens GN=Rps5 PE=1 SV=1 | 40S ribosomal protein S4 OS=Homo sapiens GN=Rps4x PE=2 SV=1 |
| 40S ribosomal protein S5 OS=Homo sapiens GN=Rps5 PE=1 SV=3 | 40S ribosomal protein S5 (Fragment) OS=Homo sapiens GN=Rps5 PE=1 SV=1 |
| 40S ribosomal protein S6 OS=Homo sapiens GN=Rps6 PE=1 SV=1 | 40S ribosomal protein S5 OS=Homo sapiens GN=Rps5 PE=1 SV=1 |
| 40S ribosomal protein S6 OS=Homo sapiens PE=2 SV=1 | 40S ribosomal protein S5 OS=Homo sapiens GN=Rps5 PE=1 SV=3 |
| 40S ribosomal protein S7 OS=Homo sapiens GN=Gm9493 PE=3 SV=1 | 40S ribosomal protein S6 OS=Homo sapiens GN=Rps6 PE=1 SV=1 |
| 40S ribosomal protein S7 OS=Homo sapiens GN=Rps7 PE=2 SV=1 | 40S ribosomal protein S6 OS=Homo sapiens PE=2 SV=1 |
| 40S ribosomal protein S8 (Fragment) OS=Homo sapiens GN=Rps8 PE=2 SV=1 | 40S ribosomal protein S7 OS=Homo sapiens GN=Gm9493 PE=3 SV=1 |
| 40S ribosomal protein S8 OS=Homo sapiens GN=Rps8 PE=1 SV=1 | 40S ribosomal protein S7 OS=Homo sapiens GN=Rps7 PE=2 SV=1 |
| 40S ribosomal protein S8 OS=Homo sapiens GN=Rps8 PE=2 SV=1 | 40S ribosomal protein S8 (Fragment) OS=Homo sapiens GN=Rps8 PE=2 SV=1 |
| 40S ribosomal protein S9 (Fragment) OS=Homo sapiens GN=Rps9 PE=1 SV=1 | 40S ribosomal protein S8 OS=Homo sapiens GN=Rps8 PE=1 SV=1 |
| 40S ribosomal protein S9 (Fragment) OS=Homo sapiens GN=Rps9 PE=1 SV=8 | 40S ribosomal protein S8 OS=Homo sapiens GN=Rps8 PE=2 SV=1 |
| 40S ribosomal protein S9 OS=Homo sapiens GN=Rps9 PE=1 SV=1 | 40S ribosomal protein S9 (Fragment) OS=Homo sapiens GN=Rps9 PE=1 SV=1 |
| 40S ribosomal protein S9 OS=Homo sapiens GN=Rps9 PE=1 SV=3 | 40S ribosomal protein S9 (Fragment) OS=Homo sapiens GN=Rps9 PE=1 SV=8 |
| 40S ribosomal protein SA (Fragment) OS=Homo sapiens GN=Rpsa PE=1 SV=1 | 40S ribosomal protein S9 OS=Homo sapiens GN=Rps9 PE=1 SV=1 |
| 40S ribosomal protein SA OS=Homo sapiens GN=Rpsa PE=1 SV=4 | 40S ribosomal protein S9 OS=Homo sapiens GN=Rps9 PE=1 SV=3 |
| 60kDa 4.1B MEF cell isoform (Fragment) OS=Homo sapiens GN=Epb41l3 PE=2 SV=1 | 40S ribosomal protein SA (Fragment) OS=Homo sapiens GN=Rpsa PE=1 SV=1 |
| 60S acidic ribosomal protein P0 (Fragment) OS=Homo sapiens GN=Rplp0 PE=1 SV=1 | 40S ribosomal protein SA OS=Homo sapiens GN=Rpsa PE=1 SV=4 |
| 60S acidic ribosomal protein P0 OS=Homo sapiens GN=Rplp0 PE=2 SV=1 | 5'-3' exoribonuclease 2 OS=Homo sapiens GN=Xrn2 PE=1 SV=1 |
| 60S ribosomal protein L10 (Fragment) OS=Homo sapiens GN=Rpl10 PE=1 SV=1 | 60S acidic ribosomal protein P0 (Fragment) OS=Homo sapiens GN=Rplp0 PE=1 SV=1 |
| 60S ribosomal protein L10 OS=Homo sapiens GN=Rpl10 PE=1 SV=3 | 60S acidic ribosomal protein P0 OS=Homo sapiens GN=Rplp0 PE=2 SV=1 |
| 60S ribosomal protein L10a OS=Homo sapiens GN=Rpl10a PE=1 SV=1 | 60S ribosomal protein L10 (Fragment) OS=Homo sapiens GN=Rpl10 PE=1 SV=1 |
| 60S ribosomal protein L10a OS=Homo sapiens GN=Rpl10a PE=1 SV=3 | 60S ribosomal protein L10 OS=Homo sapiens GN=Rpl10 PE=1 SV=3 |
| 60S ribosomal protein L10-like OS=Homo sapiens GN=Rpl10l PE=2 SV=1 | 60S ribosomal protein L10-like OS=Homo sapiens GN=Rpl10l PE=2 SV=1 |
| 60S ribosomal protein L11 OS=Homo sapiens GN=Rpl11 PE=1 SV=4 | 60S ribosomal protein L11 OS=Homo sapiens GN=Rpl11 PE=1 SV=4 |
| 60S ribosomal protein L13 OS=Homo sapiens GN=Rpl13 PE=1 SV=3 | 60S ribosomal protein L13 OS=Homo sapiens GN=Rpl13 PE=1 SV=3 |
| 60S ribosomal protein L13 OS=Homo sapiens GN=Rpl13 PE=2 SV=1 | 60S ribosomal protein L13 OS=Homo sapiens GN=Rpl13 PE=2 SV=1 |
| 60S ribosomal protein L14 (Fragment) OS=Homo sapiens GN=Rpl14 PE=1 SV=1 | 60S ribosomal protein L17 OS=Homo sapiens GN=Rpl17 PE=1 SV=1 |
| 60S ribosomal protein L14 OS=Homo sapiens GN=Rpl14 PE=1 SV=3 | 60S ribosomal protein L17 OS=Homo sapiens GN=Rpl17 PE=1 SV=3 |
| 60S ribosomal protein L17 OS=Homo sapiens GN=Rpl17 PE=1 SV=1 | 60S ribosomal protein L18 (Fragment) OS=Homo sapiens GN=Rpl18 PE=1 SV=1 |
| 60S ribosomal protein L17 OS=Homo sapiens GN=Rpl17 PE=1 SV=3 | 60S ribosomal protein L18 OS=Homo sapiens GN=Rpl18 PE=1 SV=1 |
| 60S ribosomal protein L18 (Fragment) OS=Homo sapiens GN=Rpl18 PE=1 SV=1 | 60S ribosomal protein L21 OS=Homo sapiens GN=Rpl21 PE=1 SV=1 |
| 60S ribosomal protein L18 OS=Homo sapiens GN=Rpl18 PE=1 SV=1 | 60S ribosomal protein L21 OS=Homo sapiens GN=Rpl21 PE=1 SV=3 |
| 60S ribosomal protein L18a (Fragment) OS=Homo sapiens GN=Rpl18a PE=1 SV=1 | 60S ribosomal protein L22-like 1 OS=Homo sapiens GN=Rpl22l1 PE=1 SV=1 |
| 60S ribosomal protein L18a OS=Homo sapiens GN=Rpl18a PE=1 SV=1 | 60S ribosomal protein L23 (Fragment) OS=Homo sapiens GN=Rpl23 PE=1 SV=1 |
| 60S ribosomal protein L18a OS=Homo sapiens GN=Rpl18a PE=2 SV=1 | 60S ribosomal protein L23 OS=Homo sapiens GN=Rpl23 PE=1 SV=1 |
| 60S ribosomal protein L21 OS=Homo sapiens GN=Rpl21 PE=1 SV=1 | 60S ribosomal protein L24 OS=Homo sapiens GN=Rpl24 PE=1 SV=2 |
| 60S ribosomal protein L21 OS=Homo sapiens GN=Rpl21 PE=1 SV=3 | 60S ribosomal protein L26 (Fragment) OS=Homo sapiens GN=Rpl26 PE=1 SV=1 |
| 60S ribosomal protein L22-like 1 OS=Homo sapiens GN=Rpl22l1 PE=1 SV=1 | 60S ribosomal protein L26 OS=Homo sapiens GN=Rpl26 PE=1 SV=1 |
| 60S ribosomal protein L23 (Fragment) OS=Homo sapiens GN=Rpl23 PE=1 SV=1 | 60S ribosomal protein L27 (Fragment) OS=Homo sapiens GN=Rpl27 PE=1 SV=1 |
| 60S ribosomal protein L23 OS=Homo sapiens GN=Rpl23 PE=1 SV=1 | 60S ribosomal protein L27 (Fragment) OS=Homo sapiens PE=2 SV=1 |
| 60S ribosomal protein L24 OS=Homo sapiens GN=Rpl24 PE=1 SV=2 | 60S ribosomal protein L27 OS=Homo sapiens GN=Rpl27 PE=1 SV=1 |
| 60S ribosomal protein L26 (Fragment) OS=Homo sapiens GN=Rpl26 PE=1 SV=1 | 60S ribosomal protein L27a OS=Homo sapiens GN=Rpl27a PE=1 SV=5 |
| 60S ribosomal protein L26 OS=Homo sapiens GN=Rpl26 PE=1 SV=1 | 60S ribosomal protein L29 (Fragment) OS=Homo sapiens GN=Rpl29 PE=3 SV=1 |
| 60S ribosomal protein L27 (Fragment) OS=Homo sapiens GN=Rpl27 PE=1 SV=1 | 60S ribosomal protein L29 (Fragment) OS=Homo sapiens GN=Rpl29 PE=4 SV=1 |
| 60S ribosomal protein L27 (Fragment) OS=Homo sapiens PE=2 SV=1 | 60S ribosomal protein L29 OS=Homo sapiens GN=Gm17669 PE=3 SV=1 |
| 60S ribosomal protein L27 OS=Homo sapiens GN=Rpl27 PE=1 SV=1 | 60S ribosomal protein L29 OS=Homo sapiens GN=Gm3550 PE=3 SV=1 |
| 60S ribosomal protein L27a OS=Homo sapiens GN=Rpl27a PE=1 SV=5 | 60S ribosomal protein L29 OS=Homo sapiens GN=Gm5218 PE=3 SV=1 |
| 60S ribosomal protein L3 (Fragment) OS=Homo sapiens GN=Rpl3 PE=1 SV=1 | 60S ribosomal protein L29 OS=Homo sapiens GN=Rpl29 PE=1 SV=2 |
| 60S ribosomal protein L3 OS=Homo sapiens GN=Rpl3 PE=1 SV=1 | 60S ribosomal protein L3 (Fragment) OS=Homo sapiens GN=Rpl3 PE=1 SV=1 |
| 60S ribosomal protein L3 OS=Homo sapiens GN=Rpl3 PE=1 SV=3 | 60S ribosomal protein L3 OS=Homo sapiens GN=Rpl3 PE=1 SV=1 |
| 60S ribosomal protein L31 OS=Homo sapiens GN=Rpl31 PE=1 SV=1 | 60S ribosomal protein L3 OS=Homo sapiens GN=Rpl3 PE=1 SV=3 |
| 60S ribosomal protein L32 OS=Homo sapiens GN=Rpl32 PE=1 SV=2 | 60S ribosomal protein L31 OS=Homo sapiens GN=Rpl31 PE=1 SV=1 |
| 60S ribosomal protein L35 OS=Homo sapiens GN=Rpl35 PE=1 SV=1 | 60S ribosomal protein L34 OS=Homo sapiens GN=Rpl34 PE=1 SV=2 |
| 60S ribosomal protein L36 OS=Homo sapiens GN=Rpl36 PE=1 SV=1 | 60S ribosomal protein L35 OS=Homo sapiens GN=Rpl35 PE=1 SV=1 |
| 60S ribosomal protein L36 OS=Homo sapiens GN=Rpl36 PE=2 SV=1 | 60S ribosomal protein L36a OS=Homo sapiens GN=Rpl36a PE=1 SV=2 |
| 60S ribosomal protein L36 OS=Homo sapiens GN=Rpl36 PE=3 SV=2 | 60S ribosomal protein L6 (Fragment) OS=Homo sapiens GN=Rpl6 PE=1 SV=1 |
| 60S ribosomal protein L36a OS=Homo sapiens GN=Rpl36a PE=1 SV=2 | 60S ribosomal protein L6 OS=Homo sapiens GN=Rpl6 PE=1 SV=3 |
| 60S ribosomal protein L5 (Fragment) OS=Homo sapiens GN=Rpl5 PE=1 SV=1 | 60S ribosomal protein L6 OS=Homo sapiens GN=Rpl6 PE=2 SV=1 |
| 60S ribosomal protein L5 OS=Homo sapiens GN=Rpl5 PE=1 SV=3 | 60S ribosomal protein L7 (Fragment) OS=Homo sapiens GN=Rpl7 PE=1 SV=1 |
| 60S ribosomal protein L6 (Fragment) OS=Homo sapiens GN=Rpl6 PE=1 SV=1 | 60S ribosomal protein L7 OS=Homo sapiens GN=Rpl7 PE=1 SV=2 |
| 60S ribosomal protein L6 OS=Homo sapiens GN=Rpl6 PE=1 SV=3 | 60S ribosomal protein L7a OS=Homo sapiens GN=Rpl7a PE=1 SV=2 |
| 60S ribosomal protein L6 OS=Homo sapiens GN=Rpl6 PE=2 SV=1 | 60S ribosomal protein L8 OS=Homo sapiens GN=Rpl8 PE=1 SV=2 |
| 60S ribosomal protein L7 (Fragment) OS=Homo sapiens GN=Rpl7 PE=1 SV=1 | 60S ribosomal protein L9 (Fragment) OS=Homo sapiens GN=Rpl9 PE=1 SV=1 |
| 60S ribosomal protein L7 OS=Homo sapiens GN=Rpl7 PE=1 SV=2 | 60S ribosomal protein L9 OS=Homo sapiens GN=Rpl9 PE=1 SV=1 |
| 60S ribosomal protein L7a OS=Homo sapiens GN=Rpl7a PE=1 SV=2 | 60S ribosomal protein L9 OS=Homo sapiens GN=Rpl9 PE=1 SV=2 |
| 60S ribosomal protein L8 OS=Homo sapiens GN=Rpl8 PE=1 SV=2 | 78 kDa glucose-regulated protein OS=Homo sapiens GN=Hspa5 PE=1 SV=3 |
| 60S ribosomal protein L9 (Fragment) OS=Homo sapiens GN=Rpl9 PE=1 SV=1 | Acin1 protein OS=Homo sapiens GN=Acin1 PE=1 SV=1 |
| 60S ribosomal protein L9 OS=Homo sapiens GN=Rpl9 PE=1 SV=1 | Actg2 protein OS=Homo sapiens GN=Actg2 PE=2 SV=1 |
| 60S ribosomal protein L9 OS=Homo sapiens GN=Rpl9 PE=1 SV=2 | Actin, alpha cardiac muscle 1 (Fragment) OS=Homo sapiens GN=Actc1 PE=4 SV=1 |
| 78 kDa glucose-regulated protein OS=Homo sapiens GN=Hspa5 PE=1 SV=3 | Actin, alpha cardiac muscle 1 OS=Homo sapiens GN=Actc1 PE=1 SV=1 |
| Acetyl-CoA carboxylase 1 (Fragment) OS=Homo sapiens GN=Acaca PE=1 SV=1 | Actin, alpha skeletal muscle (Fragment) OS=Homo sapiens GN=Acta1 PE=3 SV=1 |
| Acetyl-CoA carboxylase 1 OS=Homo sapiens GN=Acaca PE=1 SV=1 | Actin, alpha skeletal muscle OS=Homo sapiens GN=Acta1 PE=1 SV=1 |
| Acetyl-CoA carboxylase 2 (Fragment) OS=Homo sapiens GN=Acacb PE=1 SV=1 | Actin, aortic smooth muscle OS=Homo sapiens GN=Acta2 PE=1 SV=1 |
| Acetyl-CoA carboxylase 2 OS=Homo sapiens GN=Acacb PE=1 SV=1 | Actin, cytoplasmic 1 (Fragment) OS=Homo sapiens GN=Actb PE=1 SV=1 |
| Acetyl-CoA carboxylase 280 (Fragment) OS=Homo sapiens GN=Acacb PE=2 SV=2 | Actin, cytoplasmic 1 OS=Homo sapiens GN=Actb PE=1 SV=1 |
| Actg2 protein OS=Homo sapiens GN=Actg2 PE=2 SV=1 | Actin, cytoplasmic 2 (Fragment) OS=Homo sapiens GN=Actg1 PE=1 SV=1 |
| Actin, alpha cardiac muscle 1 (Fragment) OS=Homo sapiens GN=Actc1 PE=4 SV=1 | Actin, cytoplasmic 2 OS=Homo sapiens GN=Actg1 PE=1 SV=1 |
| Actin, alpha cardiac muscle 1 OS=Homo sapiens GN=Actc1 PE=1 SV=1 | Actin, gamma-enteric smooth muscle (Fragment) OS=Homo sapiens GN=Actg2 PE=1 SV=1 |
| Actin, alpha skeletal muscle (Fragment) OS=Homo sapiens GN=Acta1 PE=3 SV=1 | Actin, gamma-enteric smooth muscle (Fragment) OS=Homo sapiens GN=Actg2 PE=1 SV=2 |
| Actin, alpha skeletal muscle OS=Homo sapiens GN=Acta1 PE=1 SV=1 | Actin, gamma-enteric smooth muscle (Fragment) OS=Homo sapiens GN=Actg2 PE=3 SV=1 |
| Actin, aortic smooth muscle OS=Homo sapiens GN=Acta2 PE=1 SV=1 | Actin, gamma-enteric smooth muscle OS=Homo sapiens GN=Actg2 PE=1 SV=1 |
| Actin, cytoplasmic 1 (Fragment) OS=Homo sapiens GN=Actb PE=1 SV=1 | Activated RNA polymerase II transcriptional coactivator p15 OS=Homo sapiens GN=Sub1 PE=1 SV=3 |
| Actin, cytoplasmic 1 OS=Homo sapiens GN=Actb PE=1 SV=1 | ADP/ATP translocase 1 OS=Homo sapiens GN=Slc25a4 PE=1 SV=4 |
| Actin, cytoplasmic 2 (Fragment) OS=Homo sapiens GN=Actg1 PE=1 SV=1 | ADP/ATP translocase 2 OS=Homo sapiens GN=Slc25a5 PE=1 SV=3 |
| Actin, cytoplasmic 2 OS=Homo sapiens GN=Actg1 PE=1 SV=1 | Albumin 1 OS=Homo sapiens GN=Alb PE=1 SV=1 |
| Actin, gamma-enteric smooth muscle (Fragment) OS=Homo sapiens GN=Actg2 PE=1 SV=1 | Alpha-actin (AA 27-375) (Fragment) OS=Homo sapiens GN=Actc1 PE=2 SV=1 |
| Actin, gamma-enteric smooth muscle (Fragment) OS=Homo sapiens GN=Actg2 PE=1 SV=2 | Alpha-actin (Aa 40-375) (Fragment) OS=Homo sapiens GN=Acta1 PE=2 SV=1 |
| Actin, gamma-enteric smooth muscle (Fragment) OS=Homo sapiens GN=Actg2 PE=3 SV=1 | Alpha-internexin OS=Homo sapiens GN=Ina PE=1 SV=3 |
| Actin, gamma-enteric smooth muscle OS=Homo sapiens GN=Actg2 PE=1 SV=1 | Alpha-tubulin (Fragment) OS=Homo sapiens GN=Tuba1b PE=2 SV=1 |
| Activated RNA polymerase II transcriptional coactivator p15 OS=Homo sapiens GN=Sub1 PE=1 SV=3 | Aly/REF export factor 2 OS=Homo sapiens GN=Alyref2 PE=1 SV=1 |
| ADP/ATP translocase 1 OS=Homo sapiens GN=Slc25a4 PE=1 SV=4 | Apoptotic chromatin condensation inducer 1 OS=Homo sapiens GN=Acin1 PE=2 SV=1 |
| ADP/ATP translocase 2 OS=Homo sapiens GN=Slc25a5 PE=1 SV=3 | Apoptotic chromatin condensation inducer in the nucleus (Fragment) OS=Homo sapiens GN=Acin1 PE=1 SV=1 |
| Albumin 1 OS=Homo sapiens GN=Alb PE=1 SV=1 | Apoptotic chromatin condensation inducer in the nucleus OS=Homo sapiens GN=Acin1 PE=1 SV=1 |
| Alpha-actin (AA 27-375) (Fragment) OS=Homo sapiens GN=Actc1 PE=2 SV=1 | Apoptotic chromatin condensation inducer in the nucleus OS=Homo sapiens GN=Acin1 PE=1 SV=3 |
| Alpha-actin (Aa 40-375) (Fragment) OS=Homo sapiens GN=Acta1 PE=2 SV=1 | Arginine--tRNA ligase, cytoplasmic OS=Homo sapiens GN=Rars PE=1 SV=2 |
| Alpha-tubulin (Fragment) OS=Homo sapiens GN=Tuba1b PE=2 SV=1 | Argonaute RISC catalytic component 2 OS=Homo sapiens GN=Ago2 PE=1 SV=1 |
| Aly/REF export factor 2 OS=Homo sapiens GN=Alyref2 PE=1 SV=1 | Argonaute RISC catalytic component 2 OS=Homo sapiens GN=Ago2 PE=1 SV=3 |
| AP-3 complex subunit delta-1 OS=Homo sapiens GN=Ap3d1 PE=1 SV=1 | ATP synthase subunit O, mitochondrial (Fragment) OS=Homo sapiens GN=Atp5o PE=1 SV=1 |
| Ap3d1 protein (Fragment) OS=Homo sapiens GN=Ap3d1 PE=2 SV=1 | ATP synthase subunit O, mitochondrial (Fragment) OS=Homo sapiens GN=Atp5o PE=1 SV=8 |
| Arf-GAP with SH3 domain, ANK repeat and PH domain-containing protein 2 OS=Homo sapiens GN=Asap2 PE=1 SV=1 | ATP synthase subunit O, mitochondrial OS=Homo sapiens GN=Atp5o PE=1 SV=1 |
| Arf-GAP with SH3 domain, ANK repeat and PH domain-containing protein 2 OS=Homo sapiens GN=Asap2 PE=1 SV=2 | ATP-dependent RNA helicase DDX3Y OS=Homo sapiens GN=Ddx3y PE=1 SV=2 |
| Arf-GAP with SH3 domain, ANK repeat and PH domain-containing protein 2 OS=Homo sapiens GN=Asap2 PE=1 SV=3 | A-X actin OS=Homo sapiens GN=Actb PE=2 SV=1 |
| ATP synthase subunit O, mitochondrial (Fragment) OS=Homo sapiens GN=Atp5o PE=1 SV=8 | Bcl-2-associated transcription factor 1 (Fragment) OS=Homo sapiens GN=Bclaf1 PE=1 SV=1 |
| ATP synthase subunit O, mitochondrial OS=Homo sapiens GN=Atp5o PE=1 SV=1 | Bcl-2-associated transcription factor 1 OS=Homo sapiens GN=Bclaf1 PE=1 SV=1 |
| ATP-dependent RNA helicase A OS=Homo sapiens GN=Dhx9 PE=1 SV=1 | Bcl-2-associated transcription factor 1 OS=Homo sapiens GN=Bclaf1 PE=1 SV=2 |
| ATP-dependent RNA helicase A OS=Homo sapiens GN=Dhx9 PE=1 SV=2 | Beta-actin (Fragment) OS=Homo sapiens GN=Actb PE=2 SV=1 |
| ATP-dependent RNA helicase DDX3Y OS=Homo sapiens GN=Ddx3y PE=1 SV=2 | Beta-actin FE-3 (Fragment) OS=Homo sapiens GN=Actb PE=2 SV=1 |
| A-X actin OS=Homo sapiens GN=Actb PE=2 SV=1 | Beta-actin-like protein 2 OS=Homo sapiens GN=Actbl2 PE=1 SV=1 |
| Band 4.1-like protein 2 (Fragment) OS=Homo sapiens GN=Epb41l2 PE=1 SV=1 | Beta-tropomyosin OS=Homo sapiens GN=Tpm2 PE=2 SV=1 |
| Band 4.1-like protein 2 OS=Homo sapiens GN=Epb41l2 PE=1 SV=1 | C. elegans ceh-10 homeo domain containing homolog, isoform CRA_a OS=Homo sapiens GN=Vsx2 PE=2 SV=1 |
| Band 4.1-like protein 2 OS=Homo sapiens GN=Epb41l2 PE=1 SV=2 | C. elegans ceh-10 homeo domain containing homolog, isoform CRA_b OS=Homo sapiens GN=Vsx2 PE=2 SV=2 |
| Band 4.1-like protein 3 (Fragment) OS=Homo sapiens GN=Epb41l3 PE=1 SV=1 | Caprin-1 (Fragment) OS=Homo sapiens GN=Caprin1 PE=1 SV=1 |
| Band 4.1-like protein 3 OS=Homo sapiens GN=Epb41l3 PE=1 SV=1 | Caprin-1 OS=Homo sapiens GN=Caprin1 PE=1 SV=2 |
| Beta-actin (Fragment) OS=Homo sapiens GN=Actb PE=2 SV=1 | CArG-binding factor A (Fragment) OS=Homo sapiens GN=Hnrnpab PE=2 SV=1 |
| Beta-actin FE-3 (Fragment) OS=Homo sapiens GN=Actb PE=2 SV=1 | Caveolae-associated protein 1 OS=Homo sapiens GN=Cavin1 PE=1 SV=1 |
| Beta-actin-like protein 2 OS=Homo sapiens GN=Actbl2 PE=1 SV=1 | Cdc2a protein (Fragment) OS=Homo sapiens GN=Cdk1 PE=2 SV=1 |
| Bifunctional glutamate/proline--tRNA ligase OS=Homo sapiens GN=Eprs PE=1 SV=4 | Cell cycle p34 CDC2 kinase protein (Fragment) OS=Homo sapiens PE=4 SV=1 |
| Brain cDNA, clone MNCb-1272, similar to Homo sapiens chaperonin subunit 2 (beta) (Cct2), mRNA OS=Homo sapiens GN=Cct2 PE=2 SV=1 | Cell division cycle 5-like protein OS=Homo sapiens GN=Cdc5l PE=1 SV=2 |
| C. elegans ceh-10 homeo domain containing homolog, isoform CRA_a OS=Homo sapiens GN=Vsx2 PE=2 SV=1 | Cell growth-regulating nucleolar protein (Fragment) OS=Homo sapiens GN=Lyar PE=1 SV=1 |
| C. elegans ceh-10 homeo domain containing homolog, isoform CRA_b OS=Homo sapiens GN=Vsx2 PE=2 SV=2 | Cell growth-regulating nucleolar protein OS=Homo sapiens GN=Lyar PE=1 SV=2 |
| Caprin-1 OS=Homo sapiens GN=Caprin1 PE=1 SV=2 | Chromodomain-helicase-DNA-binding protein 2 (Fragment) OS=Homo sapiens GN=Chd2 PE=1 SV=1 |
| Caveolae-associated protein 1 OS=Homo sapiens GN=Cavin1 PE=1 SV=1 | Chromodomain-helicase-DNA-binding protein 2 (Fragment) OS=Homo sapiens GN=Chd2 PE=1 SV=2 |
| Cdc2a protein (Fragment) OS=Homo sapiens GN=Cdk1 PE=2 SV=1 | Chromodomain-helicase-DNA-binding protein 2 OS=Homo sapiens GN=Chd2 PE=1 SV=1 |
| Cell cycle p34 CDC2 kinase protein (Fragment) OS=Homo sapiens PE=4 SV=1 | Cleavage and polyadenylation specificity factor subunit 5 OS=Homo sapiens GN=Nudt21 PE=1 SV=1 |
| Cell division cycle 5-like protein OS=Homo sapiens GN=Cdc5l PE=1 SV=2 | Cleavage and polyadenylation-specificity factor subunit 5 (Fragment) OS=Homo sapiens GN=Nudt21 PE=1 SV=1 |
| Chaperonin subunit 2 (Beta), isoform CRA_a OS=Homo sapiens GN=Cct2 PE=1 SV=1 | Cleavage and polyadenylation-specificity factor subunit 5 OS=Homo sapiens GN=Nudt21 PE=1 SV=1 |
| Chromodomain helicase DNA-binding protein 3 (Fragment) OS=Homo sapiens GN=Chd3 PE=1 SV=1 | Coatomer subunit alpha OS=Homo sapiens GN=Copa PE=1 SV=1 |
| Chromodomain helicase DNA-binding protein 3 OS=Homo sapiens GN=Chd3 PE=1 SV=1 | Coatomer subunit alpha OS=Homo sapiens GN=Copa PE=1 SV=2 |
| Chromodomain-helicase-DNA-binding protein 4 OS=Homo sapiens GN=Chd4 PE=1 SV=1 | Coatomer subunit alpha OS=Homo sapiens GN=Copa PE=2 SV=1 |
| Cleavage and polyadenylation specificity factor subunit 5 OS=Homo sapiens GN=Nudt21 PE=1 SV=1 | Copa protein (Fragment) OS=Homo sapiens GN=Copa PE=2 SV=1 |
| Cleavage and polyadenylation-specificity factor subunit 5 (Fragment) OS=Homo sapiens GN=Nudt21 PE=1 SV=1 | Csda protein OS=Homo sapiens GN=Ybx3 PE=2 SV=1 |
| Cleavage and polyadenylation-specificity factor subunit 5 OS=Homo sapiens GN=Nudt21 PE=1 SV=1 | Cyclin-dependent kinase 1 (Fragment) OS=Homo sapiens GN=Cdk1 PE=1 SV=1 |
| Coatomer subunit gamma-1 OS=Homo sapiens GN=Copg1 PE=1 SV=1 | Cyclin-dependent kinase 1 OS=Homo sapiens GN=Cdk1 PE=1 SV=3 |
| Csda protein OS=Homo sapiens GN=Ybx3 PE=2 SV=1 | Cyclin-dependent kinase 12 OS=Homo sapiens GN=Cdk12 PE=1 SV=2 |
| Cyclin-dependent kinase 1 (Fragment) OS=Homo sapiens GN=Cdk1 PE=1 SV=1 | Cyclin-dependent kinase 13 OS=Homo sapiens GN=Cdk13 PE=1 SV=1 |
| Cyclin-dependent kinase 1 OS=Homo sapiens GN=Cdk1 PE=1 SV=3 | Cyclin-dependent kinase 13 OS=Homo sapiens GN=Cdk13 PE=1 SV=3 |
| Cyclin-dependent kinase 12 OS=Homo sapiens GN=Cdk12 PE=1 SV=2 | Cyclin-dependent kinase 14 OS=Homo sapiens GN=Cdk14 PE=1 SV=1 |
| Cyclin-dependent kinase 13 OS=Homo sapiens GN=Cdk13 PE=1 SV=1 | Cyclin-dependent kinase 14 OS=Homo sapiens GN=Cdk14 PE=1 SV=2 |
| Cyclin-dependent kinase 13 OS=Homo sapiens GN=Cdk13 PE=1 SV=3 | Cyclin-dependent kinase 15 OS=Homo sapiens GN=Cdk15 PE=1 SV=1 |
| Cyclin-dependent kinase 14 OS=Homo sapiens GN=Cdk14 PE=1 SV=1 | Cyclin-dependent kinase 15 OS=Homo sapiens GN=Cdk15 PE=2 SV=2 |
| Cyclin-dependent kinase 14 OS=Homo sapiens GN=Cdk14 PE=1 SV=2 | Cyclin-dependent kinase 17 (Fragment) OS=Homo sapiens GN=Cdk17 PE=1 SV=1 |
| Cyclin-dependent kinase 15 OS=Homo sapiens GN=Cdk15 PE=1 SV=1 | Cyclin-dependent kinase 17 OS=Homo sapiens GN=Cdk17 PE=1 SV=2 |
| Cyclin-dependent kinase 15 OS=Homo sapiens GN=Cdk15 PE=2 SV=2 | Cyclin-dependent kinase 2, isoform CRA_b OS=Homo sapiens GN=Cdk2 PE=2 SV=1 |
| Cyclin-dependent kinase 17 (Fragment) OS=Homo sapiens GN=Cdk17 PE=1 SV=1 | Cyclin-dependent kinase 20 (Fragment) OS=Homo sapiens GN=Cdk20 PE=1 SV=1 |
| Cyclin-dependent kinase 17 OS=Homo sapiens GN=Cdk17 PE=1 SV=2 | Cyclin-dependent kinase 20 (Fragment) OS=Homo sapiens GN=Cdk20 PE=4 SV=1 |
| Cyclin-dependent kinase 2, isoform CRA_b OS=Homo sapiens GN=Cdk2 PE=2 SV=1 | Cyclin-dependent kinase 20 OS=Homo sapiens GN=Cdk20 PE=1 SV=1 |
| Cyclin-dependent kinase 20 (Fragment) OS=Homo sapiens GN=Cdk20 PE=1 SV=1 | Cyclin-dependent kinase 3 OS=Homo sapiens GN=Cdk3 PE=1 SV=2 |
| Cyclin-dependent kinase 20 (Fragment) OS=Homo sapiens GN=Cdk20 PE=4 SV=1 | Cyclin-dependent kinase 4 (Fragment) OS=Homo sapiens GN=Cdk4 PE=1 SV=1 |
| Cyclin-dependent kinase 20 OS=Homo sapiens GN=Cdk20 PE=1 SV=1 | Cyclin-dependent kinase 4 OS=Homo sapiens GN=Cdk4 PE=1 SV=1 |
| Cyclin-dependent kinase 3 OS=Homo sapiens GN=Cdk3 PE=1 SV=2 | Cyclin-dependent kinase 5, isoform CRA_c OS=Homo sapiens GN=Cdk5 PE=1 SV=1 |
| Cyclin-dependent kinase 4 (Fragment) OS=Homo sapiens GN=Cdk4 PE=1 SV=1 | Cyclin-dependent kinase 6 OS=Homo sapiens GN=Cdk6 PE=1 SV=2 |
| Cyclin-dependent kinase 4 OS=Homo sapiens GN=Cdk4 PE=1 SV=1 | Cyclin-dependent kinase 9 OS=Homo sapiens GN=Cdk9 PE=1 SV=1 |
| Cyclin-dependent kinase 5, isoform CRA_c OS=Homo sapiens GN=Cdk5 PE=1 SV=1 | Cyclin-dependent-like kinase 5 OS=Homo sapiens GN=Cdk5 PE=1 SV=1 |
| Cyclin-dependent kinase 6 OS=Homo sapiens GN=Cdk6 PE=1 SV=2 | Cytokeratin KRT2-6HF (Fragment) OS=Homo sapiens GN=Krt75 PE=2 SV=1 |
| Cyclin-dependent kinase 9 OS=Homo sapiens GN=Cdk9 PE=1 SV=1 | Cytoskeletal beta-actin (Fragment) OS=Homo sapiens GN=Actb PE=2 SV=1 |
| Cyclin-dependent-like kinase 5 OS=Homo sapiens GN=Cdk5 PE=1 SV=1 | Cytoskeleton-associated protein 4 OS=Homo sapiens GN=Ckap4 PE=1 SV=2 |
| Cytokeratin KRT2-6HF (Fragment) OS=Homo sapiens GN=Krt75 PE=2 SV=1 | DbpA murine homologue OS=Homo sapiens GN=Ybx3 PE=2 SV=1 |
| Cytoskeletal beta-actin (Fragment) OS=Homo sapiens GN=Actb PE=2 SV=1 | Ddx3x protein OS=Homo sapiens GN=Ddx3x PE=2 SV=1 |
| Cytoskeleton-associated protein 4 OS=Homo sapiens GN=Ckap4 PE=1 SV=2 | Ddx5 protein (Fragment) OS=Homo sapiens GN=Ddx5 PE=2 SV=1 |
| DbpA murine homologue OS=Homo sapiens GN=Ybx3 PE=2 SV=1 | DEAD (Asp-Glu-Ala-Asp) box polypeptide 17, isoform CRA_a OS=Homo sapiens GN=Ddx17 PE=1 SV=1 |
| Ddx3x protein OS=Homo sapiens GN=Ddx3x PE=2 SV=1 | DEAD (Asp-Glu-Ala-Asp) box polypeptide 5 OS=Homo sapiens GN=Ddx5 PE=1 SV=1 |
| Ddx5 protein (Fragment) OS=Homo sapiens GN=Ddx5 PE=2 SV=1 | DEAD (Asp-Glu-Ala-Asp) box polypeptide 5 OS=Homo sapiens GN=Ddx5 PE=2 SV=1 |
| DEAD (Asp-Glu-Ala-Asp) box polypeptide 17 OS=Homo sapiens GN=Ddx17 PE=2 SV=2 | Description |
| DEAD (Asp-Glu-Ala-Asp) box polypeptide 17, isoform CRA_a OS=Homo sapiens GN=Ddx17 PE=1 SV=1 | Desmin (Fragment) OS=Homo sapiens GN=des PE=4 SV=1 |
| DEAD (Asp-Glu-Ala-Asp) box polypeptide 5 OS=Homo sapiens GN=Ddx5 PE=1 SV=1 | Desmin OS=Homo sapiens GN=Des PE=1 SV=3 |
| DEAD (Asp-Glu-Ala-Asp) box polypeptide 5 OS=Homo sapiens GN=Ddx5 PE=2 SV=1 | Developmentally regulated GTP binding protein 1 OS=Homo sapiens GN=Drg1 PE=2 SV=1 |
| Dedicator of cytokinesis protein 7 (Fragment) OS=Homo sapiens GN=Dock7 PE=1 SV=1 | Developmentally-regulated GTP-binding protein 2 OS=Homo sapiens GN=Drg2 PE=1 SV=1 |
| Dedicator of cytokinesis protein 7 (Fragment) OS=Homo sapiens GN=Dock7 PE=1 SV=2 | DNA (cytosine-5)-methyltransferase 1 OS=Homo sapiens GN=Dnmt1 PE=1 SV=5 |
| Dedicator of cytokinesis protein 7 OS=Homo sapiens GN=Dock7 PE=1 SV=1 | DNA (cytosine-5)-methyltransferase OS=Homo sapiens GN=Dnmt1 PE=2 SV=1 |
| Dedicator of cytokinesis protein 7 OS=Homo sapiens GN=Dock7 PE=1 SV=2 | DNA-(apurinic or apyrimidinic site) lyase (Fragment) OS=Homo sapiens GN=Apex1 PE=1 SV=1 |
| Dedicator of cytokinesis protein 7 OS=Homo sapiens GN=Dock7 PE=1 SV=3 | DNA-(apurinic or apyrimidinic site) lyase OS=Homo sapiens GN=Apex1 PE=1 SV=1 |
| Description | Eef2 protein (Fragment) OS=Homo sapiens GN=Eef2 PE=2 SV=1 |
| Desmin (Fragment) OS=Homo sapiens GN=des PE=4 SV=1 | EG620155 protein OS=Homo sapiens GN=Gm6133 PE=2 SV=1 |
| Desmin OS=Homo sapiens GN=Des PE=1 SV=3 | EG627828 protein OS=Homo sapiens GN=Gm6793 PE=1 SV=1 |
| Developmentally regulated GTP binding protein 1 OS=Homo sapiens GN=Drg1 PE=2 SV=1 | Eif2s2 protein OS=Homo sapiens GN=Eif2s2 PE=1 SV=1 |
| DNA-(apurinic or apyrimidinic site) lyase (Fragment) OS=Homo sapiens GN=Apex1 PE=1 SV=1 | Eif2s3x protein (Fragment) OS=Homo sapiens GN=Eif2s3x PE=2 SV=1 |
| DNA-(apurinic or apyrimidinic site) lyase OS=Homo sapiens GN=Apex1 PE=1 SV=1 | Eif5b protein OS=Homo sapiens GN=Eif5b PE=2 SV=1 |
| E3 ubiquitin-protein ligase TRIP12 (Fragment) OS=Homo sapiens GN=Trip12 PE=1 SV=1 | Elongation factor 1-alpha 1 (Fragment) OS=Homo sapiens GN=Eef1a1 PE=1 SV=1 |
| E3 ubiquitin-protein ligase TRIP12 OS=Homo sapiens GN=Trip12 PE=1 SV=1 | Elongation factor 1-alpha 2 OS=Homo sapiens GN=Eef1a2 PE=1 SV=1 |
| E3 UFM1-protein ligase 1 OS=Homo sapiens GN=Ufl1 PE=1 SV=2 | Elongation factor 1-alpha OS=Homo sapiens GN=Eef1a1 PE=1 SV=1 |
| Eef2 protein (Fragment) OS=Homo sapiens GN=Eef2 PE=2 SV=1 | Elongation factor 1-alpha OS=Homo sapiens GN=Eef1a1 PE=2 SV=1 |
| EG620155 protein OS=Homo sapiens GN=Gm6133 PE=2 SV=1 | Elongation factor 2 (Fragment) OS=Homo sapiens GN=Eef2 PE=2 SV=1 |
| Eif1a protein OS=Homo sapiens GN=Eif1a PE=2 SV=1 | Elongation factor 2 OS=Homo sapiens GN=Eef2 PE=1 SV=2 |
| Eif2s2 protein OS=Homo sapiens GN=Eif2s2 PE=1 SV=1 | Epiplakin OS=Homo sapiens GN=Eppk1 PE=1 SV=2 |
| Eif2s3x protein (Fragment) OS=Homo sapiens GN=Eif2s3x PE=2 SV=1 | Eukaryotic translation initiation factor 2 subunit 1 OS=Homo sapiens GN=Eif2s1 PE=1 SV=3 |
| Eif5b protein OS=Homo sapiens GN=Eif5b PE=2 SV=1 | Eukaryotic translation initiation factor 2 subunit 2 (Fragment) OS=Homo sapiens GN=Eif2s2 PE=1 SV=1 |
| Elongation factor 1-alpha 1 (Fragment) OS=Homo sapiens GN=Eef1a1 PE=1 SV=1 | Eukaryotic translation initiation factor 2 subunit 3, X-linked OS=Homo sapiens GN=Eif2s3x PE=1 SV=1 |
| Elongation factor 1-alpha 2 OS=Homo sapiens GN=Eef1a2 PE=1 SV=1 | Eukaryotic translation initiation factor 2 subunit 3, Y-linked OS=Homo sapiens GN=Eif2s3y PE=1 SV=2 |
| Elongation factor 1-alpha OS=Homo sapiens GN=Eef1a1 PE=1 SV=1 | Eukaryotic translation initiation factor 2, subunit 3, structural gene X-linked OS=Homo sapiens GN=Eif2s3x PE=1 SV=1 |
| Elongation factor 1-alpha OS=Homo sapiens GN=Eef1a1 PE=2 SV=1 | Eukaryotic translation initiation factor 2, subunit 3, structural gene Y-linked, isoform CRA_b OS=Homo sapiens GN=Eif2s3y PE=2 SV=1 |
| Elongation factor 2 (Fragment) OS=Homo sapiens GN=Eef2 PE=2 SV=1 | Eukaryotic translation initiation factor 3 subunit A (Fragment) OS=Homo sapiens GN=Eif3a PE=2 SV=1 |
| Elongation factor 2 OS=Homo sapiens GN=Eef2 PE=1 SV=2 | Eukaryotic translation initiation factor 3 subunit A OS=Homo sapiens GN=Eif3a PE=1 SV=5 |
| Epb4.1l2 protein (Fragment) OS=Homo sapiens GN=Epb41l2 PE=2 SV=1 | Eukaryotic translation initiation factor 5B OS=Homo sapiens GN=Eif5b PE=1 SV=2 |
| Epb4.1l2 protein OS=Homo sapiens GN=Epb41l2 PE=2 SV=1 | Ewing sarcoma breakpoint region 1 OS=Homo sapiens GN=Ewsr1 PE=2 SV=1 |
| Epb4.1l3 protein (Fragment) OS=Homo sapiens GN=Epb41l3 PE=2 SV=1 | Fast skeletal muscle SR calcium ATPase OS=Homo sapiens GN=Atp2a1 PE=2 SV=2 |
| Epiplakin OS=Homo sapiens GN=Eppk1 PE=1 SV=2 | Fc fragment of IgG-binding protein OS=Homo sapiens GN=Fcgbp PE=1 SV=1 |
| Erythrocyte protein band 4.1-like 2 OS=Homo sapiens GN=Epb41l2 PE=2 SV=1 | FGFR2 OS=Homo sapiens GN=Fgfr2 PE=2 SV=1 |
| Erythrocyte protein band 4.1-like 3 isoform B OS=Homo sapiens GN=Epb41l3 PE=2 SV=1 | Fibroblast growth factor receptor 1 OS=Homo sapiens GN=Fgfr1 PE=1 SV=2 |
| Erythrocyte protein band 4.1-like 3 isoform C OS=Homo sapiens GN=Epb41l3 PE=2 SV=1 | Fibroblast growth factor receptor 2 OS=Homo sapiens GN=Fgfr2 PE=1 SV=1 |
| Erythrocyte protein band 4.1-like 3 OS=Homo sapiens GN=Epb41l3 PE=2 SV=1 | Fibroblast growth factor receptor 2 OS=Homo sapiens GN=Fgfr2 PE=1 SV=4 |
| Eukaryotic translation initiation factor 1A, X-chromosomal OS=Homo sapiens GN=Eif1ax PE=2 SV=3 | Fibroblast growth factor receptor 3 (Fragment) OS=Homo sapiens GN=Fgfr3 PE=1 SV=1 |
| Eukaryotic translation initiation factor 2 subunit 1 OS=Homo sapiens GN=Eif2s1 PE=1 SV=3 | Fibroblast growth factor receptor 3 OS=Homo sapiens GN=Fgfr3 PE=1 SV=1 |
| Eukaryotic translation initiation factor 2 subunit 2 (Fragment) OS=Homo sapiens GN=Eif2s2 PE=1 SV=1 | Fibroblast growth factor receptor 4 OS=Homo sapiens GN=Fgfr4 PE=1 SV=3 |
| Eukaryotic translation initiation factor 2 subunit 3, X-linked OS=Homo sapiens GN=Eif2s3x PE=1 SV=1 | Fibroblast growth factor receptor OS=Homo sapiens GN=Fgfr1 PE=1 SV=1 |
| Eukaryotic translation initiation factor 2 subunit 3, Y-linked OS=Homo sapiens GN=Eif2s3y PE=1 SV=2 | Fibroblast growth factor receptor OS=Homo sapiens GN=Fgfr1 PE=2 SV=1 |
| Eukaryotic translation initiation factor 2, subunit 3, structural gene X-linked OS=Homo sapiens GN=Eif2s3x PE=1 SV=1 | Fibroblast growth factor receptor OS=Homo sapiens GN=Fgfr2 PE=1 SV=1 |
| Eukaryotic translation initiation factor 2, subunit 3, structural gene Y-linked, isoform CRA_b OS=Homo sapiens GN=Eif2s3y PE=2 SV=1 | Fibroblast growth factor receptor OS=Homo sapiens GN=Fgfr2 PE=2 SV=1 |
| Eukaryotic translation initiation factor 5B OS=Homo sapiens GN=Eif5b PE=1 SV=2 | Fibroblast growth factor receptor OS=Homo sapiens GN=Fgfr3 PE=1 SV=1 |
| Ewing sarcoma breakpoint region 1 OS=Homo sapiens GN=Ewsr1 PE=2 SV=1 | Fibroblast growth factor receptor OS=Homo sapiens GN=Fgfr3 PE=1 SV=2 |
| Expressed sequence BB287469 OS=Homo sapiens GN=BB287469 PE=2 SV=1 | Fibroblast growth factor receptor OS=Homo sapiens GN=Fgfr3 PE=2 SV=1 |
| Ezrin OS=Homo sapiens GN=Ezr PE=1 SV=1 | Fibroblast growth factor receptor OS=Homo sapiens GN=Fgfr4 PE=3 SV=1 |
| Fc fragment of IgG-binding protein OS=Homo sapiens GN=Fcgbp PE=1 SV=1 | Fibronectin OS=Homo sapiens GN=Fn1 PE=1 SV=1 |
| FGFR2 OS=Homo sapiens GN=Fgfr2 PE=2 SV=1 | Fibronectin OS=Homo sapiens GN=Fn1 PE=1 SV=4 |
| Fibroblast growth factor receptor 1 OS=Homo sapiens GN=Fgfr1 PE=1 SV=2 | Flap endonuclease 1 OS=Homo sapiens GN=Fen1 PE=1 SV=1 |
| Fibroblast growth factor receptor 2 OS=Homo sapiens GN=Fgfr2 PE=1 SV=1 | Flap endonuclease 1 OS=Homo sapiens GN=Fen1 PE=2 SV=1 |
| Fibroblast growth factor receptor 2 OS=Homo sapiens GN=Fgfr2 PE=1 SV=4 | Flap endonuclease 1 OS=Homo sapiens GN=Fen1 PE=3 SV=1 |
| Fibroblast growth factor receptor 3 (Fragment) OS=Homo sapiens GN=Fgfr3 PE=1 SV=1 | Fragile X mental retardation protein FMRP OS=Homo sapiens GN=Fmr1 PE=2 SV=1 |
| Fibroblast growth factor receptor 3 OS=Homo sapiens GN=Fgfr3 PE=1 SV=1 | Fragile X mental retardation syndrome 1 homolog OS=Homo sapiens GN=Fmr1 PE=1 SV=1 |
| Fibroblast growth factor receptor 4 OS=Homo sapiens GN=Fgfr4 PE=1 SV=3 | Fragile X mental retardation syndrome-related protein 1 OS=Homo sapiens GN=Fxr1 PE=1 SV=1 |
| Fibroblast growth factor receptor OS=Homo sapiens GN=Fgfr1 PE=1 SV=1 | Fragile X mental retardation syndrome-related protein 1 OS=Homo sapiens GN=Fxr1 PE=1 SV=2 |
| Fibroblast growth factor receptor OS=Homo sapiens GN=Fgfr1 PE=2 SV=1 | Fragile X mental retardation syndrome-related protein 2 OS=Homo sapiens GN=Fxr2 PE=1 SV=1 |
| Fibroblast growth factor receptor OS=Homo sapiens GN=Fgfr2 PE=1 SV=1 | Fusion, derived from t(12;16) malignant liposarcoma (Human), isoform CRA_a OS=Homo sapiens GN=Fus PE=1 SV=1 |
| Fibroblast growth factor receptor OS=Homo sapiens GN=Fgfr2 PE=2 SV=1 | Fusion, derived from t(1216) malignant liposarcoma (Human) OS=Homo sapiens GN=Fus PE=1 SV=1 |
| Fibroblast growth factor receptor OS=Homo sapiens GN=Fgfr3 PE=1 SV=1 | Gag OS=Homo sapiens GN=gag PE=4 SV=2 |
| Fibroblast growth factor receptor OS=Homo sapiens GN=Fgfr3 PE=1 SV=2 | Gag polyprotein OS=Homo sapiens PE=4 SV=1 |
| Fibroblast growth factor receptor OS=Homo sapiens GN=Fgfr3 PE=2 SV=1 | Gag polyprotein pr65 OS=Homo sapiens PE=4 SV=1 |
| Fibroblast growth factor receptor OS=Homo sapiens GN=Fgfr4 PE=3 SV=1 | Gag protein OS=Homo sapiens GN=gag PE=4 SV=1 |
| Fibronectin OS=Homo sapiens GN=Fn1 PE=1 SV=1 | Gag protein OS=Homo sapiens PE=2 SV=1 |
| Fibronectin OS=Homo sapiens GN=Fn1 PE=1 SV=4 | Gag-Pol polyprotein OS=Homo sapiens GN=Mela PE=2 SV=1 |
| Flap endonuclease 1 OS=Homo sapiens GN=Fen1 PE=1 SV=1 | Gag-pro-pol polyprotein OS=Homo sapiens GN=gag-pro-pol PE=4 SV=1 |
| Flap endonuclease 1 OS=Homo sapiens GN=Fen1 PE=2 SV=1 | Gag-pro-pol polyprotein OS=Homo sapiens PE=4 SV=1 |
| Flap endonuclease 1 OS=Homo sapiens GN=Fen1 PE=3 SV=1 | Gamma actin-like protein OS=Homo sapiens GN=Actg1 PE=2 SV=1 |
| Fragile X mental retardation protein FMRP OS=Homo sapiens GN=Fmr1 PE=2 SV=1 | Gapdh protein OS=Homo sapiens GN=Gapdh PE=2 SV=1 |
| Fragile X mental retardation syndrome 1 homolog OS=Homo sapiens GN=Fmr1 PE=1 SV=1 | Gene for histone H2a (Fragment) OS=Homo sapiens PE=4 SV=1 |
| Fragile X mental retardation syndrome-related protein 1 OS=Homo sapiens GN=Fxr1 PE=1 SV=1 | Glyceraldehyde-3-phosphate dehydrogenase (Fragment) OS=Homo sapiens GN=Gapdh PE=1 SV=1 |
| Fragile X mental retardation syndrome-related protein 1 OS=Homo sapiens GN=Fxr1 PE=1 SV=2 | Glyceraldehyde-3-phosphate dehydrogenase (Fragment) OS=Homo sapiens PE=2 SV=1 |
| Fragile X mental retardation syndrome-related protein 2 OS=Homo sapiens GN=Fxr2 PE=1 SV=1 | Glyceraldehyde-3-phosphate dehydrogenase OS=Homo sapiens GN=Gapdh PE=1 SV=1 |
| Fusion, derived from t(12;16) malignant liposarcoma (Human), isoform CRA_a OS=Homo sapiens GN=Fus PE=1 SV=1 | Glyceraldehyde-3-phosphate dehydrogenase OS=Homo sapiens GN=Gapdh PE=1 SV=2 |
| Fusion, derived from t(1216) malignant liposarcoma (Human) OS=Homo sapiens GN=Fus PE=1 SV=1 | Glyceraldehyde-3-phosphate dehydrogenase OS=Homo sapiens GN=Gapdhs PE=1 SV=1 |
| Gag polyprotein OS=Homo sapiens PE=4 SV=1 | Glyceraldehyde-3-phosphate dehydrogenase OS=Homo sapiens GN=Gm10358 PE=1 SV=1 |
| Gag polyprotein pr65 OS=Homo sapiens PE=4 SV=1 | Glyceraldehyde-3-phosphate dehydrogenase OS=Homo sapiens GN=Gm3839 PE=1 SV=1 |
| Gag protein OS=Homo sapiens GN=gag PE=4 SV=1 | Glyceraldehyde-3-phosphate dehydrogenase, testis-specific OS=Homo sapiens GN=Gapdhs PE=1 SV=1 |
| Gag protein OS=Homo sapiens PE=2 SV=1 | Glyceraldehyde-3-phosphate-dehydrogenase (Fragment) OS=Homo sapiens GN=Gapd PE=2 SV=1 |
| Gag-Pol polyprotein OS=Homo sapiens GN=Mela PE=2 SV=1 | Glyco-gag polyprotein OS=Homo sapiens PE=4 SV=1 |
| Gag-pro-pol polyprotein OS=Homo sapiens GN=gag-pro-pol PE=4 SV=1 | Golgi-associated plant pathogenesis-related protein 1 OS=Homo sapiens GN=Glipr2 PE=1 SV=1 |
| Gag-pro-pol polyprotein OS=Homo sapiens PE=4 SV=1 | Golgi-associated plant pathogenesis-related protein 1 OS=Homo sapiens GN=Glipr2 PE=1 SV=3 |
| Gamma actin-like protein OS=Homo sapiens GN=Actg1 PE=2 SV=1 | GTPase activating protein (SH3 domain) binding protein 2 OS=Homo sapiens GN=G3bp2 PE=2 SV=1 |
| Gapdh protein OS=Homo sapiens GN=Gapdh PE=2 SV=1 | GTP-binding nuclear protein Ran OS=Homo sapiens GN=1700009N14Rik PE=2 SV=1 |
| Gene for histone H2a (Fragment) OS=Homo sapiens PE=4 SV=1 | GTP-binding nuclear protein Ran OS=Homo sapiens GN=Ran PE=1 SV=3 |
| Glutamyl-prolyl-tRNA synthetase OS=Homo sapiens GN=Eprs PE=2 SV=1 | GTP-binding nuclear protein Ran OS=Homo sapiens GN=Ran PE=2 SV=1 |
| Glyceraldehyde-3-phosphate dehydrogenase (Fragment) OS=Homo sapiens GN=Gapdh PE=1 SV=1 | GTP-binding nuclear protein Ran, testis-specific isoform OS=Homo sapiens GN=Rasl2-9 PE=2 SV=1 |
| Glyceraldehyde-3-phosphate dehydrogenase (Fragment) OS=Homo sapiens PE=2 SV=1 | Heat shock 70 kDa protein 1B OS=Homo sapiens GN=Hspa1b PE=1 SV=3 |
| Glyceraldehyde-3-phosphate dehydrogenase OS=Homo sapiens GN=Gapdh PE=1 SV=1 | Heat shock 70 kDa protein 1-like OS=Homo sapiens GN=Hspa1l PE=1 SV=4 |
| Glyceraldehyde-3-phosphate dehydrogenase OS=Homo sapiens GN=Gapdh PE=1 SV=2 | Heat shock cognate 71 kDa protein (Fragment) OS=Homo sapiens GN=Hspa8 PE=1 SV=1 |
| Glyceraldehyde-3-phosphate dehydrogenase OS=Homo sapiens GN=Gapdhs PE=1 SV=1 | Heat shock cognate 71 kDa protein OS=Homo sapiens GN=Hspa8 PE=1 SV=1 |
| Glyceraldehyde-3-phosphate dehydrogenase OS=Homo sapiens GN=Gm10358 PE=1 SV=1 | Heat shock cognate hsc73 (Fragment) OS=Homo sapiens GN=Hspa8 PE=2 SV=1 |
| Glyceraldehyde-3-phosphate dehydrogenase OS=Homo sapiens GN=Gm3839 PE=1 SV=1 | Heat shock protein 1B OS=Homo sapiens GN=Hspa1b PE=1 SV=1 |
| Glyceraldehyde-3-phosphate dehydrogenase, testis-specific OS=Homo sapiens GN=Gapdhs PE=1 SV=1 | Heat shock protein 1-like protein OS=Homo sapiens PE=3 SV=1 |
| Glyceraldehyde-3-phosphate-dehydrogenase (Fragment) OS=Homo sapiens GN=Gapd PE=2 SV=1 | Heat shock protein 70 (Fragment) OS=Homo sapiens GN=Hsp70.3 PE=3 SV=1 |
| Glyco-gag polyprotein OS=Homo sapiens PE=4 SV=1 | Heat shock protein 70-2 OS=Homo sapiens PE=3 SV=1 |
| Golgi-associated plant pathogenesis-related protein 1 OS=Homo sapiens GN=Glipr2 PE=1 SV=1 | Heat shock-related 70 kDa protein 2 OS=Homo sapiens GN=Hspa2 PE=1 SV=2 |
| Golgi-associated plant pathogenesis-related protein 1 OS=Homo sapiens GN=Glipr2 PE=1 SV=3 | Heterogeneous nuclear ribonucleoprotein A/B OS=Homo sapiens GN=Hnrnpab PE=1 SV=1 |
| GTPase activating protein (SH3 domain) binding protein 2 OS=Homo sapiens GN=G3bp2 PE=2 SV=1 | Heterogeneous nuclear ribonucleoprotein A/B, isoform CRA_b OS=Homo sapiens GN=Hnrnpab PE=2 SV=1 |
| GTP-binding nuclear protein Ran OS=Homo sapiens GN=1700009N14Rik PE=2 SV=1 | Heterogeneous nuclear ribonucleoprotein A0 OS=Homo sapiens GN=Hnrnpa0 PE=1 SV=1 |
| GTP-binding nuclear protein Ran OS=Homo sapiens GN=Ran PE=1 SV=3 | Heterogeneous nuclear ribonucleoprotein A1 OS=Homo sapiens GN=Hnrnpa1 PE=1 SV=1 |
| GTP-binding nuclear protein Ran OS=Homo sapiens GN=Ran PE=2 SV=1 | Heterogeneous nuclear ribonucleoprotein A1 OS=Homo sapiens GN=Hnrnpa1 PE=1 SV=2 |
| GTP-binding nuclear protein Ran, testis-specific isoform OS=Homo sapiens GN=Rasl2-9 PE=2 SV=1 | Heterogeneous nuclear ribonucleoprotein A2/B1 OS=Homo sapiens GN=Hnrnpa2b1 PE=2 SV=1 |
| Heat shock 70 kDa protein 1B OS=Homo sapiens GN=Hspa1b PE=1 SV=3 | Heterogeneous nuclear ribonucleoprotein A3 (Fragment) OS=Homo sapiens GN=Hnrnpa3 PE=1 SV=1 |
| Heat shock 70 kDa protein 1-like OS=Homo sapiens GN=Hspa1l PE=1 SV=4 | Heterogeneous nuclear ribonucleoprotein A3 OS=Homo sapiens GN=Hnrnpa3 PE=1 SV=1 |
| Heat shock cognate 71 kDa protein (Fragment) OS=Homo sapiens GN=Hspa8 PE=1 SV=1 | Heterogeneous nuclear ribonucleoprotein A3 OS=Homo sapiens GN=Hnrnpa3 PE=2 SV=1 |
| Heat shock cognate 71 kDa protein OS=Homo sapiens GN=Hspa8 PE=1 SV=1 | Heterogeneous nuclear ribonucleoprotein D, isoform CRA_a OS=Homo sapiens GN=Hnrnpd PE=1 SV=1 |
| Heat shock cognate hsc73 (Fragment) OS=Homo sapiens GN=Hspa8 PE=2 SV=1 | Heterogeneous nuclear ribonucleoprotein D, isoform CRA_b OS=Homo sapiens GN=Hnrnpd PE=1 SV=1 |
| Heat shock protein 1B OS=Homo sapiens GN=Hspa1b PE=1 SV=1 | Heterogeneous nuclear ribonucleoprotein D0 (Fragment) OS=Homo sapiens GN=Hnrnpd PE=1 SV=1 |
| Heat shock protein 1-like protein OS=Homo sapiens PE=3 SV=1 | Heterogeneous nuclear ribonucleoprotein D0 OS=Homo sapiens GN=Hnrnpd PE=1 SV=2 |
| Heat shock protein 70 (Fragment) OS=Homo sapiens GN=Hsp70.3 PE=3 SV=1 | Heterogeneous nuclear ribonucleoprotein D-like (Fragment) OS=Homo sapiens GN=Hnrnpdl PE=1 SV=1 |
| Heat shock protein 70-2 OS=Homo sapiens PE=3 SV=1 | Heterogeneous nuclear ribonucleoprotein D-like OS=Homo sapiens GN=Hnrnpdl PE=1 SV=1 |
| Heat shock-related 70 kDa protein 2 OS=Homo sapiens GN=Hspa2 PE=1 SV=2 | Heterogeneous nuclear ribonucleoprotein F OS=Homo sapiens GN=Hnrnpf PE=1 SV=3 |
| Heterogeneous nuclear ribonucleoprotein A1 OS=Homo sapiens GN=Hnrnpa1 PE=1 SV=1 | Heterogeneous nuclear ribonucleoprotein H OS=Homo sapiens GN=Hnrnph1 PE=1 SV=1 |
| Heterogeneous nuclear ribonucleoprotein A1 OS=Homo sapiens GN=Hnrnpa1 PE=1 SV=2 | Heterogeneous nuclear ribonucleoprotein H1 OS=Homo sapiens GN=Hnrnph1 PE=1 SV=2 |
| Heterogeneous nuclear ribonucleoprotein A2/B1 OS=Homo sapiens GN=Hnrnpa2b1 PE=2 SV=1 | Heterogeneous nuclear ribonucleoprotein H2 OS=Homo sapiens GN=Hnrnph2 PE=1 SV=1 |
| Heterogeneous nuclear ribonucleoprotein F OS=Homo sapiens GN=Hnrnpf PE=1 SV=3 | Heterogeneous nuclear ribonucleoprotein K (Fragment) OS=Homo sapiens GN=Hnrnpk PE=1 SV=1 |
| Heterogeneous nuclear ribonucleoprotein H OS=Homo sapiens GN=Hnrnph1 PE=1 SV=1 | Heterogeneous nuclear ribonucleoprotein K (Fragment) OS=Homo sapiens GN=Hnrnpk PE=1 SV=8 |
| Heterogeneous nuclear ribonucleoprotein H1 OS=Homo sapiens GN=Hnrnph1 PE=1 SV=2 | Heterogeneous nuclear ribonucleoprotein K OS=Homo sapiens GN=Hnrnpk PE=1 SV=1 |
| Heterogeneous nuclear ribonucleoprotein H2 OS=Homo sapiens GN=Hnrnph2 PE=1 SV=1 | Heterogeneous nuclear ribonucleoprotein L (Fragment) OS=Homo sapiens GN=Hnrnpl PE=1 SV=1 |
| Heterogeneous nuclear ribonucleoprotein K (Fragment) OS=Homo sapiens GN=Hnrnpk PE=1 SV=1 | Heterogeneous nuclear ribonucleoprotein L OS=Homo sapiens GN=Hnrnpl PE=1 SV=1 |
| Heterogeneous nuclear ribonucleoprotein K (Fragment) OS=Homo sapiens GN=Hnrnpk PE=1 SV=8 | Heterogeneous nuclear ribonucleoprotein L OS=Homo sapiens GN=Hnrnpl PE=1 SV=2 |
| Heterogeneous nuclear ribonucleoprotein K OS=Homo sapiens GN=Hnrnpk PE=1 SV=1 | Heterogeneous nuclear ribonucleoprotein L-like OS=Homo sapiens GN=Hnrnpll PE=1 SV=1 |
| Heterogeneous nuclear ribonucleoprotein M OS=Homo sapiens GN=Hnrnpm PE=1 SV=1 | Heterogeneous nuclear ribonucleoprotein L-like OS=Homo sapiens GN=Hnrnpll PE=1 SV=3 |
| Heterogeneous nuclear ribonucleoprotein M OS=Homo sapiens GN=Hnrnpm PE=1 SV=3 | Heterogeneous nuclear ribonucleoprotein M OS=Homo sapiens GN=Hnrnpm PE=1 SV=1 |
| Heterogeneous nuclear ribonucleoprotein Q (Fragment) OS=Homo sapiens GN=Syncrip PE=1 SV=1 | Heterogeneous nuclear ribonucleoprotein M OS=Homo sapiens GN=Hnrnpm PE=1 SV=3 |
| Heterogeneous nuclear ribonucleoprotein Q OS=Homo sapiens GN=Syncrip PE=1 SV=1 | Heterogeneous nuclear ribonucleoprotein Q (Fragment) OS=Homo sapiens GN=Syncrip PE=1 SV=1 |
| Heterogeneous nuclear ribonucleoprotein Q OS=Homo sapiens GN=Syncrip PE=1 SV=2 | Heterogeneous nuclear ribonucleoprotein Q OS=Homo sapiens GN=Syncrip PE=1 SV=1 |
| Heterogeneous nuclear ribonucleoprotein U OS=Homo sapiens GN=Hnrnpu PE=1 SV=1 | Heterogeneous nuclear ribonucleoprotein Q OS=Homo sapiens GN=Syncrip PE=1 SV=2 |
| Heterogeneous nuclear ribonucleoproteins A2/B1 OS=Homo sapiens GN=Hnrnpa2b1 PE=1 SV=2 | Heterogeneous nuclear ribonucleoprotein R (Fragment) OS=Homo sapiens GN=Hnrnpr PE=1 SV=1 |
| Heterogenous nuclear ribonucleoprotein U OS=Homo sapiens GN=Hnrnpu PE=2 SV=1 | Heterogeneous nuclear ribonucleoprotein R OS=Homo sapiens GN=Hnrnpr PE=1 SV=1 |
| High mobility group protein B2 OS=Homo sapiens GN=Hmgb2 PE=1 SV=3 | Heterogeneous nuclear ribonucleoprotein U OS=Homo sapiens GN=Hnrnpu PE=1 SV=1 |
| High mobility group protein HMG-I/HMG-Y OS=Homo sapiens GN=Hmga1 PE=1 SV=4 | Heterogeneous nuclear ribonucleoproteins A2/B1 (Fragment) OS=Homo sapiens GN=Hnrnpa2b1 PE=1 SV=1 |
| Hist1h1b protein (Fragment) OS=Homo sapiens GN=Hist1h1b PE=2 SV=1 | Heterogeneous nuclear ribonucleoproteins A2/B1 OS=Homo sapiens GN=Hnrnpa2b1 PE=1 SV=2 |
| Hist2h2bb protein OS=Homo sapiens GN=Hist2h2bb PE=2 SV=1 | Heterogenous nuclear ribonucleoprotein U OS=Homo sapiens GN=Hnrnpu PE=2 SV=1 |
| Histone cluster 1, H1c OS=Homo sapiens GN=Hist1h1c PE=1 SV=1 | High mobility group box 1 OS=Homo sapiens GN=Hmgb1 PE=1 SV=1 |
| Histone cluster 1, H1d OS=Homo sapiens GN=Hist1h1d PE=1 SV=1 | High mobility group box 1 OS=Homo sapiens GN=Hmgb1 PE=2 SV=1 |
| Histone H1.1 OS=Homo sapiens GN=Hist1h1a PE=1 SV=2 | High mobility group protein B1 (Fragment) OS=Homo sapiens GN=Hmgb1 PE=1 SV=1 |
| Histone H1.4 OS=Homo sapiens GN=Hist1h1e PE=1 SV=2 | High mobility group protein B1 OS=Homo sapiens GN=Hmgb1 PE=1 SV=1 |
| Histone H1.5 OS=Homo sapiens GN=Hist1h1b PE=1 SV=2 | High mobility group protein B2 OS=Homo sapiens GN=Hmgb2 PE=1 SV=3 |
| Histone H1t OS=Homo sapiens GN=Hist1h1t PE=1 SV=1 | High mobility group protein HMGI-C OS=Homo sapiens GN=Hmga2 PE=1 SV=1 |
| Histone H1t OS=Homo sapiens GN=Hist1h1t PE=1 SV=4 | Hist1h1b protein (Fragment) OS=Homo sapiens GN=Hist1h1b PE=2 SV=1 |
| Histone H2A (Fragment) OS=Homo sapiens GN=Hist1h2ah PE=2 SV=1 | Hist2h2bb protein OS=Homo sapiens GN=Hist2h2bb PE=2 SV=1 |
| Histone H2A OS=Homo sapiens GN=H2afj PE=1 SV=1 | Histone cluster 1, H1c OS=Homo sapiens GN=Hist1h1c PE=1 SV=1 |
| Histone H2A OS=Homo sapiens GN=H2afv PE=2 SV=1 | Histone cluster 1, H1d OS=Homo sapiens GN=Hist1h1d PE=1 SV=1 |
| Histone H2A OS=Homo sapiens GN=H2afz PE=1 SV=1 | Histone H1.1 OS=Homo sapiens GN=Hist1h1a PE=1 SV=2 |
| Histone H2A OS=Homo sapiens GN=H2afz PE=2 SV=1 | Histone H1.4 OS=Homo sapiens GN=Hist1h1e PE=1 SV=2 |
| Histone H2A OS=Homo sapiens GN=Hist1h2aa PE=1 SV=1 | Histone H1.5 OS=Homo sapiens GN=Hist1h1b PE=1 SV=2 |
| Histone H2A OS=Homo sapiens GN=Hist1h2ah PE=2 SV=1 | Histone H1t OS=Homo sapiens GN=Hist1h1t PE=1 SV=1 |
| Histone H2A OS=Homo sapiens GN=Hist2h2aa1 PE=2 SV=1 | Histone H1t OS=Homo sapiens GN=Hist1h1t PE=1 SV=4 |
| Histone H2A type 1-F OS=Homo sapiens GN=Hist1h2af PE=1 SV=3 | Histone H2A (Fragment) OS=Homo sapiens GN=Hist1h2ah PE=2 SV=1 |
| Histone H2A type 1-I OS=Homo sapiens GN=Hist1h2ai PE=1 SV=1 | Histone H2A OS=Homo sapiens GN=H2afj PE=1 SV=1 |
| Histone H2A type 1-K OS=Homo sapiens GN=Hist1h2ak PE=1 SV=3 | Histone H2A OS=Homo sapiens GN=H2afv PE=2 SV=1 |
| Histone H2A type 2-B OS=Homo sapiens GN=Hist2h2ab PE=1 SV=3 | Histone H2A OS=Homo sapiens GN=H2afz PE=1 SV=1 |
| Histone H2A type 2-C OS=Homo sapiens GN=Hist2h2ac PE=1 SV=3 | Histone H2A OS=Homo sapiens GN=H2afz PE=2 SV=1 |
| Histone H2A type 3 OS=Homo sapiens GN=Hist3h2a PE=1 SV=3 | Histone H2A OS=Homo sapiens GN=Hist1h2aa PE=1 SV=1 |
| Histone H2A.J OS=Homo sapiens GN=H2afj PE=1 SV=1 | Histone H2A OS=Homo sapiens GN=Hist1h2ah PE=2 SV=1 |
| Histone H2A.Z OS=Homo sapiens GN=H2afz PE=1 SV=2 | Histone H2A OS=Homo sapiens GN=Hist2h2aa1 PE=2 SV=1 |
| Histone H2AX OS=Homo sapiens GN=H2afx PE=1 SV=2 | Histone H2A type 1-F OS=Homo sapiens GN=Hist1h2af PE=1 SV=3 |
| Histone H2B (Fragment) OS=Homo sapiens GN=Hist1h2bj PE=2 SV=1 | Histone H2A type 1-I OS=Homo sapiens GN=Hist1h2ai PE=1 SV=1 |
| Histone H2B OS=Homo sapiens GN=Hist1h2ba PE=2 SV=1 | Histone H2A type 1-K OS=Homo sapiens GN=Hist1h2ak PE=1 SV=3 |
| Histone H2B OS=Homo sapiens GN=Hist1h2bk PE=2 SV=1 | Histone H2A type 2-B OS=Homo sapiens GN=Hist2h2ab PE=1 SV=3 |
| Histone H2B OS=Homo sapiens GN=Hist1h2bm PE=1 SV=1 | Histone H2A type 2-C OS=Homo sapiens GN=Hist2h2ac PE=1 SV=3 |
| Histone H2B OS=Homo sapiens GN=Hist1h2bq PE=2 SV=1 | Histone H2A type 3 OS=Homo sapiens GN=Hist3h2a PE=1 SV=3 |
| Histone H2B OS=Homo sapiens GN=LOC665622 PE=2 SV=1 | Histone H2A.J OS=Homo sapiens GN=H2afj PE=1 SV=1 |
| Histone H2B type 1-A OS=Homo sapiens GN=Hist1h2ba PE=1 SV=3 | Histone H2A.Z OS=Homo sapiens GN=H2afz PE=1 SV=2 |
| Histone H2B type 1-B OS=Homo sapiens GN=Hist1h2bb PE=1 SV=3 | Histone H2AX OS=Homo sapiens GN=H2afx PE=1 SV=2 |
| Histone H2B type 1-C/E/G OS=Homo sapiens GN=Hist1h2bc PE=1 SV=3 | Histone H2B (Fragment) OS=Homo sapiens GN=Hist1h2bj PE=2 SV=1 |
| Histone H2B type 1-F/J/L OS=Homo sapiens GN=Hist1h2bf PE=1 SV=2 | Histone H2B OS=Homo sapiens GN=Hist1h2ba PE=2 SV=1 |
| Histone H2B type 1-H OS=Homo sapiens GN=Hist1h2bh PE=1 SV=3 | Histone H2B OS=Homo sapiens GN=Hist1h2bk PE=2 SV=1 |
| Histone H2B type 1-P OS=Homo sapiens GN=Hist1h2bp PE=1 SV=3 | Histone H2B OS=Homo sapiens GN=Hist1h2bm PE=1 SV=1 |
| Histone H2B type 2-B OS=Homo sapiens GN=Hist2h2bb PE=1 SV=3 | Histone H2B OS=Homo sapiens GN=Hist1h2bq PE=2 SV=1 |
| Histone H2B type 2-E OS=Homo sapiens GN=Hist2h2be PE=1 SV=3 | Histone H2B OS=Homo sapiens GN=LOC665622 PE=2 SV=1 |
| Histone H2B type 3-A OS=Homo sapiens GN=Hist3h2ba PE=1 SV=3 | Histone H2B type 1-A OS=Homo sapiens GN=Hist1h2ba PE=1 SV=3 |
| Histone H2B type 3-B OS=Homo sapiens GN=Hist3h2bb PE=1 SV=3 | Histone H2B type 1-B OS=Homo sapiens GN=Hist1h2bb PE=1 SV=3 |
| Histone H3 (Fragment) OS=Homo sapiens GN=H3f3a PE=3 SV=1 | Histone H2B type 1-C/E/G OS=Homo sapiens GN=Hist1h2bc PE=1 SV=3 |
| Histone H3 (Fragment) OS=Homo sapiens GN=Hist1h3e PE=2 SV=1 | Histone H2B type 1-F/J/L OS=Homo sapiens GN=Hist1h2bf PE=1 SV=2 |
| Histone H3 (Fragment) OS=Homo sapiens GN=Hist1h3i PE=2 SV=1 | Histone H2B type 1-H OS=Homo sapiens GN=Hist1h2bh PE=1 SV=3 |
| Histone H3 OS=Homo sapiens GN=H3f3a PE=1 SV=1 | Histone H2B type 1-P OS=Homo sapiens GN=Hist1h2bp PE=1 SV=3 |
| Histone H3.1 OS=Homo sapiens GN=Hist1h3a PE=1 SV=2 | Histone H2B type 2-B OS=Homo sapiens GN=Hist2h2bb PE=1 SV=3 |
| Histone H3.2 OS=Homo sapiens GN=Hist1h3b PE=1 SV=2 | Histone H2B type 2-E OS=Homo sapiens GN=Hist2h2be PE=1 SV=3 |
| Histone H3.2 OS=Homo sapiens GN=Hist2h3c1 PE=1 SV=1 | Histone H2B type 3-A OS=Homo sapiens GN=Hist3h2ba PE=1 SV=3 |
| Histone H3.3 OS=Homo sapiens GN=H3f3a PE=1 SV=2 | Histone H2B type 3-B OS=Homo sapiens GN=Hist3h2bb PE=1 SV=3 |
| Histone H3.3C OS=Homo sapiens GN=H3f3c PE=3 SV=3 | Histone H3 (Fragment) OS=Homo sapiens GN=H3f3a PE=3 SV=1 |
| Histone H4 (Fragment) OS=Homo sapiens PE=3 SV=1 | Histone H3 (Fragment) OS=Homo sapiens GN=Hist1h3e PE=2 SV=1 |
| Histone H4 OS=Homo sapiens GN=Hist2h4 PE=1 SV=1 | Histone H3 (Fragment) OS=Homo sapiens GN=Hist1h3i PE=2 SV=1 |
| Hnrpf protein (Fragment) OS=Homo sapiens GN=Hnrnpf PE=2 SV=1 | Histone H3 OS=Homo sapiens GN=H3f3a PE=1 SV=1 |
| Hnrpk protein OS=Homo sapiens GN=Hnrnpk PE=2 SV=1 | Histone H3.1 OS=Homo sapiens GN=Hist1h3a PE=1 SV=2 |
| Hsc70t (Fragment) OS=Homo sapiens PE=3 SV=1 | Histone H3.2 OS=Homo sapiens GN=Hist1h3b PE=1 SV=2 |
| Hspa8 protein (Fragment) OS=Homo sapiens GN=Hspa8 PE=2 SV=1 | Histone H3.2 OS=Homo sapiens GN=Hist2h3c1 PE=1 SV=1 |
| IFN-response element binding factor 2 (Fragment) OS=Homo sapiens GN=Srsf2 PE=2 SV=1 | Histone H3.3 OS=Homo sapiens GN=H3f3a PE=1 SV=2 |
| Importin subunit alpha OS=Homo sapiens GN=Kpna2 PE=1 SV=1 | Histone H3.3C OS=Homo sapiens GN=H3f3c PE=3 SV=3 |
| Importin subunit alpha OS=Homo sapiens GN=Kpna2 PE=2 SV=1 | Histone H4 (Fragment) OS=Homo sapiens PE=3 SV=1 |
| Inducible heat shock protein 70 OS=Homo sapiens PE=3 SV=1 | Histone H4 OS=Homo sapiens GN=Hist2h4 PE=1 SV=1 |
| IQ motif containing GTPase activating protein 1 OS=Homo sapiens GN=Iqgap1 PE=2 SV=1 | Hmgb1 protein OS=Homo sapiens PE=2 SV=1 |
| Iqgap1 protein (Fragment) OS=Homo sapiens GN=Iqgap1 PE=2 SV=1 | Hnrnpa1l2 protein OS=Homo sapiens GN=Hnrnpa1l2 PE=2 SV=1 |
| Keratin 15, isoform CRA_a OS=Homo sapiens GN=Krt15 PE=1 SV=1 | Hnrpa3 protein (Fragment) OS=Homo sapiens GN=Hnrnpa3 PE=2 SV=1 |
| Keratin 16 OS=Homo sapiens GN=Krt16 PE=1 SV=1 | Hnrpa3 protein OS=Homo sapiens GN=Hnrnpa3 PE=2 SV=1 |
| Keratin 16 OS=Homo sapiens GN=Krt16 PE=2 SV=1 | Hnrpf protein (Fragment) OS=Homo sapiens GN=Hnrnpf PE=2 SV=1 |
| Keratin 1b (Fragment) OS=Homo sapiens GN=Krt77 PE=2 SV=1 | Hnrpk protein OS=Homo sapiens GN=Hnrnpk PE=2 SV=1 |
| Keratin 24 variant 2 OS=Homo sapiens GN=Krt24 PE=2 SV=1 | Hnrpr protein (Fragment) OS=Homo sapiens GN=Hnrnpr PE=2 SV=1 |
| Keratin 5 OS=Homo sapiens GN=Krt5 PE=1 SV=2 | Hsc70t (Fragment) OS=Homo sapiens PE=3 SV=1 |
| Keratin 77 OS=Homo sapiens GN=Krt77 PE=2 SV=1 | Hspa8 protein (Fragment) OS=Homo sapiens GN=Hspa8 PE=2 SV=1 |
| Keratin 78 OS=Homo sapiens GN=Krt78 PE=1 SV=1 | IFN-response element binding factor 2 (Fragment) OS=Homo sapiens GN=Srsf2 PE=2 SV=1 |
| Keratin intermediate filament 16a OS=Homo sapiens GN=K16 PE=3 SV=1 | Importin subunit alpha OS=Homo sapiens GN=Kpna2 PE=1 SV=1 |
| Keratin intermediate filament 16b OS=Homo sapiens GN=K16 PE=3 SV=1 | Importin subunit alpha OS=Homo sapiens GN=Kpna2 PE=2 SV=1 |
| Keratin Kb40 OS=Homo sapiens GN=Krt78 PE=2 SV=1 | Inducible heat shock protein 70 OS=Homo sapiens PE=3 SV=1 |
| Keratin, type I cuticular Ha1 OS=Homo sapiens GN=Krt31 PE=1 SV=2 | Insulin-like growth factor 2 mRNA-binding protein 2 OS=Homo sapiens GN=Igf2bp2 PE=1 SV=1 |
| Keratin, type I cuticular Ha2 OS=Homo sapiens GN=Krt32 PE=1 SV=1 | Insulin-like growth factor 2 mRNA-binding protein 3 OS=Homo sapiens GN=Igf2bp3 PE=1 SV=1 |
| Keratin, type I cuticular Ha2 OS=Homo sapiens GN=Krt32 PE=1 SV=2 | Integrin beta OS=Homo sapiens GN=Itgb4 PE=1 SV=1 |
| Keratin, type I cuticular Ha3-II OS=Homo sapiens GN=Krt33b PE=1 SV=2 | Integrin beta-4 OS=Homo sapiens GN=Itgb4 PE=1 SV=1 |
| Keratin, type I cuticular Ha5 OS=Homo sapiens GN=Krt35 PE=1 SV=1 | Interferon-activable protein 204 OS=Homo sapiens GN=Ifi204 PE=1 SV=1 |
| Keratin, type I cuticular Ha6 OS=Homo sapiens GN=Krt36 PE=1 SV=1 | IQ and AAA domain-containing protein 1-like OS=Homo sapiens GN=Iqca1l PE=1 SV=3 |
| Keratin, type I cytoskeletal 10 OS=Homo sapiens GN=Krt10 PE=1 SV=1 | IQ motif containing GTPase activating protein 1 OS=Homo sapiens GN=Iqgap1 PE=2 SV=1 |
| Keratin, type I cytoskeletal 10 OS=Homo sapiens GN=Krt10 PE=1 SV=3 | Iqgap1 protein (Fragment) OS=Homo sapiens GN=Iqgap1 PE=2 SV=1 |
| Keratin, type I cytoskeletal 13 OS=Homo sapiens GN=Krt13 PE=1 SV=2 | Janus kinase and microtubule-interacting protein 1 OS=Homo sapiens GN=Jakmip1 PE=1 SV=1 |
| Keratin, type I cytoskeletal 14 OS=Homo sapiens GN=Krt14 PE=1 SV=2 | Keratin 15, isoform CRA_a OS=Homo sapiens GN=Krt15 PE=1 SV=1 |
| Keratin, type I cytoskeletal 15 OS=Homo sapiens GN=Krt15 PE=1 SV=2 | Keratin 1b (Fragment) OS=Homo sapiens GN=Krt77 PE=2 SV=1 |
| Keratin, type I cytoskeletal 17 OS=Homo sapiens GN=Krt17 PE=1 SV=3 | Keratin 5 OS=Homo sapiens GN=Krt5 PE=1 SV=2 |
| Keratin, type I cytoskeletal 18 OS=Homo sapiens GN=Krt18 PE=1 SV=5 | Keratin 77 OS=Homo sapiens GN=Krt77 PE=2 SV=1 |
| Keratin, type I cytoskeletal 19 OS=Homo sapiens GN=Krt19 PE=1 SV=1 | Keratin 78 OS=Homo sapiens GN=Krt78 PE=1 SV=1 |
| Keratin, type I cytoskeletal 24 OS=Homo sapiens GN=Krt24 PE=2 SV=2 | Keratin Kb40 OS=Homo sapiens GN=Krt78 PE=2 SV=1 |
| Keratin, type I cytoskeletal 28 OS=Homo sapiens GN=Krt28 PE=1 SV=1 | Keratin, type I cytoskeletal 10 OS=Homo sapiens GN=Krt10 PE=1 SV=1 |
| Keratin, type I cytoskeletal 40 OS=Homo sapiens GN=Krt40 PE=2 SV=1 | Keratin, type I cytoskeletal 10 OS=Homo sapiens GN=Krt10 PE=1 SV=3 |
| Keratin, type I cytoskeletal 42 OS=Homo sapiens GN=Krt42 PE=1 SV=1 | Keratin, type I cytoskeletal 14 OS=Homo sapiens GN=Krt14 PE=1 SV=2 |
| Keratin, type II cytoskeletal 1 OS=Homo sapiens GN=Krt1 PE=1 SV=4 | Keratin, type I cytoskeletal 15 OS=Homo sapiens GN=Krt15 PE=1 SV=2 |
| Keratin, type II cytoskeletal 1b OS=Homo sapiens GN=Krt77 PE=1 SV=1 | Keratin, type I cytoskeletal 17 OS=Homo sapiens GN=Krt17 PE=1 SV=3 |
| Keratin, type II cytoskeletal 2 oral OS=Homo sapiens GN=Krt76 PE=1 SV=1 | Keratin, type I cytoskeletal 19 OS=Homo sapiens GN=Krt19 PE=1 SV=1 |
| Keratin, type II cytoskeletal 6A OS=Homo sapiens GN=Krt6a PE=1 SV=3 | Keratin, type I cytoskeletal 42 OS=Homo sapiens GN=Krt42 PE=1 SV=1 |
| Keratin, type II cytoskeletal 6B OS=Homo sapiens GN=Krt6b PE=1 SV=1 | Keratin, type II cytoskeletal 1 OS=Homo sapiens GN=Krt1 PE=1 SV=4 |
| Keratin, type II cytoskeletal 6B OS=Homo sapiens GN=Krt6b PE=1 SV=3 | Keratin, type II cytoskeletal 1b OS=Homo sapiens GN=Krt77 PE=1 SV=1 |
| Keratin, type II cytoskeletal 7 OS=Homo sapiens GN=Krt7 PE=1 SV=1 | Keratin, type II cytoskeletal 2 epidermal OS=Homo sapiens GN=Krt2 PE=1 SV=1 |
| Keratin, type II cytoskeletal 75 OS=Homo sapiens GN=Krt75 PE=1 SV=1 | Keratin, type II cytoskeletal 6A OS=Homo sapiens GN=Krt6a PE=1 SV=3 |
| Keratin, type II cytoskeletal 79 OS=Homo sapiens GN=Krt79 PE=1 SV=2 | Keratin, type II cytoskeletal 6B OS=Homo sapiens GN=Krt6b PE=1 SV=1 |
| Keratin, type II cytoskeletal 8 OS=Homo sapiens GN=Krt8 PE=1 SV=4 | Keratin, type II cytoskeletal 6B OS=Homo sapiens GN=Krt6b PE=1 SV=3 |
| Krt6b protein (Fragment) OS=Homo sapiens GN=Krt6b PE=2 SV=1 | Keratin, type II cytoskeletal 7 OS=Homo sapiens GN=Krt7 PE=1 SV=1 |
| Krt6b protein OS=Homo sapiens GN=Krt6b PE=2 SV=1 | Keratin, type II cytoskeletal 71 OS=Homo sapiens GN=Krt71 PE=1 SV=1 |
| Krt78 protein (Fragment) OS=Homo sapiens GN=Krt78 PE=2 SV=1 | Keratin, type II cytoskeletal 73 OS=Homo sapiens GN=Krt73 PE=1 SV=1 |
| Lamina-associated polypeptide 2, isoforms alpha/zeta OS=Homo sapiens GN=Tmpo PE=1 SV=4 | Keratin, type II cytoskeletal 74 OS=Homo sapiens GN=Krt74 PE=3 SV=1 |
| Lamina-associated polypeptide 2, isoforms beta/delta/epsilon/gamma OS=Homo sapiens GN=Tmpo PE=1 SV=4 | Keratin, type II cytoskeletal 75 OS=Homo sapiens GN=Krt75 PE=1 SV=1 |
| Lamin-B receptor (Fragment) OS=Homo sapiens GN=Lbr PE=1 SV=1 | Keratin, type II cytoskeletal 79 OS=Homo sapiens GN=Krt79 PE=1 SV=2 |
| Lamin-B receptor OS=Homo sapiens GN=Lbr PE=1 SV=2 | Keratin, type II cytoskeletal 8 OS=Homo sapiens GN=Krt8 PE=1 SV=4 |
| Laminin receptor (Fragment) OS=Homo sapiens GN=Rpsa PE=2 SV=1 | Krt2 protein OS=Homo sapiens GN=Krt2 PE=2 SV=1 |
| La-related protein 4 OS=Homo sapiens GN=Larp4 PE=1 SV=1 | Krt6b protein (Fragment) OS=Homo sapiens GN=Krt6b PE=2 SV=1 |
| La-related protein 4 OS=Homo sapiens GN=Larp4 PE=1 SV=2 | Krt6b protein OS=Homo sapiens GN=Krt6b PE=2 SV=1 |
| Leucine-rich repeat-containing protein 59 OS=Homo sapiens GN=Lrrc59 PE=1 SV=1 | Krt78 protein (Fragment) OS=Homo sapiens GN=Krt78 PE=2 SV=1 |
| Lingerer protein-2a OS=Homo sapiens GN=lig-2a PE=2 SV=1 | Lamina-associated polypeptide 2, isoforms alpha/zeta OS=Homo sapiens GN=Tmpo PE=1 SV=4 |
| Lingerer protein-2b OS=Homo sapiens GN=Ubap2l PE=2 SV=1 | Lamina-associated polypeptide 2, isoforms beta/delta/epsilon/gamma OS=Homo sapiens GN=Tmpo PE=1 SV=4 |
| Luc7-like protein 3 OS=Homo sapiens GN=Luc7l3 PE=1 SV=1 | Laminin receptor (Fragment) OS=Homo sapiens GN=Rpsa PE=2 SV=1 |
| Mannosyl-oligosaccharide glucosidase OS=Homo sapiens GN=Mogs PE=1 SV=1 | Leucine-rich repeat-containing protein 59 OS=Homo sapiens GN=Lrrc59 PE=1 SV=1 |
| MCG10168 OS=Homo sapiens GN=Rplp1 PE=1 SV=1 | Lingerer protein-2a OS=Homo sapiens GN=lig-2a PE=2 SV=1 |
| MCG10266, isoform CRA_a OS=Homo sapiens GN=Rpl9 PE=1 SV=1 | Lingerer protein-2b OS=Homo sapiens GN=Ubap2l PE=2 SV=1 |
| MCG10725, isoform CRA_a OS=Homo sapiens GN=Rps25 PE=1 SV=1 | Luc7 homolog (S. cerevisiae)-like, isoform CRA_c OS=Homo sapiens GN=Luc7l PE=1 SV=1 |
| MCG10806 OS=Homo sapiens GN=Rpl23a PE=1 SV=1 | Luc7l protein (Fragment) OS=Homo sapiens GN=Luc7l PE=2 SV=1 |
| MCG116671 OS=Homo sapiens GN=Gm11361 PE=3 SV=1 | Luc7l2 protein OS=Homo sapiens GN=Luc7l2 PE=1 SV=1 |
| MCG118780 OS=Homo sapiens GN=Gm5788 PE=3 SV=1 | Luc7-like protein 3 OS=Homo sapiens GN=Luc7l3 PE=1 SV=1 |
| MCG12304 OS=Homo sapiens GN=Rpl22 PE=1 SV=1 | Lupus La protein homolog (Fragment) OS=Homo sapiens GN=Ssb PE=1 SV=1 |
| MCG123152 OS=Homo sapiens GN=Gm10269 PE=1 SV=1 | Lupus La protein homolog OS=Homo sapiens GN=Ssb PE=1 SV=1 |
| MCG124046 OS=Homo sapiens GN=Prss1 PE=1 SV=1 | Mannosyl-oligosaccharide glucosidase OS=Homo sapiens GN=Mogs PE=1 SV=1 |
| MCG126194, isoform CRA_a OS=Homo sapiens GN=Rpl31 PE=1 SV=1 | MCG10168 OS=Homo sapiens GN=Rplp1 PE=1 SV=1 |
| MCG132477, isoform CRA_a OS=Homo sapiens GN=Rpl18 PE=1 SV=1 | MCG10266, isoform CRA_a OS=Homo sapiens GN=Rpl9 PE=1 SV=1 |
| MCG13441 OS=Homo sapiens GN=Rps27a PE=2 SV=1 | MCG10725, isoform CRA_a OS=Homo sapiens GN=Rps25 PE=1 SV=1 |
| MCG140437, isoform CRA_d OS=Homo sapiens GN=Myh2 PE=1 SV=1 | MCG10806 OS=Homo sapiens GN=Rpl23a PE=1 SV=1 |
| MCG140959, isoform CRA_a OS=Homo sapiens GN=Myl6 PE=2 SV=1 | MCG116386, isoform CRA_a OS=Homo sapiens GN=Rbm3 PE=1 SV=1 |
| MCG14259, isoform CRA_a OS=Homo sapiens GN=U2af1 PE=1 SV=1 | MCG116671 OS=Homo sapiens GN=Gm11361 PE=3 SV=1 |
| MCG147570 (Fragment) OS=Homo sapiens GN=E130201H02Rik PE=2 SV=1 | MCG12304 OS=Homo sapiens GN=Rpl22 PE=1 SV=1 |
| MCG15083 OS=Homo sapiens GN=Try5 PE=1 SV=1 | MCG123152 OS=Homo sapiens GN=Gm10269 PE=1 SV=1 |
| MCG15085 OS=Homo sapiens GN=Try4 PE=1 SV=1 | MCG124046 OS=Homo sapiens GN=Prss1 PE=1 SV=1 |
| MCG17585 OS=Homo sapiens GN=Rpl39 PE=2 SV=1 | MCG126194, isoform CRA_a OS=Homo sapiens GN=Rpl31 PE=1 SV=1 |
| MCG17902, isoform CRA_a OS=Homo sapiens GN=Srsf7 PE=2 SV=1 | MCG132477, isoform CRA_a OS=Homo sapiens GN=Rpl18 PE=1 SV=1 |
| MCG18564, isoform CRA_a OS=Homo sapiens GN=Rpl12 PE=1 SV=1 | MCG13402, isoform CRA_a OS=Homo sapiens GN=Ptbp1 PE=1 SV=1 |
| MCG20799 OS=Homo sapiens GN=Rpl30 PE=1 SV=1 | MCG13402, isoform CRA_c OS=Homo sapiens GN=Ptbp1 PE=1 SV=1 |
| MCG21131, isoform CRA_a OS=Homo sapiens GN=Srsf3 PE=2 SV=1 | MCG13402, isoform CRA_d OS=Homo sapiens GN=Ptbp1 PE=1 SV=1 |
| MCG21688 OS=Homo sapiens GN=Rps10 PE=1 SV=1 | MCG140437, isoform CRA_d OS=Homo sapiens GN=Myh2 PE=1 SV=1 |
| MCG21910 OS=Homo sapiens GN=Gm12355 PE=4 SV=1 | MCG140959, isoform CRA_a OS=Homo sapiens GN=Myl6 PE=2 SV=1 |
| MCG23000, isoform CRA_b OS=Homo sapiens GN=Rps18 PE=2 SV=1 | MCG14259, isoform CRA_a OS=Homo sapiens GN=U2af1 PE=1 SV=1 |
| MCG2872, isoform CRA_b OS=Homo sapiens GN=Ddx5 PE=1 SV=1 | MCG14259, isoform CRA_b OS=Homo sapiens GN=U2af1 PE=1 SV=1 |
| MCG3574 OS=Homo sapiens GN=Rps20 PE=1 SV=1 | MCG147570 (Fragment) OS=Homo sapiens GN=E130201H02Rik PE=2 SV=1 |
| MCG4647 OS=Homo sapiens GN=Hmgb2 PE=2 SV=1 | MCG15083 OS=Homo sapiens GN=Try5 PE=1 SV=1 |
| MCG4862 OS=Homo sapiens GN=Snrpd2 PE=1 SV=1 | MCG15085 OS=Homo sapiens GN=Try4 PE=1 SV=1 |
| MCG50795 OS=Homo sapiens GN=Rpl9-ps1 PE=4 SV=1 | MCG17585 OS=Homo sapiens GN=Rpl39 PE=2 SV=1 |
| MCG5400 OS=Homo sapiens GN=Myl12a PE=1 SV=1 | MCG17902, isoform CRA_a OS=Homo sapiens GN=Srsf7 PE=2 SV=1 |
| MCG7614, isoform CRA_c OS=Homo sapiens GN=Srsf5 PE=1 SV=1 | MCG18564, isoform CRA_a OS=Homo sapiens GN=Rpl12 PE=1 SV=1 |
| Microtubule-associated protein 4 OS=Homo sapiens GN=Map4 PE=1 SV=3 | MCG20799 OS=Homo sapiens GN=Rpl30 PE=1 SV=1 |
| MKIAA0051 protein (Fragment) OS=Homo sapiens GN=Iqgap1 PE=2 SV=1 | MCG21131, isoform CRA_a OS=Homo sapiens GN=Srsf3 PE=2 SV=1 |
| MKIAA0144 protein (Fragment) OS=Homo sapiens GN=Ubap2l PE=2 SV=1 | MCG21688 OS=Homo sapiens GN=Rps10 PE=1 SV=1 |
| MKIAA0400 protein (Fragment) OS=Homo sapiens GN=Asap2 PE=2 SV=1 | MCG21910 OS=Homo sapiens GN=Gm12355 PE=4 SV=1 |
| MKIAA0741 protein (Fragment) OS=Homo sapiens GN=Eif5b PE=2 SV=1 | MCG23000, isoform CRA_b OS=Homo sapiens GN=Rps18 PE=2 SV=1 |
| MKIAA0866 protein (Fragment) OS=Homo sapiens GN=Myh11 PE=2 SV=1 | MCG2872, isoform CRA_b OS=Homo sapiens GN=Ddx5 PE=1 SV=1 |
| MKIAA0936 protein (Fragment) OS=Homo sapiens GN=Ick PE=2 SV=1 | MCG3574 OS=Homo sapiens GN=Rps20 PE=1 SV=1 |
| MKIAA3005 protein (Fragment) OS=Homo sapiens GN=Myh10 PE=2 SV=1 | MCG4647 OS=Homo sapiens GN=Hmgb2 PE=2 SV=1 |
| MKIAA4075 protein (Fragment) OS=Homo sapiens GN=Chd4 PE=2 SV=1 | MCG4862 OS=Homo sapiens GN=Snrpd2 PE=1 SV=1 |
| MKIAA4115 protein (Fragment) OS=Homo sapiens GN=G3bp1 PE=2 SV=1 | MCG53108 OS=Homo sapiens GN=Rbm31y PE=2 SV=1 |
| MKIAA4193 protein (Fragment) OS=Homo sapiens GN=Hnrnpm PE=2 SV=1 | MCG5400 OS=Homo sapiens GN=Myl12a PE=1 SV=1 |
| Moesin OS=Homo sapiens GN=Msn PE=1 SV=3 | MCG7614, isoform CRA_c OS=Homo sapiens GN=Srsf5 PE=1 SV=1 |
| Msn protein (Fragment) OS=Homo sapiens GN=Msn PE=2 SV=1 | MCG8382, isoform CRA_c OS=Homo sapiens GN=Rbm14 PE=1 SV=2 |
| MYB binding protein (P160) 1a, isoform CRA_b OS=Homo sapiens GN=Mybbp1a PE=1 SV=1 | Microtubule-associated protein (Fragment) OS=Homo sapiens GN=Map4 PE=1 SV=1 |
| MYB-1a OS=Homo sapiens GN=Ybx1 PE=2 SV=1 | Microtubule-associated protein 1A OS=Homo sapiens GN=Map1a PE=1 SV=1 |
| MYB-1b OS=Homo sapiens GN=Ybx1 PE=2 SV=1 | Microtubule-associated protein 1A OS=Homo sapiens GN=Map1a PE=1 SV=2 |
| Mybbp1a protein (Fragment) OS=Homo sapiens GN=Mybbp1a PE=2 SV=1 | Microtubule-associated protein 1B OS=Homo sapiens GN=Map1b PE=1 SV=2 |
| Myh10 protein (Fragment) OS=Homo sapiens GN=Myh10 PE=2 SV=1 | Microtubule-associated protein 1B OS=Homo sapiens GN=Map1b PE=2 SV=1 |
| Myh14 protein (Fragment) OS=Homo sapiens GN=Myh14 PE=2 SV=1 | Microtubule-associated protein 4 OS=Homo sapiens GN=Map4 PE=1 SV=3 |
| Myh2 protein OS=Homo sapiens GN=Myh2 PE=2 SV=1 | Microtubule-associated protein OS=Homo sapiens GN=Map4 PE=1 SV=1 |
| Myh9 protein (Fragment) OS=Homo sapiens GN=Myh9 PE=2 SV=1 | Microtubule-associated protein OS=Homo sapiens GN=Map4 PE=1 SV=3 |
| Myh9 protein OS=Homo sapiens GN=Myh9 PE=2 SV=1 | MKIAA0051 protein (Fragment) OS=Homo sapiens GN=Iqgap1 PE=2 SV=1 |
| Myosin heavy chain IIB (Fragment) OS=Homo sapiens GN=Myh4 PE=2 SV=1 | MKIAA0144 protein (Fragment) OS=Homo sapiens GN=Ubap2l PE=2 SV=1 |
| Myosin heavy chain IIX (Fragment) OS=Homo sapiens GN=Myh1 PE=2 SV=1 | MKIAA0670 protein (Fragment) OS=Homo sapiens GN=Acin1 PE=2 SV=1 |
| Myosin light chain 6B OS=Homo sapiens GN=Myl6b PE=1 SV=1 | MKIAA0741 protein (Fragment) OS=Homo sapiens GN=Eif5b PE=2 SV=1 |
| Myosin light polypeptide 6 alkali smooth muscle and non-muscle protein (Fragment) OS=Homo sapiens GN=Myl6 PE=2 SV=1 | MKIAA0845 protein (Fragment) OS=Homo sapiens GN=Nefh PE=2 SV=1 |
| Myosin light polypeptide 6 OS=Homo sapiens GN=Myl6 PE=1 SV=1 | MKIAA0866 protein (Fragment) OS=Homo sapiens GN=Myh11 PE=2 SV=1 |
| Myosin light polypeptide 6 OS=Homo sapiens GN=Myl6 PE=1 SV=3 | MKIAA0936 protein (Fragment) OS=Homo sapiens GN=Ick PE=2 SV=1 |
| Myosin regulatory light chain 12B OS=Homo sapiens GN=Myl12b PE=1 SV=2 | MKIAA1172 protein (Fragment) OS=Homo sapiens GN=Scaf4 PE=2 SV=1 |
| Myosin, heavy polypeptide 1, skeletal muscle, adult OS=Homo sapiens GN=Myh1 PE=2 SV=1 | MKIAA3005 protein (Fragment) OS=Homo sapiens GN=Myh10 PE=2 SV=1 |
| Myosin, heavy polypeptide 8, skeletal muscle, perinatal OS=Homo sapiens GN=Myh8 PE=2 SV=1 | MKIAA4115 protein (Fragment) OS=Homo sapiens GN=G3bp1 PE=2 SV=1 |
| Myosin-1 OS=Homo sapiens GN=Myh1 PE=1 SV=1 | MKIAA4193 protein (Fragment) OS=Homo sapiens GN=Hnrnpm PE=2 SV=1 |
| Myosin-10 OS=Homo sapiens GN=Myh10 PE=1 SV=1 | MYB binding protein (P160) 1a, isoform CRA_b OS=Homo sapiens GN=Mybbp1a PE=1 SV=1 |
| Myosin-10 OS=Homo sapiens GN=Myh10 PE=1 SV=2 | MYB-1a OS=Homo sapiens GN=Ybx1 PE=2 SV=1 |
| Myosin-11 OS=Homo sapiens GN=Myh11 PE=1 SV=1 | MYB-1b OS=Homo sapiens GN=Ybx1 PE=2 SV=1 |
| Myosin-14 (Fragment) OS=Homo sapiens GN=Myh14 PE=1 SV=1 | Mybbp1a protein (Fragment) OS=Homo sapiens GN=Mybbp1a PE=2 SV=1 |
| Myosin-14 OS=Homo sapiens GN=Myh14 PE=1 SV=1 | Myh10 protein (Fragment) OS=Homo sapiens GN=Myh10 PE=2 SV=1 |
| Myosin-4 OS=Homo sapiens GN=Myh4 PE=2 SV=1 | Myh14 protein (Fragment) OS=Homo sapiens GN=Myh14 PE=2 SV=1 |
| Myosin-8 OS=Homo sapiens GN=Myh8 PE=2 SV=2 | Myh2 protein OS=Homo sapiens GN=Myh2 PE=2 SV=1 |
| Myosin-9 OS=Homo sapiens GN=Myh9 PE=1 SV=1 | Myh9 protein (Fragment) OS=Homo sapiens GN=Myh9 PE=2 SV=1 |
| Myosin-9 OS=Homo sapiens GN=Myh9 PE=1 SV=4 | Myh9 protein OS=Homo sapiens GN=Myh9 PE=2 SV=1 |
| Nascent polypeptide-associated complex subunit alpha OS=Homo sapiens GN=Naca PE=1 SV=1 | Myosin heavy chain IIB (Fragment) OS=Homo sapiens GN=Myh4 PE=2 SV=1 |
| Nascent polypeptide-associated complex subunit alpha, muscle-specific form OS=Homo sapiens GN=Naca PE=1 SV=2 | Myosin light chain 6B OS=Homo sapiens GN=Myl6b PE=1 SV=1 |
| Nestin OS=Homo sapiens GN=Nes PE=1 SV=1 | Myosin light polypeptide 6 OS=Homo sapiens GN=Myl6 PE=1 SV=1 |
| Nonmuscle myosin heavy chain-A (Fragment) OS=Homo sapiens GN=Myh9 PE=2 SV=1 | Myosin light polypeptide 6 OS=Homo sapiens GN=Myl6 PE=1 SV=3 |
| Nono protein OS=Homo sapiens GN=Nono PE=2 SV=1 | Myosin regulatory light chain 12B OS=Homo sapiens GN=Myl12b PE=1 SV=2 |
| Non-specific protein-tyrosine kinase (Fragment) OS=Homo sapiens GN=Lck PE=2 SV=1 | Myosin, heavy polypeptide 1, skeletal muscle, adult OS=Homo sapiens GN=Myh1 PE=2 SV=1 |
| Nuclease-sensitive element-binding protein 1 (Fragment) OS=Homo sapiens GN=Ybx1 PE=1 SV=1 | Myosin, heavy polypeptide 8, skeletal muscle, perinatal OS=Homo sapiens GN=Myh8 PE=2 SV=1 |
| Nucleolin OS=Homo sapiens GN=Ncl PE=1 SV=2 | Myosin-1 OS=Homo sapiens GN=Myh1 PE=1 SV=1 |
| NudC domain-containing protein 2 OS=Homo sapiens GN=Nudcd2 PE=1 SV=1 | Myosin-10 OS=Homo sapiens GN=Myh10 PE=1 SV=1 |
| p68 RNA helicase (Fragment) OS=Homo sapiens GN=Hlr1 PE=4 SV=1 | Myosin-10 OS=Homo sapiens GN=Myh10 PE=1 SV=2 |
| PC4 and SFRS1-interacting protein (Fragment) OS=Homo sapiens GN=Psip1 PE=1 SV=1 | Myosin-11 OS=Homo sapiens GN=Myh11 PE=1 SV=1 |
| PC4 and SFRS1-interacting protein OS=Homo sapiens GN=Psip1 PE=1 SV=1 | Myosin-14 OS=Homo sapiens GN=Myh14 PE=1 SV=1 |
| PCTAIRE-motif protein kinase 1 OS=Homo sapiens GN=Cdk16 PE=2 SV=1 | Myosin-4 OS=Homo sapiens GN=Myh4 PE=2 SV=1 |
| PCTAIRE-motif protein kinase 3 OS=Homo sapiens GN=Cdk18 PE=1 SV=1 | Myosin-8 OS=Homo sapiens GN=Myh8 PE=2 SV=2 |
| Pctk2 protein OS=Homo sapiens GN=Cdk17 PE=2 SV=1 | Myosin-9 OS=Homo sapiens GN=Myh9 PE=1 SV=1 |
| Pctk3 protein (Fragment) OS=Homo sapiens GN=Cdk18 PE=2 SV=1 | Myosin-9 OS=Homo sapiens GN=Myh9 PE=1 SV=4 |
| Peptidyl-prolyl cis-trans isomerase OS=Homo sapiens GN=Ppib PE=1 SV=1 | Nascent polypeptide-associated complex subunit alpha OS=Homo sapiens GN=Naca PE=1 SV=1 |
| Peptidyl-prolyl cis-trans isomerase OS=Homo sapiens GN=Ppib PE=2 SV=1 | Nascent polypeptide-associated complex subunit alpha, muscle-specific form OS=Homo sapiens GN=Naca PE=1 SV=2 |
| Peripherin OS=Homo sapiens GN=Prph PE=1 SV=1 | Nefl protein (Fragment) OS=Homo sapiens GN=Nefl PE=2 SV=1 |
| Peripherin OS=Homo sapiens GN=Prph PE=1 SV=2 | Neurofilament 3, medium OS=Homo sapiens GN=Nefm PE=1 SV=1 |
| Peroxiredoxin-1 (Fragment) OS=Homo sapiens GN=Prdx1 PE=1 SV=1 | Neurofilament heavy polypeptide OS=Homo sapiens GN=Nefh PE=1 SV=3 |
| Peroxiredoxin-1 (Fragment) OS=Homo sapiens GN=Prdx1 PE=1 SV=8 | Neurofilament light polypeptide OS=Homo sapiens GN=Nefl PE=1 SV=5 |
| Peroxiredoxin-1 OS=Homo sapiens GN=Prdx1 PE=1 SV=1 | Neurofilament medium polypeptide OS=Homo sapiens GN=Nefm PE=1 SV=1 |
| Pftk1 protein OS=Homo sapiens GN=Cdk14 PE=2 SV=1 | Neurofilament medium polypeptide OS=Homo sapiens GN=Nefm PE=1 SV=4 |
| Plasminogen activator inhibitor 1 RNA-binding protein (Fragment) OS=Homo sapiens GN=Serbp1 PE=1 SV=1 | Nonmuscle myosin heavy chain-A (Fragment) OS=Homo sapiens GN=Myh9 PE=2 SV=1 |
| Plasminogen activator inhibitor 1 RNA-binding protein OS=Homo sapiens GN=Serbp1 PE=1 SV=1 | Nono protein OS=Homo sapiens GN=Nono PE=2 SV=1 |
| Plasminogen activator inhibitor 1 RNA-binding protein OS=Homo sapiens GN=Serbp1 PE=1 SV=2 | Non-specific protein-tyrosine kinase (Fragment) OS=Homo sapiens GN=Lck PE=2 SV=1 |
| Plec1 protein (Fragment) OS=Homo sapiens GN=Plec PE=2 SV=1 | Nuclear receptor-binding protein OS=Homo sapiens GN=Nrbp1 PE=1 SV=1 |
| Plectin (Fragment) OS=Homo sapiens GN=Plec PE=1 SV=1 | Nuclease-sensitive element-binding protein 1 (Fragment) OS=Homo sapiens GN=Ybx1 PE=1 SV=1 |
| Plectin 10 OS=Homo sapiens GN=Plec PE=2 SV=1 | Nucleolin OS=Homo sapiens GN=Ncl PE=1 SV=2 |
| Plectin 2 OS=Homo sapiens GN=Plec PE=2 SV=1 | NudC domain-containing protein 2 OS=Homo sapiens GN=Nudcd2 PE=1 SV=1 |
| Plectin 3 OS=Homo sapiens GN=Plec PE=2 SV=1 | p68 RNA helicase (Fragment) OS=Homo sapiens GN=Hlr1 PE=4 SV=1 |
| Plectin 4 OS=Homo sapiens GN=Plec PE=2 SV=1 | PC4 and SFRS1-interacting protein (Fragment) OS=Homo sapiens GN=Psip1 PE=1 SV=1 |
| Plectin 7 OS=Homo sapiens GN=Plec PE=2 SV=1 | PC4 and SFRS1-interacting protein OS=Homo sapiens GN=Psip1 PE=1 SV=1 |
| Plectin 8 OS=Homo sapiens GN=Plec PE=2 SV=1 | Pcbp3 protein OS=Homo sapiens GN=Pcbp3 PE=2 SV=1 |
| Plectin OS=Homo sapiens GN=Plec PE=1 SV=1 | PCTAIRE-motif protein kinase 1 OS=Homo sapiens GN=Cdk16 PE=2 SV=1 |
| Plectin OS=Homo sapiens GN=Plec PE=1 SV=3 | PCTAIRE-motif protein kinase 3 OS=Homo sapiens GN=Cdk18 PE=1 SV=1 |
| Poly A binding protein, cytoplasmic 5 OS=Homo sapiens GN=Pabpc5 PE=2 SV=1 | Pctk2 protein OS=Homo sapiens GN=Cdk17 PE=2 SV=1 |
| Poly(A)-binding protein cytoplasmic 5 (Fragment) OS=Homo sapiens GN=PABPC5 PE=4 SV=1 | Pctk3 protein (Fragment) OS=Homo sapiens GN=Cdk18 PE=2 SV=1 |
| Poly(A)-binding protein, cytoplasmic 4 OS=Homo sapiens GN=Pabpc4 PE=4 SV=1 | Peptidyl-prolyl cis-trans isomerase OS=Homo sapiens GN=Ppib PE=1 SV=1 |
| Poly(rC)-binding protein 1 OS=Homo sapiens GN=Pcbp1 PE=1 SV=1 | Peptidyl-prolyl cis-trans isomerase OS=Homo sapiens GN=Ppib PE=2 SV=1 |
| Poly(U)-binding-splicing factor PUF60 OS=Homo sapiens GN=Puf60 PE=1 SV=2 | Peripherin OS=Homo sapiens GN=Prph PE=1 SV=1 |
| Polyadenylate-binding protein (Fragment) OS=Homo sapiens GN=Pabpc1 PE=2 SV=1 | Peripherin OS=Homo sapiens GN=Prph PE=1 SV=2 |
| Polyadenylate-binding protein 1 (Fragment) OS=Homo sapiens GN=Pabpc1 PE=1 SV=1 | Pftk1 protein OS=Homo sapiens GN=Cdk14 PE=2 SV=1 |
| Polyadenylate-binding protein 1 OS=Homo sapiens GN=Pabpc1 PE=1 SV=2 | Phosphatidylinositol transfer protein beta isoform OS=Homo sapiens GN=Pitpnb PE=1 SV=1 |
| Polyadenylate-binding protein OS=Homo sapiens GN=Gm10110 PE=3 SV=1 | Phosphatidylinositol transfer protein beta isoform OS=Homo sapiens GN=Pitpnb PE=1 SV=2 |
| Polyadenylate-binding protein OS=Homo sapiens GN=Pabpc1 PE=2 SV=1 | Plasminogen activator inhibitor 1 RNA-binding protein (Fragment) OS=Homo sapiens GN=Serbp1 PE=1 SV=1 |
| Polyadenylate-binding protein OS=Homo sapiens GN=Pabpc2 PE=1 SV=1 | Plasminogen activator inhibitor 1 RNA-binding protein OS=Homo sapiens GN=Serbp1 PE=1 SV=1 |
| Polyadenylate-binding protein OS=Homo sapiens GN=Pabpc4 PE=1 SV=1 | Plasminogen activator inhibitor 1 RNA-binding protein OS=Homo sapiens GN=Serbp1 PE=1 SV=2 |
| Polyadenylate-binding protein OS=Homo sapiens GN=Pabpc4 PE=2 SV=1 | Plec1 protein (Fragment) OS=Homo sapiens GN=Plec PE=2 SV=1 |
| Polyadenylate-binding protein OS=Homo sapiens GN=Pabpc6 PE=1 SV=1 | Plectin (Fragment) OS=Homo sapiens GN=Plec PE=1 SV=1 |
| Polyadenylate-binding protein OS=Homo sapiens PE=3 SV=1 | Plectin 10 OS=Homo sapiens GN=Plec PE=2 SV=1 |
| Predicted gene 10036 OS=Homo sapiens GN=Gm10036 PE=3 SV=1 | Plectin 2 OS=Homo sapiens GN=Plec PE=2 SV=1 |
| Predicted gene 10260 OS=Homo sapiens GN=Gm10260 PE=3 SV=2 | Plectin 3 OS=Homo sapiens GN=Plec PE=2 SV=1 |
| Predicted gene 14214 (Fragment) OS=Homo sapiens GN=Gm14214 PE=4 SV=1 | Plectin 4 OS=Homo sapiens GN=Plec PE=2 SV=1 |
| Predicted gene 15294 OS=Homo sapiens GN=Gm15294 PE=1 SV=1 | Plectin 7 OS=Homo sapiens GN=Plec PE=2 SV=1 |
| Predicted gene 17087 OS=Homo sapiens GN=Gm17087 PE=1 SV=1 | Plectin 8 OS=Homo sapiens GN=Plec PE=2 SV=1 |
| Predicted gene 2016 OS=Homo sapiens GN=Gm2016 PE=2 SV=1 | Plectin OS=Homo sapiens GN=Plec PE=1 SV=1 |
| Predicted gene 20425 OS=Homo sapiens GN=Gm20425 PE=4 SV=1 | Plectin OS=Homo sapiens GN=Plec PE=1 SV=3 |
| Predicted gene 2056 OS=Homo sapiens GN=Gm2056 PE=3 SV=1 | Poly [ADP-ribose] polymerase (Fragment) OS=Homo sapiens GN=Parp1 PE=2 SV=1 |
| Predicted gene 2075 OS=Homo sapiens GN=Gm2075 PE=3 SV=1 | Poly [ADP-ribose] polymerase OS=Homo sapiens GN=Parp1 PE=1 SV=1 |
| Predicted gene 2663 OS=Homo sapiens GN=Gm2663 PE=3 SV=1 | Poly [ADP-ribose] polymerase OS=Homo sapiens GN=Parp1 PE=2 SV=1 |
| Predicted gene 5039 OS=Homo sapiens GN=Gm5039 PE=3 SV=1 | Poly A binding protein, cytoplasmic 5 OS=Homo sapiens GN=Pabpc5 PE=2 SV=1 |
| Predicted gene 5093 OS=Homo sapiens GN=Gm5093 PE=1 SV=1 | Poly(A)-binding protein cytoplasmic 5 (Fragment) OS=Homo sapiens GN=PABPC5 PE=4 SV=1 |
| Predicted gene 5662 OS=Homo sapiens GN=Gm5662 PE=2 SV=1 | Poly(A)-binding protein, cytoplasmic 4 OS=Homo sapiens GN=Pabpc4 PE=4 SV=1 |
| Predicted gene 6096 OS=Homo sapiens GN=Gm6096 PE=1 SV=1 | Poly(RC) binding protein 2 OS=Homo sapiens GN=Pcbp2 PE=2 SV=1 |
| Predicted gene 6576 OS=Homo sapiens GN=Gm6576 PE=3 SV=2 | Poly(rC)-binding protein 1 OS=Homo sapiens GN=Pcbp1 PE=1 SV=1 |
| Predicted gene 6803 OS=Homo sapiens GN=Gm6803 PE=3 SV=1 | Poly(rC)-binding protein 2 OS=Homo sapiens GN=Pcbp2 PE=1 SV=1 |
| Predicted gene 8225 OS=Homo sapiens GN=Gm8225 PE=3 SV=1 | Poly(rC)-binding protein 3 OS=Homo sapiens GN=Pcbp3 PE=1 SV=1 |
| Predicted gene 8300 OS=Homo sapiens GN=Gm8332 PE=2 SV=1 | Poly(rC)-binding protein 3 OS=Homo sapiens GN=Pcbp3 PE=1 SV=3 |
| Predicted gene, 16519 OS=Homo sapiens GN=Gm16519 PE=1 SV=2 | Poly(U)-binding-splicing factor PUF60 OS=Homo sapiens GN=Puf60 PE=1 SV=2 |
| Predicted gene, 17430 OS=Homo sapiens GN=Gm17430 PE=4 SV=1 | Polyadenylate-binding protein (Fragment) OS=Homo sapiens GN=Pabpc1 PE=2 SV=1 |
| Predicted gene, 21936 OS=Homo sapiens GN=Gm21936 PE=3 SV=1 | Polyadenylate-binding protein 1 (Fragment) OS=Homo sapiens GN=Pabpc1 PE=1 SV=1 |
| Predicted pseudogene 10073 OS=Homo sapiens GN=Gm10073 PE=1 SV=1 | Polyadenylate-binding protein 1 OS=Homo sapiens GN=Pabpc1 PE=1 SV=2 |
| Predicted pseudogene 2035 OS=Homo sapiens GN=Gm2035 PE=3 SV=1 | Polyadenylate-binding protein OS=Homo sapiens GN=Gm10110 PE=3 SV=1 |
| Prelamin-A/C (Fragment) OS=Homo sapiens GN=Lmna PE=1 SV=1 | Polyadenylate-binding protein OS=Homo sapiens GN=Pabpc1 PE=2 SV=1 |
| Prelamin-A/C OS=Homo sapiens GN=Lmna PE=1 SV=2 | Polyadenylate-binding protein OS=Homo sapiens GN=Pabpc1l PE=2 SV=1 |
| Pre-mRNA-processing factor 19 OS=Homo sapiens GN=Prpf19 PE=1 SV=1 | Polyadenylate-binding protein OS=Homo sapiens GN=Pabpc2 PE=1 SV=1 |
| Pre-rRNA-processing protein TSR1 homolog OS=Homo sapiens GN=Tsr1 PE=1 SV=1 | Polyadenylate-binding protein OS=Homo sapiens GN=Pabpc4 PE=1 SV=1 |
| Probable ATP-dependent RNA helicase DDX17 OS=Homo sapiens GN=Ddx17 PE=1 SV=1 | Polyadenylate-binding protein OS=Homo sapiens GN=Pabpc4 PE=2 SV=1 |
| Probable ATP-dependent RNA helicase DDX5 (Fragment) OS=Homo sapiens GN=Ddx5 PE=1 SV=8 | Polyadenylate-binding protein OS=Homo sapiens GN=Pabpc6 PE=1 SV=1 |
| Probable ATP-dependent RNA helicase DDX5 OS=Homo sapiens GN=Ddx5 PE=1 SV=1 | Polyadenylate-binding protein OS=Homo sapiens PE=3 SV=1 |
| Probable ATP-dependent RNA helicase DDX5 OS=Homo sapiens GN=Ddx5 PE=1 SV=2 | Polypirimidine tract binding protein OS=Homo sapiens GN=Ptbp1 PE=2 SV=1 |
| Probable ATP-dependent RNA helicase DDX6 OS=Homo sapiens GN=Ddx6 PE=1 SV=1 | Polypyrimidine tract binding protein 1 OS=Homo sapiens GN=Ptbp1 PE=2 SV=1 |
| Protein 4.1G OS=Homo sapiens GN=Epb41l2 PE=2 SV=1 | Polypyrimidine tract-binding protein 1 (Fragment) OS=Homo sapiens GN=Ptbp1 PE=1 SV=1 |
| Protein arginine N-methyltransferase 3 OS=Homo sapiens GN=Prmt3 PE=1 SV=2 | Polypyrimidine tract-binding protein 1 OS=Homo sapiens GN=Ptbp1 PE=1 SV=1 |
| Protein LYRIC OS=Homo sapiens GN=Mtdh PE=1 SV=1 | Polypyrimidine tract-binding protein 1 OS=Homo sapiens GN=Ptbp1 PE=1 SV=2 |
| Protein RCC2 (Fragment) OS=Homo sapiens GN=Rcc2 PE=1 SV=1 | Predicted gene 10036 OS=Homo sapiens GN=Gm10036 PE=3 SV=1 |
| Protein RCC2 OS=Homo sapiens GN=Rcc2 PE=1 SV=1 | Predicted gene 10260 OS=Homo sapiens GN=Gm10260 PE=3 SV=2 |
| Proto-oncogene tyrosine-protein kinase LCK OS=Homo sapiens GN=Lck PE=1 SV=4 | Predicted gene 14214 (Fragment) OS=Homo sapiens GN=Gm14214 PE=4 SV=1 |
| Psip1 protein (Fragment) OS=Homo sapiens GN=Psip1 PE=2 SV=1 | Predicted gene 15294 OS=Homo sapiens GN=Gm15294 PE=1 SV=1 |
| PTB-associated splicing factor (Fragment) OS=Homo sapiens GN=Sfpq PE=2 SV=1 | Predicted gene 17087 OS=Homo sapiens GN=Gm17087 PE=1 SV=1 |
| Puf60 protein (Fragment) OS=Homo sapiens GN=Puf60 PE=2 SV=1 | Predicted gene 17190 OS=Homo sapiens GN=Gm17190 PE=1 SV=1 |
| Putative ATP-dependent RNA helicase Pl10 OS=Homo sapiens GN=D1Pas1 PE=1 SV=1 | Predicted gene 20425 OS=Homo sapiens GN=Gm20425 PE=4 SV=1 |
| Pyruvate kinase (Fragment) OS=Homo sapiens GN=Pklr PE=2 SV=1 | Predicted gene 2663 OS=Homo sapiens GN=Gm2663 PE=3 SV=1 |
| Pyruvate kinase OS=Homo sapiens GN=Pklr PE=1 SV=1 | Predicted gene 5093 OS=Homo sapiens GN=Gm5093 PE=1 SV=1 |
| Pyruvate kinase OS=Homo sapiens GN=Pklr PE=2 SV=1 | Predicted gene 6096 OS=Homo sapiens GN=Gm6096 PE=1 SV=1 |
| Pyruvate kinase PKLR OS=Homo sapiens GN=Pklr PE=1 SV=1 | Predicted gene 6576 OS=Homo sapiens GN=Gm6576 PE=3 SV=2 |
| Pyruvate kinase PKM (Fragment) OS=Homo sapiens GN=Pkm PE=1 SV=1 | Predicted gene 8225 OS=Homo sapiens GN=Gm8225 PE=3 SV=1 |
| Pyruvate kinase PKM OS=Homo sapiens GN=Pkm PE=1 SV=4 | Predicted gene, 17430 OS=Homo sapiens GN=Gm17430 PE=4 SV=1 |
| Radixin OS=Homo sapiens GN=Rdx PE=1 SV=1 | Predicted pseudogene 10073 OS=Homo sapiens GN=Gm10073 PE=1 SV=1 |
| Radixin OS=Homo sapiens GN=Rdx PE=1 SV=3 | Predicted pseudogene 9242 OS=Homo sapiens GN=Gm9242 PE=4 SV=1 |
| Ras GTPase-activating protein-binding protein 1 OS=Homo sapiens GN=G3bp1 PE=1 SV=1 | Pre-mRNA-processing factor 19 OS=Homo sapiens GN=Prpf19 PE=1 SV=1 |
| Ras GTPase-activating protein-binding protein 2 OS=Homo sapiens GN=G3bp2 PE=1 SV=2 | Pre-rRNA-processing protein TSR1 homolog OS=Homo sapiens GN=Tsr1 PE=1 SV=1 |
| Ras GTPase-activating-like protein IQGAP1 OS=Homo sapiens GN=Iqgap1 PE=1 SV=2 | Probable ATP-dependent RNA helicase DDX17 OS=Homo sapiens GN=Ddx17 PE=1 SV=1 |
| Rbm39 protein OS=Homo sapiens GN=Rbm39 PE=2 SV=1 | Probable ATP-dependent RNA helicase DDX5 (Fragment) OS=Homo sapiens GN=Ddx5 PE=1 SV=8 |
| Rdx protein (Fragment) OS=Homo sapiens GN=Rdx PE=2 SV=1 | Probable ATP-dependent RNA helicase DDX5 OS=Homo sapiens GN=Ddx5 PE=1 SV=1 |
| Receptor protein-tyrosine kinase (Fragment) OS=Homo sapiens GN=Fgfr2 PE=1 SV=1 | Probable ATP-dependent RNA helicase DDX5 OS=Homo sapiens GN=Ddx5 PE=1 SV=2 |
| Receptor protein-tyrosine kinase (Fragment) OS=Homo sapiens GN=Fgfr3 PE=2 SV=1 | Protein LYRIC OS=Homo sapiens GN=Mtdh PE=1 SV=1 |
| Ribosomal protein L14 OS=Homo sapiens GN=Rpl14-ps1 PE=2 SV=1 | Protein RCC2 (Fragment) OS=Homo sapiens GN=Rcc2 PE=1 SV=1 |
| Ribosomal protein L18 (Fragment) OS=Homo sapiens GN=Rpl18 PE=2 SV=1 | Protein RCC2 OS=Homo sapiens GN=Rcc2 PE=1 SV=1 |
| Ribosomal protein L18 OS=Homo sapiens GN=Rpl18 PE=2 SV=1 | Protein SCAF8 OS=Homo sapiens GN=Scaf8 PE=1 SV=1 |
| Ribosomal protein L19 OS=Homo sapiens GN=Rpl19 PE=1 SV=1 | Protein-serine/threonine phosphatase OS=Homo sapiens GN=Ppp1ca PE=2 SV=1 |
| Ribosomal protein L21 OS=Homo sapiens GN=Rpl21 PE=2 SV=1 | Proto-oncogene tyrosine-protein kinase LCK OS=Homo sapiens GN=Lck PE=1 SV=4 |
| Ribosomal protein L23A, pseudogene 3 OS=Homo sapiens GN=Rpl23a-ps3 PE=3 SV=1 | Psip1 protein (Fragment) OS=Homo sapiens GN=Psip1 PE=2 SV=1 |
| Ribosomal protein L3 (Fragment) OS=Homo sapiens GN=Rp13 PE=4 SV=1 | PTB-associated splicing factor (Fragment) OS=Homo sapiens GN=Sfpq PE=2 SV=1 |
| Ribosomal protein L32 OS=Homo sapiens GN=Rpl32 PE=2 SV=1 | Puf60 protein (Fragment) OS=Homo sapiens GN=Puf60 PE=2 SV=1 |
| Ribosomal protein L3-like OS=Homo sapiens GN=Rpl3l PE=1 SV=1 | Putative ATP-dependent RNA helicase Pl10 OS=Homo sapiens GN=D1Pas1 PE=1 SV=1 |
| Ribosomal protein L4 OS=Homo sapiens GN=Rpl4 PE=1 SV=1 | Putative gag-pro-pol polyprotein OS=Homo sapiens PE=4 SV=1 |
| Ribosomal protein L5 OS=Homo sapiens GN=Rpl5 PE=2 SV=1 | Putative RNA-binding protein Luc7-like 1 (Fragment) OS=Homo sapiens GN=Luc7l PE=1 SV=1 |
| Ribosomal protein L7A OS=Homo sapiens GN=Rpl7a PE=2 SV=1 | Putative RNA-binding protein Luc7-like 1 OS=Homo sapiens GN=Luc7l PE=1 SV=2 |
| Ribosomal protein L7A, pseudogene 3 OS=Homo sapiens GN=Rpl7a-ps3 PE=4 SV=1 | Putative RNA-binding protein Luc7-like 2 OS=Homo sapiens GN=Luc7l2 PE=1 SV=1 |
| Ribosomal protein L7A, pseudogene 5 OS=Homo sapiens GN=Rpl7a-ps5 PE=4 SV=1 | Pyruvate kinase (Fragment) OS=Homo sapiens GN=Pklr PE=2 SV=1 |
| Ribosomal protein L8 (Fragment) OS=Homo sapiens PE=4 SV=1 | Pyruvate kinase OS=Homo sapiens GN=Pklr PE=1 SV=1 |
| Ribosomal protein L9 OS=Homo sapiens GN=Rpl9 PE=2 SV=1 | Pyruvate kinase OS=Homo sapiens GN=Pklr PE=2 SV=1 |
| Ribosomal protein L9, pseudogene 6 OS=Homo sapiens GN=Rpl9-ps6 PE=4 SV=1 | Pyruvate kinase PKLR OS=Homo sapiens GN=Pklr PE=1 SV=1 |
| Ribosomal protein OS=Homo sapiens GN=Rpl10a PE=1 SV=1 | Pyruvate kinase PKM (Fragment) OS=Homo sapiens GN=Pkm PE=1 SV=1 |
| Ribosomal protein OS=Homo sapiens GN=Rpl10a PE=2 SV=1 | Pyruvate kinase PKM OS=Homo sapiens GN=Pkm PE=1 SV=4 |
| Ribosomal protein S14 OS=Homo sapiens GN=rps14 PE=3 SV=1 | Ras GTPase-activating protein-binding protein 1 OS=Homo sapiens GN=G3bp1 PE=1 SV=1 |
| Ribosomal protein S2 (Fragment) OS=Homo sapiens GN=Rps2 PE=2 SV=1 | Ras GTPase-activating protein-binding protein 2 OS=Homo sapiens GN=G3bp2 PE=1 SV=2 |
| Ribosomal protein S2, pseudogene 6 OS=Homo sapiens GN=Rps2-ps6 PE=3 SV=1 | Ras GTPase-activating-like protein IQGAP1 (Fragment) OS=Homo sapiens GN=Iqgap1 PE=1 SV=2 |
| Ribosomal protein S23 OS=Homo sapiens GN=Rps23 PE=2 SV=1 | Ras GTPase-activating-like protein IQGAP1 OS=Homo sapiens GN=Iqgap1 PE=1 SV=2 |
| Ribosomal protein S3 (Fragment) OS=Homo sapiens GN=Rps3 PE=2 SV=1 | Rbm39 protein OS=Homo sapiens GN=Rbm39 PE=2 SV=1 |
| Ribosomal protein S3 OS=Homo sapiens GN=Rps3 PE=1 SV=1 | Receptor protein-tyrosine kinase (Fragment) OS=Homo sapiens GN=Fgfr2 PE=1 SV=1 |
| Ribosome-binding protein 1 OS=Homo sapiens GN=Rrbp1 PE=1 SV=1 | Receptor protein-tyrosine kinase (Fragment) OS=Homo sapiens GN=Fgfr3 PE=2 SV=1 |
| Ribosome-binding protein 1 OS=Homo sapiens GN=Rrbp1 PE=1 SV=2 | Rho guanine nucleotide exchange factor 2 OS=Homo sapiens GN=Arhgef2 PE=1 SV=1 |
| RIKEN cDNA 1810009J06 gene OS=Homo sapiens GN=1810009J06Rik PE=2 SV=1 | Rho guanine nucleotide exchange factor 2 OS=Homo sapiens GN=Arhgef2 PE=1 SV=4 |
| RIKEN cDNA 2210010C04 gene OS=Homo sapiens GN=2210010C04Rik PE=1 SV=1 | Ribonuclease inhibitor (Fragment) OS=Homo sapiens GN=Rnh1 PE=1 SV=1 |
| RIKEN cDNA 9530053A07 gene OS=Homo sapiens GN=9530053A07Rik PE=1 SV=1 | Ribonuclease inhibitor OS=Homo sapiens GN=Rnh1 PE=1 SV=1 |
| RNA and export factor binding protein 2 OS=Homo sapiens GN=Alyref2 PE=2 SV=1 | Ribosomal protein L18 (Fragment) OS=Homo sapiens GN=Rpl18 PE=2 SV=1 |
| RNA and export factor binding protein 2 OS=Homo sapiens GN=Refbp2 PE=4 SV=1 | Ribosomal protein L18 OS=Homo sapiens GN=Rpl18 PE=2 SV=1 |
| RNA-binding protein 39 (Fragment) OS=Homo sapiens GN=Rbm39 PE=1 SV=1 | Ribosomal protein L19 OS=Homo sapiens GN=Rpl19 PE=1 SV=1 |
| RNA-binding protein 39 OS=Homo sapiens GN=Rbm39 PE=1 SV=1 | Ribosomal protein L21 OS=Homo sapiens GN=Rpl21 PE=2 SV=1 |
| RNA-binding protein 39 OS=Homo sapiens GN=Rbm39 PE=1 SV=2 | Ribosomal protein L23A, pseudogene 3 OS=Homo sapiens GN=Rpl23a-ps3 PE=3 SV=1 |
| RNA-binding protein EWS OS=Homo sapiens GN=Ewsr1 PE=1 SV=1 | Ribosomal protein L3 (Fragment) OS=Homo sapiens GN=Rp13 PE=4 SV=1 |
| RNA-binding protein EWS OS=Homo sapiens GN=Ewsr1 PE=1 SV=2 | Ribosomal protein L3-like OS=Homo sapiens GN=Rpl3l PE=1 SV=1 |
| RNA-binding protein FUS (Fragment) OS=Homo sapiens GN=Fus PE=1 SV=1 | Ribosomal protein L4 (Fragment) OS=Homo sapiens GN=Rpl4 PE=2 SV=1 |
| Rpl11 protein OS=Homo sapiens GN=Rpl11 PE=2 SV=1 | Ribosomal protein L4 OS=Homo sapiens GN=Rpl4 PE=1 SV=1 |
| Rpl12 protein (Fragment) OS=Homo sapiens GN=Rpl12 PE=2 SV=1 | Ribosomal protein L7A OS=Homo sapiens GN=Rpl7a PE=2 SV=1 |
| Rpl17 protein (Fragment) OS=Homo sapiens GN=Rpl17 PE=2 SV=1 | Ribosomal protein L7A, pseudogene 3 OS=Homo sapiens GN=Rpl7a-ps3 PE=4 SV=1 |
| Rpl17 protein OS=Homo sapiens GN=Rpl17 PE=2 SV=1 | Ribosomal protein L7A, pseudogene 5 OS=Homo sapiens GN=Rpl7a-ps5 PE=4 SV=1 |
| Rpl23a protein (Fragment) OS=Homo sapiens GN=Rpl23a PE=2 SV=1 | Ribosomal protein L8 (Fragment) OS=Homo sapiens PE=4 SV=1 |
| Rpl30 protein OS=Homo sapiens GN=Rpl30 PE=2 SV=1 | Ribosomal protein L9 OS=Homo sapiens GN=Rpl9 PE=2 SV=1 |
| Rpl31 protein OS=Homo sapiens GN=Rpl31 PE=2 SV=1 | Ribosomal protein L9, pseudogene 6 OS=Homo sapiens GN=Rpl9-ps6 PE=4 SV=1 |
| Rpl3l protein (Fragment) OS=Homo sapiens GN=Rpl3l PE=2 SV=1 | Ribosomal protein S14 OS=Homo sapiens GN=rps14 PE=3 SV=1 |
| Rpl7a protein (Fragment) OS=Homo sapiens GN=Rpl7a PE=2 SV=1 | Ribosomal protein S2 (Fragment) OS=Homo sapiens GN=Rps2 PE=2 SV=1 |
| Rps16 protein (Fragment) OS=Homo sapiens GN=Rps16 PE=2 SV=1 | Ribosomal protein S2, pseudogene 6 OS=Homo sapiens GN=Rps2-ps6 PE=3 SV=1 |
| Rps16 protein OS=Homo sapiens GN=Rps16 PE=2 SV=1 | Ribosomal protein S23 OS=Homo sapiens GN=Rps23 PE=2 SV=1 |
| Rps19 protein (Fragment) OS=Homo sapiens GN=Rps19 PE=2 SV=1 | Ribosomal protein S3 (Fragment) OS=Homo sapiens GN=Rps3 PE=2 SV=1 |
| Rps9 protein (Fragment) OS=Homo sapiens GN=Rps9 PE=2 SV=1 | Ribosomal protein S3 OS=Homo sapiens GN=Rps3 PE=1 SV=1 |
| Serine/arginine-rich splicing factor 1 OS=Homo sapiens GN=Srsf1 PE=1 SV=3 | Ribosome-binding protein 1 OS=Homo sapiens GN=Rrbp1 PE=1 SV=1 |
| Serine/arginine-rich splicing factor 2 OS=Homo sapiens GN=Srsf2 PE=1 SV=4 | Ribosome-binding protein 1 OS=Homo sapiens GN=Rrbp1 PE=1 SV=2 |
| Serine/arginine-rich splicing factor 3 OS=Homo sapiens GN=Srsf3 PE=1 SV=1 | RIKEN cDNA 1810009J06 gene OS=Homo sapiens GN=1810009J06Rik PE=2 SV=1 |
| Serine/arginine-rich splicing factor 4 OS=Homo sapiens GN=Srsf4 PE=2 SV=1 | RIKEN cDNA 2210010C04 gene OS=Homo sapiens GN=2210010C04Rik PE=1 SV=1 |
| Serine/arginine-rich splicing factor 5 OS=Homo sapiens GN=Srsf5 PE=1 SV=2 | RIKEN cDNA 9530053A07 gene OS=Homo sapiens GN=9530053A07Rik PE=1 SV=1 |
| Serine/arginine-rich splicing factor 6 OS=Homo sapiens GN=Srsf6 PE=1 SV=1 | RNA and export factor binding protein 2 OS=Homo sapiens GN=Alyref2 PE=2 SV=1 |
| Serine/arginine-rich splicing factor 7 OS=Homo sapiens GN=Srsf7 PE=1 SV=1 | RNA and export factor binding protein 2 OS=Homo sapiens GN=Refbp2 PE=4 SV=1 |
| Serine/arginine-rich-splicing factor 1 (Fragment) OS=Homo sapiens GN=Srsf1 PE=1 SV=1 | RNA binding motif protein, X chromosome, isoform CRA_b OS=Homo sapiens GN=Rbmx PE=1 SV=1 |
| Serine/arginine-rich-splicing factor 1 OS=Homo sapiens GN=Srsf1 PE=1 SV=1 | RNA binding motif protein, X-linked-like-1 OS=Homo sapiens GN=Rbmxl1 PE=1 SV=1 |
| Serine/arginine-rich-splicing factor 11 (Fragment) OS=Homo sapiens GN=Srsf11 PE=1 SV=1 | RNA-binding motif protein, X chromosome (Fragment) OS=Homo sapiens GN=Rbmx PE=1 SV=1 |
| Serine/arginine-rich-splicing factor 11 OS=Homo sapiens GN=Srsf11 PE=1 SV=1 | RNA-binding motif protein, X chromosome OS=Homo sapiens GN=Rbmx PE=1 SV=1 |
| Serine/arginine-rich-splicing factor 4 OS=Homo sapiens GN=Srsf4 PE=1 SV=1 | RNA-binding motif, single-stranded-interacting protein 2 OS=Homo sapiens GN=Rbms2 PE=1 SV=1 |
| Serine/threonine-protein kinase ICK OS=Homo sapiens GN=Ick PE=1 SV=1 | RNA-binding protein 14 OS=Homo sapiens GN=Rbm14 PE=1 SV=1 |
| Serine/threonine-protein kinase ICK OS=Homo sapiens GN=Ick PE=1 SV=2 | RNA-binding protein 3 OS=Homo sapiens GN=Rbm3 PE=1 SV=1 |
| Serine/threonine-protein kinase MAK (Fragment) OS=Homo sapiens GN=Mak PE=3 SV=1 | RNA-binding protein 39 (Fragment) OS=Homo sapiens GN=Rbm39 PE=1 SV=1 |
| Serine/threonine-protein kinase MAK OS=Homo sapiens GN=Mak PE=1 SV=2 | RNA-binding protein 39 OS=Homo sapiens GN=Rbm39 PE=1 SV=1 |
| Serine/threonine-protein kinase MAK OS=Homo sapiens GN=Mak PE=4 SV=1 | RNA-binding protein 39 OS=Homo sapiens GN=Rbm39 PE=1 SV=2 |
| Sfpq protein (Fragment) OS=Homo sapiens GN=Sfpq PE=2 SV=1 | RNA-binding protein EWS OS=Homo sapiens GN=Ewsr1 PE=1 SV=1 |
| Sfrs11 protein (Fragment) OS=Homo sapiens GN=Srsf11 PE=2 SV=1 | RNA-binding protein EWS OS=Homo sapiens GN=Ewsr1 PE=1 SV=2 |
| Sfrs4 protein OS=Homo sapiens GN=Srsf4 PE=2 SV=1 | RNA-binding protein FUS (Fragment) OS=Homo sapiens GN=Fus PE=1 SV=1 |
| Sfrs5 protein OS=Homo sapiens GN=Srsf5 PE=2 SV=1 | Rpl11 protein OS=Homo sapiens GN=Rpl11 PE=2 SV=1 |
| Signal recognition particle receptor subunit beta OS=Homo sapiens GN=Srprb PE=1 SV=1 | Rpl12 protein (Fragment) OS=Homo sapiens GN=Rpl12 PE=2 SV=1 |
| Skeletal muscle alpha-actin mRNA (Fragment) OS=Homo sapiens GN=Acta1 PE=2 SV=1 | Rpl17 protein (Fragment) OS=Homo sapiens GN=Rpl17 PE=2 SV=1 |
| Small nuclear ribonucleoprotein 70 (U1) OS=Homo sapiens GN=Snrnp70 PE=2 SV=1 | Rpl17 protein OS=Homo sapiens GN=Rpl17 PE=2 SV=1 |
| Small nuclear ribonucleoprotein D3 OS=Homo sapiens GN=Snrpd3 PE=2 SV=1 | Rpl23a protein (Fragment) OS=Homo sapiens GN=Rpl23a PE=2 SV=1 |
| Small nuclear ribonucleoprotein Sm D1 OS=Homo sapiens GN=Snrpd1 PE=1 SV=1 | Rpl30 protein OS=Homo sapiens GN=Rpl30 PE=2 SV=1 |
| Small nuclear ribonucleoprotein Sm D3 OS=Homo sapiens GN=Snrpd3 PE=1 SV=1 | Rpl31 protein OS=Homo sapiens GN=Rpl31 PE=2 SV=1 |
| Small nuclear ribonucleoprotein-associated protein B OS=Homo sapiens GN=Snrpb PE=1 SV=1 | Rpl3l protein (Fragment) OS=Homo sapiens GN=Rpl3l PE=2 SV=1 |
| Small nuclear ribonucleoprotein-associated protein N OS=Homo sapiens GN=Snrpn PE=1 SV=1 | Rpl7a protein (Fragment) OS=Homo sapiens GN=Rpl7a PE=2 SV=1 |
| Small nuclear ribonucleoprotein-associated protein OS=Homo sapiens GN=Snrpn PE=3 SV=1 | Rps16 protein (Fragment) OS=Homo sapiens GN=Rps16 PE=2 SV=1 |
| Smooth muscle gamma-actin OS=Homo sapiens PE=3 SV=1 | Rps16 protein OS=Homo sapiens GN=Rps16 PE=2 SV=1 |
| Snrnp70 protein (Fragment) OS=Homo sapiens GN=Snrnp70 PE=2 SV=1 | Rps19 protein (Fragment) OS=Homo sapiens GN=Rps19 PE=2 SV=1 |
| Snrnp70 protein OS=Homo sapiens GN=Snrnp70 PE=2 SV=1 | Rps9 protein (Fragment) OS=Homo sapiens GN=Rps9 PE=2 SV=1 |
| Solute carrier family 25 (Mitochondrial carrier adenine nucleotide translocator), member 31 OS=Homo sapiens GN=Slc25a31 PE=1 SV=1 | Sarcoplasmic/endoplasmic reticulum calcium ATPase 1 (Fragment) OS=Homo sapiens GN=Atp2a1 PE=1 SV=1 |
| Spermatid-specific heat shock protein 70 (Fragment) OS=Homo sapiens GN=Hsc70t PE=3 SV=1 | Sarcoplasmic/endoplasmic reticulum calcium ATPase 1 OS=Homo sapiens GN=Atp2a1 PE=1 SV=1 |
| Splicing factor 3b, subunit 3 OS=Homo sapiens GN=Sf3b3 PE=2 SV=1 | Scr3 OS=Homo sapiens GN=Rbms2 PE=2 SV=1 |
| Splicing factor U2AF 65 kDa subunit OS=Homo sapiens GN=U2af2 PE=1 SV=3 | Secreted frizzled-related protein 5 OS=Homo sapiens GN=Sfrp5 PE=2 SV=1 |
| Splicing factor, proline- and glutamine-rich OS=Homo sapiens GN=Sfpq PE=1 SV=1 | Secreted frizzled-related protein 5 OS=Homo sapiens GN=Sfrp5 PE=4 SV=1 |
| Synaptic functional regulator FMR1 OS=Homo sapiens GN=Fmr1 PE=1 SV=1 | Serine/arginine repetitive matrix protein 1 (Fragment) OS=Homo sapiens GN=Srrm1 PE=1 SV=1 |
| TAF15 RNA polymerase II, TATA box binding protein (TBP)-associated factor OS=Homo sapiens GN=Taf15 PE=1 SV=1 | Serine/arginine-rich splicing factor 1 OS=Homo sapiens GN=Srsf1 PE=1 SV=3 |
| TATA-box-binding protein-associated factor 15 (Fragment) OS=Homo sapiens GN=Taf15 PE=1 SV=1 | Serine/arginine-rich splicing factor 2 OS=Homo sapiens GN=Srsf2 PE=1 SV=4 |
| T-complex protein 1 subunit beta (Fragment) OS=Homo sapiens GN=Cct2 PE=1 SV=1 | Serine/arginine-rich splicing factor 3 OS=Homo sapiens GN=Srsf3 PE=1 SV=1 |
| T-complex protein 1 subunit beta OS=Homo sapiens GN=Cct2 PE=1 SV=1 | Serine/arginine-rich splicing factor 4 OS=Homo sapiens GN=Srsf4 PE=2 SV=1 |
| THO complex subunit 4 OS=Homo sapiens GN=Alyref PE=1 SV=3 | Serine/arginine-rich splicing factor 5 OS=Homo sapiens GN=Srsf5 PE=1 SV=2 |
| Translin-associated factor X-interacting protein 1 OS=Homo sapiens GN=Tsnaxip1 PE=1 SV=1 | Serine/arginine-rich splicing factor 6 OS=Homo sapiens GN=Srsf6 PE=1 SV=1 |
| Translin-associated factor X-interacting protein 1 OS=Homo sapiens GN=Tsnaxip1 PE=1 SV=2 | Serine/arginine-rich splicing factor 7 OS=Homo sapiens GN=Srsf7 PE=1 SV=1 |
| Trypsinogen 5 OS=Homo sapiens GN=trypsinogen PE=3 SV=1 | Serine/arginine-rich-splicing factor 1 (Fragment) OS=Homo sapiens GN=Srsf1 PE=1 SV=1 |
| Tubb2a protein (Fragment) OS=Homo sapiens GN=Tubb2a PE=2 SV=1 | Serine/arginine-rich-splicing factor 1 OS=Homo sapiens GN=Srsf1 PE=1 SV=1 |
| Tubb5 protein (Fragment) OS=Homo sapiens GN=Tubb5 PE=2 SV=1 | Serine/arginine-rich-splicing factor 11 (Fragment) OS=Homo sapiens GN=Srsf11 PE=1 SV=1 |
| Tubulin alpha chain (Fragment) OS=Homo sapiens GN=Tuba1b PE=2 SV=1 | Serine/arginine-rich-splicing factor 11 OS=Homo sapiens GN=Srsf11 PE=1 SV=1 |
| Tubulin alpha chain (Fragment) OS=Homo sapiens GN=Tuba4a PE=1 SV=1 | Serine/arginine-rich-splicing factor 4 OS=Homo sapiens GN=Srsf4 PE=1 SV=1 |
| Tubulin alpha chain OS=Homo sapiens GN=Tuba1c PE=1 SV=1 | Serine/threonine-protein kinase ICK OS=Homo sapiens GN=Ick PE=1 SV=1 |
| Tubulin alpha chain OS=Homo sapiens GN=Tuba1c PE=2 SV=1 | Serine/threonine-protein kinase ICK OS=Homo sapiens GN=Ick PE=1 SV=2 |
| Tubulin alpha-1A chain OS=Homo sapiens GN=Tuba1a PE=1 SV=1 | Serine/threonine-protein kinase MAK (Fragment) OS=Homo sapiens GN=Mak PE=3 SV=1 |
| Tubulin alpha-1B chain OS=Homo sapiens GN=Tuba1b PE=1 SV=2 | Serine/threonine-protein kinase MAK OS=Homo sapiens GN=Mak PE=1 SV=2 |
| Tubulin alpha-3 chain OS=Homo sapiens GN=Tuba3a PE=1 SV=1 | Serine/threonine-protein kinase MAK OS=Homo sapiens GN=Mak PE=4 SV=1 |
| Tubulin alpha-4A chain (Fragment) OS=Homo sapiens GN=Tuba4a PE=1 SV=1 | Serine/threonine-protein phosphatase (Fragment) OS=Homo sapiens GN=Ppp1ca PE=2 SV=1 |
| Tubulin alpha-4A chain OS=Homo sapiens GN=Tuba4a PE=1 SV=1 | Serine/threonine-protein phosphatase OS=Homo sapiens GN=mCG_126872 PE=2 SV=1 |
| Tubulin alpha-8 chain OS=Homo sapiens GN=Tuba8 PE=1 SV=1 | Serine/threonine-protein phosphatase OS=Homo sapiens GN=Ppp1cc PE=1 SV=1 |
| Tubulin beta chain (Fragment) OS=Homo sapiens GN=Tubb4b PE=2 SV=2 | Serine/threonine-protein phosphatase OS=Homo sapiens PE=2 SV=1 |
| Tubulin beta chain OS=Homo sapiens GN=Tubb2b PE=1 SV=1 | Serine/threonine-protein phosphatase PP1-alpha catalytic subunit OS=Homo sapiens GN=Ppp1ca PE=1 SV=1 |
| Tubulin beta chain OS=Homo sapiens GN=Tubb6 PE=2 SV=1 | Serine/threonine-protein phosphatase PP1-beta catalytic subunit OS=Homo sapiens GN=Ppp1cb PE=1 SV=3 |
| Tubulin beta-2A chain OS=Homo sapiens GN=Tubb2a PE=1 SV=1 | Sfpq protein (Fragment) OS=Homo sapiens GN=Sfpq PE=2 SV=1 |
| Tubulin beta-3 chain OS=Homo sapiens GN=Tubb3 PE=1 SV=1 | Sfrs11 protein (Fragment) OS=Homo sapiens GN=Srsf11 PE=2 SV=1 |
| Tubulin beta-4A chain OS=Homo sapiens GN=Tubb4a PE=1 SV=3 | Sfrs4 protein OS=Homo sapiens GN=Srsf4 PE=2 SV=1 |
| Tubulin beta-4B chain OS=Homo sapiens GN=Tubb4b PE=1 SV=1 | Sfrs5 protein OS=Homo sapiens GN=Srsf5 PE=2 SV=1 |
| Tubulin beta-5 chain (Fragment) OS=Homo sapiens GN=Tubb5 PE=1 SV=8 | Signal recognition particle receptor subunit beta OS=Homo sapiens GN=Srprb PE=1 SV=1 |
| Tubulin beta-5 chain OS=Homo sapiens GN=Tubb5 PE=1 SV=1 | Skeletal muscle alpha-actin mRNA (Fragment) OS=Homo sapiens GN=Acta1 PE=2 SV=1 |
| Tubulin beta-6 chain OS=Homo sapiens GN=Tubb6 PE=1 SV=1 | Small nuclear ribonucleoprotein 70 (U1) OS=Homo sapiens GN=Snrnp70 PE=2 SV=1 |
| Type II cytokeratin Kb39 (Fragment) OS=Homo sapiens GN=Krt77 PE=2 SV=1 | Small nuclear ribonucleoprotein D3 OS=Homo sapiens GN=Snrpd3 PE=2 SV=1 |
| Tyrosine-protein kinase Blk OS=Homo sapiens GN=Blk PE=1 SV=4 | Small nuclear ribonucleoprotein Sm D1 OS=Homo sapiens GN=Snrpd1 PE=1 SV=1 |
| Tyrosine-protein kinase Fgr OS=Homo sapiens GN=Fgr PE=1 SV=2 | Small nuclear ribonucleoprotein Sm D3 OS=Homo sapiens GN=Snrpd3 PE=1 SV=1 |
| Tyrosine-protein kinase Fyn OS=Homo sapiens GN=Fyn PE=1 SV=4 | Small nuclear ribonucleoprotein-associated protein B OS=Homo sapiens GN=Snrpb PE=1 SV=1 |
| Tyrosine-protein kinase OS=Homo sapiens GN=Blk PE=2 SV=1 | Small nuclear ribonucleoprotein-associated protein N OS=Homo sapiens GN=Snrpn PE=1 SV=1 |
| Tyrosine-protein kinase OS=Homo sapiens GN=Fgr PE=2 SV=1 | Small nuclear ribonucleoprotein-associated protein OS=Homo sapiens GN=Snrpn PE=3 SV=1 |
| Tyrosine-protein kinase OS=Homo sapiens GN=Fyn PE=1 SV=1 | Smooth muscle gamma-actin OS=Homo sapiens PE=3 SV=1 |
| Tyrosine-protein kinase OS=Homo sapiens GN=Hck PE=1 SV=1 | Snrnp70 protein (Fragment) OS=Homo sapiens GN=Snrnp70 PE=2 SV=1 |
| Tyrosine-protein kinase OS=Homo sapiens GN=Lck PE=1 SV=1 | Snrnp70 protein OS=Homo sapiens GN=Snrnp70 PE=2 SV=1 |
| Tyrosine-protein kinase OS=Homo sapiens GN=Lyn PE=2 SV=1 | Solute carrier family 25 (Mitochondrial carrier adenine nucleotide translocator), member 31 OS=Homo sapiens GN=Slc25a31 PE=1 SV=1 |
| Tyrosine-protein kinase OS=Homo sapiens GN=Yes1 PE=2 SV=1 | Spermatid-specific heat shock protein 70 (Fragment) OS=Homo sapiens GN=Hsc70t PE=3 SV=1 |
| Tyrosine-protein kinase Yes OS=Homo sapiens GN=Yes1 PE=1 SV=3 | Splicing factor 3b, subunit 3 OS=Homo sapiens GN=Sf3b3 PE=2 SV=1 |
| U1 small nuclear ribonucleoprotein 70 kDa (Fragment) OS=Homo sapiens GN=Snrnp70 PE=1 SV=1 | Splicing factor U2AF 26 kDa subunit (Fragment) OS=Homo sapiens GN=U2af1l4 PE=4 SV=1 |
| U1 small nuclear ribonucleoprotein 70 kDa OS=Homo sapiens GN=Snrnp70 PE=1 SV=1 | Splicing factor U2AF 26 kDa subunit OS=Homo sapiens GN=U2af1l4 PE=1 SV=1 |
| U1 small nuclear ribonucleoprotein A (Fragment) OS=Homo sapiens GN=Snrpa PE=1 SV=1 | Splicing factor U2AF 26 kDa subunit OS=Homo sapiens GN=U2af1l4 PE=4 SV=1 |
| U1 small nuclear ribonucleoprotein A OS=Homo sapiens GN=Snrpa PE=1 SV=1 | Splicing factor U2AF 65 kDa subunit OS=Homo sapiens GN=U2af2 PE=1 SV=3 |
| U1 small nuclear ribonucleoprotein A OS=Homo sapiens GN=Snrpa PE=1 SV=3 | Splicing factor, proline- and glutamine-rich OS=Homo sapiens GN=Sfpq PE=1 SV=1 |
| U2 snRNP auxiliary factor large subunit (Fragment) OS=Homo sapiens GN=U2af2 PE=2 SV=1 | SR-related CTD-associated factor 4 OS=Homo sapiens GN=Scaf4 PE=1 SV=1 |
| U2 snRNP auxiliary factor large subunit OS=Homo sapiens GN=U2af2 PE=1 SV=1 | Staphylococcal nuclease domain-containing protein 1 OS=Homo sapiens GN=Snd1 PE=1 SV=1 |
| U2 snRNP auxiliary factor large subunit OS=Homo sapiens GN=U2af2 PE=2 SV=1 | Synaptic functional regulator FMR1 OS=Homo sapiens GN=Fmr1 PE=1 SV=1 |
| U4/U6.U5 tri-snRNP-associated protein 2 OS=Homo sapiens GN=Usp39 PE=1 SV=2 | TAF15 RNA polymerase II, TATA box binding protein (TBP)-associated factor OS=Homo sapiens GN=Taf15 PE=1 SV=1 |
| Ubiquitin associated protein 2-like, isoform CRA_b OS=Homo sapiens GN=Ubap2l PE=1 SV=1 | TATA-box-binding protein-associated factor 15 (Fragment) OS=Homo sapiens GN=Taf15 PE=1 SV=1 |
| Ubiquitin associated protein 2-like, isoform CRA_g OS=Homo sapiens GN=Ubap2l PE=1 SV=1 | THO complex subunit 4 OS=Homo sapiens GN=Alyref PE=1 SV=3 |
| Ubiquitin-associated protein 2-like (Fragment) OS=Homo sapiens GN=Ubap2l PE=1 SV=1 | Tpm1 protein OS=Homo sapiens GN=Tpm1 PE=1 SV=1 |
| Ubiquitin-associated protein 2-like OS=Homo sapiens GN=Ubap2l PE=1 SV=1 | Tpm2 protein OS=Homo sapiens GN=Tpm2 PE=2 SV=1 |
| Vim protein (Fragment) OS=Homo sapiens GN=Vim PE=2 SV=1 | Tpm3 protein OS=Homo sapiens GN=Tpm3 PE=2 SV=1 |
| Vimentin (Fragment) OS=Homo sapiens GN=Vim PE=1 SV=2 | Translin-associated factor X-interacting protein 1 OS=Homo sapiens GN=Tsnaxip1 PE=1 SV=1 |
| Vimentin OS=Homo sapiens GN=Vim PE=1 SV=1 | Translin-associated factor X-interacting protein 1 OS=Homo sapiens GN=Tsnaxip1 PE=1 SV=2 |
| Vimentin OS=Homo sapiens GN=Vim PE=1 SV=3 | tRNA-splicing ligase RtcB homolog OS=Homo sapiens GN=Rtcb PE=1 SV=1 |
| Y box protein 1 OS=Homo sapiens GN=Ybx1 PE=1 SV=1 | tRNA-splicing ligase RtcB homolog OS=Homo sapiens GN=Rtcb PE=2 SV=1 |
| Y box protein 1 OS=Homo sapiens GN=Ybx1 PE=2 SV=1 | Tropomyosin 1, alpha OS=Homo sapiens GN=Tpm1 PE=2 SV=1 |
| Y box protein 2 OS=Homo sapiens GN=Ybx2 PE=1 SV=1 | Tropomyosin 1, alpha, isoform CRA_c OS=Homo sapiens GN=Tpm1 PE=1 SV=1 |
| Y box transcription factor (Fragment) OS=Homo sapiens GN=Ybx1 PE=2 SV=1 | Tropomyosin 1, alpha, isoform CRA_i OS=Homo sapiens GN=Tpm1 PE=1 SV=1 |
| Y-box binding protein (Fragment) OS=Homo sapiens GN=Igf2bp3 PE=2 SV=1 | Tropomyosin 1, alpha, isoform CRA_j OS=Homo sapiens GN=Tpm1 PE=1 SV=1 |
| Y-box-binding protein 2 OS=Homo sapiens GN=Ybx2 PE=1 SV=1 | Tropomyosin 1, alpha, isoform CRA_k OS=Homo sapiens GN=Tpm1 PE=1 SV=1 |
| Y-box-binding protein 3 OS=Homo sapiens GN=Ybx3 PE=1 SV=2 | Tropomyosin 1, alpha, isoform CRA_l OS=Homo sapiens GN=Tpm1 PE=2 SV=1 |
| Ybx2 protein OS=Homo sapiens GN=Ybx2 PE=2 SV=1 | Tropomyosin 3, gamma OS=Homo sapiens GN=Tpm3 PE=2 SV=1 |
|  | Tropomyosin 3, related sequence 7 OS=Homo sapiens GN=Tpm3-rs7 PE=3 SV=1 |
|  | Tropomyosin alpha-1 chain OS=Homo sapiens GN=Tpm1 PE=1 SV=1 |
|  | Tropomyosin alpha-3 chain OS=Homo sapiens GN=Tpm3 PE=1 SV=1 |
|  | Tropomyosin alpha-3 chain OS=Homo sapiens GN=Tpm3 PE=1 SV=3 |
|  | Tropomyosin beta chain (Fragment) OS=Homo sapiens GN=Tpm2 PE=1 SV=8 |
|  | Tropomyosin beta chain OS=Homo sapiens GN=Tpm2 PE=1 SV=1 |
|  | Trypsinogen 5 OS=Homo sapiens GN=trypsinogen PE=3 SV=1 |
|  | Tubb2a protein (Fragment) OS=Homo sapiens GN=Tubb2a PE=2 SV=1 |
|  | Tubb5 protein (Fragment) OS=Homo sapiens GN=Tubb5 PE=2 SV=1 |
|  | Tubulin alpha chain (Fragment) OS=Homo sapiens GN=Tuba1b PE=2 SV=1 |
|  | Tubulin alpha chain (Fragment) OS=Homo sapiens GN=Tuba4a PE=1 SV=1 |
|  | Tubulin alpha chain OS=Homo sapiens GN=Tuba1c PE=1 SV=1 |
|  | Tubulin alpha chain OS=Homo sapiens GN=Tuba1c PE=2 SV=1 |
|  | Tubulin alpha-1A chain OS=Homo sapiens GN=Tuba1a PE=1 SV=1 |
|  | Tubulin alpha-1B chain OS=Homo sapiens GN=Tuba1b PE=1 SV=2 |
|  | Tubulin alpha-3 chain OS=Homo sapiens GN=Tuba3a PE=1 SV=1 |
|  | Tubulin alpha-4A chain OS=Homo sapiens GN=Tuba4a PE=1 SV=1 |
|  | Tubulin alpha-8 chain OS=Homo sapiens GN=Tuba8 PE=1 SV=1 |
|  | Tubulin beta chain OS=Homo sapiens GN=Tubb2b PE=1 SV=1 |
|  | Tubulin beta chain OS=Homo sapiens GN=Tubb6 PE=2 SV=1 |
|  | Tubulin beta-2A chain OS=Homo sapiens GN=Tubb2a PE=1 SV=1 |
|  | Tubulin beta-3 chain OS=Homo sapiens GN=Tubb3 PE=1 SV=1 |
|  | Tubulin beta-4A chain OS=Homo sapiens GN=Tubb4a PE=1 SV=3 |
|  | Tubulin beta-4B chain OS=Homo sapiens GN=Tubb4b PE=1 SV=1 |
|  | Tubulin beta-5 chain OS=Homo sapiens GN=Tubb5 PE=1 SV=1 |
|  | Tubulin beta-6 chain OS=Homo sapiens GN=Tubb6 PE=1 SV=1 |
|  | Type II cytokeratin Kb39 (Fragment) OS=Homo sapiens GN=Krt77 PE=2 SV=1 |
|  | Tyrosine-protein kinase Blk OS=Homo sapiens GN=Blk PE=1 SV=4 |
|  | Tyrosine-protein kinase Fgr OS=Homo sapiens GN=Fgr PE=1 SV=2 |
|  | Tyrosine-protein kinase Fyn OS=Homo sapiens GN=Fyn PE=1 SV=4 |
|  | Tyrosine-protein kinase OS=Homo sapiens GN=Blk PE=2 SV=1 |
|  | Tyrosine-protein kinase OS=Homo sapiens GN=Fgr PE=2 SV=1 |
|  | Tyrosine-protein kinase OS=Homo sapiens GN=Fyn PE=1 SV=1 |
|  | Tyrosine-protein kinase OS=Homo sapiens GN=Hck PE=1 SV=1 |
|  | Tyrosine-protein kinase OS=Homo sapiens GN=Lck PE=1 SV=1 |
|  | Tyrosine-protein kinase OS=Homo sapiens GN=Lyn PE=2 SV=1 |
|  | Tyrosine-protein kinase OS=Homo sapiens GN=Yes1 PE=2 SV=1 |
|  | Tyrosine-protein kinase Yes OS=Homo sapiens GN=Yes1 PE=1 SV=3 |
|  | U1 small nuclear ribonucleoprotein 70 kDa (Fragment) OS=Homo sapiens GN=Snrnp70 PE=1 SV=1 |
|  | U1 small nuclear ribonucleoprotein 70 kDa OS=Homo sapiens GN=Snrnp70 PE=1 SV=1 |
|  | U1 small nuclear ribonucleoprotein A (Fragment) OS=Homo sapiens GN=Snrpa PE=1 SV=1 |
|  | U1 small nuclear ribonucleoprotein A OS=Homo sapiens GN=Snrpa PE=1 SV=3 |
|  | U2 snRNP auxiliary factor large subunit (Fragment) OS=Homo sapiens GN=U2af2 PE=2 SV=1 |
|  | U2 snRNP auxiliary factor large subunit OS=Homo sapiens GN=U2af2 PE=1 SV=1 |
|  | U2 snRNP auxiliary factor large subunit OS=Homo sapiens GN=U2af2 PE=2 SV=1 |
|  | U4/U6.U5 tri-snRNP-associated protein 1 OS=Homo sapiens GN=Sart1 PE=1 SV=1 |
|  | Ubiquitin associated protein 2-like, isoform CRA_b OS=Homo sapiens GN=Ubap2l PE=1 SV=1 |
|  | Ubiquitin associated protein 2-like, isoform CRA_g OS=Homo sapiens GN=Ubap2l PE=1 SV=1 |
|  | Ubiquitin-associated protein 2-like (Fragment) OS=Homo sapiens GN=Ubap2l PE=1 SV=1 |
|  | Ubiquitin-associated protein 2-like OS=Homo sapiens GN=Ubap2l PE=1 SV=1 |
|  | Vim protein (Fragment) OS=Homo sapiens GN=Vim PE=2 SV=1 |
|  | Vimentin (Fragment) OS=Homo sapiens GN=Vim PE=1 SV=2 |
|  | Vimentin OS=Homo sapiens GN=Vim PE=1 SV=1 |
|  | Vimentin OS=Homo sapiens GN=Vim PE=1 SV=3 |
|  | V-type proton ATPase 116 kDa subunit a isoform 1 OS=Homo sapiens GN=Atp6v0a1 PE=1 SV=3 |
|  | V-type proton ATPase subunit a OS=Homo sapiens GN=Atp6v0a1 PE=1 SV=1 |
|  | V-type proton ATPase subunit a OS=Homo sapiens GN=Atp6v0a1 PE=2 SV=1 |
|  | Y box protein 1 OS=Homo sapiens GN=Ybx1 PE=1 SV=1 |
|  | Y box protein 1 OS=Homo sapiens GN=Ybx1 PE=2 SV=1 |
|  | Y box protein 2 OS=Homo sapiens GN=Ybx2 PE=1 SV=1 |
|  | Y box transcription factor (Fragment) OS=Homo sapiens GN=Ybx1 PE=2 SV=1 |
|  | Y-box binding protein (Fragment) OS=Homo sapiens GN=Igf2bp3 PE=2 SV=1 |
|  | Y-box-binding protein 2 OS=Homo sapiens GN=Ybx2 PE=1 SV=1 |
|  | Y-box-binding protein 3 OS=Homo sapiens GN=Ybx3 PE=1 SV=2 |
|  | Ybx2 protein OS=Homo sapiens GN=Ybx2 PE=2 SV=1 |
|  | Zinc finger CCCH domain-containing protein 15 OS=Homo sapiens GN=Zc3h15 PE=1 SV=2 |

**Table S8. The miRNAs differentially expressing between MetS and non-MetS via microarray**

| **miRNA ID** | **AVG ΔC_t_ (Ct(GOI) - Ave Ct (HKG))** | | **2^-ΔC_t_** | | **Fold Difference** | **T-TEST** | **Fold Up- or Down-**  **Regulation** |
| --- | --- | --- | --- | --- | --- | --- | --- |
|  | **A组** | **B组** | **A组** | **B组** | **A组 /B组** | **p value** | **A组 /B组** |
| hsa-miR-20a-3p | 6.94 | 9.01 | 8.2E-03 | 1.9E-03 | **4.19** | **0.0086** | **4.19** |
| hsa-miR-148a-3p | 5.29 | 7.01 | 2.6E-02 | 7.7E-03 | **3.30** | 0.0918 | **3.30** |
| hsa-miR-143-3p | 5.23 | 6.95 | 2.7E-02 | 8.1E-03 | **3.28** | **0.0034** | **3.28** |
| hsa-miR-365a-3p | 4.35 | 5.92 | 4.9E-02 | 1.7E-02 | **2.98** | **0.0042** | **2.98** |
| hsa-miR-22-5p | 6.64 | 8.18 | 1.0E-02 | 3.4E-03 | **2.91** | **0.0093** | **2.91** |
| hsa-miR-15b-5p | 1.81 | 3.27 | 2.8E-01 | 1.0E-01 | **2.75** | 0.1324 | **2.75** |
| hsa-let-7i-5p | 2.11 | 3.57 | 2.3E-01 | 8.4E-02 | **2.74** | **0.0206** | **2.74** |
| hsa-miR-29a-3p | 4.13 | 5.38 | 5.7E-02 | 2.4E-02 | **2.37** | **0.0286** | **2.37** |
| hsa-miR-182-5p | 9.22 | 10.47 | 1.7E-03 | 7.1E-04 | **2.36** | 0.2921 | **2.36** |
| hsa-miR-605 | 10.14 | 11.38 | 8.9E-04 | 3.8E-04 | **2.36** | 0.2121 | **2.36** |
| hsa-miR-424-5p | 9.95 | 11.16 | 1.0E-03 | 4.4E-04 | **2.32** | 0.1187 | **2.32** |
| hsa-miR-29b-2-5p | 7.70 | 8.89 | 4.8E-03 | 2.1E-03 | **2.28** | 0.1761 | **2.28** |
| hsa-miR-99a-5p | 4.65 | 5.82 | 4.0E-02 | 1.8E-02 | **2.26** | **0.0008** | **2.26** |
| hsa-miR-21-5p | -0.30 | 0.83 | 1.2E+00 | 5.6E-01 | **2.19** | 0.2059 | **2.19** |
| hsa-let-7f-5p | 2.41 | 3.50 | 1.9E-01 | 8.9E-02 | **2.12** | 0.1161 | **2.12** |
| hsa-miR-27a-3p | 8.64 | 9.66 | 2.5E-03 | 1.2E-03 | **2.04** | 0.2520 | **2.04** |
| hsa-miR-29a-5p | 7.63 | 8.56 | 5.1E-03 | 2.7E-03 | 1.90 | 0.3493 | 1.90 |
| hsa-miR-17-5p | 5.61 | 6.53 | 2.1E-02 | 1.1E-02 | 1.90 | 0.0648 | 1.90 |
| hsa-miR-20b-5p | 9.08 | 10.00 | 1.8E-03 | 9.7E-04 | 1.90 | 0.1203 | 1.90 |
| hsa-miR-338-3p | 6.54 | 7.43 | 1.1E-02 | 5.8E-03 | 1.85 | 0.1340 | 1.85 |
| hsa-miR-145-5p | 1.84 | 2.72 | 2.8E-01 | 1.5E-01 | 1.84 | 0.3668 | 1.84 |
| hsa-miR-22-3p | -0.41 | 0.45 | 1.3E+00 | 7.3E-01 | 1.82 | **0.0274** | 1.82 |
| hsa-miR-151a-3p | 7.30 | 8.16 | 6.3E-03 | 3.5E-03 | 1.81 | 0.5050 | 1.81 |
| hsa-miR-29c-3p | 3.69 | 4.54 | 7.7E-02 | 4.3E-02 | 1.79 | **0.0087** | 1.79 |
| hsa-miR-144-3p | 1.17 | 2.01 | 4.4E-01 | 2.5E-01 | 1.78 | **0.0218** | 1.78 |
| hsa-miR-34a-5p | 6.53 | 7.35 | 1.1E-02 | 6.1E-03 | 1.77 | 0.1836 | 1.77 |
| hsa-miR-194-5p | 4.15 | 4.97 | 5.6E-02 | 3.2E-02 | 1.76 | 0.0699 | 1.76 |
| hsa-miR-2110 | 6.15 | 6.96 | 1.4E-02 | 8.0E-03 | 1.76 | **0.0494** | 1.76 |
| hsa-miR-204-5p | 5.18 | 5.97 | 2.8E-02 | 1.6E-02 | 1.73 | 0.3343 | 1.73 |
| hsa-miR-190a | 9.93 | 10.67 | 1.0E-03 | 6.1E-04 | 1.68 | 0.4327 | 1.68 |
| hsa-miR-532-5p | 5.54 | 6.23 | 2.2E-02 | 1.3E-02 | 1.62 | 0.2456 | 1.62 |
| hsa-miR-320a | 5.04 | 5.72 | 3.0E-02 | 1.9E-02 | 1.61 | 0.1748 | 1.61 |
| hsa-miR-15a-5p | 1.14 | 1.82 | 4.5E-01 | 2.8E-01 | 1.60 | 0.0612 | 1.60 |
| hsa-miR-27b-3p | 1.62 | 2.28 | 3.3E-01 | 2.1E-01 | 1.58 | 0.1159 | 1.58 |
| hsa-miR-363-3p | 3.38 | 4.02 | 9.6E-02 | 6.2E-02 | 1.56 | 0.1662 | 1.56 |
| hsa-miR-885-5p | 5.02 | 5.65 | 3.1E-02 | 2.0E-02 | 1.55 | 0.4847 | 1.55 |
| hsa-miR-185-5p | 2.78 | 3.42 | 1.5E-01 | 9.4E-02 | 1.55 | 0.3106 | 1.55 |
| hsa-miR-30d-5p | 5.70 | 6.31 | 1.9E-02 | 1.3E-02 | 1.53 | 0.1396 | 1.53 |
| hsa-miR-335-5p | 7.62 | 8.17 | 5.1E-03 | 3.5E-03 | 1.46 | 0.4977 | 1.46 |
| hsa-let-7e-5p | 3.81 | 4.34 | 7.1E-02 | 4.9E-02 | 1.44 | 0.0997 | 1.44 |
| hsa-miR-152 | 3.74 | 4.27 | 7.5E-02 | 5.2E-02 | 1.44 | 0.1591 | 1.44 |
| hsa-miR-33a-5p | 7.14 | 7.67 | 7.1E-03 | 4.9E-03 | 1.44 | 0.3397 | 1.44 |
| hsa-miR-146b-5p | 7.85 | 8.36 | 4.3E-03 | 3.0E-03 | 1.42 | 0.3587 | 1.42 |
| hsa-miR-32-5p | 2.85 | 3.35 | 1.4E-01 | 9.8E-02 | 1.42 | 0.0897 | 1.42 |
| hsa-miR-125a-5p | 2.48 | 2.98 | 1.8E-01 | 1.3E-01 | 1.42 | 0.0715 | 1.42 |
| hsa-miR-125b-5p | 2.35 | 2.84 | 2.0E-01 | 1.4E-01 | 1.41 | 0.2880 | 1.41 |
| hsa-miR-629-5p | 6.18 | 6.67 | 1.4E-02 | 9.8E-03 | 1.41 | 0.2559 | 1.41 |
| hsa-miR-215 | 2.77 | 3.25 | 1.5E-01 | 1.1E-01 | 1.39 | 0.2013 | 1.39 |
| hsa-miR-584-5p | 6.92 | 7.40 | 8.3E-03 | 5.9E-03 | 1.39 | 0.3079 | 1.39 |
| hsa-miR-30a-5p | 5.56 | 6.03 | 2.1E-02 | 1.5E-02 | 1.39 | 0.2537 | 1.39 |
| hsa-miR-324-5p | 5.14 | 5.61 | 2.8E-02 | 2.0E-02 | 1.38 | 0.1809 | 1.38 |
| hsa-miR-30e-5p | 2.38 | 2.84 | 1.9E-01 | 1.4E-01 | 1.38 | 0.2674 | 1.38 |
| hsa-miR-18b-5p | 2.51 | 2.97 | 1.8E-01 | 1.3E-01 | 1.38 | 0.2198 | 1.38 |
| hsa-miR-16-2-3p | 5.92 | 6.36 | 1.7E-02 | 1.2E-02 | 1.36 | 0.3051 | 1.36 |
| hsa-miR-19b-3p | -1.58 | -1.17 | 3.0E+00 | 2.2E+00 | 1.33 | 0.3399 | 1.33 |
| hsa-miR-500a-5p | 8.06 | 8.47 | 3.7E-03 | 2.8E-03 | 1.33 | 0.6996 | 1.33 |
| hsa-miR-132-3p | 5.14 | 5.55 | 2.8E-02 | 2.1E-02 | 1.33 | 0.4365 | 1.33 |
| hsa-miR-320b | 3.10 | 3.51 | 1.2E-01 | 8.8E-02 | 1.32 | **0.0478** | 1.32 |
| hsa-let-7c | 5.20 | 5.59 | 2.7E-02 | 2.1E-02 | 1.31 | 0.3861 | 1.31 |
| hsa-miR-101-3p | 1.98 | 2.37 | 2.5E-01 | 1.9E-01 | 1.31 | **0.0028** | 1.31 |
| hsa-let-7g-5p | 0.05 | 0.43 | 9.7E-01 | 7.4E-01 | 1.31 | 0.1686 | 1.31 |
| hsa-miR-378a-3p | 2.94 | 3.33 | 1.3E-01 | 1.0E-01 | 1.31 | 0.2985 | 1.31 |
| hsa-miR-660-5p | 1.89 | 2.27 | 2.7E-01 | 2.1E-01 | 1.30 | 0.1738 | 1.30 |
| hsa-miR-195-5p | 10.66 | 11.02 | 6.2E-04 | 4.8E-04 | 1.29 | 0.4863 | 1.29 |
| hsa-miR-99b-5p | 4.40 | 4.76 | 4.7E-02 | 3.7E-02 | 1.28 | 0.2330 | 1.28 |
| hsa-miR-28-5p | 4.19 | 4.55 | 5.5E-02 | 4.3E-02 | 1.28 | 0.4741 | 1.28 |
| hsa-miR-146a-5p | 1.46 | 1.82 | 3.6E-01 | 2.8E-01 | 1.28 | 0.2613 | 1.28 |
| hsa-miR-497-5p | 5.65 | 5.99 | 2.0E-02 | 1.6E-02 | 1.27 | 0.3965 | 1.27 |
| hsa-miR-590-5p | 4.20 | 4.52 | 5.5E-02 | 4.3E-02 | 1.25 | 0.3414 | 1.25 |
| hsa-miR-551b-3p | 6.39 | 6.71 | 1.2E-02 | 9.5E-03 | 1.25 | 0.5144 | 1.25 |
| hsa-miR-142-3p | 0.15 | 0.46 | 9.0E-01 | 7.2E-01 | 1.25 | 0.2977 | 1.25 |
| hsa-miR-142-5p | 2.79 | 3.10 | 1.4E-01 | 1.2E-01 | 1.24 | 0.3478 | 1.24 |
| hsa-miR-425-3p | 6.78 | 7.07 | 9.1E-03 | 7.5E-03 | 1.22 | 0.6347 | 1.22 |
| UniSp3 IPC | -5.60 | -5.32 | 4.9E+01 | 4.0E+01 | 1.21 | 0.4675 | 1.21 |
| hsa-miR-15b-3p | 2.96 | 3.23 | 1.3E-01 | 1.1E-01 | 1.20 | 0.3038 | 1.20 |
| hsa-miR-505-3p | 5.14 | 5.40 | 2.8E-02 | 2.4E-02 | 1.20 | 0.4480 | 1.20 |
| hsa-miR-126-3p | -1.44 | -1.19 | 2.7E+00 | 2.3E+00 | 1.19 | 0.2034 | 1.19 |
| hsa-miR-103a-3p | 1.19 | 1.43 | 4.4E-01 | 3.7E-01 | 1.19 | 0.6676 | 1.19 |
| hsa-miR-26a-5p | 0.43 | 0.67 | 7.4E-01 | 6.3E-01 | 1.18 | 0.5656 | 1.18 |
| hsa-miR-10b-5p | 4.88 | 5.12 | 3.4E-02 | 2.9E-02 | 1.18 | 0.5999 | 1.18 |
| UniSp3 IPC | -5.13 | -4.89 | 3.5E+01 | 3.0E+01 | 1.18 | 0.4748 | 1.18 |
| hsa-miR-199a-3p | 2.24 | 2.47 | 2.1E-01 | 1.8E-01 | 1.17 | 0.6150 | 1.17 |
| hsa-miR-130a-3p | 2.64 | 2.84 | 1.6E-01 | 1.4E-01 | 1.15 | 0.4138 | 1.15 |
| hsa-miR-374a-5p | 6.42 | 6.62 | 1.2E-02 | 1.0E-02 | 1.15 | 0.6987 | 1.15 |
| hsa-miR-122-5p | 2.35 | 2.54 | 2.0E-01 | 1.7E-01 | 1.15 | 0.8271 | 1.15 |
| hsa-miR-23a-3p | -0.91 | -0.72 | 1.9E+00 | 1.6E+00 | 1.14 | 0.6517 | 1.14 |
| hsa-miR-107 | 3.17 | 3.36 | 1.1E-01 | 9.8E-02 | 1.14 | 0.7506 | 1.14 |
| hsa-miR-30e-3p | 7.16 | 7.33 | 7.0E-03 | 6.2E-03 | 1.13 | 0.7665 | 1.13 |
| UniSp3 IPC | -5.35 | -5.17 | 4.1E+01 | 3.6E+01 | 1.13 | 0.5900 | 1.13 |
| hsa-miR-192-5p | 2.55 | 2.72 | 1.7E-01 | 1.5E-01 | 1.12 | 0.5848 | 1.12 |
| hsa-miR-18a-3p | 9.04 | 9.21 | 1.9E-03 | 1.7E-03 | 1.12 | 0.7704 | 1.12 |
| hsa-miR-140-3p | 1.36 | 1.50 | 3.9E-01 | 3.5E-01 | 1.10 | 0.6731 | 1.10 |
| hsa-miR-451a | -5.70 | -5.58 | 5.2E+01 | 4.8E+01 | 1.09 | 0.6216 | 1.09 |
| hsa-miR-208a | 11.25 | 11.38 | 4.1E-04 | 3.8E-04 | 1.09 | 0.7577 | 1.09 |
| hsa-miR-186-5p | 2.80 | 2.92 | 1.4E-01 | 1.3E-01 | 1.09 | 0.2967 | 1.09 |
| hsa-miR-148b-3p | 1.57 | 1.68 | 3.4E-01 | 3.1E-01 | 1.08 | 0.5965 | 1.08 |
| hsa-miR-423-5p | 0.91 | 1.01 | 5.3E-01 | 5.0E-01 | 1.07 | 0.3376 | 1.07 |
| hsa-miR-222-3p | 1.20 | 1.29 | 4.4E-01 | 4.1E-01 | 1.07 | 0.6597 | 1.07 |
| hsa-miR-140-5p | 3.93 | 4.02 | 6.5E-02 | 6.2E-02 | 1.06 | 0.7846 | 1.06 |
| UniSp3 IPC | -5.86 | -5.78 | 5.8E+01 | 5.5E+01 | 1.06 | 0.8251 | 1.06 |
| hsa-let-7a-5p | -1.38 | -1.30 | 2.6E+00 | 2.5E+00 | 1.06 | 0.7202 | 1.06 |
| hsa-miR-19a-3p | -1.15 | -1.07 | 2.2E+00 | 2.1E+00 | 1.05 | 0.7323 | 1.05 |
| hsa-miR-10a-5p | 11.15 | 11.22 | 4.4E-04 | 4.2E-04 | 1.05 | 0.8268 | 1.05 |
| hsa-miR-150-5p | 0.37 | 0.44 | 7.7E-01 | 7.4E-01 | 1.05 | 0.8576 | 1.05 |
| hsa-miR-205-5p | 9.95 | 10.02 | 1.0E-03 | 9.6E-04 | 1.04 | 0.9246 | 1.04 |
| hsa-miR-301a-3p | 2.77 | 2.83 | 1.5E-01 | 1.4E-01 | 1.04 | 0.8152 | 1.04 |
| hsa-miR-193b-3p | 4.60 | 4.65 | 4.1E-02 | 4.0E-02 | 1.03 | 0.9152 | 1.03 |
| hsa-miR-199a-5p | 2.65 | 2.69 | 1.6E-01 | 1.6E-01 | 1.03 | 0.9476 | 1.03 |
| UniSp3 IPC | -5.64 | -5.61 | 5.0E+01 | 4.9E+01 | 1.02 | 0.9383 | 1.02 |
| UniSp3 IPC | -5.32 | -5.30 | 4.0E+01 | 3.9E+01 | 1.02 | 0.9412 | 1.02 |
| hsa-miR-95 | 10.59 | 10.61 | 6.5E-04 | 6.4E-04 | 1.02 | 0.9787 | 1.02 |
| hsa-miR-29b-3p | 6.61 | 6.63 | 1.0E-02 | 1.0E-02 | 1.01 | 0.8975 | 1.01 |
| UniSp6 | 0.58 | 0.59 | 6.7E-01 | 6.6E-01 | 1.01 | 0.9782 | 1.01 |
| hsa-miR-16-5p | -5.97 | -5.97 | 6.3E+01 | 6.3E+01 | 1.00 | 0.9797 | 1.00 |
| hsa-miR-144-5p | 1.76 | 1.76 | 2.9E-01 | 3.0E-01 | 1.00 | 0.9992 | -1.00 |
| hsa-miR-18a-5p | 2.55 | 2.54 | 1.7E-01 | 1.7E-01 | 1.00 | 0.9960 | -1.00 |
| hsa-miR-106b-5p | 1.21 | 1.20 | 4.3E-01 | 4.4E-01 | 0.99 | 0.9711 | -1.01 |
| hsa-miR-361-3p | 11.36 | 11.35 | 3.8E-04 | 3.8E-04 | 0.99 | 0.9609 | -1.01 |
| hsa-miR-30b-5p | 1.04 | 1.02 | 4.8E-01 | 4.9E-01 | 0.98 | 0.9488 | -1.02 |
| hsa-miR-155-5p | 6.11 | 6.07 | 1.4E-02 | 1.5E-02 | 0.97 | 0.9052 | -1.03 |
| hsa-miR-20a-5p | -1.47 | -1.52 | 2.8E+00 | 2.9E+00 | 0.97 | 0.7670 | -1.03 |
| hsa-miR-652-3p | 0.96 | 0.91 | 5.1E-01 | 5.3E-01 | 0.96 | 0.8352 | -1.04 |
| hsa-let-7i-3p | 7.37 | 7.32 | 6.0E-03 | 6.3E-03 | 0.96 | 0.8593 | -1.04 |
| hsa-miR-92a-3p | -2.68 | -2.74 | 6.4E+00 | 6.7E+00 | 0.96 | 0.8347 | -1.04 |
| hsa-miR-24-3p | -1.07 | -1.15 | 2.1E+00 | 2.2E+00 | 0.95 | 0.7728 | -1.05 |
| hsa-miR-30c-5p | 0.45 | 0.38 | 7.3E-01 | 7.7E-01 | 0.95 | 0.7600 | -1.05 |
| hsa-miR-486-5p | -2.25 | -2.34 | 4.8E+00 | 5.1E+00 | 0.94 | 0.7922 | -1.06 |
| UniSp2 | 11.47 | 11.38 | 3.5E-04 | 3.8E-04 | 0.94 | 0.8070 | -1.07 |
| UniSp4 | 11.47 | 11.38 | 3.5E-04 | 3.8E-04 | 0.94 | 0.8070 | -1.07 |
| UniSp5 | 11.47 | 11.38 | 3.5E-04 | 3.8E-04 | 0.94 | 0.8070 | -1.07 |
| cel-miR-39-3p | 11.47 | 11.38 | 3.5E-04 | 3.8E-04 | 0.94 | 0.8070 | -1.07 |
| Blank (H2O) | 11.47 | 11.38 | 3.5E-04 | 3.8E-04 | 0.94 | 0.8070 | -1.07 |
| Blank (H2O) | 11.47 | 11.38 | 3.5E-04 | 3.8E-04 | 0.94 | 0.8070 | -1.07 |
| hsa-miR-296-5p | 6.58 | 6.48 | 1.0E-02 | 1.1E-02 | 0.94 | 0.7649 | -1.07 |
| hsa-miR-93-5p | -0.91 | -1.01 | 1.9E+00 | 2.0E+00 | 0.93 | 0.3376 | -1.07 |
| hsa-miR-223-5p | 6.76 | 6.66 | 9.2E-03 | 9.9E-03 | 0.93 | 0.8896 | -1.07 |
| hsa-miR-130b-3p | 6.24 | 6.14 | 1.3E-02 | 1.4E-02 | 0.93 | 0.7898 | -1.07 |
| hsa-miR-128 | 3.49 | 3.39 | 8.9E-02 | 9.5E-02 | 0.93 | 0.7081 | -1.07 |
| hsa-miR-25-3p | -1.26 | -1.36 | 2.4E+00 | 2.6E+00 | 0.93 | 0.4338 | -1.08 |
| hsa-miR-574-3p | 3.84 | 3.70 | 7.0E-02 | 7.7E-02 | 0.91 | 0.6382 | -1.10 |
| hsa-miR-181a-5p | 1.82 | 1.67 | 2.8E-01 | 3.1E-01 | 0.90 | 0.5724 | -1.11 |
| hsa-miR-532-3p | 4.54 | 4.39 | 4.3E-02 | 4.8E-02 | 0.90 | 0.5337 | -1.11 |
| hsa-miR-26b-5p | 1.46 | 1.30 | 3.6E-01 | 4.1E-01 | 0.90 | 0.4524 | -1.12 |
| hsa-miR-331-3p | 7.47 | 7.30 | 5.6E-03 | 6.3E-03 | 0.89 | 0.5889 | -1.12 |
| hsa-miR-139-5p | 3.05 | 2.88 | 1.2E-01 | 1.4E-01 | 0.89 | 0.2471 | -1.12 |
| hsa-miR-106a-5p | -1.51 | -1.69 | 2.9E+00 | 3.2E+00 | 0.88 | 0.2657 | -1.13 |
| hsa-miR-191-5p | 1.44 | 1.26 | 3.7E-01 | 4.2E-01 | 0.88 | 0.6116 | -1.13 |
| hsa-miR-484 | 0.69 | 0.51 | 6.2E-01 | 7.0E-01 | 0.88 | 0.2004 | -1.13 |
| hsa-miR-374b-5p | 3.47 | 3.29 | 9.0E-02 | 1.0E-01 | 0.88 | 0.6103 | -1.13 |
| hsa-miR-326 | 4.64 | 4.46 | 4.0E-02 | 4.6E-02 | 0.88 | 0.6780 | -1.14 |
| hsa-miR-421 | 8.14 | 7.95 | 3.5E-03 | 4.0E-03 | 0.88 | 0.7933 | -1.14 |
| hsa-miR-210 | 4.10 | 3.91 | 5.8E-02 | 6.7E-02 | 0.87 | 0.5082 | -1.15 |
| hsa-let-7b-5p | 1.19 | 0.98 | 4.4E-01 | 5.1E-01 | 0.87 | 0.5853 | -1.15 |
| hsa-miR-221-3p | -0.35 | -0.59 | 1.3E+00 | 1.5E+00 | 0.85 | 0.5154 | -1.18 |
| hsa-miR-425-5p | 0.60 | 0.36 | 6.6E-01 | 7.8E-01 | 0.85 | 0.1378 | -1.18 |
| hsa-let-7d-3p | 0.52 | 0.26 | 7.0E-01 | 8.3E-01 | 0.84 | 0.3033 | -1.19 |
| hsa-miR-106b-3p | 6.13 | 5.87 | 1.4E-02 | 1.7E-02 | 0.84 | 0.2387 | -1.20 |
| hsa-miR-342-3p | 2.53 | 2.27 | 1.7E-01 | 2.1E-01 | 0.84 | 0.3787 | -1.20 |
| hsa-miR-23b-3p | 0.82 | 0.54 | 5.7E-01 | 6.9E-01 | 0.82 | 0.3776 | -1.22 |
| hsa-miR-502-3p | 5.52 | 5.24 | 2.2E-02 | 2.6E-02 | 0.82 | 0.4484 | -1.22 |
| hsa-miR-92b-3p | 10.68 | 10.39 | 6.1E-04 | 7.4E-04 | 0.82 | 0.4327 | -1.22 |
| hsa-miR-197-3p | 2.98 | 2.67 | 1.3E-01 | 1.6E-01 | 0.80 | 0.4187 | -1.24 |
| hsa-let-7d-5p | 1.97 | 1.65 | 2.6E-01 | 3.2E-01 | 0.80 | 0.3561 | -1.25 |
| hsa-miR-28-3p | 3.75 | 3.41 | 7.5E-02 | 9.4E-02 | 0.79 | 0.1767 | -1.26 |
| hsa-miR-151a-5p | 0.54 | 0.15 | 6.9E-01 | 9.0E-01 | 0.76 | 0.1746 | -1.31 |
| hsa-miR-339-3p | 5.39 | 4.97 | 2.4E-02 | 3.2E-02 | 0.75 | 0.3057 | -1.34 |
| hsa-miR-133b | 6.43 | 5.98 | 1.2E-02 | 1.6E-02 | 0.73 | 0.4982 | -1.37 |
| hsa-miR-223-3p | -2.69 | -3.15 | 6.4E+00 | 8.9E+00 | 0.72 | 0.1943 | -1.38 |
| hsa-miR-324-3p | 2.66 | 2.19 | 1.6E-01 | 2.2E-01 | 0.72 | 0.0553 | -1.39 |
| hsa-miR-423-3p | 2.13 | 1.65 | 2.3E-01 | 3.2E-01 | 0.72 | 0.1178 | -1.39 |
| hsa-let-7b-3p | 5.36 | 4.81 | 2.4E-02 | 3.6E-02 | 0.69 | 0.0572 | -1.46 |
| hsa-miR-200c-3p | 7.66 | 7.11 | 4.9E-03 | 7.3E-03 | 0.68 | 0.7130 | -1.47 |
| hsa-miR-766-3p | 3.39 | 2.80 | 9.6E-02 | 1.4E-01 | 0.67 | 0.3111 | -1.50 |
| hsa-miR-328 | 4.11 | 3.42 | 5.8E-02 | 9.3E-02 | 0.62 | 0.0593 | -1.61 |
| hsa-miR-1 | 9.43 | 8.73 | 1.4E-03 | 2.4E-03 | 0.61 | 0.4474 | -1.63 |
| hsa-miR-93-3p | 4.89 | 4.17 | 3.4E-02 | 5.5E-02 | 0.61 | **0.0006** | -1.64 |
| hsa-miR-376a-3p | 10.95 | 10.16 | 5.1E-04 | 8.7E-04 | 0.58 | 0.0614 | -1.73 |
| hsa-miR-501-3p | 7.51 | 6.71 | 5.5E-03 | 9.6E-03 | 0.57 | 0.4418 | -1.75 |
| hsa-miR-339-5p | 4.64 | 3.74 | 4.0E-02 | 7.5E-02 | 0.54 | **0.0427** | -1.87 |
| hsa-miR-409-3p | 5.79 | 4.86 | 1.8E-02 | 3.5E-02 | 0.52 | 0.1761 | -1.91 |
| hsa-miR-141-3p | 8.12 | 7.16 | 3.6E-03 | 7.0E-03 | 0.52 | 0.2212 | -1.94 |
| hsa-miR-133a | 6.69 | 5.62 | 9.7E-03 | 2.0E-02 | **0.48** | 0.1396 | **-2.10** |
| hsa-miR-543 | 7.83 | 6.62 | 4.4E-03 | 1.0E-02 | **0.43** | 0.2857 | **-2.31** |
| hsa-miR-136-5p | 6.11 | 4.88 | 1.5E-02 | 3.4E-02 | **0.43** | 0.1052 | **-2.34** |
| hsa-miR-200a-3p | 10.55 | 9.19 | 6.7E-04 | 1.7E-03 | **0.39** | 0.1114 | **-2.56** |
| hsa-miR-375 | 6.29 | 4.77 | 1.3E-02 | 3.7E-02 | **0.35** | **0.0390** | **-2.86** |
| hsa-miR-154-5p | 6.84 | 5.29 | 8.7E-03 | 2.6E-02 | **0.34** | **0.0100** | **-2.93** |
| hsa-miR-127-3p | 7.05 | 5.20 | 7.5E-03 | 2.7E-02 | **0.28** | 0.0622 | **-3.60** |
| hsa-miR-495-3p | 8.26 | 6.31 | 3.3E-03 | 1.3E-02 | **0.26** | 0.1476 | **-3.84** |
| hsa-miR-485-3p | 8.15 | 6.08 | 3.5E-03 | 1.5E-02 | **0.24** | 0.0709 | **-4.20** |
| hsa-miR-346 | 10.82 | 8.31 | 5.5E-04 | 3.2E-03 | **0.18** | **0.0113** | **-5.71** |
| hsa-miR-382-5p | 7.89 | 4.82 | 4.2E-03 | 3.5E-02 | **0.12** | **0.0318** | **-8.43** |

**Table S9. Overlapping of miRanda, circBank, TargetScan and microarray**

| **miRanda** | **circBank** | **Targetscan** | **microarray** |
| --- | --- | --- | --- |
| hsa-miR-1 | hsa-miR-1252-5p | hsa-let-7a-3p | hsa-let-7a-5p |
| hsa-miR-101 | hsa-miR-1253 | hsa-let-7b-3p | hsa-let-7b-3p |
| hsa-miR-1297 | hsa-miR-1273h-3p | hsa-let-7c-3p | hsa-let-7b-5p |
| hsa-miR-139-5p | hsa-miR-1285-5p | hsa-let-7f-1-3p | hsa-let-7c |
| hsa-miR-143 | hsa-miR-135a-5p | hsa-let-7f-2-3p | hsa-let-7d-3p |
| hsa-miR-154 | hsa-miR-135b-5p | hsa-let-7f-2-3p | hsa-let-7d-5p |
| hsa-miR-15a | hsa-miR-143-3p | hsa-miR-101-3p.1 | hsa-let-7e-5p |
| hsa-miR-15b | hsa-miR-15b-3p | hsa-miR-101-3p.2 | hsa-let-7f-5p |
| hsa-miR-16 | hsa-miR-200c-5p | hsa-miR-103a-3p | hsa-let-7g-5p |
| hsa-miR-195 | hsa-miR-204-3p | hsa-miR-105-5p | hsa-let-7i-3p |
| hsa-miR-196a | hsa-miR-2115-3p | hsa-miR-106a-3p | hsa-let-7i-5p |
| hsa-miR-196b | hsa-miR-211-5p | hsa-miR-106a-3p | hsa-miR-1 |
| hsa-miR-19a | hsa-miR-27a-3p | hsa-miR-106a-3p | hsa-miR-101-3p |
| hsa-miR-19b | hsa-miR-27b-3p | hsa-miR-107 | hsa-miR-103a-3p |
| hsa-miR-203 | hsa-miR-298 | hsa-miR-10a-3p | hsa-miR-106a-5p |
| hsa-miR-206 | hsa-miR-302b-5p | hsa-miR-1178-5p | hsa-miR-106b-3p |
| hsa-miR-21 | hsa-miR-302d-5p | hsa-miR-1179 | hsa-miR-106b-5p |
| hsa-miR-218 | hsa-miR-3140-3p | hsa-miR-1182 | hsa-miR-107 |
| hsa-miR-23a | hsa-miR-3152-5p | hsa-miR-1183 | hsa-miR-10a-5p |
| hsa-miR-23b | hsa-miR-3173-3p | hsa-miR-1184 | hsa-miR-10b-5p |
| hsa-miR-26a | hsa-miR-324-3p | hsa-miR-1185-1-3p | hsa-miR-122-5p |
| hsa-miR-26b | hsa-miR-335-3p | hsa-miR-1185-1-3p | hsa-miR-125a-5p |
| hsa-miR-27a | hsa-miR-34b-3p | hsa-miR-1185-2-3p | hsa-miR-125b-5p |
| hsa-miR-27b | hsa-miR-361-5p | hsa-miR-1185-2-3p | hsa-miR-126-3p |
| hsa-miR-300 | hsa-miR-3916 | hsa-miR-1193 | hsa-miR-127-3p |
| hsa-miR-30a | hsa-miR-4428 | hsa-miR-1205 | hsa-miR-128 |
| hsa-miR-30b | hsa-miR-4434 | hsa-miR-1206 | hsa-miR-130a-3p |
| hsa-miR-30c | hsa-miR-4461 | hsa-miR-1206 | hsa-miR-130b-3p |
| hsa-miR-30d | hsa-miR-4495 | hsa-miR-1207-3p | hsa-miR-132-3p |
| hsa-miR-30e | hsa-miR-4659a-5p | hsa-miR-1226-3p | hsa-miR-133a |
| hsa-miR-31 | hsa-miR-4659b-5p | hsa-miR-1233-5p | hsa-miR-133b |
| hsa-miR-320a | hsa-miR-4674 | hsa-miR-1236-5p | hsa-miR-136-5p |
| hsa-miR-320b | hsa-miR-4677-5p | hsa-miR-1238-5p | hsa-miR-139-5p |
| hsa-miR-320c | hsa-miR-4698 | hsa-miR-1243 | hsa-miR-140-3p |
| hsa-miR-320d | hsa-miR-4708-3p | hsa-miR-1245a | hsa-miR-140-5p |
| hsa-miR-324-5p | hsa-miR-4743-3p | hsa-miR-1245a | hsa-miR-141-3p |
| hsa-miR-335 | hsa-miR-4753-3p | hsa-miR-124-5p | hsa-miR-142-3p |
| hsa-miR-340 | hsa-miR-4760-3p | hsa-miR-1250-3p | hsa-miR-142-5p |
| hsa-miR-381 | hsa-miR-4778-3p | hsa-miR-1250-3p | hsa-miR-143-3p |
| hsa-miR-410 | hsa-miR-4782-5p | hsa-miR-1250-3p | hsa-miR-144-3p |
| hsa-miR-421 | hsa-miR-495-3p | hsa-miR-1251-3p | hsa-miR-144-5p |
| hsa-miR-424 | hsa-miR-5011-3p | hsa-miR-1252-3p | hsa-miR-145-5p |
| hsa-miR-455-5p | hsa-miR-511-5p | hsa-miR-1255b-2-3p | hsa-miR-146a-5p |
| hsa-miR-486-5p | hsa-miR-515-5p | hsa-miR-125b-2-3p | hsa-miR-146b-5p |
| hsa-miR-488 | hsa-miR-518a-5p | hsa-miR-1261 | hsa-miR-148a-3p |
| hsa-miR-494 | hsa-miR-518c-5p | hsa-miR-1263 | hsa-miR-148b-3p |
| hsa-miR-495 | hsa-miR-519d-5p | hsa-miR-1271-3p | hsa-miR-150-5p |
| hsa-miR-496 | hsa-miR-519e-5p | hsa-miR-1271-5p | hsa-miR-151a-3p |
| hsa-miR-497 | hsa-miR-527 | hsa-miR-1275 | hsa-miR-151a-5p |
| hsa-miR-503 | hsa-miR-548aw | hsa-miR-127-5p | hsa-miR-152 |
| hsa-miR-505 | hsa-miR-5582-3p | hsa-miR-1283 | hsa-miR-154-5p |
| hsa-miR-539 | hsa-miR-580-3p | hsa-miR-128-3p | hsa-miR-155-5p |
| hsa-miR-590-3p | hsa-miR-583 | hsa-miR-1292-5p | hsa-miR-15a-5p |
| hsa-miR-590-5p | hsa-miR-606 | hsa-miR-1292-5p | hsa-miR-15b-3p |
| hsa-miR-613 | hsa-miR-6074 | hsa-miR-1297 | hsa-miR-15b-5p |
| hsa-miR-653 | hsa-miR-6505-3p | hsa-miR-1298-3p | hsa-miR-16-2-3p |
| hsa-miR-7 | hsa-miR-6515-3p | hsa-miR-1298-3p | hsa-miR-16-5p |
| hsa-miR-9 | hsa-miR-653-3p | hsa-miR-1303 | hsa-miR-17-5p |
|  | hsa-miR-6750-5p | hsa-miR-1305 | hsa-miR-181a-5p |
|  | hsa-miR-6764-3p | hsa-miR-1305 | hsa-miR-182-5p |
|  | hsa-miR-6780a-3p | hsa-miR-130a-5p | hsa-miR-185-5p |
|  | hsa-miR-6817-5p | hsa-miR-130a-5p | hsa-miR-186-5p |
|  | hsa-miR-6822-5p | hsa-miR-1321 | hsa-miR-18a-3p |
|  | hsa-miR-6834-5p | hsa-miR-1324 | hsa-miR-18a-5p |
|  | hsa-miR-6852-3p | hsa-miR-1343-3p | hsa-miR-18b-5p |
|  | hsa-miR-6859-5p | hsa-miR-134-5p | hsa-miR-190a |
|  | hsa-miR-6866-3p | hsa-miR-138-1-3p | hsa-miR-191-5p |
|  | hsa-miR-6868-3p | hsa-miR-139-5p | hsa-miR-192-5p |
|  | hsa-miR-6868-5p | hsa-miR-1-3p | hsa-miR-193b-3p |
|  | hsa-miR-6893-3p | hsa-miR-142-3p.2 | hsa-miR-194-5p |
|  | hsa-miR-7111-3p | hsa-miR-143-3p | hsa-miR-195-5p |
|  | hsa-miR-7-1-3p | hsa-miR-144-3p | hsa-miR-197-3p |
|  | hsa-miR-7160-5p | hsa-miR-144-3p | hsa-miR-199a-3p |
|  | hsa-miR-7-2-3p | hsa-miR-144-5p | hsa-miR-199a-5p |
|  | hsa-miR-744-3p | hsa-miR-145-5p | hsa-miR-19a-3p |
|  | hsa-miR-760 | hsa-miR-1468-3p | hsa-miR-19b-3p |
|  | hsa-miR-876-3p | hsa-miR-148a-5p | hsa-miR-200a-3p |
|  | hsa-miR-93-3p | hsa-miR-148a-5p | hsa-miR-200c-3p |
|  | hsa-miR-942-5p | hsa-miR-149-3p | hsa-miR-204-5p |
|  |  | hsa-miR-150-3p | hsa-miR-205-5p |
|  |  | hsa-miR-153-5p | hsa-miR-208a |
|  |  | hsa-miR-153-5p | hsa-miR-20a-3p |
|  |  | hsa-miR-153-5p | hsa-miR-20a-5p |
|  |  | hsa-miR-153-5p | hsa-miR-20b-5p |
|  |  | hsa-miR-155-5p | hsa-miR-210 |
|  |  | hsa-miR-15a-5p | hsa-miR-2110 |
|  |  | hsa-miR-15a-5p | hsa-miR-215 |
|  |  | hsa-miR-15b-3p | hsa-miR-21-5p |
|  |  | hsa-miR-15b-5p | hsa-miR-221-3p |
|  |  | hsa-miR-15b-5p | hsa-miR-222-3p |
|  |  | hsa-miR-16-1-3p | hsa-miR-223-3p |
|  |  | hsa-miR-16-2-3p | hsa-miR-223-5p |
|  |  | hsa-miR-16-2-3p | hsa-miR-22-3p |
|  |  | hsa-miR-16-5p | hsa-miR-22-5p |
|  |  | hsa-miR-16-5p | hsa-miR-23a-3p |
|  |  | hsa-miR-17-3p | hsa-miR-23b-3p |
|  |  | hsa-miR-17-3p | hsa-miR-24-3p |
|  |  | hsa-miR-181a-2-3p | hsa-miR-25-3p |
|  |  | hsa-miR-181a-3p | hsa-miR-26a-5p |
|  |  | hsa-miR-181b-2-3p | hsa-miR-26b-5p |
|  |  | hsa-miR-181b-3p | hsa-miR-27a-3p |
|  |  | hsa-miR-1825 | hsa-miR-27b-3p |
|  |  | hsa-miR-183-3p | hsa-miR-28-3p |
|  |  | hsa-miR-183-5p.2 | hsa-miR-28-5p |
|  |  | hsa-miR-186-5p | hsa-miR-296-5p |
|  |  | hsa-miR-188-5p | hsa-miR-29a-3p |
|  |  | hsa-miR-1910-5p | hsa-miR-29a-5p |
|  |  | hsa-miR-1911-3p | hsa-miR-29b-2-5p |
|  |  | hsa-miR-191-3p | hsa-miR-29b-3p |
|  |  | hsa-miR-1914-5p | hsa-miR-29c-3p |
|  |  | hsa-miR-1915-5p | hsa-miR-301a-3p |
|  |  | hsa-miR-194-5p | hsa-miR-30a-5p |
|  |  | hsa-miR-195-3p | hsa-miR-30b-5p |
|  |  | hsa-miR-195-3p | hsa-miR-30c-5p |
|  |  | hsa-miR-195-5p | hsa-miR-30d-5p |
|  |  | hsa-miR-195-5p | hsa-miR-30e-3p |
|  |  | hsa-miR-196a-5p | hsa-miR-30e-5p |
|  |  | hsa-miR-196b-5p | hsa-miR-320a |
|  |  | hsa-miR-199a-3p | hsa-miR-320b |
|  |  | hsa-miR-199a-5p | hsa-miR-324-3p |
|  |  | hsa-miR-199b-3p | hsa-miR-324-5p |
|  |  | hsa-miR-199b-5p | hsa-miR-32-5p |
|  |  | hsa-miR-19a-3p | hsa-miR-326 |
|  |  | hsa-miR-19b-3p | hsa-miR-328 |
|  |  | hsa-miR-200a-5p | hsa-miR-331-3p |
|  |  | hsa-miR-200a-5p | hsa-miR-335-5p |
|  |  | hsa-miR-200b-5p | hsa-miR-338-3p |
|  |  | hsa-miR-200b-5p | hsa-miR-339-3p |
|  |  | hsa-miR-200c-5p | hsa-miR-339-5p |
|  |  | hsa-miR-203a-3p.1 | hsa-miR-33a-5p |
|  |  | hsa-miR-203a-3p.1 | hsa-miR-342-3p |
|  |  | hsa-miR-2053 | hsa-miR-346 |
|  |  | hsa-miR-205-5p | hsa-miR-34a-5p |
|  |  | hsa-miR-206 | hsa-miR-361-3p |
|  |  | hsa-miR-208a-5p | hsa-miR-363-3p |
|  |  | hsa-miR-208b-5p | hsa-miR-365a-3p |
|  |  | hsa-miR-2113 | hsa-miR-374a-5p |
|  |  | hsa-miR-211-3p | hsa-miR-374b-5p |
|  |  | hsa-miR-2114-5p | hsa-miR-375 |
|  |  | hsa-miR-2115-3p | hsa-miR-376a-3p |
|  |  | hsa-miR-2115-5p | hsa-miR-378a-3p |
|  |  | hsa-miR-212-5p | hsa-miR-382-5p |
|  |  | hsa-miR-214-3p | hsa-miR-409-3p |
|  |  | hsa-miR-21-5p | hsa-miR-421 |
|  |  | hsa-miR-21-5p | hsa-miR-423-3p |
|  |  | hsa-miR-216a-3p | hsa-miR-423-5p |
|  |  | hsa-miR-218-2-3p | hsa-miR-424-5p |
|  |  | hsa-miR-218-5p | hsa-miR-425-3p |
|  |  | hsa-miR-222-5p | hsa-miR-425-5p |
|  |  | hsa-miR-224-5p | hsa-miR-451a |
|  |  | hsa-miR-224-5p | hsa-miR-484 |
|  |  | hsa-miR-2276-3p | hsa-miR-485-3p |
|  |  | hsa-miR-2276-3p | hsa-miR-486-5p |
|  |  | hsa-miR-2355-3p | hsa-miR-495-3p |
|  |  | hsa-miR-2355-5p | hsa-miR-497-5p |
|  |  | hsa-miR-2392 | hsa-miR-500a-5p |
|  |  | hsa-miR-2392 | hsa-miR-501-3p |
|  |  | hsa-miR-23a-3p | hsa-miR-502-3p |
|  |  | hsa-miR-23a-3p | hsa-miR-505-3p |
|  |  | hsa-miR-23b-3p | hsa-miR-532-3p |
|  |  | hsa-miR-23b-3p | hsa-miR-532-5p |
|  |  | hsa-miR-23c | hsa-miR-543 |
|  |  | hsa-miR-23c | hsa-miR-551b-3p |
|  |  | hsa-miR-24-1-5p | hsa-miR-574-3p |
|  |  | hsa-miR-24-2-5p | hsa-miR-584-5p |
|  |  | hsa-miR-26a-5p | hsa-miR-590-5p |
|  |  | hsa-miR-26b-3p | hsa-miR-605 |
|  |  | hsa-miR-26b-5p | hsa-miR-629-5p |
|  |  | hsa-miR-27a-3p | hsa-miR-652-3p |
|  |  | hsa-miR-27a-3p | hsa-miR-660-5p |
|  |  | hsa-miR-27b-3p | hsa-miR-766-3p |
|  |  | hsa-miR-27b-3p | hsa-miR-885-5p |
|  |  | hsa-miR-2909 | hsa-miR-92a-3p |
|  |  | hsa-miR-298 | hsa-miR-92b-3p |
|  |  | hsa-miR-29a-5p | hsa-miR-93-3p |
|  |  | hsa-miR-300 | hsa-miR-93-5p |
|  |  | hsa-miR-301a-5p | hsa-miR-95 |
|  |  | hsa-miR-301a-5p | hsa-miR-99a-5p |
|  |  | hsa-miR-302b-5p | hsa-miR-99b-5p |
|  |  | hsa-miR-302c-5p |  |
|  |  | hsa-miR-302d-5p |  |
|  |  | hsa-miR-3065-5p |  |
|  |  | hsa-miR-30a-5p |  |
|  |  | hsa-miR-30b-5p |  |
|  |  | hsa-miR-30c-5p |  |
|  |  | hsa-miR-30d-5p |  |
|  |  | hsa-miR-30e-5p |  |
|  |  | hsa-miR-3115 |  |
|  |  | hsa-miR-3118 |  |
|  |  | hsa-miR-3120-3p |  |
|  |  | hsa-miR-3121-3p |  |
|  |  | hsa-miR-3123 |  |
|  |  | hsa-miR-3125 |  |
|  |  | hsa-miR-3126-3p |  |
|  |  | hsa-miR-3129-3p |  |
|  |  | hsa-miR-3129-3p |  |
|  |  | hsa-miR-3129-3p |  |
|  |  | hsa-miR-3129-5p |  |
|  |  | hsa-miR-3130-3p |  |
|  |  | hsa-miR-3130-3p |  |
|  |  | hsa-miR-3136-5p |  |
|  |  | hsa-miR-3140-5p |  |
|  |  | hsa-miR-3143 |  |
|  |  | hsa-miR-3143 |  |
|  |  | hsa-miR-3145-3p |  |
|  |  | hsa-miR-3148 |  |
|  |  | hsa-miR-3150a-5p |  |
|  |  | hsa-miR-3150b-5p |  |
|  |  | hsa-miR-3152-5p |  |
|  |  | hsa-miR-3158-5p |  |
|  |  | hsa-miR-31-5p |  |
|  |  | hsa-miR-3161 |  |
|  |  | hsa-miR-3163 |  |
|  |  | hsa-miR-3163 |  |
|  |  | hsa-miR-3163 |  |
|  |  | hsa-miR-3163 |  |
|  |  | hsa-miR-3164 |  |
|  |  | hsa-miR-3164 |  |
|  |  | hsa-miR-3166 |  |
|  |  | hsa-miR-3168 |  |
|  |  | hsa-miR-3171 |  |
|  |  | hsa-miR-3171 |  |
|  |  | hsa-miR-3185 |  |
|  |  | hsa-miR-3200-3p |  |
|  |  | hsa-miR-3201 |  |
|  |  | hsa-miR-3201 |  |
|  |  | hsa-miR-3201 |  |
|  |  | hsa-miR-323a-3p |  |
|  |  | hsa-miR-323a-3p |  |
|  |  | hsa-miR-324-5p |  |
|  |  | hsa-miR-330-3p |  |
|  |  | hsa-miR-330-3p |  |
|  |  | hsa-miR-331-3p |  |
|  |  | hsa-miR-335-3p |  |
|  |  | hsa-miR-335-5p |  |
|  |  | hsa-miR-337-3p |  |
|  |  | hsa-miR-338-5p |  |
|  |  | hsa-miR-33a-3p |  |
|  |  | hsa-miR-340-5p |  |
|  |  | hsa-miR-340-5p |  |
|  |  | hsa-miR-340-5p |  |
|  |  | hsa-miR-345-5p |  |
|  |  | hsa-miR-34a-5p |  |
|  |  | hsa-miR-34c-3p |  |
|  |  | hsa-miR-34c-3p |  |
|  |  | hsa-miR-34c-5p |  |
|  |  | hsa-miR-3529-3p |  |
|  |  | hsa-miR-3529-3p |  |
|  |  | hsa-miR-3591-5p |  |
|  |  | hsa-miR-3606-3p |  |
|  |  | hsa-miR-3609 |  |
|  |  | hsa-miR-3613-3p |  |
|  |  | hsa-miR-3613-5p |  |
|  |  | hsa-miR-3614-5p |  |
|  |  | hsa-miR-361-5p |  |
|  |  | hsa-miR-3616-3p |  |
|  |  | hsa-miR-3616-5p |  |
|  |  | hsa-miR-3616-5p |  |
|  |  | hsa-miR-3618 |  |
|  |  | hsa-miR-3619-5p |  |
|  |  | hsa-miR-3646 |  |
|  |  | hsa-miR-3649 |  |
|  |  | hsa-miR-3662 |  |
|  |  | hsa-miR-3665 |  |
|  |  | hsa-miR-3668 |  |
|  |  | hsa-miR-3671 |  |
|  |  | hsa-miR-3671 |  |
|  |  | hsa-miR-3671 |  |
|  |  | hsa-miR-3671 |  |
|  |  | hsa-miR-3679-3p |  |
|  |  | hsa-miR-3681-3p |  |
|  |  | hsa-miR-3688-3p |  |
|  |  | hsa-miR-3688-5p |  |
|  |  | hsa-miR-3689a-5p |  |
|  |  | hsa-miR-3689b-5p |  |
|  |  | hsa-miR-3689e |  |
|  |  | hsa-miR-3689f |  |
|  |  | hsa-miR-369-5p |  |
|  |  | hsa-miR-370-5p |  |
|  |  | hsa-miR-374a-3p |  |
|  |  | hsa-miR-374a-3p |  |
|  |  | hsa-miR-374a-3p |  |
|  |  | hsa-miR-374b-3p |  |
|  |  | hsa-miR-374c-5p |  |
|  |  | hsa-miR-376a-2-5p |  |
|  |  | hsa-miR-376a-2-5p |  |
|  |  | hsa-miR-376a-5p |  |
|  |  | hsa-miR-379-3p |  |
|  |  | hsa-miR-380-3p |  |
|  |  | hsa-miR-380-5p |  |
|  |  | hsa-miR-380-5p |  |
|  |  | hsa-miR-381-3p |  |
|  |  | hsa-miR-382-3p |  |
|  |  | hsa-miR-383-3p |  |
|  |  | hsa-miR-3908 |  |
|  |  | hsa-miR-3909 |  |
|  |  | hsa-miR-3913-3p |  |
|  |  | hsa-miR-3914 |  |
|  |  | hsa-miR-3916 |  |
|  |  | hsa-miR-3920 |  |
|  |  | hsa-miR-3922-5p |  |
|  |  | hsa-miR-3924 |  |
|  |  | hsa-miR-3925-5p |  |
|  |  | hsa-miR-3935 |  |
|  |  | hsa-miR-3940-5p |  |
|  |  | hsa-miR-3942-5p |  |
|  |  | hsa-miR-3945 |  |
|  |  | hsa-miR-3973 |  |
|  |  | hsa-miR-3973 |  |
|  |  | hsa-miR-3973 |  |
|  |  | hsa-miR-3976 |  |
|  |  | hsa-miR-410-3p |  |
|  |  | hsa-miR-411-3p |  |
|  |  | hsa-miR-412-3p |  |
|  |  | hsa-miR-421 |  |
|  |  | hsa-miR-424-5p |  |
|  |  | hsa-miR-424-5p |  |
|  |  | hsa-miR-4255 |  |
|  |  | hsa-miR-4270 |  |
|  |  | hsa-miR-4272 |  |
|  |  | hsa-miR-4272 |  |
|  |  | hsa-miR-4274 |  |
|  |  | hsa-miR-4276 |  |
|  |  | hsa-miR-4277 |  |
|  |  | hsa-miR-4282 |  |
|  |  | hsa-miR-4282 |  |
|  |  | hsa-miR-4282 |  |
|  |  | hsa-miR-4282 |  |
|  |  | hsa-miR-4282 |  |
|  |  | hsa-miR-4299 |  |
|  |  | hsa-miR-4328 |  |
|  |  | hsa-miR-4328 |  |
|  |  | hsa-miR-4418 |  |
|  |  | hsa-miR-4419a |  |
|  |  | hsa-miR-4420 |  |
|  |  | hsa-miR-4422 |  |
|  |  | hsa-miR-4426 |  |
|  |  | hsa-miR-4426 |  |
|  |  | hsa-miR-4428 |  |
|  |  | hsa-miR-4431 |  |
|  |  | hsa-miR-4436b-5p |  |
|  |  | hsa-miR-4438 |  |
|  |  | hsa-miR-4438 |  |
|  |  | hsa-miR-4439 |  |
|  |  | hsa-miR-4441 |  |
|  |  | hsa-miR-4446-5p |  |
|  |  | hsa-miR-4446-5p |  |
|  |  | hsa-miR-4452 |  |
|  |  | hsa-miR-4456 |  |
|  |  | hsa-miR-4457 |  |
|  |  | hsa-miR-4465 |  |
|  |  | hsa-miR-4470 |  |
|  |  | hsa-miR-4471 |  |
|  |  | hsa-miR-4476 |  |
|  |  | hsa-miR-4477a |  |
|  |  | hsa-miR-4477a |  |
|  |  | hsa-miR-4477a |  |
|  |  | hsa-miR-4477a |  |
|  |  | hsa-miR-4477b |  |
|  |  | hsa-miR-4477b |  |
|  |  | hsa-miR-4495 |  |
|  |  | hsa-miR-4495 |  |
|  |  | hsa-miR-4496 |  |
|  |  | hsa-miR-449a |  |
|  |  | hsa-miR-449b-5p |  |
|  |  | hsa-miR-4501 |  |
|  |  | hsa-miR-4502 |  |
|  |  | hsa-miR-4503 |  |
|  |  | hsa-miR-4507 |  |
|  |  | hsa-miR-450b-5p |  |
|  |  | hsa-miR-4510 |  |
|  |  | hsa-miR-4517 |  |
|  |  | hsa-miR-4517 |  |
|  |  | hsa-miR-4517 |  |
|  |  | hsa-miR-4520-2-3p |  |
|  |  | hsa-miR-4520-3p |  |
|  |  | hsa-miR-4524a-5p |  |
|  |  | hsa-miR-4524a-5p |  |
|  |  | hsa-miR-4524b-3p |  |
|  |  | hsa-miR-4524b-5p |  |
|  |  | hsa-miR-4524b-5p |  |
|  |  | hsa-miR-4525 |  |
|  |  | hsa-miR-452-5p |  |
|  |  | hsa-miR-452-5p |  |
|  |  | hsa-miR-4529-3p |  |
|  |  | hsa-miR-4532 |  |
|  |  | hsa-miR-4533 |  |
|  |  | hsa-miR-4536-3p |  |
|  |  | hsa-miR-455-3p.1 |  |
|  |  | hsa-miR-455-5p |  |
|  |  | hsa-miR-4639-3p |  |
|  |  | hsa-miR-4639-3p |  |
|  |  | hsa-miR-4639-5p |  |
|  |  | hsa-miR-4645-3p |  |
|  |  | hsa-miR-4646-3p |  |
|  |  | hsa-miR-4647 |  |
|  |  | hsa-miR-4647 |  |
|  |  | hsa-miR-4652-3p |  |
|  |  | hsa-miR-4658 |  |
|  |  | hsa-miR-4659a-3p |  |
|  |  | hsa-miR-4659a-3p |  |
|  |  | hsa-miR-4659b-3p |  |
|  |  | hsa-miR-4659b-3p |  |
|  |  | hsa-miR-4662b |  |
|  |  | hsa-miR-4662b |  |
|  |  | hsa-miR-4665-5p |  |
|  |  | hsa-miR-4666a-5p |  |
|  |  | hsa-miR-4666b |  |
|  |  | hsa-miR-4667-5p |  |
|  |  | hsa-miR-4667-5p |  |
|  |  | hsa-miR-4668-3p |  |
|  |  | hsa-miR-4676-3p |  |
|  |  | hsa-miR-4676-3p |  |
|  |  | hsa-miR-4686 |  |
|  |  | hsa-miR-4686 |  |
|  |  | hsa-miR-4691-5p |  |
|  |  | hsa-miR-4691-5p |  |
|  |  | hsa-miR-4693-5p |  |
|  |  | hsa-miR-4698 |  |
|  |  | hsa-miR-4698 |  |
|  |  | hsa-miR-4699-3p |  |
|  |  | hsa-miR-4699-3p |  |
|  |  | hsa-miR-4699-3p |  |
|  |  | hsa-miR-4700-5p |  |
|  |  | hsa-miR-4700-5p |  |
|  |  | hsa-miR-4703-5p |  |
|  |  | hsa-miR-4709-5p |  |
|  |  | hsa-miR-4709-5p |  |
|  |  | hsa-miR-4711-3p |  |
|  |  | hsa-miR-4712-3p |  |
|  |  | hsa-miR-4713-5p |  |
|  |  | hsa-miR-4719 |  |
|  |  | hsa-miR-4722-3p |  |
|  |  | hsa-miR-4723-5p |  |
|  |  | hsa-miR-4724-3p |  |
|  |  | hsa-miR-4728-3p |  |
|  |  | hsa-miR-4728-5p |  |
|  |  | hsa-miR-4731-3p |  |
|  |  | hsa-miR-4732-3p |  |
|  |  | hsa-miR-4733-3p |  |
|  |  | hsa-miR-4739 |  |
|  |  | hsa-miR-4755-5p |  |
|  |  | hsa-miR-4756-5p |  |
|  |  | hsa-miR-4758-5p |  |
|  |  | hsa-miR-4760-3p |  |
|  |  | hsa-miR-4762-5p |  |
|  |  | hsa-miR-4762-5p |  |
|  |  | hsa-miR-4762-5p |  |
|  |  | hsa-miR-4763-5p |  |
|  |  | hsa-miR-4764-3p |  |
|  |  | hsa-miR-4765 |  |
|  |  | hsa-miR-4766-3p |  |
|  |  | hsa-miR-4772-3p |  |
|  |  | hsa-miR-4773 |  |
|  |  | hsa-miR-4774-3p |  |
|  |  | hsa-miR-4777-5p |  |
|  |  | hsa-miR-4778-3p |  |
|  |  | hsa-miR-4778-5p |  |
|  |  | hsa-miR-4780 |  |
|  |  | hsa-miR-4781-3p |  |
|  |  | hsa-miR-4786-3p |  |
|  |  | hsa-miR-4789-3p |  |
|  |  | hsa-miR-4789-5p |  |
|  |  | hsa-miR-4791 |  |
|  |  | hsa-miR-4793-3p |  |
|  |  | hsa-miR-4793-3p |  |
|  |  | hsa-miR-4793-5p |  |
|  |  | hsa-miR-4795-5p |  |
|  |  | hsa-miR-4795-5p |  |
|  |  | hsa-miR-4799-5p |  |
|  |  | hsa-miR-4799-5p |  |
|  |  | hsa-miR-4799-5p |  |
|  |  | hsa-miR-4801 |  |
|  |  | hsa-miR-486-3p |  |
|  |  | hsa-miR-486-5p |  |
|  |  | hsa-miR-488-3p |  |
|  |  | hsa-miR-489-3p |  |
|  |  | hsa-miR-489-3p |  |
|  |  | hsa-miR-491-3p |  |
|  |  | hsa-miR-491-3p |  |
|  |  | hsa-miR-494-3p |  |
|  |  | hsa-miR-495-3p |  |
|  |  | hsa-miR-495-3p |  |
|  |  | hsa-miR-497-5p |  |
|  |  | hsa-miR-497-5p |  |
|  |  | hsa-miR-498 |  |
|  |  | hsa-miR-499a-3p |  |
|  |  | hsa-miR-499b-3p |  |
|  |  | hsa-miR-5002-3p |  |
|  |  | hsa-miR-5002-5p |  |
|  |  | hsa-miR-5003-3p |  |
|  |  | hsa-miR-5003-3p |  |
|  |  | hsa-miR-5003-5p |  |
|  |  | hsa-miR-5006-3p |  |
|  |  | hsa-miR-5008-3p |  |
|  |  | hsa-miR-5010-5p |  |
|  |  | hsa-miR-5011-3p |  |
|  |  | hsa-miR-503-3p |  |
|  |  | hsa-miR-503-5p |  |
|  |  | hsa-miR-505-3p.1 |  |
|  |  | hsa-miR-506-5p |  |
|  |  | hsa-miR-509-3-5p |  |
|  |  | hsa-miR-5094 |  |
|  |  | hsa-miR-5094 |  |
|  |  | hsa-miR-5095 |  |
|  |  | hsa-miR-509-5p |  |
|  |  | hsa-miR-511-5p |  |
|  |  | hsa-miR-513a-3p |  |
|  |  | hsa-miR-513a-5p |  |
|  |  | hsa-miR-513b-3p |  |
|  |  | hsa-miR-513b-3p |  |
|  |  | hsa-miR-513b-3p |  |
|  |  | hsa-miR-513b-5p |  |
|  |  | hsa-miR-513c-3p |  |
|  |  | hsa-miR-514a-3p |  |
|  |  | hsa-miR-514a-5p |  |
|  |  | hsa-miR-514b-3p |  |
|  |  | hsa-miR-516a-3p |  |
|  |  | hsa-miR-516b-3p |  |
|  |  | hsa-miR-517a-3p |  |
|  |  | hsa-miR-517a-3p |  |
|  |  | hsa-miR-517b-3p |  |
|  |  | hsa-miR-517b-3p |  |
|  |  | hsa-miR-517c-3p |  |
|  |  | hsa-miR-517c-3p |  |
|  |  | hsa-miR-5187-3p |  |
|  |  | hsa-miR-518a-5p |  |
|  |  | hsa-miR-518d-5p |  |
|  |  | hsa-miR-518e-5p |  |
|  |  | hsa-miR-518f-5p |  |
|  |  | hsa-miR-5190 |  |
|  |  | hsa-miR-5190 |  |
|  |  | hsa-miR-5195-3p |  |
|  |  | hsa-miR-5197-5p |  |
|  |  | hsa-miR-519a-5p |  |
|  |  | hsa-miR-519b-5p |  |
|  |  | hsa-miR-519c-5p |  |
|  |  | hsa-miR-519d-5p |  |
|  |  | hsa-miR-520c-5p |  |
|  |  | hsa-miR-520d-5p |  |
|  |  | hsa-miR-520g-3p |  |
|  |  | hsa-miR-520g-3p |  |
|  |  | hsa-miR-520g-5p |  |
|  |  | hsa-miR-520h |  |
|  |  | hsa-miR-520h |  |
|  |  | hsa-miR-522-5p |  |
|  |  | hsa-miR-523-5p |  |
|  |  | hsa-miR-524-5p |  |
|  |  | hsa-miR-526a |  |
|  |  | hsa-miR-527 |  |
|  |  | hsa-miR-539-5p |  |
|  |  | hsa-miR-539-5p |  |
|  |  | hsa-miR-539-5p |  |
|  |  | hsa-miR-541-5p |  |
|  |  | hsa-miR-543 |  |
|  |  | hsa-miR-543 |  |
|  |  | hsa-miR-545-3p |  |
|  |  | hsa-miR-545-5p |  |
|  |  | hsa-miR-545-5p |  |
|  |  | hsa-miR-548a-3p |  |
|  |  | hsa-miR-548a-3p |  |
|  |  | hsa-miR-548a-5p |  |
|  |  | hsa-miR-548a-5p |  |
|  |  | hsa-miR-548ab |  |
|  |  | hsa-miR-548ab |  |
|  |  | hsa-miR-548ac |  |
|  |  | hsa-miR-548ad-5p |  |
|  |  | hsa-miR-548ad-5p |  |
|  |  | hsa-miR-548ae-3p |  |
|  |  | hsa-miR-548ae-3p |  |
|  |  | hsa-miR-548ae-5p |  |
|  |  | hsa-miR-548ae-5p |  |
|  |  | hsa-miR-548ag |  |
|  |  | hsa-miR-548ah-3p |  |
|  |  | hsa-miR-548ah-3p |  |
|  |  | hsa-miR-548ah-5p |  |
|  |  | hsa-miR-548ai |  |
|  |  | hsa-miR-548aj-3p |  |
|  |  | hsa-miR-548aj-3p |  |
|  |  | hsa-miR-548aj-5p |  |
|  |  | hsa-miR-548ak |  |
|  |  | hsa-miR-548ak |  |
|  |  | hsa-miR-548am-3p |  |
|  |  | hsa-miR-548am-3p |  |
|  |  | hsa-miR-548am-5p |  |
|  |  | hsa-miR-548am-5p |  |
|  |  | hsa-miR-548ao-5p |  |
|  |  | hsa-miR-548ap-5p |  |
|  |  | hsa-miR-548ap-5p |  |
|  |  | hsa-miR-548aq-3p |  |
|  |  | hsa-miR-548aq-3p |  |
|  |  | hsa-miR-548aq-5p |  |
|  |  | hsa-miR-548aq-5p |  |
|  |  | hsa-miR-548ar-3p |  |
|  |  | hsa-miR-548ar-3p |  |
|  |  | hsa-miR-548ar-5p |  |
|  |  | hsa-miR-548ar-5p |  |
|  |  | hsa-miR-548as-5p |  |
|  |  | hsa-miR-548as-5p |  |
|  |  | hsa-miR-548au-3p |  |
|  |  | hsa-miR-548au-5p |  |
|  |  | hsa-miR-548au-5p |  |
|  |  | hsa-miR-548av-3p |  |
|  |  | hsa-miR-548av-3p |  |
|  |  | hsa-miR-548av-5p |  |
|  |  | hsa-miR-548aw |  |
|  |  | hsa-miR-548ax |  |
|  |  | hsa-miR-548ay-5p |  |
|  |  | hsa-miR-548ay-5p |  |
|  |  | hsa-miR-548az-3p |  |
|  |  | hsa-miR-548az-3p |  |
|  |  | hsa-miR-548az-5p |  |
|  |  | hsa-miR-548b-5p |  |
|  |  | hsa-miR-548b-5p |  |
|  |  | hsa-miR-548ba |  |
|  |  | hsa-miR-548bb-3p |  |
|  |  | hsa-miR-548bb-5p |  |
|  |  | hsa-miR-548bb-5p |  |
|  |  | hsa-miR-548c-3p |  |
|  |  | hsa-miR-548c-3p |  |
|  |  | hsa-miR-548c-3p |  |
|  |  | hsa-miR-548c-3p |  |
|  |  | hsa-miR-548c-5p |  |
|  |  | hsa-miR-548c-5p |  |
|  |  | hsa-miR-548d-3p |  |
|  |  | hsa-miR-548d-5p |  |
|  |  | hsa-miR-548d-5p |  |
|  |  | hsa-miR-548e-3p |  |
|  |  | hsa-miR-548e-3p |  |
|  |  | hsa-miR-548f-3p |  |
|  |  | hsa-miR-548f-3p |  |
|  |  | hsa-miR-548f-5p |  |
|  |  | hsa-miR-548g-3p |  |
|  |  | hsa-miR-548g-3p |  |
|  |  | hsa-miR-548g-5p |  |
|  |  | hsa-miR-548h-3p |  |
|  |  | hsa-miR-548h-5p |  |
|  |  | hsa-miR-548h-5p |  |
|  |  | hsa-miR-548i |  |
|  |  | hsa-miR-548i |  |
|  |  | hsa-miR-548j-3p |  |
|  |  | hsa-miR-548j-3p |  |
|  |  | hsa-miR-548j-5p |  |
|  |  | hsa-miR-548j-5p |  |
|  |  | hsa-miR-548k |  |
|  |  | hsa-miR-548l |  |
|  |  | hsa-miR-548l |  |
|  |  | hsa-miR-548m |  |
|  |  | hsa-miR-548n |  |
|  |  | hsa-miR-548o-5p |  |
|  |  | hsa-miR-548o-5p |  |
|  |  | hsa-miR-548p |  |
|  |  | hsa-miR-548q |  |
|  |  | hsa-miR-548t-5p |  |
|  |  | hsa-miR-548w |  |
|  |  | hsa-miR-548w |  |
|  |  | hsa-miR-548x-3p |  |
|  |  | hsa-miR-548x-3p |  |
|  |  | hsa-miR-548x-5p |  |
|  |  | hsa-miR-548y |  |
|  |  | hsa-miR-548y |  |
|  |  | hsa-miR-548z |  |
|  |  | hsa-miR-549a |  |
|  |  | hsa-miR-550a-3-5p |  |
|  |  | hsa-miR-550a-3p |  |
|  |  | hsa-miR-550a-5p |  |
|  |  | hsa-miR-550b-2-5p |  |
|  |  | hsa-miR-551b-5p |  |
|  |  | hsa-miR-551b-5p |  |
|  |  | hsa-miR-551b-5p |  |
|  |  | hsa-miR-5579-3p |  |
|  |  | hsa-miR-5580-3p |  |
|  |  | hsa-miR-5582-3p |  |
|  |  | hsa-miR-5583-3p |  |
|  |  | hsa-miR-5583-3p |  |
|  |  | hsa-miR-5583-3p |  |
|  |  | hsa-miR-5583-5p |  |
|  |  | hsa-miR-5583-5p |  |
|  |  | hsa-miR-5583-5p |  |
|  |  | hsa-miR-5584-5p |  |
|  |  | hsa-miR-5585-5p |  |
|  |  | hsa-miR-559 |  |
|  |  | hsa-miR-559 |  |
|  |  | hsa-miR-563 |  |
|  |  | hsa-miR-563 |  |
|  |  | hsa-miR-567 |  |
|  |  | hsa-miR-5680 |  |
|  |  | hsa-miR-5680 |  |
|  |  | hsa-miR-5680 |  |
|  |  | hsa-miR-5680 |  |
|  |  | hsa-miR-5681a |  |
|  |  | hsa-miR-5681b |  |
|  |  | hsa-miR-5688 |  |
|  |  | hsa-miR-5688 |  |
|  |  | hsa-miR-5690 |  |
|  |  | hsa-miR-5691 |  |
|  |  | hsa-miR-5692a |  |
|  |  | hsa-miR-5692b |  |
|  |  | hsa-miR-5692b |  |
|  |  | hsa-miR-5692c |  |
|  |  | hsa-miR-5692c |  |
|  |  | hsa-miR-5693 |  |
|  |  | hsa-miR-5696 |  |
|  |  | hsa-miR-5697 |  |
|  |  | hsa-miR-5698 |  |
|  |  | hsa-miR-5700 |  |
|  |  | hsa-miR-570-5p |  |
|  |  | hsa-miR-573 |  |
|  |  | hsa-miR-573 |  |
|  |  | hsa-miR-577 |  |
|  |  | hsa-miR-579-3p |  |
|  |  | hsa-miR-582-5p |  |
|  |  | hsa-miR-584-5p |  |
|  |  | hsa-miR-586 |  |
|  |  | hsa-miR-586 |  |
|  |  | hsa-miR-589-3p |  |
|  |  | hsa-miR-589-3p |  |
|  |  | hsa-miR-590-3p |  |
|  |  | hsa-miR-590-3p |  |
|  |  | hsa-miR-590-5p |  |
|  |  | hsa-miR-590-5p |  |
|  |  | hsa-miR-595 |  |
|  |  | hsa-miR-599 |  |
|  |  | hsa-miR-600 |  |
|  |  | hsa-miR-601 |  |
|  |  | hsa-miR-605-5p |  |
|  |  | hsa-miR-605-5p |  |
|  |  | hsa-miR-606 |  |
|  |  | hsa-miR-607 |  |
|  |  | hsa-miR-607 |  |
|  |  | hsa-miR-607 |  |
|  |  | hsa-miR-607 |  |
|  |  | hsa-miR-6074 |  |
|  |  | hsa-miR-6074 |  |
|  |  | hsa-miR-6077 |  |
|  |  | hsa-miR-6083 |  |
|  |  | hsa-miR-6083 |  |
|  |  | hsa-miR-6124 |  |
|  |  | hsa-miR-6127 |  |
|  |  | hsa-miR-6129 |  |
|  |  | hsa-miR-613 |  |
|  |  | hsa-miR-6130 |  |
|  |  | hsa-miR-6133 |  |
|  |  | hsa-miR-6165 |  |
|  |  | hsa-miR-621 |  |
|  |  | hsa-miR-623 |  |
|  |  | hsa-miR-624-3p |  |
|  |  | hsa-miR-624-5p |  |
|  |  | hsa-miR-625-3p |  |
|  |  | hsa-miR-625-5p |  |
|  |  | hsa-miR-627-3p |  |
|  |  | hsa-miR-629-3p |  |
|  |  | hsa-miR-630 |  |
|  |  | hsa-miR-636 |  |
|  |  | hsa-miR-637 |  |
|  |  | hsa-miR-637 |  |
|  |  | hsa-miR-640 |  |
|  |  | hsa-miR-642b-5p |  |
|  |  | hsa-miR-643 |  |
|  |  | hsa-miR-646 |  |
|  |  | hsa-miR-646 |  |
|  |  | hsa-miR-648 |  |
|  |  | hsa-miR-6500-3p |  |
|  |  | hsa-miR-6501-3p |  |
|  |  | hsa-miR-6504-3p |  |
|  |  | hsa-miR-6508-5p |  |
|  |  | hsa-miR-651-3p |  |
|  |  | hsa-miR-651-3p |  |
|  |  | hsa-miR-6514-3p |  |
|  |  | hsa-miR-653-5p |  |
|  |  | hsa-miR-655-3p |  |
|  |  | hsa-miR-656-3p |  |
|  |  | hsa-miR-656-3p |  |
|  |  | hsa-miR-656-3p |  |
|  |  | hsa-miR-659-3p |  |
|  |  | hsa-miR-659-5p |  |
|  |  | hsa-miR-664b-3p |  |
|  |  | hsa-miR-6715b-3p |  |
|  |  | hsa-miR-6716-5p |  |
|  |  | hsa-miR-6727-3p |  |
|  |  | hsa-miR-6730-5p |  |
|  |  | hsa-miR-6737-3p |  |
|  |  | hsa-miR-6742-3p |  |
|  |  | hsa-miR-6749-3p |  |
|  |  | hsa-miR-6750-3p |  |
|  |  | hsa-miR-6753-5p |  |
|  |  | hsa-miR-6754-3p |  |
|  |  | hsa-miR-6754-5p |  |
|  |  | hsa-miR-6758-5p |  |
|  |  | hsa-miR-6759-3p |  |
|  |  | hsa-miR-6759-3p |  |
|  |  | hsa-miR-676-3p |  |
|  |  | hsa-miR-6764-3p |  |
|  |  | hsa-miR-6778-5p |  |
|  |  | hsa-miR-6780b-3p |  |
|  |  | hsa-miR-6782-3p |  |
|  |  | hsa-miR-6783-3p |  |
|  |  | hsa-miR-6785-5p |  |
|  |  | hsa-miR-6790-5p |  |
|  |  | hsa-miR-6792-3p |  |
|  |  | hsa-miR-6792-3p |  |
|  |  | hsa-miR-6792-5p |  |
|  |  | hsa-miR-6792-5p |  |
|  |  | hsa-miR-6794-3p |  |
|  |  | hsa-miR-6794-3p |  |
|  |  | hsa-miR-6796-3p |  |
|  |  | hsa-miR-6801-3p |  |
|  |  | hsa-miR-6805-3p |  |
|  |  | hsa-miR-6810-3p |  |
|  |  | hsa-miR-6815-5p |  |
|  |  | hsa-miR-6816-3p |  |
|  |  | hsa-miR-6820-3p |  |
|  |  | hsa-miR-6820-3p |  |
|  |  | hsa-miR-6824-3p |  |
|  |  | hsa-miR-6830-3p |  |
|  |  | hsa-miR-6830-5p |  |
|  |  | hsa-miR-6835-3p |  |
|  |  | hsa-miR-6835-3p |  |
|  |  | hsa-miR-6837-3p |  |
|  |  | hsa-miR-6837-3p |  |
|  |  | hsa-miR-6838-5p |  |
|  |  | hsa-miR-6838-5p |  |
|  |  | hsa-miR-6852-3p |  |
|  |  | hsa-miR-6852-5p |  |
|  |  | hsa-miR-6853-3p |  |
|  |  | hsa-miR-6853-3p |  |
|  |  | hsa-miR-6856-5p |  |
|  |  | hsa-miR-6858-3p |  |
|  |  | hsa-miR-6859-5p |  |
|  |  | hsa-miR-6865-5p |  |
|  |  | hsa-miR-6866-3p |  |
|  |  | hsa-miR-6867-3p |  |
|  |  | hsa-miR-6870-5p |  |
|  |  | hsa-miR-6873-5p |  |
|  |  | hsa-miR-6875-3p |  |
|  |  | hsa-miR-6875-3p |  |
|  |  | hsa-miR-6876-5p |  |
|  |  | hsa-miR-6882-5p |  |
|  |  | hsa-miR-6883-3p |  |
|  |  | hsa-miR-6883-5p |  |
|  |  | hsa-miR-6894-3p |  |
|  |  | hsa-miR-7111-5p |  |
|  |  | hsa-miR-7-1-3p |  |
|  |  | hsa-miR-7-1-3p |  |
|  |  | hsa-miR-7-1-3p |  |
|  |  | hsa-miR-7-1-3p |  |
|  |  | hsa-miR-7151-3p |  |
|  |  | hsa-miR-7151-5p |  |
|  |  | hsa-miR-7155-5p |  |
|  |  | hsa-miR-7155-5p |  |
|  |  | hsa-miR-7157-3p |  |
|  |  | hsa-miR-7159-3p |  |
|  |  | hsa-miR-7162-5p |  |
|  |  | hsa-miR-7-2-3p |  |
|  |  | hsa-miR-7-2-3p |  |
|  |  | hsa-miR-7-2-3p |  |
|  |  | hsa-miR-7-2-3p |  |
|  |  | hsa-miR-758-5p |  |
|  |  | hsa-miR-7-5p |  |
|  |  | hsa-miR-7-5p |  |
|  |  | hsa-miR-761 |  |
|  |  | hsa-miR-7843-3p |  |
|  |  | hsa-miR-7843-5p |  |
|  |  | hsa-miR-7843-5p |  |
|  |  | hsa-miR-7844-5p |  |
|  |  | hsa-miR-7844-5p |  |
|  |  | hsa-miR-7850-5p |  |
|  |  | hsa-miR-7852-3p |  |
|  |  | hsa-miR-7853-5p |  |
|  |  | hsa-miR-7977 |  |
|  |  | hsa-miR-7978 |  |
|  |  | hsa-miR-8054 |  |
|  |  | hsa-miR-8059 |  |
|  |  | hsa-miR-8063 |  |
|  |  | hsa-miR-8063 |  |
|  |  | hsa-miR-8063 |  |
|  |  | hsa-miR-8064 |  |
|  |  | hsa-miR-8065 |  |
|  |  | hsa-miR-8066 |  |
|  |  | hsa-miR-8067 |  |
|  |  | hsa-miR-8076 |  |
|  |  | hsa-miR-8076 |  |
|  |  | hsa-miR-8079 |  |
|  |  | hsa-miR-8079 |  |
|  |  | hsa-miR-8080 |  |
|  |  | hsa-miR-8081 |  |
|  |  | hsa-miR-8083 |  |
|  |  | hsa-miR-8089 |  |
|  |  | hsa-miR-8089 |  |
|  |  | hsa-miR-877-5p |  |
|  |  | hsa-miR-885-5p |  |
|  |  | hsa-miR-888-3p |  |
|  |  | hsa-miR-889-3p |  |
|  |  | hsa-miR-892a |  |
|  |  | hsa-miR-892c-3p |  |
|  |  | hsa-miR-892c-3p |  |
|  |  | hsa-miR-934 |  |
|  |  | hsa-miR-936 |  |
|  |  | hsa-miR-939-3p |  |
|  |  | hsa-miR-943 |  |
|  |  | hsa-miR-944 |  |
|  |  | hsa-miR-944 |  |
|  |  | hsa-miR-95-5p |  |
|  |  | hsa-miR-9-5p |  |
|  |  | hsa-miR-96-5p |  |
|  |  | hsa-miR-98-3p |  |
|  |  | hsa-miR-99a-3p |  |
|  |  | hsa-miR-99b-3p |  |

**Table S10. Primers and sequences used in this study**

| **Primers for qRT-PCR** | | |
| --- | --- | --- |
| MiR-143-3p RT | GTCGTATCCAGTGCAGGGTCCGAGGTATTCGCACTGGATACGACGAGCTA | |
| MiR-15b-3p RT | GTCGTATCCAGTGCAGGGTCCGAGGTATTCGCACTGGATACGACTGTAAA | |
| MiR-27a-3p RT | GTCGTATCCAGTGCAGGGTCCGAGGTATTCGCACTGGATACGACGCGGAA | |
| MiR-27b-3p RT | GTCGTATCCAGTGCAGGGTCCGAGGTATTCGCACTGGATACGACGCAGAA | |
| MiR-335 RT | GTCGTATCCAGTGCAGGGTCCGAGGTATTCGCACTGGATACGACACATTT | |
| MiR-495 RT | GTCGTATCCAGTGCAGGGTCCGAGGTATTCGCACTGGATACGACAAGAAG | |
| MiR-7 RT | GTCGTATCCAGTGCAGGGTCCGAGGTATTCGCACTGGATACGACAACAAC | |
| U6 RT | GTCGTATCCAGTGCAGGGTCCGAGGTATTCGCACTGGAT  ACGACAAAATATG | |
| MiR-143-3p | F | CGCGCTGAGATGAAGCACTG |
| MiR-15b-3p | F | CCGGGTAGCAGCACATCATG |
| MiR-27a-3p | F | GCGCTTCACAGTGGCTAAG |
| MiR-27b-3p | F | GCGCTTCACAGTGGCTAAG |
| MiR-335 | F | GCGCTCAAGAGCAATAACGAA |
| MiR-495 | F | GCGCAAACAAACATGGTGCA |
| MiR-7 | F | GCGCTGGAAGACTAGTGATTTT |
| U6 | F | GCGCGTCGTGAAGCGTTC |
| Universal Reverse  Primer | R | CAGTGCAGGGTCCGAGGT |
| RNF111 | F | CATTGGGGCAGCCAAAAGTT |
|  | R | TGAGGGAACAGCTGGATTGT |
| CircRNF111 | F | TAGCAGTTCCCCAATCCTTG |
|  | R | CACAAATTCCCATCATTCCC |
| Hsa_circ_0000431 | F | CATCTCTGCCGTTTCTTGCG |
|  | R | GCACAGCTAACTCCTTTTCTCTTG |
| Hsa_circ_0000937 | F | CACTATCTGCTGAGCCAAGG |
|  | R | CCAGAGATGAAAACTGCTGCTG |
| Hsa_circ_0001564 | F | CATCCTTTGCGCTCAGAGGA |
|  | R | GATTGGCCTGACCACAGTCTA |
| Hsa_circ_0001849 | F | AGCCTCAGAAGCCAACTCCTTTG |
|  | R | TCAGGTTGAGATTTGAAGTCAAGAT |
| Hsa_circ_0000711 | F | AACTCATCATCGAGCCCATT |
|  | R | ATGCACAATCATCTGGCTCA |
| Hsa_circ_0000798 | F | TTGAAGTGCAGGTACAGGTGA |
|  | R | GCTGGACCCACACTTGATGA |
| Hsa_circ_0000816 | F | AGACAGCCCGAAGGTGC |
|  | R | CTTCACTGGAGACTCAGACGC |
| Hsa_circ_0001163 | F | ACAGGATGATCGAAGCAAAGC |
|  | R | GAATCTGGCGCTTGGGAGAG |
| Hsa_circ_0001524 | F | TTGGGCACAACTGGTTCACAG |
|  | R | AGCTGCTGAGAGATGCAGACC |
| GADPH | F | AGCCACATCGCTCAGACAC |
|  | R | GCCCAATACGACCAAATCC |
| IGF2R | F | CCGGCGTGCTCTGGA |
|  | R | CCAGAGGGTCACAGTGGAAGA |
| IGFBP5 | F | TACCTGCCCAATTGTGACC |
|  | R | AACGTTGCTGCTGTCGAAG |
| **Cloning primers** | | |
| oe-IGF2R | F | GeneChem |
|  | R | GeneChem |
| IGF2R-3’UTR-MT | F | CCGCTCGAGTGGGGTATAGGTCCCGTAAAT |
|  | R | CCCAAGCTTAGACCTGACATGTCTTCCTCA |
| IGF2R-3’UTR-Mut | F | CCGCTCGAGTGGGGTATAGGTTCATCTCT |
|  | R | CCCAAGCTTAGACCTGACATGTCTTCCTCA |
|  |  |  |
| **Primers for PCR** | | |
| Divergent-GAPDH | F | GAAGGTGAAGGTCGAGTC |
|  | R | GAAGATGGTGATGGGATTTC |
| Converge-GAPDH | F | CAATGACCCCTTCATTGACC |
|  | R | TTGATTTTGGAGGGATCTCG |
| Divergent-circRNF111 | F | TAGCAGTTCCCCAATCCTTG |
|  | R | CACAAATTCCCATCATTCCC |
| Converge- circRNF111 | F | CATTGGGGCAGCCAAAAGTT |
|  | R | TGAGGGAACAGCTGGATTGT |
|  |  |  |
| **ShRNA sequences** | | |
| CircRNF111 shRNA |  | CCGGCCGCCTCAAGTGGATTATGTTCTCGAGAACATAATCCACTTGAGGCGGTTTTT |
|  |  |  |
| **SiRNAs sequences** | | |
| Scramble siRNA |  | UUCUCCGAACGUGUCACGUTT |
| CircRNF111 siRNA-1 |  | CUCAGGCUUUCCUUAAAGUUU |
| CircRNF111 siRNA-2 |  | CCCUCAGGCUUUCCUUAAAGU |
| CircRNF111 siRNA-3 |  | CCUCAGGCUUUCCUUAAAGUU |
| CircRNF111 siRNA-4 |  | CCAGCUGUUCCCUCAGGCUUU |
|  |  |  |
| **Probes for FISH** | | |
| Alexa flour 488-miR-143-3p |  | GAGCTACAGTGCTTCATCUCA |
| Cy3-circRNF111 |  | TACAGAGTTACCTGAGGACTTATATTGC |
|  |  |  |
| **Pull down probe sequences** | | |
| CircRNF111 pull-down probes | 1 | GAATTTCAAAGGGTACAGAGTTACCTGAGGACTTATATTGCTCGAGATGTGGAATTTTCA |
|  | 2 | TACTTCTCACTCTAAGGAAGACTACGTGGTTTCTGTGTCCTCTCAGACTTTCCCTAGGAA |
|  | 3 | GATCATCAAGGTCACTCTTACTGGAGTCGTCACTTAGGAGAAGATCGAGTAGTTGACTTC |
| Control probes | 1 | CAAACGGCGGATTGACCGTAATGGGATAGGTCACGTTGGTGTAGATGGGCGCATCGTAAC |
|  | 2 | CACCACATACAGGCCGTAGCGGTCGCACAGCGTGTACCACAGCGGATGGTTCGGATAATG |
|  | 3 | CCAATCCGCGCCGGATGCGGTGTATCGCTCGCCACTTCAACATCAACGGTAATCGCCATT |
|  |  |  |
| **MiRNA regent sequences** | | |
| MiR-143-3p agomir |  | RiboBio |
| Agomir NC |  | RiboBio |
| MiR-143-3p antagomir |  | RiboBio |
| Antagomir NC |  | RiboBio |
