# Supplementary material for: CircRNF111 Protects Against Insulin Resistance and Lipid Deposition via Regulating miR-143-3p/IGF2R Axis in Metabolic Syndrome
Source: Front Cell Dev Biol. 2021 Aug 17;9:663148. doi: 10.3389/fcell.2021.663148 (PMC8415985; doi:10.3389/fcell.2021.663148)
Supplement: Supplementary file 1 [file Data_Sheet_1.ZIP › Supplemental File Sets/Supplementary Table 1.docx]

Supplementary Table 1. The circRNAs differentially expressing between MetS and non-MetS with fold change >2 and p<0.05

| ID | log2Fold Change (MetS/Control) | p value | direction | significant |
| --- | --- | --- | --- | --- |
| 5:32955852-32968771 | -7.2095933 | 0.00013432 | down | yes |
| 17:57406833-57408495 | -5.7256104 | 0.0001382 | down | yes |
| 15:59323002-59323901 | -5.2760784 | 0.0001862 | down | yes |
| 6:134115219-134137417 | 4.06243391 | 0.00040484 | up | yes |
| 1:146682575-146701846 | -4.0531467 | 0.00111472 | down | yes |
| 6:134062063-134066465 | -3.7749837 | 0.00122753 | down | yes |
| 15:27553688-27571654 | -3.4219483 | 0.00296286 | down | yes |
| 17:50036940-50055627 | -3.85047 | 0.00300782 | down | yes |
| 11:117290397-117291036 | 3.52357915 | 0.00303382 | up | yes |
| 12:83574631-83575136 | 4.56983277 | 0.00524299 | up | yes |
| 10:98968693-98969124 | 2.04618052 | 0.0061645 | up | yes |
| 6:99435215-99456580 | -3.8014826 | 0.00705903 | down | yes |
| 14:118654149-118669107 | 2.86567306 | 0.00779452 | up | yes |
| 15:6836915-6842917 | -3.4977975 | 0.00811642 | down | yes |
| 6:99429209-99435345 | -3.3472394 | 0.00862442 | down | yes |
| 9:100834417-100855904 | -3.6844846 | 0.00873229 | down | yes |
| 4:88121269-88129052 | -3.3103606 | 0.00929394 | down | yes |
| 14:48168196-48179276 | -3.5440215 | 0.00954736 | down | yes |
| 1:191568290-191570704 | -3.2840934 | 0.01020717 | down | yes |
| 1:39973926-39980650 | 3.91773309 | 0.01077985 | up | yes |
| 17:57406833-57420524 | -2.8955174 | 0.01078439 | down | yes |
| 2:173688146-173695999 | -2.7507601 | 0.0134045 | down | yes |
| 12:69302323-69303962 | 2.2484411 | 0.01342013 | up | yes |
| 3:95991443-95992278 | -2.7923131 | 0.01370568 | down | yes |
| 8:56564557-56569410 | -2.7154978 | 0.0139525 | down | yes |
| 2:119076935-119086650 | -2.8798374 | 0.01416384 | down | yes |
| 9:64212583-64214609 | 3.54082201 | 0.01564169 | up | yes |
| 16:91782160-91793767 | -2.6103884 | 0.01569604 | down | yes |
| 17:74225431-74245087 | -4.0256687 | 0.01604729 | down | yes |
| 18:82955523-82957449 | 2.1840816 | 0.01628771 | up | yes |
| 17:39843774-39843992 | -2.3340353 | 0.01870983 | down | yes |
| 2:156177632-156178952 | -2.6963238 | 0.01990161 | down | yes |
| 1:165604609-165608604 | -2.298018 | 0.02001182 | down | yes |
| 4:63461281-63472868 | 2.33188823 | 0.0213266 | up | yes |
| 15:98951542-98953500 | -2.5316566 | 0.02133504 | down | yes |
| 9:119284545-119294523 | -2.8672356 | 0.02267481 | down | yes |
| 1:181956393-181961919 | -2.4410486 | 0.0227498 | down | yes |
| 17:79650301-79659473 | -2.7149079 | 0.02489881 | down | yes |
| 2:70768861-70786995 | -2.336148 | 0.02519941 | down | yes |
| 9:115266093-115280519 | 3.20707142 | 0.02528824 | up | yes |
| 3:98070859-98072922 | -3.0206565 | 0.02539876 | down | yes |
| 15:33249925-33302547 | -2.8597513 | 0.02762357 | down | yes |
| 7:118193449-118196035 | -2.4169485 | 0.02873991 | down | yes |
| 15:38493473-38498841 | 3.03998404 | 0.02895611 | up | yes |
| X:142832500-142834393 | 2.08199876 | 0.02926914 | up | yes |
| 18:5641381-5705243 | 2.07557749 | 0.03222577 | up | yes |
| 11:74656422-74657339 | 1.90831565 | 0.03320324 | up | yes |
| 9:82945757-82959851 | -2.4219052 | 0.03337239 | down | yes |
| 4:9529885-9532065 | -2.2898914 | 0.03571296 | down | yes |
| 15:12889618-12905701 | -2.1584349 | 0.03659215 | down | yes |
| 10:34583897-34591970 | 1.58844426 | 0.03682162 | up | yes |
| 19:4784723-4788042 | -2.5143186 | 0.0371715 | down | yes |
| 2:69914413-69922933 | -2.8364955 | 0.03729233 | down | yes |
| 18:61157342-61157761 | 2.12444385 | 0.03814177 | up | yes |
| X:151376747-151399803 | 3.08890248 | 0.03816561 | up | yes |
| 7:110052710-110054116 | 3.01222312 | 0.03835631 | up | yes |
| 18:36638204-36638708 | -2.181505 | 0.03909558 | down | yes |
| 11:97229829-97238258 | 2.89470144 | 0.03929864 | up | yes |
| 7:98243948-98266247 | 2.14474309 | 0.03967021 | up | yes |
| 1:33512015-33533363 | -2.7392927 | 0.03997965 | down | yes |
| 5:115884865-115886807 | -2.1872198 | 0.04022615 | down | yes |
| 4:152206763-152223162 | 2.0526393 | 0.04027099 | up | yes |
| 9:83952523-83991822 | -2.0173228 | 0.04080457 | down | yes |
| 2:128668924-128684663 | 1.70272444 | 0.041213 | up | yes |
| 6:119002576-119057515 | -2.1089966 | 0.04141039 | down | yes |
| 19:45630864-45640521 | -2.5100824 | 0.0422463 | down | yes |
| 19:40573770-40580076 | -2.0894124 | 0.04274839 | down | yes |
| 7:126551974-126552338 | -2.1791208 | 0.04383696 | down | yes |
| 14:57856609-57865641 | -2.8769173 | 0.04408098 | down | yes |
| 2:27922016-27929541 | -2.1044171 | 0.04411623 | down | yes |
| 5:36571295-36572037 | -2.3004161 | 0.04427124 | down | yes |
| 5:96128942-96134132 | 1.90239975 | 0.04490208 | up | yes |
| 5:114102382-114103826 | 2.00810182 | 0.0450386 | up | yes |
| 11:96770638-96773222 | -2.448017 | 0.04527876 | down | yes |
| 14:52181195-52181536 | -2.309055 | 0.04536368 | down | yes |
| 6:113408904-113409340 | 1.95850887 | 0.04637736 | up | yes |
| 10:116517316-116549175 | -2.4859603 | 0.04698215 | down | yes |
| 2:131511983-131516443 | 1.76079796 | 0.0470835 | up | yes |
| 9:21772744-21774752 | 1.91782781 | 0.0474196 | up | yes |
| 13:103884142-103889285 | 2.26421757 | 0.04906542 | up | yes |
| 13:103140162-103171556 | -2.104443 | 0.04925581 | down | yes |
| 4:59514273-59524476 | -2.234238 | 0.04941287 | down | yes |
| 3:116647781-116651499 | 2.13070195 | 0.04979502 | up | yes |
| 7:111989015-112004131 | -1.9814785 | 0.04990831 | down | yes |
