# Supplementary material for: CircRNF111 Protects Against Insulin Resistance and Lipid Deposition via Regulating miR-143-3p/IGF2R Axis in Metabolic Syndrome
Source: Front Cell Dev Biol. 2021 Aug 17;9:663148. doi: 10.3389/fcell.2021.663148 (PMC8415985; doi:10.3389/fcell.2021.663148)
Supplement: Supplementary file 1 [file Data_Sheet_1.ZIP › Supplemental File Sets/Supplementary Table 10.docx]

Supplementary Table 10. Primers and sequences used in this study

| **Primers for qRT-PCR** | | |
| --- | --- | --- |
| MiR-143-3p RT | GTCGTATCCAGTGCAGGGTCCGAGGTATTCGCACTGGATACGACGAGCTA | |
| MiR-15b-3p RT | GTCGTATCCAGTGCAGGGTCCGAGGTATTCGCACTGGATACGACTGTAAA | |
| MiR-27a-3p RT | GTCGTATCCAGTGCAGGGTCCGAGGTATTCGCACTGGATACGACGCGGAA | |
| MiR-27b-3p RT | GTCGTATCCAGTGCAGGGTCCGAGGTATTCGCACTGGATACGACGCAGAA | |
| MiR-335 RT | GTCGTATCCAGTGCAGGGTCCGAGGTATTCGCACTGGATACGACACATTT | |
| MiR-495 RT | GTCGTATCCAGTGCAGGGTCCGAGGTATTCGCACTGGATACGACAAGAAG | |
| MiR-7 RT | GTCGTATCCAGTGCAGGGTCCGAGGTATTCGCACTGGATACGACAACAAC | |
| U6 RT | GTCGTATCCAGTGCAGGGTCCGAGGTATTCGCACTGGAT  ACGACAAAATATG | |
| MiR-143-3p | F | CGCGCTGAGATGAAGCACTG |
| MiR-15b-3p | F | CCGGGTAGCAGCACATCATG |
| MiR-27a-3p | F | GCGCTTCACAGTGGCTAAG |
| MiR-27b-3p | F | GCGCTTCACAGTGGCTAAG |
| MiR-335 | F | GCGCTCAAGAGCAATAACGAA |
| MiR-495 | F | GCGCAAACAAACATGGTGCA |
| MiR-7 | F | GCGCTGGAAGACTAGTGATTTT |
| U6 | F | GCGCGTCGTGAAGCGTTC |
| Universal Reverse  Primer | R | CAGTGCAGGGTCCGAGGT |
| RNF111 | F | CATTGGGGCAGCCAAAAGTT |
|  | R | TGAGGGAACAGCTGGATTGT |
| CircRNF111 | F | TAGCAGTTCCCCAATCCTTG |
|  | R | CACAAATTCCCATCATTCCC |
| Hsa_circ_0000431 | F | CATCTCTGCCGTTTCTTGCG |
|  | R | GCACAGCTAACTCCTTTTCTCTTG |
| Hsa_circ_0000937 | F | CACTATCTGCTGAGCCAAGG |
|  | R | CCAGAGATGAAAACTGCTGCTG |
| Hsa_circ_0001564 | F | CATCCTTTGCGCTCAGAGGA |
|  | R | GATTGGCCTGACCACAGTCTA |
| Hsa_circ_0001849 | F | AGCCTCAGAAGCCAACTCCTTTG |
|  | R | TCAGGTTGAGATTTGAAGTCAAGAT |
| Hsa_circ_0000711 | F | AACTCATCATCGAGCCCATT |
|  | R | ATGCACAATCATCTGGCTCA |
| Hsa_circ_0000798 | F | TTGAAGTGCAGGTACAGGTGA |
|  | R | GCTGGACCCACACTTGATGA |
| Hsa_circ_0000816 | F | AGACAGCCCGAAGGTGC |
|  | R | CTTCACTGGAGACTCAGACGC |
| Hsa_circ_0001163 | F | ACAGGATGATCGAAGCAAAGC |
|  | R | GAATCTGGCGCTTGGGAGAG |
| Hsa_circ_0001524 | F | TTGGGCACAACTGGTTCACAG |
|  | R | AGCTGCTGAGAGATGCAGACC |
| GADPH | F | AGCCACATCGCTCAGACAC |
|  | R | GCCCAATACGACCAAATCC |
| IGF2R | F | CCGGCGTGCTCTGGA |
|  | R | CCAGAGGGTCACAGTGGAAGA |
| IGFBP5 | F | TACCTGCCCAATTGTGACC |
|  | R | AACGTTGCTGCTGTCGAAG |
| **Cloning primers** | | |
| oe-IGF2R | F | GeneChem |
|  | R | GeneChem |
| IGF2R-3’UTR-MT | F | CCGCTCGAGTGGGGTATAGGTCCCGTAAAT |
|  | R | CCCAAGCTTAGACCTGACATGTCTTCCTCA |
| IGF2R-3’UTR-Mut | F | CCGCTCGAGTGGGGTATAGGTTCATCTCT |
|  | R | CCCAAGCTTAGACCTGACATGTCTTCCTCA |
|  |  |  |
| **Primers for PCR** | | |
| Divergent-GAPDH | F | GAAGGTGAAGGTCGAGTC |
|  | R | GAAGATGGTGATGGGATTTC |
| Converge-GAPDH | F | CAATGACCCCTTCATTGACC |
|  | R | TTGATTTTGGAGGGATCTCG |
| Divergent-circRNF111 | F | TAGCAGTTCCCCAATCCTTG |
|  | R | CACAAATTCCCATCATTCCC |
| Converge- circRNF111 | F | CATTGGGGCAGCCAAAAGTT |
|  | R | TGAGGGAACAGCTGGATTGT |
|  |  |  |
| **ShRNA sequences** | | |
| CircRNF111 shRNA |  | CCGGCCGCCTCAAGTGGATTATGTTCTCGAGAACATAATCCACTTGAGGCGGTTTTT |
|  |  |  |
| **SiRNAs sequences** | | |
| Scramble siRNA |  | UUCUCCGAACGUGUCACGUTT |
| CircRNF111 siRNA-1 |  | CUCAGGCUUUCCUUAAAGUUU |
| CircRNF111 siRNA-2 |  | CCCUCAGGCUUUCCUUAAAGU |
| CircRNF111 siRNA-3 |  | CCUCAGGCUUUCCUUAAAGUU |
| CircRNF111 siRNA-4 |  | CCAGCUGUUCCCUCAGGCUUU |
|  |  |  |
| **Probes for FISH** | | |
| Alexa flour 488-miR-143-3p |  | GAGCTACAGTGCTTCATCUCA |
| Cy3-circRNF111 |  | TACAGAGTTACCTGAGGACTTATATTGC |
|  |  |  |
| **Pull down probe sequences** | | |
| CircRNF111 pull-down probes | 1 | GAATTTCAAAGGGTACAGAGTTACCTGAGGACTTATATTGCTCGAGATGTGGAATTTTCA |
|  | 2 | TACTTCTCACTCTAAGGAAGACTACGTGGTTTCTGTGTCCTCTCAGACTTTCCCTAGGAA |
|  | 3 | GATCATCAAGGTCACTCTTACTGGAGTCGTCACTTAGGAGAAGATCGAGTAGTTGACTTC |
| Control probes | 1 | CAAACGGCGGATTGACCGTAATGGGATAGGTCACGTTGGTGTAGATGGGCGCATCGTAAC |
|  | 2 | CACCACATACAGGCCGTAGCGGTCGCACAGCGTGTACCACAGCGGATGGTTCGGATAATG |
|  | 3 | CCAATCCGCGCCGGATGCGGTGTATCGCTCGCCACTTCAACATCAACGGTAATCGCCATT |
|  |  |  |
| **MiRNA regent sequences** | | |
| MiR-143-3p agomir |  | RiboBio |
| Agomir NC |  | RiboBio |
| MiR-143-3p antagomir |  | RiboBio |
| Antagomir NC |  | RiboBio |
