# Supplementary material for: CircRNF111 Protects Against Insulin Resistance and Lipid Deposition via Regulating miR-143-3p/IGF2R Axis in Metabolic Syndrome
Source: Front Cell Dev Biol. 2021 Aug 17;9:663148. doi: 10.3389/fcell.2021.663148 (PMC8415985; doi:10.3389/fcell.2021.663148)
Supplement: Supplementary file 1 [file Data_Sheet_1.ZIP › Supplemental File Sets/Supplementary Table 2.docx]

Supplementary Table 2. Clinical characteristics of the participants included in the validation study

| Characteristic | Total | non-MetS | MetS | p value |
| --- | --- | --- | --- | --- |
| N | 80 | 40 | 40 |  |
| Serum circRNF111 | 0.96±0.65 | 1.10±0.71 | 0.82±0.56 | 0.0219 |
| Urine circRNF111 | 0.49±0.41 | 0.58±0.40 | 0.40±0.41 | 0.0109 |
| Age (years) | 56.40±6.52 | 56.33±6.12 | 56.82±6.58 | 0.782 |
| Male, n(%) | 41(51.25) | 18(45.00) | 23(57.50) | 0.532 |
| Current smoker, n(%) | 34(42.5) | 8(10) | 26(32.5) | <0.001 |
| Alcohol drinker, n(%) | 25(31.25) | 13(16.25) | 12(15) | 0.117 |
| BMI (kg/m^2^) | 23.65±3.12 | 21.87±2.26 | 25.32±2.53 | <0.001 |
| WC (cm) | 80.56(68.13-91.10) | 72.86(68.13-83.25) | 86.66(83.23-91.10) | <0.001 |
| WHR | 0.89(0.80-0.98) | 0.84(0.80-0.88) | 0.93(0.89-0.98) | <0.001 |
| Body fat (%) | 28.70±6.48 | 26.24±5.66 | 30.89±6.40 | 0.001 |
| SBP (mmHg) | 123.45(110.10-141.52) | 116.68(110.10-123.30) | 124.82(118.82-141.52) | <0.001 |
| DBP (mmHg) | 81.17±9.20 | 78.35±7.86 | 84.30±8.72 | <0.001 |
| HbA1c (%) | 5.66(5.30-6.20) | 5.50(5.13-5.80) | 5.72(5.40-6.20) | 0.011 |
| ALT (U/l) | 18.10(14.00-26.10) | 17.60(10.90-25.40) | 29.15(16.90-33.30) | 0.001 |
| AST (U/l) | 19.00(16.00-23.00) | 19.82(14.70-24.80) | 23.53(17.50-29.60) | 0.164 |
| FPG (mmol/l) | 5.25(4.60-5.54) | 6.72(6.40-7.43) | 7.44(6.83-7.87) | 0.002 |
| 2 h postprandial glucose (mmol/l) | 6.05(4.82-7.12) | 7.59(6.4-8.6) | 10.19(7.62-15.44) | <0.001 |
| FINS (μU/ml) | 10.68(8.18-13.88) | 11.50±3.20 | 18.27±4.10 | <0.001 |
| 2 h INS (μU/ml) | 57.35(37.32-87.90) | 54.42(36.30-83.60) | 81.70(47.70-155.25) | <0.001 |
| HOMA-IR | 2.78(1.72-3.14) | 2.12(1.67-2.88) | 3.53(2.54-5.30) | <0.001 |
| TC (mmol/l) | 5.55±1.02 | 5.35±0.86 | 5.77±1.10 | 0.298 |
| LDL-c (mmol/l) | 2.24(2.00-2.60) | 2.32(1.91-2.74) | 2.30(1.70-2.89) | 0.801 |
| HDL-c (mmol/l) | 1.30(1.01-1.66) | 1.64(1.32-1.96) | 1.08(0.85-1.35) | <0.001 |
| TG (mmol/l) | 1.58(0.88-2.22) | 1.04(0.60-1.45) | 3.25(1.84-3.29) | <0.001 |
| SFA (cm^2^) | 152.74(122.50-192.45) | 150.69(105.70-205.10) | 170.45(122.28-228.26) | <0.001 |
| VFA (cm^2^) | 70.60(47.16-122.20) | 55.48(23.23-87.58) | 118.25(72.25-164.38) | <0.001 |

Data are presented as the mean ± standard deviation, the median with 25-75% interquartile range or n(%). BMI, body mass index; WC, waist circumference; WHR, waist-to-hip ratio; SBP, systolic blood pressure; DBP, diastolic blood pressure; HbA1c, hemoglobin A1c; ALT, alanine transaminase; AST, aspartate transaminase; FPG, fasting plasma glucose; FINS, fasting serum insulin levels; 2h INS, 2h postprandial insulin levels; HOMA-IR, homeostasis model assessment-insulin resistance; TC, total cholesterol; LDL-c, low-density lipoprotein-cholesterol; HDL-c high density lipoprotein-cholesterol; TG, triglyceride; SFA, abdominal subcutaneous fat area; VFA, visceral fat area.
