# Supplementary material for: CircRNF111 Protects Against Insulin Resistance and Lipid Deposition via Regulating miR-143-3p/IGF2R Axis in Metabolic Syndrome
Source: Front Cell Dev Biol. 2021 Aug 17;9:663148. doi: 10.3389/fcell.2021.663148 (PMC8415985; doi:10.3389/fcell.2021.663148)
Supplement: Supplementary file 1 [file Data_Sheet_1.ZIP › Supplemental File Sets/Supplementary Table 3&4.docx]

Supplementary Table3 - Spearman correlation analyses of serum circRNF111 with metabolic risk

|  | Unadjusted | | Adjusted  (age, gender, smoking drinking) | |
| --- | --- | --- | --- | --- |
|  | r | p value | r | p value |
| BMI (kg/m^2^) | -0.232 | 0.024 | -0.255 | 0.020 |
| WC (cm) | -0.187 | 0.062 | -0.221 | 0.032 |
| WHR | -0.167 | 0.097 | -0.161 | 0.146 |
| Fat% (%) | -0.302 | 0.009 | -0.309 | 0.008 |
| SBP (mmHg) | 0.071 | 0.382 | 0.085 | 0.414 |
| DBP (mmHg) | -0.081 | 0.393 | -0.064 | 0.570 |
| HbA1c (%) | -0.252 | 0.012 | -0.234 | 0.034 |
| FPG (mmol/L) | -0.125 | 0.215 | -0.109 | 0.328 |
| 2h PG (mmol/L) | -0.137 | 0.174 | -0.112 | 0.041 |
| FINS (μU/ml) | -0.313 | 0.006 | -0.275 | 0.056 |
| 2h INS (μU/ml) | -0.272 | 0.007 | -0.318 | 0.003 |
| HOMA-IR | -0.287 | 0.013 | -0.279 | 0.012 |
| TC (mmol/L) | -0.249 | 0.025 | -0.271 | 0.018 |
| LDL-c (mmol/L) | -0.085 | 0.405 | -0.111 | 0.316 |
| HDL-c (mmol/L) | 0.244 | 0.022 | 0.220 | 0.006 |
| TG (mmol/L) | -0.305 | 0.002 | -0.287 | 0.010 |
| SFA (cm^2^) | -0.185 | 0.091 | -0.126 | 0.060 |
| VFA (cm^2^) | -0.352 | ＜0.001 | -0.227 | 0.008 |

Supplementary Table4 - Spearman correlation analyses of urine circRNF111 with metabolic risk

|  | Unadjusted | | Adjusted  (age, gender, smoking drinking) | |
| --- | --- | --- | --- | --- |
|  | r | p value | r | p value |
| BMI (kg/m^2^) | -0.282 | 0.006 | -0.308 | 0.002 |
| WC (cm) | -0.172 | 0.086 | -0.208 | 0.018 |
| WHR | -0.124 | 0.221 | -0.191 | 0.066 |
| Fat% (%) | -0.262 | 0.002 | -0.239 | ＜0.001 |
| SBP (mmHg) | 0.171 | 0.182 | 0.185 | 0.114 |
| DBP (mmHg) | 0.044 | 0.633 | 0.094 | 0.366 |
| HbA1c (%) | -0.176 | 0.082 | -0.134 | 0.094 |
| FPG (mmol/L) | -0.225 | 0.015 | -0.209 | 0.026 |
| 2h PG (mmol/L) | -0.217 | 0.028 | -0.206 | 0.038 |
| FINS (μU/ml) | -0.214 | 0.032 | -0.278 | 0.016 |
| 2h INS (μU/ml) | -0.218 | 0.029 | -0.225 | 0.028 |
| HOMA-IR | -0.286 | 0.011 | -0.231 | 0.039 |
| TC (mmol/L) | -0.190 | 0.065 | -0.171 | 0.082 |
| LDL-c (mmol/L) | -0.142 | 0.115 | -0.116 | 0.266 |
| HDL-c (mmol/L) | 0.224 | 0.014 | 0.281 | 0.008 |
| TG (mmol/L) | -0.295 | ＜0.001 | -0.286 | 0.010 |
| SFA (cm^2^) | -0.225 | 0.024 | -0.220 | 0.035 |
| VFA (cm^2^) | -0.204 | 0.041 | -0.279 | 0.006 |
