# Supplementary material for: CircRNF111 Protects Against Insulin Resistance and Lipid Deposition via Regulating miR-143-3p/IGF2R Axis in Metabolic Syndrome
Source: Front Cell Dev Biol. 2021 Aug 17;9:663148. doi: 10.3389/fcell.2021.663148 (PMC8415985; doi:10.3389/fcell.2021.663148)
Supplement: Supplementary file 1 [file Data_Sheet_1.ZIP › Supplemental File Sets/Supplementary Table 5&6.docx]

Supplementary Table5 - Multiple stepwise regression analyses of independent factors associated with serum circRNF111 levels

| Independent variables | *β* | SE | Standard *β* | p value |
| --- | --- | --- | --- | --- |
| TG | -0.328 | 0.008 | -0.582 | <0.001 |
| HOMA-IR | -0.016 | 0.025 | -0.286 | 0.002 |
| VFA | -0.019 | 0.016 | -0.275 | 0.020 |

Supplementary Table6 - Multiple stepwise regression analyses of independent factors associated with urine circRNF111 levels

| Independent variables | *β* | SE | Standard *β* | p value |
| --- | --- | --- | --- | --- |
| HOMA-IR | -0.245 | 0.026 | -0.525 | <0.001 |
| Fat% | -0.140 | 0.019 | -0.308 | <0.001 |
| TG | -0.013 | 0.006 | -0.107 | 0.030 |
