# Supplementary material for: CircRNF111 Protects Against Insulin Resistance and Lipid Deposition via Regulating miR-143-3p/IGF2R Axis in Metabolic Syndrome
Source: Front Cell Dev Biol. 2021 Aug 17;9:663148. doi: 10.3389/fcell.2021.663148 (PMC8415985; doi:10.3389/fcell.2021.663148)
Supplement: Supplementary file 1 [file Data_Sheet_1.ZIP › Supplemental File Sets/Supplementary Table 7.docx]

Supplementary Table 7. RNA pull-down assay with a biotinylated circRNF111 probe followed by mass spectrometry (MS) analysis

| Anti-sense（control probe） | Sense（circRNF111 probe） |
| --- | --- |
| 130kDa Protein 4.1B MEF cell isoform OS=Homo sapiens GN=Epb41l3 PE=1 SV=1 | 16 kDa protein OS=Homo sapiens GN=p16 PE=4 SV=1 |
| 17beta-hydroxysteroid dehydrogenase type 10/short chain L-3-hydroxyacyl-CoA dehydrogenase OS=Homo sapiens GN=Hsd17b10 PE=1 SV=1 | 17beta-hydroxysteroid dehydrogenase type 10/short chain L-3-hydroxyacyl-CoA dehydrogenase OS=Homo sapiens GN=Hsd17b10 PE=1 SV=1 |
| 2900073G15Rik protein (Fragment) OS=Homo sapiens GN=Myl12a PE=2 SV=1 | 2900073G15Rik protein (Fragment) OS=Homo sapiens GN=Myl12a PE=2 SV=1 |
| 3-hydroxyacyl-CoA dehydrogenase type-2 OS=Homo sapiens GN=Hsd17b10 PE=1 SV=1 | 3-hydroxyacyl-CoA dehydrogenase type-2 OS=Homo sapiens GN=Hsd17b10 PE=1 SV=1 |
| 3-hydroxyacyl-CoA dehydrogenase type-2 OS=Homo sapiens GN=Hsd17b10 PE=1 SV=4 | 3-hydroxyacyl-CoA dehydrogenase type-2 OS=Homo sapiens GN=Hsd17b10 PE=1 SV=4 |
| 4.1G protein (Fragment) OS=Homo sapiens GN=Epb41l2 PE=2 SV=1 | 40S ribosomal protein S10 OS=Homo sapiens GN=Rps10 PE=1 SV=1 |
| 40S ribosomal protein S10 OS=Homo sapiens GN=Rps10 PE=1 SV=1 | 40S ribosomal protein S11 (Fragment) OS=Homo sapiens GN=Rps11 PE=1 SV=1 |
| 40S ribosomal protein S11 (Fragment) OS=Homo sapiens GN=Rps11 PE=1 SV=1 | 40S ribosomal protein S11 OS=Homo sapiens GN=Rps11 PE=1 SV=1 |
| 40S ribosomal protein S11 OS=Homo sapiens GN=Rps11 PE=1 SV=1 | 40S ribosomal protein S11 OS=Homo sapiens GN=Rps11 PE=1 SV=3 |
| 40S ribosomal protein S11 OS=Homo sapiens GN=Rps11 PE=1 SV=3 | 40S ribosomal protein S13 OS=Homo sapiens GN=Rps13 PE=1 SV=1 |
| 40S ribosomal protein S13 OS=Homo sapiens GN=Rps13 PE=1 SV=1 | 40S ribosomal protein S13 OS=Homo sapiens GN=Rps13 PE=1 SV=2 |
| 40S ribosomal protein S13 OS=Homo sapiens GN=Rps13 PE=1 SV=2 | 40S ribosomal protein S14 (Fragment) OS=Homo sapiens GN=Rps14 PE=1 SV=1 |
| 40S ribosomal protein S14 (Fragment) OS=Homo sapiens GN=Rps14 PE=1 SV=1 | 40S ribosomal protein S14 OS=Homo sapiens GN=Rps14 PE=1 SV=3 |
| 40S ribosomal protein S14 OS=Homo sapiens GN=Rps14 PE=1 SV=3 | 40S ribosomal protein S15a (Fragment) OS=Homo sapiens GN=Rps15a PE=1 SV=1 |
| 40S ribosomal protein S15a (Fragment) OS=Homo sapiens GN=Rps15a PE=1 SV=1 | 40S ribosomal protein S15a (Fragment) OS=Homo sapiens GN=Rps15a PE=1 SV=2 |
| 40S ribosomal protein S15a (Fragment) OS=Homo sapiens GN=Rps15a PE=1 SV=2 | 40S ribosomal protein S15a OS=Homo sapiens GN=Rps15a PE=1 SV=2 |
| 40S ribosomal protein S15a OS=Homo sapiens GN=Rps15a PE=1 SV=2 | 40S ribosomal protein S16 OS=Homo sapiens GN=Rps16 PE=1 SV=4 |
| 40S ribosomal protein S16 OS=Homo sapiens GN=Rps16 PE=1 SV=4 | 40S ribosomal protein S17 OS=Homo sapiens GN=Rps17 PE=1 SV=2 |
| 40S ribosomal protein S17 OS=Homo sapiens GN=Rps17 PE=1 SV=2 | 40S ribosomal protein S18 OS=Homo sapiens GN=Rps18 PE=3 SV=1 |
| 40S ribosomal protein S18 OS=Homo sapiens GN=Rps18 PE=3 SV=1 | 40S ribosomal protein S19 (Fragment) OS=Homo sapiens GN=Rps19 PE=1 SV=2 |
| 40S ribosomal protein S19 (Fragment) OS=Homo sapiens GN=Rps19 PE=1 SV=2 | 40S ribosomal protein S19 (Fragment) OS=Homo sapiens GN=Rps19 PE=1 SV=8 |
| 40S ribosomal protein S19 (Fragment) OS=Homo sapiens GN=Rps19 PE=1 SV=8 | 40S ribosomal protein S19 OS=Homo sapiens GN=Rps19 PE=1 SV=1 |
| 40S ribosomal protein S19 OS=Homo sapiens GN=Rps19 PE=1 SV=1 | 40S ribosomal protein S19 OS=Homo sapiens GN=Rps19 PE=1 SV=3 |
| 40S ribosomal protein S19 OS=Homo sapiens GN=Rps19 PE=1 SV=3 | 40S ribosomal protein S2 (Fragment) OS=Homo sapiens GN=Rps2 PE=1 SV=1 |
| 40S ribosomal protein S2 (Fragment) OS=Homo sapiens GN=Rps2 PE=1 SV=1 | 40S ribosomal protein S2 OS=Homo sapiens GN=Rps2 PE=1 SV=1 |
| 40S ribosomal protein S2 OS=Homo sapiens GN=Rps2 PE=1 SV=1 | 40S ribosomal protein S2 OS=Homo sapiens GN=Rps2 PE=1 SV=3 |
| 40S ribosomal protein S2 OS=Homo sapiens GN=Rps2 PE=1 SV=3 | 40S ribosomal protein S23 OS=Homo sapiens GN=Rps23 PE=1 SV=3 |
| 40S ribosomal protein S23 OS=Homo sapiens GN=Rps23 PE=1 SV=3 | 40S ribosomal protein S24 OS=Homo sapiens GN=Rps24 PE=1 SV=1 |
| 40S ribosomal protein S24 OS=Homo sapiens GN=Rps24 PE=1 SV=1 | 40S ribosomal protein S24 OS=Homo sapiens GN=Rps24 PE=2 SV=1 |
| 40S ribosomal protein S24 OS=Homo sapiens GN=Rps24 PE=2 SV=1 | 40S ribosomal protein S25 OS=Homo sapiens GN=Rps25 PE=1 SV=1 |
| 40S ribosomal protein S25 OS=Homo sapiens GN=Rps25 PE=1 SV=1 | 40S ribosomal protein S26 OS=Homo sapiens GN=Rps26 PE=1 SV=3 |
| 40S ribosomal protein S26 OS=Homo sapiens GN=Rps26 PE=1 SV=3 | 40S ribosomal protein S27 (Fragment) OS=Homo sapiens GN=Rps27 PE=1 SV=1 |
| 40S ribosomal protein S29 OS=Homo sapiens GN=Rps29 PE=3 SV=2 | 40S ribosomal protein S27 OS=Homo sapiens GN=Rps27 PE=1 SV=3 |
| 40S ribosomal protein S3 OS=Homo sapiens GN=Rps3 PE=1 SV=1 | 40S ribosomal protein S27 OS=Homo sapiens GN=Rps27 PE=3 SV=1 |
| 40S ribosomal protein S30 (Fragment) OS=Homo sapiens GN=fau PE=3 SV=1 | 40S ribosomal protein S27-like OS=Homo sapiens GN=Rps27l PE=1 SV=3 |
| 40S ribosomal protein S30 OS=Homo sapiens GN=Fau PE=1 SV=1 | 40S ribosomal protein S3 OS=Homo sapiens GN=Rps3 PE=1 SV=1 |
| 40S ribosomal protein S30 OS=Homo sapiens PE=2 SV=1 | 40S ribosomal protein S30 (Fragment) OS=Homo sapiens GN=fau PE=3 SV=1 |
| 40S ribosomal protein S3a OS=Homo sapiens GN=Rps3a PE=1 SV=3 | 40S ribosomal protein S30 OS=Homo sapiens GN=Fau PE=1 SV=1 |
| 40S ribosomal protein S3a OS=Homo sapiens GN=Rps3a1 PE=2 SV=1 | 40S ribosomal protein S30 OS=Homo sapiens PE=2 SV=1 |
| 40S ribosomal protein S4 OS=Homo sapiens GN=Gm15013 PE=3 SV=1 | 40S ribosomal protein S3a OS=Homo sapiens GN=Rps3a PE=1 SV=3 |
| 40S ribosomal protein S4 OS=Homo sapiens GN=Rps4l PE=2 SV=1 | 40S ribosomal protein S3a OS=Homo sapiens GN=Rps3a1 PE=2 SV=1 |
| 40S ribosomal protein S4 OS=Homo sapiens GN=Rps4x PE=1 SV=1 | 40S ribosomal protein S4 OS=Homo sapiens GN=Gm15013 PE=3 SV=1 |
| 40S ribosomal protein S4 OS=Homo sapiens GN=Rps4x PE=2 SV=1 | 40S ribosomal protein S4 OS=Homo sapiens GN=Rps4l PE=2 SV=1 |
| 40S ribosomal protein S5 (Fragment) OS=Homo sapiens GN=Rps5 PE=1 SV=1 | 40S ribosomal protein S4 OS=Homo sapiens GN=Rps4x PE=1 SV=1 |
| 40S ribosomal protein S5 OS=Homo sapiens GN=Rps5 PE=1 SV=1 | 40S ribosomal protein S4 OS=Homo sapiens GN=Rps4x PE=2 SV=1 |
| 40S ribosomal protein S5 OS=Homo sapiens GN=Rps5 PE=1 SV=3 | 40S ribosomal protein S5 (Fragment) OS=Homo sapiens GN=Rps5 PE=1 SV=1 |
| 40S ribosomal protein S6 OS=Homo sapiens GN=Rps6 PE=1 SV=1 | 40S ribosomal protein S5 OS=Homo sapiens GN=Rps5 PE=1 SV=1 |
| 40S ribosomal protein S6 OS=Homo sapiens PE=2 SV=1 | 40S ribosomal protein S5 OS=Homo sapiens GN=Rps5 PE=1 SV=3 |
| 40S ribosomal protein S7 OS=Homo sapiens GN=Gm9493 PE=3 SV=1 | 40S ribosomal protein S6 OS=Homo sapiens GN=Rps6 PE=1 SV=1 |
| 40S ribosomal protein S7 OS=Homo sapiens GN=Rps7 PE=2 SV=1 | 40S ribosomal protein S6 OS=Homo sapiens PE=2 SV=1 |
| 40S ribosomal protein S8 (Fragment) OS=Homo sapiens GN=Rps8 PE=2 SV=1 | 40S ribosomal protein S7 OS=Homo sapiens GN=Gm9493 PE=3 SV=1 |
| 40S ribosomal protein S8 OS=Homo sapiens GN=Rps8 PE=1 SV=1 | 40S ribosomal protein S7 OS=Homo sapiens GN=Rps7 PE=2 SV=1 |
| 40S ribosomal protein S8 OS=Homo sapiens GN=Rps8 PE=2 SV=1 | 40S ribosomal protein S8 (Fragment) OS=Homo sapiens GN=Rps8 PE=2 SV=1 |
| 40S ribosomal protein S9 (Fragment) OS=Homo sapiens GN=Rps9 PE=1 SV=1 | 40S ribosomal protein S8 OS=Homo sapiens GN=Rps8 PE=1 SV=1 |
| 40S ribosomal protein S9 (Fragment) OS=Homo sapiens GN=Rps9 PE=1 SV=8 | 40S ribosomal protein S8 OS=Homo sapiens GN=Rps8 PE=2 SV=1 |
| 40S ribosomal protein S9 OS=Homo sapiens GN=Rps9 PE=1 SV=1 | 40S ribosomal protein S9 (Fragment) OS=Homo sapiens GN=Rps9 PE=1 SV=1 |
| 40S ribosomal protein S9 OS=Homo sapiens GN=Rps9 PE=1 SV=3 | 40S ribosomal protein S9 (Fragment) OS=Homo sapiens GN=Rps9 PE=1 SV=8 |
| 40S ribosomal protein SA (Fragment) OS=Homo sapiens GN=Rpsa PE=1 SV=1 | 40S ribosomal protein S9 OS=Homo sapiens GN=Rps9 PE=1 SV=1 |
| 40S ribosomal protein SA OS=Homo sapiens GN=Rpsa PE=1 SV=4 | 40S ribosomal protein S9 OS=Homo sapiens GN=Rps9 PE=1 SV=3 |
| 60kDa 4.1B MEF cell isoform (Fragment) OS=Homo sapiens GN=Epb41l3 PE=2 SV=1 | 40S ribosomal protein SA (Fragment) OS=Homo sapiens GN=Rpsa PE=1 SV=1 |
| 60S acidic ribosomal protein P0 (Fragment) OS=Homo sapiens GN=Rplp0 PE=1 SV=1 | 40S ribosomal protein SA OS=Homo sapiens GN=Rpsa PE=1 SV=4 |
| 60S acidic ribosomal protein P0 OS=Homo sapiens GN=Rplp0 PE=2 SV=1 | 5'-3' exoribonuclease 2 OS=Homo sapiens GN=Xrn2 PE=1 SV=1 |
| 60S ribosomal protein L10 (Fragment) OS=Homo sapiens GN=Rpl10 PE=1 SV=1 | 60S acidic ribosomal protein P0 (Fragment) OS=Homo sapiens GN=Rplp0 PE=1 SV=1 |
| 60S ribosomal protein L10 OS=Homo sapiens GN=Rpl10 PE=1 SV=3 | 60S acidic ribosomal protein P0 OS=Homo sapiens GN=Rplp0 PE=2 SV=1 |
| 60S ribosomal protein L10a OS=Homo sapiens GN=Rpl10a PE=1 SV=1 | 60S ribosomal protein L10 (Fragment) OS=Homo sapiens GN=Rpl10 PE=1 SV=1 |
| 60S ribosomal protein L10a OS=Homo sapiens GN=Rpl10a PE=1 SV=3 | 60S ribosomal protein L10 OS=Homo sapiens GN=Rpl10 PE=1 SV=3 |
| 60S ribosomal protein L10-like OS=Homo sapiens GN=Rpl10l PE=2 SV=1 | 60S ribosomal protein L10-like OS=Homo sapiens GN=Rpl10l PE=2 SV=1 |
| 60S ribosomal protein L11 OS=Homo sapiens GN=Rpl11 PE=1 SV=4 | 60S ribosomal protein L11 OS=Homo sapiens GN=Rpl11 PE=1 SV=4 |
| 60S ribosomal protein L13 OS=Homo sapiens GN=Rpl13 PE=1 SV=3 | 60S ribosomal protein L13 OS=Homo sapiens GN=Rpl13 PE=1 SV=3 |
| 60S ribosomal protein L13 OS=Homo sapiens GN=Rpl13 PE=2 SV=1 | 60S ribosomal protein L13 OS=Homo sapiens GN=Rpl13 PE=2 SV=1 |
| 60S ribosomal protein L14 (Fragment) OS=Homo sapiens GN=Rpl14 PE=1 SV=1 | 60S ribosomal protein L17 OS=Homo sapiens GN=Rpl17 PE=1 SV=1 |
| 60S ribosomal protein L14 OS=Homo sapiens GN=Rpl14 PE=1 SV=3 | 60S ribosomal protein L17 OS=Homo sapiens GN=Rpl17 PE=1 SV=3 |
| 60S ribosomal protein L17 OS=Homo sapiens GN=Rpl17 PE=1 SV=1 | 60S ribosomal protein L18 (Fragment) OS=Homo sapiens GN=Rpl18 PE=1 SV=1 |
| 60S ribosomal protein L17 OS=Homo sapiens GN=Rpl17 PE=1 SV=3 | 60S ribosomal protein L18 OS=Homo sapiens GN=Rpl18 PE=1 SV=1 |
| 60S ribosomal protein L18 (Fragment) OS=Homo sapiens GN=Rpl18 PE=1 SV=1 | 60S ribosomal protein L21 OS=Homo sapiens GN=Rpl21 PE=1 SV=1 |
| 60S ribosomal protein L18 OS=Homo sapiens GN=Rpl18 PE=1 SV=1 | 60S ribosomal protein L21 OS=Homo sapiens GN=Rpl21 PE=1 SV=3 |
| 60S ribosomal protein L18a (Fragment) OS=Homo sapiens GN=Rpl18a PE=1 SV=1 | 60S ribosomal protein L22-like 1 OS=Homo sapiens GN=Rpl22l1 PE=1 SV=1 |
| 60S ribosomal protein L18a OS=Homo sapiens GN=Rpl18a PE=1 SV=1 | 60S ribosomal protein L23 (Fragment) OS=Homo sapiens GN=Rpl23 PE=1 SV=1 |
| 60S ribosomal protein L18a OS=Homo sapiens GN=Rpl18a PE=2 SV=1 | 60S ribosomal protein L23 OS=Homo sapiens GN=Rpl23 PE=1 SV=1 |
| 60S ribosomal protein L21 OS=Homo sapiens GN=Rpl21 PE=1 SV=1 | 60S ribosomal protein L24 OS=Homo sapiens GN=Rpl24 PE=1 SV=2 |
| 60S ribosomal protein L21 OS=Homo sapiens GN=Rpl21 PE=1 SV=3 | 60S ribosomal protein L26 (Fragment) OS=Homo sapiens GN=Rpl26 PE=1 SV=1 |
| 60S ribosomal protein L22-like 1 OS=Homo sapiens GN=Rpl22l1 PE=1 SV=1 | 60S ribosomal protein L26 OS=Homo sapiens GN=Rpl26 PE=1 SV=1 |
| 60S ribosomal protein L23 (Fragment) OS=Homo sapiens GN=Rpl23 PE=1 SV=1 | 60S ribosomal protein L27 (Fragment) OS=Homo sapiens GN=Rpl27 PE=1 SV=1 |
| 60S ribosomal protein L23 OS=Homo sapiens GN=Rpl23 PE=1 SV=1 | 60S ribosomal protein L27 (Fragment) OS=Homo sapiens PE=2 SV=1 |
| 60S ribosomal protein L24 OS=Homo sapiens GN=Rpl24 PE=1 SV=2 | 60S ribosomal protein L27 OS=Homo sapiens GN=Rpl27 PE=1 SV=1 |
| 60S ribosomal protein L26 (Fragment) OS=Homo sapiens GN=Rpl26 PE=1 SV=1 | 60S ribosomal protein L27a OS=Homo sapiens GN=Rpl27a PE=1 SV=5 |
| 60S ribosomal protein L26 OS=Homo sapiens GN=Rpl26 PE=1 SV=1 | 60S ribosomal protein L29 (Fragment) OS=Homo sapiens GN=Rpl29 PE=3 SV=1 |
| 60S ribosomal protein L27 (Fragment) OS=Homo sapiens GN=Rpl27 PE=1 SV=1 | 60S ribosomal protein L29 (Fragment) OS=Homo sapiens GN=Rpl29 PE=4 SV=1 |
| 60S ribosomal protein L27 (Fragment) OS=Homo sapiens PE=2 SV=1 | 60S ribosomal protein L29 OS=Homo sapiens GN=Gm17669 PE=3 SV=1 |
| 60S ribosomal protein L27 OS=Homo sapiens GN=Rpl27 PE=1 SV=1 | 60S ribosomal protein L29 OS=Homo sapiens GN=Gm3550 PE=3 SV=1 |
| 60S ribosomal protein L27a OS=Homo sapiens GN=Rpl27a PE=1 SV=5 | 60S ribosomal protein L29 OS=Homo sapiens GN=Gm5218 PE=3 SV=1 |
| 60S ribosomal protein L3 (Fragment) OS=Homo sapiens GN=Rpl3 PE=1 SV=1 | 60S ribosomal protein L29 OS=Homo sapiens GN=Rpl29 PE=1 SV=2 |
| 60S ribosomal protein L3 OS=Homo sapiens GN=Rpl3 PE=1 SV=1 | 60S ribosomal protein L3 (Fragment) OS=Homo sapiens GN=Rpl3 PE=1 SV=1 |
| 60S ribosomal protein L3 OS=Homo sapiens GN=Rpl3 PE=1 SV=3 | 60S ribosomal protein L3 OS=Homo sapiens GN=Rpl3 PE=1 SV=1 |
| 60S ribosomal protein L31 OS=Homo sapiens GN=Rpl31 PE=1 SV=1 | 60S ribosomal protein L3 OS=Homo sapiens GN=Rpl3 PE=1 SV=3 |
| 60S ribosomal protein L32 OS=Homo sapiens GN=Rpl32 PE=1 SV=2 | 60S ribosomal protein L31 OS=Homo sapiens GN=Rpl31 PE=1 SV=1 |
| 60S ribosomal protein L35 OS=Homo sapiens GN=Rpl35 PE=1 SV=1 | 60S ribosomal protein L34 OS=Homo sapiens GN=Rpl34 PE=1 SV=2 |
| 60S ribosomal protein L36 OS=Homo sapiens GN=Rpl36 PE=1 SV=1 | 60S ribosomal protein L35 OS=Homo sapiens GN=Rpl35 PE=1 SV=1 |
| 60S ribosomal protein L36 OS=Homo sapiens GN=Rpl36 PE=2 SV=1 | 60S ribosomal protein L36a OS=Homo sapiens GN=Rpl36a PE=1 SV=2 |
| 60S ribosomal protein L36 OS=Homo sapiens GN=Rpl36 PE=3 SV=2 | 60S ribosomal protein L6 (Fragment) OS=Homo sapiens GN=Rpl6 PE=1 SV=1 |
| 60S ribosomal protein L36a OS=Homo sapiens GN=Rpl36a PE=1 SV=2 | 60S ribosomal protein L6 OS=Homo sapiens GN=Rpl6 PE=1 SV=3 |
| 60S ribosomal protein L5 (Fragment) OS=Homo sapiens GN=Rpl5 PE=1 SV=1 | 60S ribosomal protein L6 OS=Homo sapiens GN=Rpl6 PE=2 SV=1 |
| 60S ribosomal protein L5 OS=Homo sapiens GN=Rpl5 PE=1 SV=3 | 60S ribosomal protein L7 (Fragment) OS=Homo sapiens GN=Rpl7 PE=1 SV=1 |
| 60S ribosomal protein L6 (Fragment) OS=Homo sapiens GN=Rpl6 PE=1 SV=1 | 60S ribosomal protein L7 OS=Homo sapiens GN=Rpl7 PE=1 SV=2 |
| 60S ribosomal protein L6 OS=Homo sapiens GN=Rpl6 PE=1 SV=3 | 60S ribosomal protein L7a OS=Homo sapiens GN=Rpl7a PE=1 SV=2 |
| 60S ribosomal protein L6 OS=Homo sapiens GN=Rpl6 PE=2 SV=1 | 60S ribosomal protein L8 OS=Homo sapiens GN=Rpl8 PE=1 SV=2 |
| 60S ribosomal protein L7 (Fragment) OS=Homo sapiens GN=Rpl7 PE=1 SV=1 | 60S ribosomal protein L9 (Fragment) OS=Homo sapiens GN=Rpl9 PE=1 SV=1 |
| 60S ribosomal protein L7 OS=Homo sapiens GN=Rpl7 PE=1 SV=2 | 60S ribosomal protein L9 OS=Homo sapiens GN=Rpl9 PE=1 SV=1 |
| 60S ribosomal protein L7a OS=Homo sapiens GN=Rpl7a PE=1 SV=2 | 60S ribosomal protein L9 OS=Homo sapiens GN=Rpl9 PE=1 SV=2 |
| 60S ribosomal protein L8 OS=Homo sapiens GN=Rpl8 PE=1 SV=2 | 78 kDa glucose-regulated protein OS=Homo sapiens GN=Hspa5 PE=1 SV=3 |
| 60S ribosomal protein L9 (Fragment) OS=Homo sapiens GN=Rpl9 PE=1 SV=1 | Acin1 protein OS=Homo sapiens GN=Acin1 PE=1 SV=1 |
| 60S ribosomal protein L9 OS=Homo sapiens GN=Rpl9 PE=1 SV=1 | Actg2 protein OS=Homo sapiens GN=Actg2 PE=2 SV=1 |
| 60S ribosomal protein L9 OS=Homo sapiens GN=Rpl9 PE=1 SV=2 | Actin, alpha cardiac muscle 1 (Fragment) OS=Homo sapiens GN=Actc1 PE=4 SV=1 |
| 78 kDa glucose-regulated protein OS=Homo sapiens GN=Hspa5 PE=1 SV=3 | Actin, alpha cardiac muscle 1 OS=Homo sapiens GN=Actc1 PE=1 SV=1 |
| Acetyl-CoA carboxylase 1 (Fragment) OS=Homo sapiens GN=Acaca PE=1 SV=1 | Actin, alpha skeletal muscle (Fragment) OS=Homo sapiens GN=Acta1 PE=3 SV=1 |
| Acetyl-CoA carboxylase 1 OS=Homo sapiens GN=Acaca PE=1 SV=1 | Actin, alpha skeletal muscle OS=Homo sapiens GN=Acta1 PE=1 SV=1 |
| Acetyl-CoA carboxylase 2 (Fragment) OS=Homo sapiens GN=Acacb PE=1 SV=1 | Actin, aortic smooth muscle OS=Homo sapiens GN=Acta2 PE=1 SV=1 |
| Acetyl-CoA carboxylase 2 OS=Homo sapiens GN=Acacb PE=1 SV=1 | Actin, cytoplasmic 1 (Fragment) OS=Homo sapiens GN=Actb PE=1 SV=1 |
| Acetyl-CoA carboxylase 280 (Fragment) OS=Homo sapiens GN=Acacb PE=2 SV=2 | Actin, cytoplasmic 1 OS=Homo sapiens GN=Actb PE=1 SV=1 |
| Actg2 protein OS=Homo sapiens GN=Actg2 PE=2 SV=1 | Actin, cytoplasmic 2 (Fragment) OS=Homo sapiens GN=Actg1 PE=1 SV=1 |
| Actin, alpha cardiac muscle 1 (Fragment) OS=Homo sapiens GN=Actc1 PE=4 SV=1 | Actin, cytoplasmic 2 OS=Homo sapiens GN=Actg1 PE=1 SV=1 |
| Actin, alpha cardiac muscle 1 OS=Homo sapiens GN=Actc1 PE=1 SV=1 | Actin, gamma-enteric smooth muscle (Fragment) OS=Homo sapiens GN=Actg2 PE=1 SV=1 |
| Actin, alpha skeletal muscle (Fragment) OS=Homo sapiens GN=Acta1 PE=3 SV=1 | Actin, gamma-enteric smooth muscle (Fragment) OS=Homo sapiens GN=Actg2 PE=1 SV=2 |
| Actin, alpha skeletal muscle OS=Homo sapiens GN=Acta1 PE=1 SV=1 | Actin, gamma-enteric smooth muscle (Fragment) OS=Homo sapiens GN=Actg2 PE=3 SV=1 |
| Actin, aortic smooth muscle OS=Homo sapiens GN=Acta2 PE=1 SV=1 | Actin, gamma-enteric smooth muscle OS=Homo sapiens GN=Actg2 PE=1 SV=1 |
| Actin, cytoplasmic 1 (Fragment) OS=Homo sapiens GN=Actb PE=1 SV=1 | Activated RNA polymerase II transcriptional coactivator p15 OS=Homo sapiens GN=Sub1 PE=1 SV=3 |
| Actin, cytoplasmic 1 OS=Homo sapiens GN=Actb PE=1 SV=1 | ADP/ATP translocase 1 OS=Homo sapiens GN=Slc25a4 PE=1 SV=4 |
| Actin, cytoplasmic 2 (Fragment) OS=Homo sapiens GN=Actg1 PE=1 SV=1 | ADP/ATP translocase 2 OS=Homo sapiens GN=Slc25a5 PE=1 SV=3 |
| Actin, cytoplasmic 2 OS=Homo sapiens GN=Actg1 PE=1 SV=1 | Albumin 1 OS=Homo sapiens GN=Alb PE=1 SV=1 |
| Actin, gamma-enteric smooth muscle (Fragment) OS=Homo sapiens GN=Actg2 PE=1 SV=1 | Alpha-actin (AA 27-375) (Fragment) OS=Homo sapiens GN=Actc1 PE=2 SV=1 |
| Actin, gamma-enteric smooth muscle (Fragment) OS=Homo sapiens GN=Actg2 PE=1 SV=2 | Alpha-actin (Aa 40-375) (Fragment) OS=Homo sapiens GN=Acta1 PE=2 SV=1 |
| Actin, gamma-enteric smooth muscle (Fragment) OS=Homo sapiens GN=Actg2 PE=3 SV=1 | Alpha-internexin OS=Homo sapiens GN=Ina PE=1 SV=3 |
| Actin, gamma-enteric smooth muscle OS=Homo sapiens GN=Actg2 PE=1 SV=1 | Alpha-tubulin (Fragment) OS=Homo sapiens GN=Tuba1b PE=2 SV=1 |
| Activated RNA polymerase II transcriptional coactivator p15 OS=Homo sapiens GN=Sub1 PE=1 SV=3 | Aly/REF export factor 2 OS=Homo sapiens GN=Alyref2 PE=1 SV=1 |
| ADP/ATP translocase 1 OS=Homo sapiens GN=Slc25a4 PE=1 SV=4 | Apoptotic chromatin condensation inducer 1 OS=Homo sapiens GN=Acin1 PE=2 SV=1 |
| ADP/ATP translocase 2 OS=Homo sapiens GN=Slc25a5 PE=1 SV=3 | Apoptotic chromatin condensation inducer in the nucleus (Fragment) OS=Homo sapiens GN=Acin1 PE=1 SV=1 |
| Albumin 1 OS=Homo sapiens GN=Alb PE=1 SV=1 | Apoptotic chromatin condensation inducer in the nucleus OS=Homo sapiens GN=Acin1 PE=1 SV=1 |
| Alpha-actin (AA 27-375) (Fragment) OS=Homo sapiens GN=Actc1 PE=2 SV=1 | Apoptotic chromatin condensation inducer in the nucleus OS=Homo sapiens GN=Acin1 PE=1 SV=3 |
| Alpha-actin (Aa 40-375) (Fragment) OS=Homo sapiens GN=Acta1 PE=2 SV=1 | Arginine--tRNA ligase, cytoplasmic OS=Homo sapiens GN=Rars PE=1 SV=2 |
| Alpha-tubulin (Fragment) OS=Homo sapiens GN=Tuba1b PE=2 SV=1 | Argonaute RISC catalytic component 2 OS=Homo sapiens GN=Ago2 PE=1 SV=1 |
| Aly/REF export factor 2 OS=Homo sapiens GN=Alyref2 PE=1 SV=1 | Argonaute RISC catalytic component 2 OS=Homo sapiens GN=Ago2 PE=1 SV=3 |
| AP-3 complex subunit delta-1 OS=Homo sapiens GN=Ap3d1 PE=1 SV=1 | ATP synthase subunit O, mitochondrial (Fragment) OS=Homo sapiens GN=Atp5o PE=1 SV=1 |
| Ap3d1 protein (Fragment) OS=Homo sapiens GN=Ap3d1 PE=2 SV=1 | ATP synthase subunit O, mitochondrial (Fragment) OS=Homo sapiens GN=Atp5o PE=1 SV=8 |
| Arf-GAP with SH3 domain, ANK repeat and PH domain-containing protein 2 OS=Homo sapiens GN=Asap2 PE=1 SV=1 | ATP synthase subunit O, mitochondrial OS=Homo sapiens GN=Atp5o PE=1 SV=1 |
| Arf-GAP with SH3 domain, ANK repeat and PH domain-containing protein 2 OS=Homo sapiens GN=Asap2 PE=1 SV=2 | ATP-dependent RNA helicase DDX3Y OS=Homo sapiens GN=Ddx3y PE=1 SV=2 |
| Arf-GAP with SH3 domain, ANK repeat and PH domain-containing protein 2 OS=Homo sapiens GN=Asap2 PE=1 SV=3 | A-X actin OS=Homo sapiens GN=Actb PE=2 SV=1 |
| ATP synthase subunit O, mitochondrial (Fragment) OS=Homo sapiens GN=Atp5o PE=1 SV=8 | Bcl-2-associated transcription factor 1 (Fragment) OS=Homo sapiens GN=Bclaf1 PE=1 SV=1 |
| ATP synthase subunit O, mitochondrial OS=Homo sapiens GN=Atp5o PE=1 SV=1 | Bcl-2-associated transcription factor 1 OS=Homo sapiens GN=Bclaf1 PE=1 SV=1 |
| ATP-dependent RNA helicase A OS=Homo sapiens GN=Dhx9 PE=1 SV=1 | Bcl-2-associated transcription factor 1 OS=Homo sapiens GN=Bclaf1 PE=1 SV=2 |
| ATP-dependent RNA helicase A OS=Homo sapiens GN=Dhx9 PE=1 SV=2 | Beta-actin (Fragment) OS=Homo sapiens GN=Actb PE=2 SV=1 |
| ATP-dependent RNA helicase DDX3Y OS=Homo sapiens GN=Ddx3y PE=1 SV=2 | Beta-actin FE-3 (Fragment) OS=Homo sapiens GN=Actb PE=2 SV=1 |
| A-X actin OS=Homo sapiens GN=Actb PE=2 SV=1 | Beta-actin-like protein 2 OS=Homo sapiens GN=Actbl2 PE=1 SV=1 |
| Band 4.1-like protein 2 (Fragment) OS=Homo sapiens GN=Epb41l2 PE=1 SV=1 | Beta-tropomyosin OS=Homo sapiens GN=Tpm2 PE=2 SV=1 |
| Band 4.1-like protein 2 OS=Homo sapiens GN=Epb41l2 PE=1 SV=1 | C. elegans ceh-10 homeo domain containing homolog, isoform CRA_a OS=Homo sapiens GN=Vsx2 PE=2 SV=1 |
| Band 4.1-like protein 2 OS=Homo sapiens GN=Epb41l2 PE=1 SV=2 | C. elegans ceh-10 homeo domain containing homolog, isoform CRA_b OS=Homo sapiens GN=Vsx2 PE=2 SV=2 |
| Band 4.1-like protein 3 (Fragment) OS=Homo sapiens GN=Epb41l3 PE=1 SV=1 | Caprin-1 (Fragment) OS=Homo sapiens GN=Caprin1 PE=1 SV=1 |
| Band 4.1-like protein 3 OS=Homo sapiens GN=Epb41l3 PE=1 SV=1 | Caprin-1 OS=Homo sapiens GN=Caprin1 PE=1 SV=2 |
| Beta-actin (Fragment) OS=Homo sapiens GN=Actb PE=2 SV=1 | CArG-binding factor A (Fragment) OS=Homo sapiens GN=Hnrnpab PE=2 SV=1 |
| Beta-actin FE-3 (Fragment) OS=Homo sapiens GN=Actb PE=2 SV=1 | Caveolae-associated protein 1 OS=Homo sapiens GN=Cavin1 PE=1 SV=1 |
| Beta-actin-like protein 2 OS=Homo sapiens GN=Actbl2 PE=1 SV=1 | Cdc2a protein (Fragment) OS=Homo sapiens GN=Cdk1 PE=2 SV=1 |
| Bifunctional glutamate/proline--tRNA ligase OS=Homo sapiens GN=Eprs PE=1 SV=4 | Cell cycle p34 CDC2 kinase protein (Fragment) OS=Homo sapiens PE=4 SV=1 |
| Brain cDNA, clone MNCb-1272, similar to Homo sapiens chaperonin subunit 2 (beta) (Cct2), mRNA OS=Homo sapiens GN=Cct2 PE=2 SV=1 | Cell division cycle 5-like protein OS=Homo sapiens GN=Cdc5l PE=1 SV=2 |
| C. elegans ceh-10 homeo domain containing homolog, isoform CRA_a OS=Homo sapiens GN=Vsx2 PE=2 SV=1 | Cell growth-regulating nucleolar protein (Fragment) OS=Homo sapiens GN=Lyar PE=1 SV=1 |
| C. elegans ceh-10 homeo domain containing homolog, isoform CRA_b OS=Homo sapiens GN=Vsx2 PE=2 SV=2 | Cell growth-regulating nucleolar protein OS=Homo sapiens GN=Lyar PE=1 SV=2 |
| Caprin-1 OS=Homo sapiens GN=Caprin1 PE=1 SV=2 | Chromodomain-helicase-DNA-binding protein 2 (Fragment) OS=Homo sapiens GN=Chd2 PE=1 SV=1 |
| Caveolae-associated protein 1 OS=Homo sapiens GN=Cavin1 PE=1 SV=1 | Chromodomain-helicase-DNA-binding protein 2 (Fragment) OS=Homo sapiens GN=Chd2 PE=1 SV=2 |
| Cdc2a protein (Fragment) OS=Homo sapiens GN=Cdk1 PE=2 SV=1 | Chromodomain-helicase-DNA-binding protein 2 OS=Homo sapiens GN=Chd2 PE=1 SV=1 |
| Cell cycle p34 CDC2 kinase protein (Fragment) OS=Homo sapiens PE=4 SV=1 | Cleavage and polyadenylation specificity factor subunit 5 OS=Homo sapiens GN=Nudt21 PE=1 SV=1 |
| Cell division cycle 5-like protein OS=Homo sapiens GN=Cdc5l PE=1 SV=2 | Cleavage and polyadenylation-specificity factor subunit 5 (Fragment) OS=Homo sapiens GN=Nudt21 PE=1 SV=1 |
| Chaperonin subunit 2 (Beta), isoform CRA_a OS=Homo sapiens GN=Cct2 PE=1 SV=1 | Cleavage and polyadenylation-specificity factor subunit 5 OS=Homo sapiens GN=Nudt21 PE=1 SV=1 |
| Chromodomain helicase DNA-binding protein 3 (Fragment) OS=Homo sapiens GN=Chd3 PE=1 SV=1 | Coatomer subunit alpha OS=Homo sapiens GN=Copa PE=1 SV=1 |
| Chromodomain helicase DNA-binding protein 3 OS=Homo sapiens GN=Chd3 PE=1 SV=1 | Coatomer subunit alpha OS=Homo sapiens GN=Copa PE=1 SV=2 |
| Chromodomain-helicase-DNA-binding protein 4 OS=Homo sapiens GN=Chd4 PE=1 SV=1 | Coatomer subunit alpha OS=Homo sapiens GN=Copa PE=2 SV=1 |
| Cleavage and polyadenylation specificity factor subunit 5 OS=Homo sapiens GN=Nudt21 PE=1 SV=1 | Copa protein (Fragment) OS=Homo sapiens GN=Copa PE=2 SV=1 |
| Cleavage and polyadenylation-specificity factor subunit 5 (Fragment) OS=Homo sapiens GN=Nudt21 PE=1 SV=1 | Csda protein OS=Homo sapiens GN=Ybx3 PE=2 SV=1 |
| Cleavage and polyadenylation-specificity factor subunit 5 OS=Homo sapiens GN=Nudt21 PE=1 SV=1 | Cyclin-dependent kinase 1 (Fragment) OS=Homo sapiens GN=Cdk1 PE=1 SV=1 |
| Coatomer subunit gamma-1 OS=Homo sapiens GN=Copg1 PE=1 SV=1 | Cyclin-dependent kinase 1 OS=Homo sapiens GN=Cdk1 PE=1 SV=3 |
| Csda protein OS=Homo sapiens GN=Ybx3 PE=2 SV=1 | Cyclin-dependent kinase 12 OS=Homo sapiens GN=Cdk12 PE=1 SV=2 |
| Cyclin-dependent kinase 1 (Fragment) OS=Homo sapiens GN=Cdk1 PE=1 SV=1 | Cyclin-dependent kinase 13 OS=Homo sapiens GN=Cdk13 PE=1 SV=1 |
| Cyclin-dependent kinase 1 OS=Homo sapiens GN=Cdk1 PE=1 SV=3 | Cyclin-dependent kinase 13 OS=Homo sapiens GN=Cdk13 PE=1 SV=3 |
| Cyclin-dependent kinase 12 OS=Homo sapiens GN=Cdk12 PE=1 SV=2 | Cyclin-dependent kinase 14 OS=Homo sapiens GN=Cdk14 PE=1 SV=1 |
| Cyclin-dependent kinase 13 OS=Homo sapiens GN=Cdk13 PE=1 SV=1 | Cyclin-dependent kinase 14 OS=Homo sapiens GN=Cdk14 PE=1 SV=2 |
| Cyclin-dependent kinase 13 OS=Homo sapiens GN=Cdk13 PE=1 SV=3 | Cyclin-dependent kinase 15 OS=Homo sapiens GN=Cdk15 PE=1 SV=1 |
| Cyclin-dependent kinase 14 OS=Homo sapiens GN=Cdk14 PE=1 SV=1 | Cyclin-dependent kinase 15 OS=Homo sapiens GN=Cdk15 PE=2 SV=2 |
| Cyclin-dependent kinase 14 OS=Homo sapiens GN=Cdk14 PE=1 SV=2 | Cyclin-dependent kinase 17 (Fragment) OS=Homo sapiens GN=Cdk17 PE=1 SV=1 |
| Cyclin-dependent kinase 15 OS=Homo sapiens GN=Cdk15 PE=1 SV=1 | Cyclin-dependent kinase 17 OS=Homo sapiens GN=Cdk17 PE=1 SV=2 |
| Cyclin-dependent kinase 15 OS=Homo sapiens GN=Cdk15 PE=2 SV=2 | Cyclin-dependent kinase 2, isoform CRA_b OS=Homo sapiens GN=Cdk2 PE=2 SV=1 |
| Cyclin-dependent kinase 17 (Fragment) OS=Homo sapiens GN=Cdk17 PE=1 SV=1 | Cyclin-dependent kinase 20 (Fragment) OS=Homo sapiens GN=Cdk20 PE=1 SV=1 |
| Cyclin-dependent kinase 17 OS=Homo sapiens GN=Cdk17 PE=1 SV=2 | Cyclin-dependent kinase 20 (Fragment) OS=Homo sapiens GN=Cdk20 PE=4 SV=1 |
| Cyclin-dependent kinase 2, isoform CRA_b OS=Homo sapiens GN=Cdk2 PE=2 SV=1 | Cyclin-dependent kinase 20 OS=Homo sapiens GN=Cdk20 PE=1 SV=1 |
| Cyclin-dependent kinase 20 (Fragment) OS=Homo sapiens GN=Cdk20 PE=1 SV=1 | Cyclin-dependent kinase 3 OS=Homo sapiens GN=Cdk3 PE=1 SV=2 |
| Cyclin-dependent kinase 20 (Fragment) OS=Homo sapiens GN=Cdk20 PE=4 SV=1 | Cyclin-dependent kinase 4 (Fragment) OS=Homo sapiens GN=Cdk4 PE=1 SV=1 |
| Cyclin-dependent kinase 20 OS=Homo sapiens GN=Cdk20 PE=1 SV=1 | Cyclin-dependent kinase 4 OS=Homo sapiens GN=Cdk4 PE=1 SV=1 |
| Cyclin-dependent kinase 3 OS=Homo sapiens GN=Cdk3 PE=1 SV=2 | Cyclin-dependent kinase 5, isoform CRA_c OS=Homo sapiens GN=Cdk5 PE=1 SV=1 |
| Cyclin-dependent kinase 4 (Fragment) OS=Homo sapiens GN=Cdk4 PE=1 SV=1 | Cyclin-dependent kinase 6 OS=Homo sapiens GN=Cdk6 PE=1 SV=2 |
| Cyclin-dependent kinase 4 OS=Homo sapiens GN=Cdk4 PE=1 SV=1 | Cyclin-dependent kinase 9 OS=Homo sapiens GN=Cdk9 PE=1 SV=1 |
| Cyclin-dependent kinase 5, isoform CRA_c OS=Homo sapiens GN=Cdk5 PE=1 SV=1 | Cyclin-dependent-like kinase 5 OS=Homo sapiens GN=Cdk5 PE=1 SV=1 |
| Cyclin-dependent kinase 6 OS=Homo sapiens GN=Cdk6 PE=1 SV=2 | Cytokeratin KRT2-6HF (Fragment) OS=Homo sapiens GN=Krt75 PE=2 SV=1 |
| Cyclin-dependent kinase 9 OS=Homo sapiens GN=Cdk9 PE=1 SV=1 | Cytoskeletal beta-actin (Fragment) OS=Homo sapiens GN=Actb PE=2 SV=1 |
| Cyclin-dependent-like kinase 5 OS=Homo sapiens GN=Cdk5 PE=1 SV=1 | Cytoskeleton-associated protein 4 OS=Homo sapiens GN=Ckap4 PE=1 SV=2 |
| Cytokeratin KRT2-6HF (Fragment) OS=Homo sapiens GN=Krt75 PE=2 SV=1 | DbpA murine homologue OS=Homo sapiens GN=Ybx3 PE=2 SV=1 |
| Cytoskeletal beta-actin (Fragment) OS=Homo sapiens GN=Actb PE=2 SV=1 | Ddx3x protein OS=Homo sapiens GN=Ddx3x PE=2 SV=1 |
| Cytoskeleton-associated protein 4 OS=Homo sapiens GN=Ckap4 PE=1 SV=2 | Ddx5 protein (Fragment) OS=Homo sapiens GN=Ddx5 PE=2 SV=1 |
| DbpA murine homologue OS=Homo sapiens GN=Ybx3 PE=2 SV=1 | DEAD (Asp-Glu-Ala-Asp) box polypeptide 17, isoform CRA_a OS=Homo sapiens GN=Ddx17 PE=1 SV=1 |
| Ddx3x protein OS=Homo sapiens GN=Ddx3x PE=2 SV=1 | DEAD (Asp-Glu-Ala-Asp) box polypeptide 5 OS=Homo sapiens GN=Ddx5 PE=1 SV=1 |
| Ddx5 protein (Fragment) OS=Homo sapiens GN=Ddx5 PE=2 SV=1 | DEAD (Asp-Glu-Ala-Asp) box polypeptide 5 OS=Homo sapiens GN=Ddx5 PE=2 SV=1 |
| DEAD (Asp-Glu-Ala-Asp) box polypeptide 17 OS=Homo sapiens GN=Ddx17 PE=2 SV=2 | Description |
| DEAD (Asp-Glu-Ala-Asp) box polypeptide 17, isoform CRA_a OS=Homo sapiens GN=Ddx17 PE=1 SV=1 | Desmin (Fragment) OS=Homo sapiens GN=des PE=4 SV=1 |
| DEAD (Asp-Glu-Ala-Asp) box polypeptide 5 OS=Homo sapiens GN=Ddx5 PE=1 SV=1 | Desmin OS=Homo sapiens GN=Des PE=1 SV=3 |
| DEAD (Asp-Glu-Ala-Asp) box polypeptide 5 OS=Homo sapiens GN=Ddx5 PE=2 SV=1 | Developmentally regulated GTP binding protein 1 OS=Homo sapiens GN=Drg1 PE=2 SV=1 |
| Dedicator of cytokinesis protein 7 (Fragment) OS=Homo sapiens GN=Dock7 PE=1 SV=1 | Developmentally-regulated GTP-binding protein 2 OS=Homo sapiens GN=Drg2 PE=1 SV=1 |
| Dedicator of cytokinesis protein 7 (Fragment) OS=Homo sapiens GN=Dock7 PE=1 SV=2 | DNA (cytosine-5)-methyltransferase 1 OS=Homo sapiens GN=Dnmt1 PE=1 SV=5 |
| Dedicator of cytokinesis protein 7 OS=Homo sapiens GN=Dock7 PE=1 SV=1 | DNA (cytosine-5)-methyltransferase OS=Homo sapiens GN=Dnmt1 PE=2 SV=1 |
| Dedicator of cytokinesis protein 7 OS=Homo sapiens GN=Dock7 PE=1 SV=2 | DNA-(apurinic or apyrimidinic site) lyase (Fragment) OS=Homo sapiens GN=Apex1 PE=1 SV=1 |
| Dedicator of cytokinesis protein 7 OS=Homo sapiens GN=Dock7 PE=1 SV=3 | DNA-(apurinic or apyrimidinic site) lyase OS=Homo sapiens GN=Apex1 PE=1 SV=1 |
| Description | Eef2 protein (Fragment) OS=Homo sapiens GN=Eef2 PE=2 SV=1 |
| Desmin (Fragment) OS=Homo sapiens GN=des PE=4 SV=1 | EG620155 protein OS=Homo sapiens GN=Gm6133 PE=2 SV=1 |
| Desmin OS=Homo sapiens GN=Des PE=1 SV=3 | EG627828 protein OS=Homo sapiens GN=Gm6793 PE=1 SV=1 |
| Developmentally regulated GTP binding protein 1 OS=Homo sapiens GN=Drg1 PE=2 SV=1 | Eif2s2 protein OS=Homo sapiens GN=Eif2s2 PE=1 SV=1 |
| DNA-(apurinic or apyrimidinic site) lyase (Fragment) OS=Homo sapiens GN=Apex1 PE=1 SV=1 | Eif2s3x protein (Fragment) OS=Homo sapiens GN=Eif2s3x PE=2 SV=1 |
| DNA-(apurinic or apyrimidinic site) lyase OS=Homo sapiens GN=Apex1 PE=1 SV=1 | Eif5b protein OS=Homo sapiens GN=Eif5b PE=2 SV=1 |
| E3 ubiquitin-protein ligase TRIP12 (Fragment) OS=Homo sapiens GN=Trip12 PE=1 SV=1 | Elongation factor 1-alpha 1 (Fragment) OS=Homo sapiens GN=Eef1a1 PE=1 SV=1 |
| E3 ubiquitin-protein ligase TRIP12 OS=Homo sapiens GN=Trip12 PE=1 SV=1 | Elongation factor 1-alpha 2 OS=Homo sapiens GN=Eef1a2 PE=1 SV=1 |
| E3 UFM1-protein ligase 1 OS=Homo sapiens GN=Ufl1 PE=1 SV=2 | Elongation factor 1-alpha OS=Homo sapiens GN=Eef1a1 PE=1 SV=1 |
| Eef2 protein (Fragment) OS=Homo sapiens GN=Eef2 PE=2 SV=1 | Elongation factor 1-alpha OS=Homo sapiens GN=Eef1a1 PE=2 SV=1 |
| EG620155 protein OS=Homo sapiens GN=Gm6133 PE=2 SV=1 | Elongation factor 2 (Fragment) OS=Homo sapiens GN=Eef2 PE=2 SV=1 |
| Eif1a protein OS=Homo sapiens GN=Eif1a PE=2 SV=1 | Elongation factor 2 OS=Homo sapiens GN=Eef2 PE=1 SV=2 |
| Eif2s2 protein OS=Homo sapiens GN=Eif2s2 PE=1 SV=1 | Epiplakin OS=Homo sapiens GN=Eppk1 PE=1 SV=2 |
| Eif2s3x protein (Fragment) OS=Homo sapiens GN=Eif2s3x PE=2 SV=1 | Eukaryotic translation initiation factor 2 subunit 1 OS=Homo sapiens GN=Eif2s1 PE=1 SV=3 |
| Eif5b protein OS=Homo sapiens GN=Eif5b PE=2 SV=1 | Eukaryotic translation initiation factor 2 subunit 2 (Fragment) OS=Homo sapiens GN=Eif2s2 PE=1 SV=1 |
| Elongation factor 1-alpha 1 (Fragment) OS=Homo sapiens GN=Eef1a1 PE=1 SV=1 | Eukaryotic translation initiation factor 2 subunit 3, X-linked OS=Homo sapiens GN=Eif2s3x PE=1 SV=1 |
| Elongation factor 1-alpha 2 OS=Homo sapiens GN=Eef1a2 PE=1 SV=1 | Eukaryotic translation initiation factor 2 subunit 3, Y-linked OS=Homo sapiens GN=Eif2s3y PE=1 SV=2 |
| Elongation factor 1-alpha OS=Homo sapiens GN=Eef1a1 PE=1 SV=1 | Eukaryotic translation initiation factor 2, subunit 3, structural gene X-linked OS=Homo sapiens GN=Eif2s3x PE=1 SV=1 |
| Elongation factor 1-alpha OS=Homo sapiens GN=Eef1a1 PE=2 SV=1 | Eukaryotic translation initiation factor 2, subunit 3, structural gene Y-linked, isoform CRA_b OS=Homo sapiens GN=Eif2s3y PE=2 SV=1 |
| Elongation factor 2 (Fragment) OS=Homo sapiens GN=Eef2 PE=2 SV=1 | Eukaryotic translation initiation factor 3 subunit A (Fragment) OS=Homo sapiens GN=Eif3a PE=2 SV=1 |
| Elongation factor 2 OS=Homo sapiens GN=Eef2 PE=1 SV=2 | Eukaryotic translation initiation factor 3 subunit A OS=Homo sapiens GN=Eif3a PE=1 SV=5 |
| Epb4.1l2 protein (Fragment) OS=Homo sapiens GN=Epb41l2 PE=2 SV=1 | Eukaryotic translation initiation factor 5B OS=Homo sapiens GN=Eif5b PE=1 SV=2 |
| Epb4.1l2 protein OS=Homo sapiens GN=Epb41l2 PE=2 SV=1 | Ewing sarcoma breakpoint region 1 OS=Homo sapiens GN=Ewsr1 PE=2 SV=1 |
| Epb4.1l3 protein (Fragment) OS=Homo sapiens GN=Epb41l3 PE=2 SV=1 | Fast skeletal muscle SR calcium ATPase OS=Homo sapiens GN=Atp2a1 PE=2 SV=2 |
| Epiplakin OS=Homo sapiens GN=Eppk1 PE=1 SV=2 | Fc fragment of IgG-binding protein OS=Homo sapiens GN=Fcgbp PE=1 SV=1 |
| Erythrocyte protein band 4.1-like 2 OS=Homo sapiens GN=Epb41l2 PE=2 SV=1 | FGFR2 OS=Homo sapiens GN=Fgfr2 PE=2 SV=1 |
| Erythrocyte protein band 4.1-like 3 isoform B OS=Homo sapiens GN=Epb41l3 PE=2 SV=1 | Fibroblast growth factor receptor 1 OS=Homo sapiens GN=Fgfr1 PE=1 SV=2 |
| Erythrocyte protein band 4.1-like 3 isoform C OS=Homo sapiens GN=Epb41l3 PE=2 SV=1 | Fibroblast growth factor receptor 2 OS=Homo sapiens GN=Fgfr2 PE=1 SV=1 |
| Erythrocyte protein band 4.1-like 3 OS=Homo sapiens GN=Epb41l3 PE=2 SV=1 | Fibroblast growth factor receptor 2 OS=Homo sapiens GN=Fgfr2 PE=1 SV=4 |
| Eukaryotic translation initiation factor 1A, X-chromosomal OS=Homo sapiens GN=Eif1ax PE=2 SV=3 | Fibroblast growth factor receptor 3 (Fragment) OS=Homo sapiens GN=Fgfr3 PE=1 SV=1 |
| Eukaryotic translation initiation factor 2 subunit 1 OS=Homo sapiens GN=Eif2s1 PE=1 SV=3 | Fibroblast growth factor receptor 3 OS=Homo sapiens GN=Fgfr3 PE=1 SV=1 |
| Eukaryotic translation initiation factor 2 subunit 2 (Fragment) OS=Homo sapiens GN=Eif2s2 PE=1 SV=1 | Fibroblast growth factor receptor 4 OS=Homo sapiens GN=Fgfr4 PE=1 SV=3 |
| Eukaryotic translation initiation factor 2 subunit 3, X-linked OS=Homo sapiens GN=Eif2s3x PE=1 SV=1 | Fibroblast growth factor receptor OS=Homo sapiens GN=Fgfr1 PE=1 SV=1 |
| Eukaryotic translation initiation factor 2 subunit 3, Y-linked OS=Homo sapiens GN=Eif2s3y PE=1 SV=2 | Fibroblast growth factor receptor OS=Homo sapiens GN=Fgfr1 PE=2 SV=1 |
| Eukaryotic translation initiation factor 2, subunit 3, structural gene X-linked OS=Homo sapiens GN=Eif2s3x PE=1 SV=1 | Fibroblast growth factor receptor OS=Homo sapiens GN=Fgfr2 PE=1 SV=1 |
| Eukaryotic translation initiation factor 2, subunit 3, structural gene Y-linked, isoform CRA_b OS=Homo sapiens GN=Eif2s3y PE=2 SV=1 | Fibroblast growth factor receptor OS=Homo sapiens GN=Fgfr2 PE=2 SV=1 |
| Eukaryotic translation initiation factor 5B OS=Homo sapiens GN=Eif5b PE=1 SV=2 | Fibroblast growth factor receptor OS=Homo sapiens GN=Fgfr3 PE=1 SV=1 |
| Ewing sarcoma breakpoint region 1 OS=Homo sapiens GN=Ewsr1 PE=2 SV=1 | Fibroblast growth factor receptor OS=Homo sapiens GN=Fgfr3 PE=1 SV=2 |
| Expressed sequence BB287469 OS=Homo sapiens GN=BB287469 PE=2 SV=1 | Fibroblast growth factor receptor OS=Homo sapiens GN=Fgfr3 PE=2 SV=1 |
| Ezrin OS=Homo sapiens GN=Ezr PE=1 SV=1 | Fibroblast growth factor receptor OS=Homo sapiens GN=Fgfr4 PE=3 SV=1 |
| Fc fragment of IgG-binding protein OS=Homo sapiens GN=Fcgbp PE=1 SV=1 | Fibronectin OS=Homo sapiens GN=Fn1 PE=1 SV=1 |
| FGFR2 OS=Homo sapiens GN=Fgfr2 PE=2 SV=1 | Fibronectin OS=Homo sapiens GN=Fn1 PE=1 SV=4 |
| Fibroblast growth factor receptor 1 OS=Homo sapiens GN=Fgfr1 PE=1 SV=2 | Flap endonuclease 1 OS=Homo sapiens GN=Fen1 PE=1 SV=1 |
| Fibroblast growth factor receptor 2 OS=Homo sapiens GN=Fgfr2 PE=1 SV=1 | Flap endonuclease 1 OS=Homo sapiens GN=Fen1 PE=2 SV=1 |
| Fibroblast growth factor receptor 2 OS=Homo sapiens GN=Fgfr2 PE=1 SV=4 | Flap endonuclease 1 OS=Homo sapiens GN=Fen1 PE=3 SV=1 |
| Fibroblast growth factor receptor 3 (Fragment) OS=Homo sapiens GN=Fgfr3 PE=1 SV=1 | Fragile X mental retardation protein FMRP OS=Homo sapiens GN=Fmr1 PE=2 SV=1 |
| Fibroblast growth factor receptor 3 OS=Homo sapiens GN=Fgfr3 PE=1 SV=1 | Fragile X mental retardation syndrome 1 homolog OS=Homo sapiens GN=Fmr1 PE=1 SV=1 |
| Fibroblast growth factor receptor 4 OS=Homo sapiens GN=Fgfr4 PE=1 SV=3 | Fragile X mental retardation syndrome-related protein 1 OS=Homo sapiens GN=Fxr1 PE=1 SV=1 |
| Fibroblast growth factor receptor OS=Homo sapiens GN=Fgfr1 PE=1 SV=1 | Fragile X mental retardation syndrome-related protein 1 OS=Homo sapiens GN=Fxr1 PE=1 SV=2 |
| Fibroblast growth factor receptor OS=Homo sapiens GN=Fgfr1 PE=2 SV=1 | Fragile X mental retardation syndrome-related protein 2 OS=Homo sapiens GN=Fxr2 PE=1 SV=1 |
| Fibroblast growth factor receptor OS=Homo sapiens GN=Fgfr2 PE=1 SV=1 | Fusion, derived from t(12;16) malignant liposarcoma (Human), isoform CRA_a OS=Homo sapiens GN=Fus PE=1 SV=1 |
| Fibroblast growth factor receptor OS=Homo sapiens GN=Fgfr2 PE=2 SV=1 | Fusion, derived from t(1216) malignant liposarcoma (Human) OS=Homo sapiens GN=Fus PE=1 SV=1 |
| Fibroblast growth factor receptor OS=Homo sapiens GN=Fgfr3 PE=1 SV=1 | Gag OS=Homo sapiens GN=gag PE=4 SV=2 |
| Fibroblast growth factor receptor OS=Homo sapiens GN=Fgfr3 PE=1 SV=2 | Gag polyprotein OS=Homo sapiens PE=4 SV=1 |
| Fibroblast growth factor receptor OS=Homo sapiens GN=Fgfr3 PE=2 SV=1 | Gag polyprotein pr65 OS=Homo sapiens PE=4 SV=1 |
| Fibroblast growth factor receptor OS=Homo sapiens GN=Fgfr4 PE=3 SV=1 | Gag protein OS=Homo sapiens GN=gag PE=4 SV=1 |
| Fibronectin OS=Homo sapiens GN=Fn1 PE=1 SV=1 | Gag protein OS=Homo sapiens PE=2 SV=1 |
| Fibronectin OS=Homo sapiens GN=Fn1 PE=1 SV=4 | Gag-Pol polyprotein OS=Homo sapiens GN=Mela PE=2 SV=1 |
| Flap endonuclease 1 OS=Homo sapiens GN=Fen1 PE=1 SV=1 | Gag-pro-pol polyprotein OS=Homo sapiens GN=gag-pro-pol PE=4 SV=1 |
| Flap endonuclease 1 OS=Homo sapiens GN=Fen1 PE=2 SV=1 | Gag-pro-pol polyprotein OS=Homo sapiens PE=4 SV=1 |
| Flap endonuclease 1 OS=Homo sapiens GN=Fen1 PE=3 SV=1 | Gamma actin-like protein OS=Homo sapiens GN=Actg1 PE=2 SV=1 |
| Fragile X mental retardation protein FMRP OS=Homo sapiens GN=Fmr1 PE=2 SV=1 | Gapdh protein OS=Homo sapiens GN=Gapdh PE=2 SV=1 |
| Fragile X mental retardation syndrome 1 homolog OS=Homo sapiens GN=Fmr1 PE=1 SV=1 | Gene for histone H2a (Fragment) OS=Homo sapiens PE=4 SV=1 |
| Fragile X mental retardation syndrome-related protein 1 OS=Homo sapiens GN=Fxr1 PE=1 SV=1 | Glyceraldehyde-3-phosphate dehydrogenase (Fragment) OS=Homo sapiens GN=Gapdh PE=1 SV=1 |
| Fragile X mental retardation syndrome-related protein 1 OS=Homo sapiens GN=Fxr1 PE=1 SV=2 | Glyceraldehyde-3-phosphate dehydrogenase (Fragment) OS=Homo sapiens PE=2 SV=1 |
| Fragile X mental retardation syndrome-related protein 2 OS=Homo sapiens GN=Fxr2 PE=1 SV=1 | Glyceraldehyde-3-phosphate dehydrogenase OS=Homo sapiens GN=Gapdh PE=1 SV=1 |
| Fusion, derived from t(12;16) malignant liposarcoma (Human), isoform CRA_a OS=Homo sapiens GN=Fus PE=1 SV=1 | Glyceraldehyde-3-phosphate dehydrogenase OS=Homo sapiens GN=Gapdh PE=1 SV=2 |
| Fusion, derived from t(1216) malignant liposarcoma (Human) OS=Homo sapiens GN=Fus PE=1 SV=1 | Glyceraldehyde-3-phosphate dehydrogenase OS=Homo sapiens GN=Gapdhs PE=1 SV=1 |
| Gag polyprotein OS=Homo sapiens PE=4 SV=1 | Glyceraldehyde-3-phosphate dehydrogenase OS=Homo sapiens GN=Gm10358 PE=1 SV=1 |
| Gag polyprotein pr65 OS=Homo sapiens PE=4 SV=1 | Glyceraldehyde-3-phosphate dehydrogenase OS=Homo sapiens GN=Gm3839 PE=1 SV=1 |
| Gag protein OS=Homo sapiens GN=gag PE=4 SV=1 | Glyceraldehyde-3-phosphate dehydrogenase, testis-specific OS=Homo sapiens GN=Gapdhs PE=1 SV=1 |
| Gag protein OS=Homo sapiens PE=2 SV=1 | Glyceraldehyde-3-phosphate-dehydrogenase (Fragment) OS=Homo sapiens GN=Gapd PE=2 SV=1 |
| Gag-Pol polyprotein OS=Homo sapiens GN=Mela PE=2 SV=1 | Glyco-gag polyprotein OS=Homo sapiens PE=4 SV=1 |
| Gag-pro-pol polyprotein OS=Homo sapiens GN=gag-pro-pol PE=4 SV=1 | Golgi-associated plant pathogenesis-related protein 1 OS=Homo sapiens GN=Glipr2 PE=1 SV=1 |
| Gag-pro-pol polyprotein OS=Homo sapiens PE=4 SV=1 | Golgi-associated plant pathogenesis-related protein 1 OS=Homo sapiens GN=Glipr2 PE=1 SV=3 |
| Gamma actin-like protein OS=Homo sapiens GN=Actg1 PE=2 SV=1 | GTPase activating protein (SH3 domain) binding protein 2 OS=Homo sapiens GN=G3bp2 PE=2 SV=1 |
| Gapdh protein OS=Homo sapiens GN=Gapdh PE=2 SV=1 | GTP-binding nuclear protein Ran OS=Homo sapiens GN=1700009N14Rik PE=2 SV=1 |
| Gene for histone H2a (Fragment) OS=Homo sapiens PE=4 SV=1 | GTP-binding nuclear protein Ran OS=Homo sapiens GN=Ran PE=1 SV=3 |
| Glutamyl-prolyl-tRNA synthetase OS=Homo sapiens GN=Eprs PE=2 SV=1 | GTP-binding nuclear protein Ran OS=Homo sapiens GN=Ran PE=2 SV=1 |
| Glyceraldehyde-3-phosphate dehydrogenase (Fragment) OS=Homo sapiens GN=Gapdh PE=1 SV=1 | GTP-binding nuclear protein Ran, testis-specific isoform OS=Homo sapiens GN=Rasl2-9 PE=2 SV=1 |
| Glyceraldehyde-3-phosphate dehydrogenase (Fragment) OS=Homo sapiens PE=2 SV=1 | Heat shock 70 kDa protein 1B OS=Homo sapiens GN=Hspa1b PE=1 SV=3 |
| Glyceraldehyde-3-phosphate dehydrogenase OS=Homo sapiens GN=Gapdh PE=1 SV=1 | Heat shock 70 kDa protein 1-like OS=Homo sapiens GN=Hspa1l PE=1 SV=4 |
| Glyceraldehyde-3-phosphate dehydrogenase OS=Homo sapiens GN=Gapdh PE=1 SV=2 | Heat shock cognate 71 kDa protein (Fragment) OS=Homo sapiens GN=Hspa8 PE=1 SV=1 |
| Glyceraldehyde-3-phosphate dehydrogenase OS=Homo sapiens GN=Gapdhs PE=1 SV=1 | Heat shock cognate 71 kDa protein OS=Homo sapiens GN=Hspa8 PE=1 SV=1 |
| Glyceraldehyde-3-phosphate dehydrogenase OS=Homo sapiens GN=Gm10358 PE=1 SV=1 | Heat shock cognate hsc73 (Fragment) OS=Homo sapiens GN=Hspa8 PE=2 SV=1 |
| Glyceraldehyde-3-phosphate dehydrogenase OS=Homo sapiens GN=Gm3839 PE=1 SV=1 | Heat shock protein 1B OS=Homo sapiens GN=Hspa1b PE=1 SV=1 |
| Glyceraldehyde-3-phosphate dehydrogenase, testis-specific OS=Homo sapiens GN=Gapdhs PE=1 SV=1 | Heat shock protein 1-like protein OS=Homo sapiens PE=3 SV=1 |
| Glyceraldehyde-3-phosphate-dehydrogenase (Fragment) OS=Homo sapiens GN=Gapd PE=2 SV=1 | Heat shock protein 70 (Fragment) OS=Homo sapiens GN=Hsp70.3 PE=3 SV=1 |
| Glyco-gag polyprotein OS=Homo sapiens PE=4 SV=1 | Heat shock protein 70-2 OS=Homo sapiens PE=3 SV=1 |
| Golgi-associated plant pathogenesis-related protein 1 OS=Homo sapiens GN=Glipr2 PE=1 SV=1 | Heat shock-related 70 kDa protein 2 OS=Homo sapiens GN=Hspa2 PE=1 SV=2 |
| Golgi-associated plant pathogenesis-related protein 1 OS=Homo sapiens GN=Glipr2 PE=1 SV=3 | Heterogeneous nuclear ribonucleoprotein A/B OS=Homo sapiens GN=Hnrnpab PE=1 SV=1 |
| GTPase activating protein (SH3 domain) binding protein 2 OS=Homo sapiens GN=G3bp2 PE=2 SV=1 | Heterogeneous nuclear ribonucleoprotein A/B, isoform CRA_b OS=Homo sapiens GN=Hnrnpab PE=2 SV=1 |
| GTP-binding nuclear protein Ran OS=Homo sapiens GN=1700009N14Rik PE=2 SV=1 | Heterogeneous nuclear ribonucleoprotein A0 OS=Homo sapiens GN=Hnrnpa0 PE=1 SV=1 |
| GTP-binding nuclear protein Ran OS=Homo sapiens GN=Ran PE=1 SV=3 | Heterogeneous nuclear ribonucleoprotein A1 OS=Homo sapiens GN=Hnrnpa1 PE=1 SV=1 |
| GTP-binding nuclear protein Ran OS=Homo sapiens GN=Ran PE=2 SV=1 | Heterogeneous nuclear ribonucleoprotein A1 OS=Homo sapiens GN=Hnrnpa1 PE=1 SV=2 |
| GTP-binding nuclear protein Ran, testis-specific isoform OS=Homo sapiens GN=Rasl2-9 PE=2 SV=1 | Heterogeneous nuclear ribonucleoprotein A2/B1 OS=Homo sapiens GN=Hnrnpa2b1 PE=2 SV=1 |
| Heat shock 70 kDa protein 1B OS=Homo sapiens GN=Hspa1b PE=1 SV=3 | Heterogeneous nuclear ribonucleoprotein A3 (Fragment) OS=Homo sapiens GN=Hnrnpa3 PE=1 SV=1 |
| Heat shock 70 kDa protein 1-like OS=Homo sapiens GN=Hspa1l PE=1 SV=4 | Heterogeneous nuclear ribonucleoprotein A3 OS=Homo sapiens GN=Hnrnpa3 PE=1 SV=1 |
| Heat shock cognate 71 kDa protein (Fragment) OS=Homo sapiens GN=Hspa8 PE=1 SV=1 | Heterogeneous nuclear ribonucleoprotein A3 OS=Homo sapiens GN=Hnrnpa3 PE=2 SV=1 |
| Heat shock cognate 71 kDa protein OS=Homo sapiens GN=Hspa8 PE=1 SV=1 | Heterogeneous nuclear ribonucleoprotein D, isoform CRA_a OS=Homo sapiens GN=Hnrnpd PE=1 SV=1 |
| Heat shock cognate hsc73 (Fragment) OS=Homo sapiens GN=Hspa8 PE=2 SV=1 | Heterogeneous nuclear ribonucleoprotein D, isoform CRA_b OS=Homo sapiens GN=Hnrnpd PE=1 SV=1 |
| Heat shock protein 1B OS=Homo sapiens GN=Hspa1b PE=1 SV=1 | Heterogeneous nuclear ribonucleoprotein D0 (Fragment) OS=Homo sapiens GN=Hnrnpd PE=1 SV=1 |
| Heat shock protein 1-like protein OS=Homo sapiens PE=3 SV=1 | Heterogeneous nuclear ribonucleoprotein D0 OS=Homo sapiens GN=Hnrnpd PE=1 SV=2 |
| Heat shock protein 70 (Fragment) OS=Homo sapiens GN=Hsp70.3 PE=3 SV=1 | Heterogeneous nuclear ribonucleoprotein D-like (Fragment) OS=Homo sapiens GN=Hnrnpdl PE=1 SV=1 |
| Heat shock protein 70-2 OS=Homo sapiens PE=3 SV=1 | Heterogeneous nuclear ribonucleoprotein D-like OS=Homo sapiens GN=Hnrnpdl PE=1 SV=1 |
| Heat shock-related 70 kDa protein 2 OS=Homo sapiens GN=Hspa2 PE=1 SV=2 | Heterogeneous nuclear ribonucleoprotein F OS=Homo sapiens GN=Hnrnpf PE=1 SV=3 |
| Heterogeneous nuclear ribonucleoprotein A1 OS=Homo sapiens GN=Hnrnpa1 PE=1 SV=1 | Heterogeneous nuclear ribonucleoprotein H OS=Homo sapiens GN=Hnrnph1 PE=1 SV=1 |
| Heterogeneous nuclear ribonucleoprotein A1 OS=Homo sapiens GN=Hnrnpa1 PE=1 SV=2 | Heterogeneous nuclear ribonucleoprotein H1 OS=Homo sapiens GN=Hnrnph1 PE=1 SV=2 |
| Heterogeneous nuclear ribonucleoprotein A2/B1 OS=Homo sapiens GN=Hnrnpa2b1 PE=2 SV=1 | Heterogeneous nuclear ribonucleoprotein H2 OS=Homo sapiens GN=Hnrnph2 PE=1 SV=1 |
| Heterogeneous nuclear ribonucleoprotein F OS=Homo sapiens GN=Hnrnpf PE=1 SV=3 | Heterogeneous nuclear ribonucleoprotein K (Fragment) OS=Homo sapiens GN=Hnrnpk PE=1 SV=1 |
| Heterogeneous nuclear ribonucleoprotein H OS=Homo sapiens GN=Hnrnph1 PE=1 SV=1 | Heterogeneous nuclear ribonucleoprotein K (Fragment) OS=Homo sapiens GN=Hnrnpk PE=1 SV=8 |
| Heterogeneous nuclear ribonucleoprotein H1 OS=Homo sapiens GN=Hnrnph1 PE=1 SV=2 | Heterogeneous nuclear ribonucleoprotein K OS=Homo sapiens GN=Hnrnpk PE=1 SV=1 |
| Heterogeneous nuclear ribonucleoprotein H2 OS=Homo sapiens GN=Hnrnph2 PE=1 SV=1 | Heterogeneous nuclear ribonucleoprotein L (Fragment) OS=Homo sapiens GN=Hnrnpl PE=1 SV=1 |
| Heterogeneous nuclear ribonucleoprotein K (Fragment) OS=Homo sapiens GN=Hnrnpk PE=1 SV=1 | Heterogeneous nuclear ribonucleoprotein L OS=Homo sapiens GN=Hnrnpl PE=1 SV=1 |
| Heterogeneous nuclear ribonucleoprotein K (Fragment) OS=Homo sapiens GN=Hnrnpk PE=1 SV=8 | Heterogeneous nuclear ribonucleoprotein L OS=Homo sapiens GN=Hnrnpl PE=1 SV=2 |
| Heterogeneous nuclear ribonucleoprotein K OS=Homo sapiens GN=Hnrnpk PE=1 SV=1 | Heterogeneous nuclear ribonucleoprotein L-like OS=Homo sapiens GN=Hnrnpll PE=1 SV=1 |
| Heterogeneous nuclear ribonucleoprotein M OS=Homo sapiens GN=Hnrnpm PE=1 SV=1 | Heterogeneous nuclear ribonucleoprotein L-like OS=Homo sapiens GN=Hnrnpll PE=1 SV=3 |
| Heterogeneous nuclear ribonucleoprotein M OS=Homo sapiens GN=Hnrnpm PE=1 SV=3 | Heterogeneous nuclear ribonucleoprotein M OS=Homo sapiens GN=Hnrnpm PE=1 SV=1 |
| Heterogeneous nuclear ribonucleoprotein Q (Fragment) OS=Homo sapiens GN=Syncrip PE=1 SV=1 | Heterogeneous nuclear ribonucleoprotein M OS=Homo sapiens GN=Hnrnpm PE=1 SV=3 |
| Heterogeneous nuclear ribonucleoprotein Q OS=Homo sapiens GN=Syncrip PE=1 SV=1 | Heterogeneous nuclear ribonucleoprotein Q (Fragment) OS=Homo sapiens GN=Syncrip PE=1 SV=1 |
| Heterogeneous nuclear ribonucleoprotein Q OS=Homo sapiens GN=Syncrip PE=1 SV=2 | Heterogeneous nuclear ribonucleoprotein Q OS=Homo sapiens GN=Syncrip PE=1 SV=1 |
| Heterogeneous nuclear ribonucleoprotein U OS=Homo sapiens GN=Hnrnpu PE=1 SV=1 | Heterogeneous nuclear ribonucleoprotein Q OS=Homo sapiens GN=Syncrip PE=1 SV=2 |
| Heterogeneous nuclear ribonucleoproteins A2/B1 OS=Homo sapiens GN=Hnrnpa2b1 PE=1 SV=2 | Heterogeneous nuclear ribonucleoprotein R (Fragment) OS=Homo sapiens GN=Hnrnpr PE=1 SV=1 |
| Heterogenous nuclear ribonucleoprotein U OS=Homo sapiens GN=Hnrnpu PE=2 SV=1 | Heterogeneous nuclear ribonucleoprotein R OS=Homo sapiens GN=Hnrnpr PE=1 SV=1 |
| High mobility group protein B2 OS=Homo sapiens GN=Hmgb2 PE=1 SV=3 | Heterogeneous nuclear ribonucleoprotein U OS=Homo sapiens GN=Hnrnpu PE=1 SV=1 |
| High mobility group protein HMG-I/HMG-Y OS=Homo sapiens GN=Hmga1 PE=1 SV=4 | Heterogeneous nuclear ribonucleoproteins A2/B1 (Fragment) OS=Homo sapiens GN=Hnrnpa2b1 PE=1 SV=1 |
| Hist1h1b protein (Fragment) OS=Homo sapiens GN=Hist1h1b PE=2 SV=1 | Heterogeneous nuclear ribonucleoproteins A2/B1 OS=Homo sapiens GN=Hnrnpa2b1 PE=1 SV=2 |
| Hist2h2bb protein OS=Homo sapiens GN=Hist2h2bb PE=2 SV=1 | Heterogenous nuclear ribonucleoprotein U OS=Homo sapiens GN=Hnrnpu PE=2 SV=1 |
| Histone cluster 1, H1c OS=Homo sapiens GN=Hist1h1c PE=1 SV=1 | High mobility group box 1 OS=Homo sapiens GN=Hmgb1 PE=1 SV=1 |
| Histone cluster 1, H1d OS=Homo sapiens GN=Hist1h1d PE=1 SV=1 | High mobility group box 1 OS=Homo sapiens GN=Hmgb1 PE=2 SV=1 |
| Histone H1.1 OS=Homo sapiens GN=Hist1h1a PE=1 SV=2 | High mobility group protein B1 (Fragment) OS=Homo sapiens GN=Hmgb1 PE=1 SV=1 |
| Histone H1.4 OS=Homo sapiens GN=Hist1h1e PE=1 SV=2 | High mobility group protein B1 OS=Homo sapiens GN=Hmgb1 PE=1 SV=1 |
| Histone H1.5 OS=Homo sapiens GN=Hist1h1b PE=1 SV=2 | High mobility group protein B2 OS=Homo sapiens GN=Hmgb2 PE=1 SV=3 |
| Histone H1t OS=Homo sapiens GN=Hist1h1t PE=1 SV=1 | High mobility group protein HMGI-C OS=Homo sapiens GN=Hmga2 PE=1 SV=1 |
| Histone H1t OS=Homo sapiens GN=Hist1h1t PE=1 SV=4 | Hist1h1b protein (Fragment) OS=Homo sapiens GN=Hist1h1b PE=2 SV=1 |
| Histone H2A (Fragment) OS=Homo sapiens GN=Hist1h2ah PE=2 SV=1 | Hist2h2bb protein OS=Homo sapiens GN=Hist2h2bb PE=2 SV=1 |
| Histone H2A OS=Homo sapiens GN=H2afj PE=1 SV=1 | Histone cluster 1, H1c OS=Homo sapiens GN=Hist1h1c PE=1 SV=1 |
| Histone H2A OS=Homo sapiens GN=H2afv PE=2 SV=1 | Histone cluster 1, H1d OS=Homo sapiens GN=Hist1h1d PE=1 SV=1 |
| Histone H2A OS=Homo sapiens GN=H2afz PE=1 SV=1 | Histone H1.1 OS=Homo sapiens GN=Hist1h1a PE=1 SV=2 |
| Histone H2A OS=Homo sapiens GN=H2afz PE=2 SV=1 | Histone H1.4 OS=Homo sapiens GN=Hist1h1e PE=1 SV=2 |
| Histone H2A OS=Homo sapiens GN=Hist1h2aa PE=1 SV=1 | Histone H1.5 OS=Homo sapiens GN=Hist1h1b PE=1 SV=2 |
| Histone H2A OS=Homo sapiens GN=Hist1h2ah PE=2 SV=1 | Histone H1t OS=Homo sapiens GN=Hist1h1t PE=1 SV=1 |
| Histone H2A OS=Homo sapiens GN=Hist2h2aa1 PE=2 SV=1 | Histone H1t OS=Homo sapiens GN=Hist1h1t PE=1 SV=4 |
| Histone H2A type 1-F OS=Homo sapiens GN=Hist1h2af PE=1 SV=3 | Histone H2A (Fragment) OS=Homo sapiens GN=Hist1h2ah PE=2 SV=1 |
| Histone H2A type 1-I OS=Homo sapiens GN=Hist1h2ai PE=1 SV=1 | Histone H2A OS=Homo sapiens GN=H2afj PE=1 SV=1 |
| Histone H2A type 1-K OS=Homo sapiens GN=Hist1h2ak PE=1 SV=3 | Histone H2A OS=Homo sapiens GN=H2afv PE=2 SV=1 |
| Histone H2A type 2-B OS=Homo sapiens GN=Hist2h2ab PE=1 SV=3 | Histone H2A OS=Homo sapiens GN=H2afz PE=1 SV=1 |
| Histone H2A type 2-C OS=Homo sapiens GN=Hist2h2ac PE=1 SV=3 | Histone H2A OS=Homo sapiens GN=H2afz PE=2 SV=1 |
| Histone H2A type 3 OS=Homo sapiens GN=Hist3h2a PE=1 SV=3 | Histone H2A OS=Homo sapiens GN=Hist1h2aa PE=1 SV=1 |
| Histone H2A.J OS=Homo sapiens GN=H2afj PE=1 SV=1 | Histone H2A OS=Homo sapiens GN=Hist1h2ah PE=2 SV=1 |
| Histone H2A.Z OS=Homo sapiens GN=H2afz PE=1 SV=2 | Histone H2A OS=Homo sapiens GN=Hist2h2aa1 PE=2 SV=1 |
| Histone H2AX OS=Homo sapiens GN=H2afx PE=1 SV=2 | Histone H2A type 1-F OS=Homo sapiens GN=Hist1h2af PE=1 SV=3 |
| Histone H2B (Fragment) OS=Homo sapiens GN=Hist1h2bj PE=2 SV=1 | Histone H2A type 1-I OS=Homo sapiens GN=Hist1h2ai PE=1 SV=1 |
| Histone H2B OS=Homo sapiens GN=Hist1h2ba PE=2 SV=1 | Histone H2A type 1-K OS=Homo sapiens GN=Hist1h2ak PE=1 SV=3 |
| Histone H2B OS=Homo sapiens GN=Hist1h2bk PE=2 SV=1 | Histone H2A type 2-B OS=Homo sapiens GN=Hist2h2ab PE=1 SV=3 |
| Histone H2B OS=Homo sapiens GN=Hist1h2bm PE=1 SV=1 | Histone H2A type 2-C OS=Homo sapiens GN=Hist2h2ac PE=1 SV=3 |
| Histone H2B OS=Homo sapiens GN=Hist1h2bq PE=2 SV=1 | Histone H2A type 3 OS=Homo sapiens GN=Hist3h2a PE=1 SV=3 |
| Histone H2B OS=Homo sapiens GN=LOC665622 PE=2 SV=1 | Histone H2A.J OS=Homo sapiens GN=H2afj PE=1 SV=1 |
| Histone H2B type 1-A OS=Homo sapiens GN=Hist1h2ba PE=1 SV=3 | Histone H2A.Z OS=Homo sapiens GN=H2afz PE=1 SV=2 |
| Histone H2B type 1-B OS=Homo sapiens GN=Hist1h2bb PE=1 SV=3 | Histone H2AX OS=Homo sapiens GN=H2afx PE=1 SV=2 |
| Histone H2B type 1-C/E/G OS=Homo sapiens GN=Hist1h2bc PE=1 SV=3 | Histone H2B (Fragment) OS=Homo sapiens GN=Hist1h2bj PE=2 SV=1 |
| Histone H2B type 1-F/J/L OS=Homo sapiens GN=Hist1h2bf PE=1 SV=2 | Histone H2B OS=Homo sapiens GN=Hist1h2ba PE=2 SV=1 |
| Histone H2B type 1-H OS=Homo sapiens GN=Hist1h2bh PE=1 SV=3 | Histone H2B OS=Homo sapiens GN=Hist1h2bk PE=2 SV=1 |
| Histone H2B type 1-P OS=Homo sapiens GN=Hist1h2bp PE=1 SV=3 | Histone H2B OS=Homo sapiens GN=Hist1h2bm PE=1 SV=1 |
| Histone H2B type 2-B OS=Homo sapiens GN=Hist2h2bb PE=1 SV=3 | Histone H2B OS=Homo sapiens GN=Hist1h2bq PE=2 SV=1 |
| Histone H2B type 2-E OS=Homo sapiens GN=Hist2h2be PE=1 SV=3 | Histone H2B OS=Homo sapiens GN=LOC665622 PE=2 SV=1 |
| Histone H2B type 3-A OS=Homo sapiens GN=Hist3h2ba PE=1 SV=3 | Histone H2B type 1-A OS=Homo sapiens GN=Hist1h2ba PE=1 SV=3 |
| Histone H2B type 3-B OS=Homo sapiens GN=Hist3h2bb PE=1 SV=3 | Histone H2B type 1-B OS=Homo sapiens GN=Hist1h2bb PE=1 SV=3 |
| Histone H3 (Fragment) OS=Homo sapiens GN=H3f3a PE=3 SV=1 | Histone H2B type 1-C/E/G OS=Homo sapiens GN=Hist1h2bc PE=1 SV=3 |
| Histone H3 (Fragment) OS=Homo sapiens GN=Hist1h3e PE=2 SV=1 | Histone H2B type 1-F/J/L OS=Homo sapiens GN=Hist1h2bf PE=1 SV=2 |
| Histone H3 (Fragment) OS=Homo sapiens GN=Hist1h3i PE=2 SV=1 | Histone H2B type 1-H OS=Homo sapiens GN=Hist1h2bh PE=1 SV=3 |
| Histone H3 OS=Homo sapiens GN=H3f3a PE=1 SV=1 | Histone H2B type 1-P OS=Homo sapiens GN=Hist1h2bp PE=1 SV=3 |
| Histone H3.1 OS=Homo sapiens GN=Hist1h3a PE=1 SV=2 | Histone H2B type 2-B OS=Homo sapiens GN=Hist2h2bb PE=1 SV=3 |
| Histone H3.2 OS=Homo sapiens GN=Hist1h3b PE=1 SV=2 | Histone H2B type 2-E OS=Homo sapiens GN=Hist2h2be PE=1 SV=3 |
| Histone H3.2 OS=Homo sapiens GN=Hist2h3c1 PE=1 SV=1 | Histone H2B type 3-A OS=Homo sapiens GN=Hist3h2ba PE=1 SV=3 |
| Histone H3.3 OS=Homo sapiens GN=H3f3a PE=1 SV=2 | Histone H2B type 3-B OS=Homo sapiens GN=Hist3h2bb PE=1 SV=3 |
| Histone H3.3C OS=Homo sapiens GN=H3f3c PE=3 SV=3 | Histone H3 (Fragment) OS=Homo sapiens GN=H3f3a PE=3 SV=1 |
| Histone H4 (Fragment) OS=Homo sapiens PE=3 SV=1 | Histone H3 (Fragment) OS=Homo sapiens GN=Hist1h3e PE=2 SV=1 |
| Histone H4 OS=Homo sapiens GN=Hist2h4 PE=1 SV=1 | Histone H3 (Fragment) OS=Homo sapiens GN=Hist1h3i PE=2 SV=1 |
| Hnrpf protein (Fragment) OS=Homo sapiens GN=Hnrnpf PE=2 SV=1 | Histone H3 OS=Homo sapiens GN=H3f3a PE=1 SV=1 |
| Hnrpk protein OS=Homo sapiens GN=Hnrnpk PE=2 SV=1 | Histone H3.1 OS=Homo sapiens GN=Hist1h3a PE=1 SV=2 |
| Hsc70t (Fragment) OS=Homo sapiens PE=3 SV=1 | Histone H3.2 OS=Homo sapiens GN=Hist1h3b PE=1 SV=2 |
| Hspa8 protein (Fragment) OS=Homo sapiens GN=Hspa8 PE=2 SV=1 | Histone H3.2 OS=Homo sapiens GN=Hist2h3c1 PE=1 SV=1 |
| IFN-response element binding factor 2 (Fragment) OS=Homo sapiens GN=Srsf2 PE=2 SV=1 | Histone H3.3 OS=Homo sapiens GN=H3f3a PE=1 SV=2 |
| Importin subunit alpha OS=Homo sapiens GN=Kpna2 PE=1 SV=1 | Histone H3.3C OS=Homo sapiens GN=H3f3c PE=3 SV=3 |
| Importin subunit alpha OS=Homo sapiens GN=Kpna2 PE=2 SV=1 | Histone H4 (Fragment) OS=Homo sapiens PE=3 SV=1 |
| Inducible heat shock protein 70 OS=Homo sapiens PE=3 SV=1 | Histone H4 OS=Homo sapiens GN=Hist2h4 PE=1 SV=1 |
| IQ motif containing GTPase activating protein 1 OS=Homo sapiens GN=Iqgap1 PE=2 SV=1 | Hmgb1 protein OS=Homo sapiens PE=2 SV=1 |
| Iqgap1 protein (Fragment) OS=Homo sapiens GN=Iqgap1 PE=2 SV=1 | Hnrnpa1l2 protein OS=Homo sapiens GN=Hnrnpa1l2 PE=2 SV=1 |
| Keratin 15, isoform CRA_a OS=Homo sapiens GN=Krt15 PE=1 SV=1 | Hnrpa3 protein (Fragment) OS=Homo sapiens GN=Hnrnpa3 PE=2 SV=1 |
| Keratin 16 OS=Homo sapiens GN=Krt16 PE=1 SV=1 | Hnrpa3 protein OS=Homo sapiens GN=Hnrnpa3 PE=2 SV=1 |
| Keratin 16 OS=Homo sapiens GN=Krt16 PE=2 SV=1 | Hnrpf protein (Fragment) OS=Homo sapiens GN=Hnrnpf PE=2 SV=1 |
| Keratin 1b (Fragment) OS=Homo sapiens GN=Krt77 PE=2 SV=1 | Hnrpk protein OS=Homo sapiens GN=Hnrnpk PE=2 SV=1 |
| Keratin 24 variant 2 OS=Homo sapiens GN=Krt24 PE=2 SV=1 | Hnrpr protein (Fragment) OS=Homo sapiens GN=Hnrnpr PE=2 SV=1 |
| Keratin 5 OS=Homo sapiens GN=Krt5 PE=1 SV=2 | Hsc70t (Fragment) OS=Homo sapiens PE=3 SV=1 |
| Keratin 77 OS=Homo sapiens GN=Krt77 PE=2 SV=1 | Hspa8 protein (Fragment) OS=Homo sapiens GN=Hspa8 PE=2 SV=1 |
| Keratin 78 OS=Homo sapiens GN=Krt78 PE=1 SV=1 | IFN-response element binding factor 2 (Fragment) OS=Homo sapiens GN=Srsf2 PE=2 SV=1 |
| Keratin intermediate filament 16a OS=Homo sapiens GN=K16 PE=3 SV=1 | Importin subunit alpha OS=Homo sapiens GN=Kpna2 PE=1 SV=1 |
| Keratin intermediate filament 16b OS=Homo sapiens GN=K16 PE=3 SV=1 | Importin subunit alpha OS=Homo sapiens GN=Kpna2 PE=2 SV=1 |
| Keratin Kb40 OS=Homo sapiens GN=Krt78 PE=2 SV=1 | Inducible heat shock protein 70 OS=Homo sapiens PE=3 SV=1 |
| Keratin, type I cuticular Ha1 OS=Homo sapiens GN=Krt31 PE=1 SV=2 | Insulin-like growth factor 2 mRNA-binding protein 2 OS=Homo sapiens GN=Igf2bp2 PE=1 SV=1 |
| Keratin, type I cuticular Ha2 OS=Homo sapiens GN=Krt32 PE=1 SV=1 | Insulin-like growth factor 2 mRNA-binding protein 3 OS=Homo sapiens GN=Igf2bp3 PE=1 SV=1 |
| Keratin, type I cuticular Ha2 OS=Homo sapiens GN=Krt32 PE=1 SV=2 | Integrin beta OS=Homo sapiens GN=Itgb4 PE=1 SV=1 |
| Keratin, type I cuticular Ha3-II OS=Homo sapiens GN=Krt33b PE=1 SV=2 | Integrin beta-4 OS=Homo sapiens GN=Itgb4 PE=1 SV=1 |
| Keratin, type I cuticular Ha5 OS=Homo sapiens GN=Krt35 PE=1 SV=1 | Interferon-activable protein 204 OS=Homo sapiens GN=Ifi204 PE=1 SV=1 |
| Keratin, type I cuticular Ha6 OS=Homo sapiens GN=Krt36 PE=1 SV=1 | IQ and AAA domain-containing protein 1-like OS=Homo sapiens GN=Iqca1l PE=1 SV=3 |
| Keratin, type I cytoskeletal 10 OS=Homo sapiens GN=Krt10 PE=1 SV=1 | IQ motif containing GTPase activating protein 1 OS=Homo sapiens GN=Iqgap1 PE=2 SV=1 |
| Keratin, type I cytoskeletal 10 OS=Homo sapiens GN=Krt10 PE=1 SV=3 | Iqgap1 protein (Fragment) OS=Homo sapiens GN=Iqgap1 PE=2 SV=1 |
| Keratin, type I cytoskeletal 13 OS=Homo sapiens GN=Krt13 PE=1 SV=2 | Janus kinase and microtubule-interacting protein 1 OS=Homo sapiens GN=Jakmip1 PE=1 SV=1 |
| Keratin, type I cytoskeletal 14 OS=Homo sapiens GN=Krt14 PE=1 SV=2 | Keratin 15, isoform CRA_a OS=Homo sapiens GN=Krt15 PE=1 SV=1 |
| Keratin, type I cytoskeletal 15 OS=Homo sapiens GN=Krt15 PE=1 SV=2 | Keratin 1b (Fragment) OS=Homo sapiens GN=Krt77 PE=2 SV=1 |
| Keratin, type I cytoskeletal 17 OS=Homo sapiens GN=Krt17 PE=1 SV=3 | Keratin 5 OS=Homo sapiens GN=Krt5 PE=1 SV=2 |
| Keratin, type I cytoskeletal 18 OS=Homo sapiens GN=Krt18 PE=1 SV=5 | Keratin 77 OS=Homo sapiens GN=Krt77 PE=2 SV=1 |
| Keratin, type I cytoskeletal 19 OS=Homo sapiens GN=Krt19 PE=1 SV=1 | Keratin 78 OS=Homo sapiens GN=Krt78 PE=1 SV=1 |
| Keratin, type I cytoskeletal 24 OS=Homo sapiens GN=Krt24 PE=2 SV=2 | Keratin Kb40 OS=Homo sapiens GN=Krt78 PE=2 SV=1 |
| Keratin, type I cytoskeletal 28 OS=Homo sapiens GN=Krt28 PE=1 SV=1 | Keratin, type I cytoskeletal 10 OS=Homo sapiens GN=Krt10 PE=1 SV=1 |
| Keratin, type I cytoskeletal 40 OS=Homo sapiens GN=Krt40 PE=2 SV=1 | Keratin, type I cytoskeletal 10 OS=Homo sapiens GN=Krt10 PE=1 SV=3 |
| Keratin, type I cytoskeletal 42 OS=Homo sapiens GN=Krt42 PE=1 SV=1 | Keratin, type I cytoskeletal 14 OS=Homo sapiens GN=Krt14 PE=1 SV=2 |
| Keratin, type II cytoskeletal 1 OS=Homo sapiens GN=Krt1 PE=1 SV=4 | Keratin, type I cytoskeletal 15 OS=Homo sapiens GN=Krt15 PE=1 SV=2 |
| Keratin, type II cytoskeletal 1b OS=Homo sapiens GN=Krt77 PE=1 SV=1 | Keratin, type I cytoskeletal 17 OS=Homo sapiens GN=Krt17 PE=1 SV=3 |
| Keratin, type II cytoskeletal 2 oral OS=Homo sapiens GN=Krt76 PE=1 SV=1 | Keratin, type I cytoskeletal 19 OS=Homo sapiens GN=Krt19 PE=1 SV=1 |
| Keratin, type II cytoskeletal 6A OS=Homo sapiens GN=Krt6a PE=1 SV=3 | Keratin, type I cytoskeletal 42 OS=Homo sapiens GN=Krt42 PE=1 SV=1 |
| Keratin, type II cytoskeletal 6B OS=Homo sapiens GN=Krt6b PE=1 SV=1 | Keratin, type II cytoskeletal 1 OS=Homo sapiens GN=Krt1 PE=1 SV=4 |
| Keratin, type II cytoskeletal 6B OS=Homo sapiens GN=Krt6b PE=1 SV=3 | Keratin, type II cytoskeletal 1b OS=Homo sapiens GN=Krt77 PE=1 SV=1 |
| Keratin, type II cytoskeletal 7 OS=Homo sapiens GN=Krt7 PE=1 SV=1 | Keratin, type II cytoskeletal 2 epidermal OS=Homo sapiens GN=Krt2 PE=1 SV=1 |
| Keratin, type II cytoskeletal 75 OS=Homo sapiens GN=Krt75 PE=1 SV=1 | Keratin, type II cytoskeletal 6A OS=Homo sapiens GN=Krt6a PE=1 SV=3 |
| Keratin, type II cytoskeletal 79 OS=Homo sapiens GN=Krt79 PE=1 SV=2 | Keratin, type II cytoskeletal 6B OS=Homo sapiens GN=Krt6b PE=1 SV=1 |
| Keratin, type II cytoskeletal 8 OS=Homo sapiens GN=Krt8 PE=1 SV=4 | Keratin, type II cytoskeletal 6B OS=Homo sapiens GN=Krt6b PE=1 SV=3 |
| Krt6b protein (Fragment) OS=Homo sapiens GN=Krt6b PE=2 SV=1 | Keratin, type II cytoskeletal 7 OS=Homo sapiens GN=Krt7 PE=1 SV=1 |
| Krt6b protein OS=Homo sapiens GN=Krt6b PE=2 SV=1 | Keratin, type II cytoskeletal 71 OS=Homo sapiens GN=Krt71 PE=1 SV=1 |
| Krt78 protein (Fragment) OS=Homo sapiens GN=Krt78 PE=2 SV=1 | Keratin, type II cytoskeletal 73 OS=Homo sapiens GN=Krt73 PE=1 SV=1 |
| Lamina-associated polypeptide 2, isoforms alpha/zeta OS=Homo sapiens GN=Tmpo PE=1 SV=4 | Keratin, type II cytoskeletal 74 OS=Homo sapiens GN=Krt74 PE=3 SV=1 |
| Lamina-associated polypeptide 2, isoforms beta/delta/epsilon/gamma OS=Homo sapiens GN=Tmpo PE=1 SV=4 | Keratin, type II cytoskeletal 75 OS=Homo sapiens GN=Krt75 PE=1 SV=1 |
| Lamin-B receptor (Fragment) OS=Homo sapiens GN=Lbr PE=1 SV=1 | Keratin, type II cytoskeletal 79 OS=Homo sapiens GN=Krt79 PE=1 SV=2 |
| Lamin-B receptor OS=Homo sapiens GN=Lbr PE=1 SV=2 | Keratin, type II cytoskeletal 8 OS=Homo sapiens GN=Krt8 PE=1 SV=4 |
| Laminin receptor (Fragment) OS=Homo sapiens GN=Rpsa PE=2 SV=1 | Krt2 protein OS=Homo sapiens GN=Krt2 PE=2 SV=1 |
| La-related protein 4 OS=Homo sapiens GN=Larp4 PE=1 SV=1 | Krt6b protein (Fragment) OS=Homo sapiens GN=Krt6b PE=2 SV=1 |
| La-related protein 4 OS=Homo sapiens GN=Larp4 PE=1 SV=2 | Krt6b protein OS=Homo sapiens GN=Krt6b PE=2 SV=1 |
| Leucine-rich repeat-containing protein 59 OS=Homo sapiens GN=Lrrc59 PE=1 SV=1 | Krt78 protein (Fragment) OS=Homo sapiens GN=Krt78 PE=2 SV=1 |
| Lingerer protein-2a OS=Homo sapiens GN=lig-2a PE=2 SV=1 | Lamina-associated polypeptide 2, isoforms alpha/zeta OS=Homo sapiens GN=Tmpo PE=1 SV=4 |
| Lingerer protein-2b OS=Homo sapiens GN=Ubap2l PE=2 SV=1 | Lamina-associated polypeptide 2, isoforms beta/delta/epsilon/gamma OS=Homo sapiens GN=Tmpo PE=1 SV=4 |
| Luc7-like protein 3 OS=Homo sapiens GN=Luc7l3 PE=1 SV=1 | Laminin receptor (Fragment) OS=Homo sapiens GN=Rpsa PE=2 SV=1 |
| Mannosyl-oligosaccharide glucosidase OS=Homo sapiens GN=Mogs PE=1 SV=1 | Leucine-rich repeat-containing protein 59 OS=Homo sapiens GN=Lrrc59 PE=1 SV=1 |
| MCG10168 OS=Homo sapiens GN=Rplp1 PE=1 SV=1 | Lingerer protein-2a OS=Homo sapiens GN=lig-2a PE=2 SV=1 |
| MCG10266, isoform CRA_a OS=Homo sapiens GN=Rpl9 PE=1 SV=1 | Lingerer protein-2b OS=Homo sapiens GN=Ubap2l PE=2 SV=1 |
| MCG10725, isoform CRA_a OS=Homo sapiens GN=Rps25 PE=1 SV=1 | Luc7 homolog (S. cerevisiae)-like, isoform CRA_c OS=Homo sapiens GN=Luc7l PE=1 SV=1 |
| MCG10806 OS=Homo sapiens GN=Rpl23a PE=1 SV=1 | Luc7l protein (Fragment) OS=Homo sapiens GN=Luc7l PE=2 SV=1 |
| MCG116671 OS=Homo sapiens GN=Gm11361 PE=3 SV=1 | Luc7l2 protein OS=Homo sapiens GN=Luc7l2 PE=1 SV=1 |
| MCG118780 OS=Homo sapiens GN=Gm5788 PE=3 SV=1 | Luc7-like protein 3 OS=Homo sapiens GN=Luc7l3 PE=1 SV=1 |
| MCG12304 OS=Homo sapiens GN=Rpl22 PE=1 SV=1 | Lupus La protein homolog (Fragment) OS=Homo sapiens GN=Ssb PE=1 SV=1 |
| MCG123152 OS=Homo sapiens GN=Gm10269 PE=1 SV=1 | Lupus La protein homolog OS=Homo sapiens GN=Ssb PE=1 SV=1 |
| MCG124046 OS=Homo sapiens GN=Prss1 PE=1 SV=1 | Mannosyl-oligosaccharide glucosidase OS=Homo sapiens GN=Mogs PE=1 SV=1 |
| MCG126194, isoform CRA_a OS=Homo sapiens GN=Rpl31 PE=1 SV=1 | MCG10168 OS=Homo sapiens GN=Rplp1 PE=1 SV=1 |
| MCG132477, isoform CRA_a OS=Homo sapiens GN=Rpl18 PE=1 SV=1 | MCG10266, isoform CRA_a OS=Homo sapiens GN=Rpl9 PE=1 SV=1 |
| MCG13441 OS=Homo sapiens GN=Rps27a PE=2 SV=1 | MCG10725, isoform CRA_a OS=Homo sapiens GN=Rps25 PE=1 SV=1 |
| MCG140437, isoform CRA_d OS=Homo sapiens GN=Myh2 PE=1 SV=1 | MCG10806 OS=Homo sapiens GN=Rpl23a PE=1 SV=1 |
| MCG140959, isoform CRA_a OS=Homo sapiens GN=Myl6 PE=2 SV=1 | MCG116386, isoform CRA_a OS=Homo sapiens GN=Rbm3 PE=1 SV=1 |
| MCG14259, isoform CRA_a OS=Homo sapiens GN=U2af1 PE=1 SV=1 | MCG116671 OS=Homo sapiens GN=Gm11361 PE=3 SV=1 |
| MCG147570 (Fragment) OS=Homo sapiens GN=E130201H02Rik PE=2 SV=1 | MCG12304 OS=Homo sapiens GN=Rpl22 PE=1 SV=1 |
| MCG15083 OS=Homo sapiens GN=Try5 PE=1 SV=1 | MCG123152 OS=Homo sapiens GN=Gm10269 PE=1 SV=1 |
| MCG15085 OS=Homo sapiens GN=Try4 PE=1 SV=1 | MCG124046 OS=Homo sapiens GN=Prss1 PE=1 SV=1 |
| MCG17585 OS=Homo sapiens GN=Rpl39 PE=2 SV=1 | MCG126194, isoform CRA_a OS=Homo sapiens GN=Rpl31 PE=1 SV=1 |
| MCG17902, isoform CRA_a OS=Homo sapiens GN=Srsf7 PE=2 SV=1 | MCG132477, isoform CRA_a OS=Homo sapiens GN=Rpl18 PE=1 SV=1 |
| MCG18564, isoform CRA_a OS=Homo sapiens GN=Rpl12 PE=1 SV=1 | MCG13402, isoform CRA_a OS=Homo sapiens GN=Ptbp1 PE=1 SV=1 |
| MCG20799 OS=Homo sapiens GN=Rpl30 PE=1 SV=1 | MCG13402, isoform CRA_c OS=Homo sapiens GN=Ptbp1 PE=1 SV=1 |
| MCG21131, isoform CRA_a OS=Homo sapiens GN=Srsf3 PE=2 SV=1 | MCG13402, isoform CRA_d OS=Homo sapiens GN=Ptbp1 PE=1 SV=1 |
| MCG21688 OS=Homo sapiens GN=Rps10 PE=1 SV=1 | MCG140437, isoform CRA_d OS=Homo sapiens GN=Myh2 PE=1 SV=1 |
| MCG21910 OS=Homo sapiens GN=Gm12355 PE=4 SV=1 | MCG140959, isoform CRA_a OS=Homo sapiens GN=Myl6 PE=2 SV=1 |
| MCG23000, isoform CRA_b OS=Homo sapiens GN=Rps18 PE=2 SV=1 | MCG14259, isoform CRA_a OS=Homo sapiens GN=U2af1 PE=1 SV=1 |
| MCG2872, isoform CRA_b OS=Homo sapiens GN=Ddx5 PE=1 SV=1 | MCG14259, isoform CRA_b OS=Homo sapiens GN=U2af1 PE=1 SV=1 |
| MCG3574 OS=Homo sapiens GN=Rps20 PE=1 SV=1 | MCG147570 (Fragment) OS=Homo sapiens GN=E130201H02Rik PE=2 SV=1 |
| MCG4647 OS=Homo sapiens GN=Hmgb2 PE=2 SV=1 | MCG15083 OS=Homo sapiens GN=Try5 PE=1 SV=1 |
| MCG4862 OS=Homo sapiens GN=Snrpd2 PE=1 SV=1 | MCG15085 OS=Homo sapiens GN=Try4 PE=1 SV=1 |
| MCG50795 OS=Homo sapiens GN=Rpl9-ps1 PE=4 SV=1 | MCG17585 OS=Homo sapiens GN=Rpl39 PE=2 SV=1 |
| MCG5400 OS=Homo sapiens GN=Myl12a PE=1 SV=1 | MCG17902, isoform CRA_a OS=Homo sapiens GN=Srsf7 PE=2 SV=1 |
| MCG7614, isoform CRA_c OS=Homo sapiens GN=Srsf5 PE=1 SV=1 | MCG18564, isoform CRA_a OS=Homo sapiens GN=Rpl12 PE=1 SV=1 |
| Microtubule-associated protein 4 OS=Homo sapiens GN=Map4 PE=1 SV=3 | MCG20799 OS=Homo sapiens GN=Rpl30 PE=1 SV=1 |
| MKIAA0051 protein (Fragment) OS=Homo sapiens GN=Iqgap1 PE=2 SV=1 | MCG21131, isoform CRA_a OS=Homo sapiens GN=Srsf3 PE=2 SV=1 |
| MKIAA0144 protein (Fragment) OS=Homo sapiens GN=Ubap2l PE=2 SV=1 | MCG21688 OS=Homo sapiens GN=Rps10 PE=1 SV=1 |
| MKIAA0400 protein (Fragment) OS=Homo sapiens GN=Asap2 PE=2 SV=1 | MCG21910 OS=Homo sapiens GN=Gm12355 PE=4 SV=1 |
| MKIAA0741 protein (Fragment) OS=Homo sapiens GN=Eif5b PE=2 SV=1 | MCG23000, isoform CRA_b OS=Homo sapiens GN=Rps18 PE=2 SV=1 |
| MKIAA0866 protein (Fragment) OS=Homo sapiens GN=Myh11 PE=2 SV=1 | MCG2872, isoform CRA_b OS=Homo sapiens GN=Ddx5 PE=1 SV=1 |
| MKIAA0936 protein (Fragment) OS=Homo sapiens GN=Ick PE=2 SV=1 | MCG3574 OS=Homo sapiens GN=Rps20 PE=1 SV=1 |
| MKIAA3005 protein (Fragment) OS=Homo sapiens GN=Myh10 PE=2 SV=1 | MCG4647 OS=Homo sapiens GN=Hmgb2 PE=2 SV=1 |
| MKIAA4075 protein (Fragment) OS=Homo sapiens GN=Chd4 PE=2 SV=1 | MCG4862 OS=Homo sapiens GN=Snrpd2 PE=1 SV=1 |
| MKIAA4115 protein (Fragment) OS=Homo sapiens GN=G3bp1 PE=2 SV=1 | MCG53108 OS=Homo sapiens GN=Rbm31y PE=2 SV=1 |
| MKIAA4193 protein (Fragment) OS=Homo sapiens GN=Hnrnpm PE=2 SV=1 | MCG5400 OS=Homo sapiens GN=Myl12a PE=1 SV=1 |
| Moesin OS=Homo sapiens GN=Msn PE=1 SV=3 | MCG7614, isoform CRA_c OS=Homo sapiens GN=Srsf5 PE=1 SV=1 |
| Msn protein (Fragment) OS=Homo sapiens GN=Msn PE=2 SV=1 | MCG8382, isoform CRA_c OS=Homo sapiens GN=Rbm14 PE=1 SV=2 |
| MYB binding protein (P160) 1a, isoform CRA_b OS=Homo sapiens GN=Mybbp1a PE=1 SV=1 | Microtubule-associated protein (Fragment) OS=Homo sapiens GN=Map4 PE=1 SV=1 |
| MYB-1a OS=Homo sapiens GN=Ybx1 PE=2 SV=1 | Microtubule-associated protein 1A OS=Homo sapiens GN=Map1a PE=1 SV=1 |
| MYB-1b OS=Homo sapiens GN=Ybx1 PE=2 SV=1 | Microtubule-associated protein 1A OS=Homo sapiens GN=Map1a PE=1 SV=2 |
| Mybbp1a protein (Fragment) OS=Homo sapiens GN=Mybbp1a PE=2 SV=1 | Microtubule-associated protein 1B OS=Homo sapiens GN=Map1b PE=1 SV=2 |
| Myh10 protein (Fragment) OS=Homo sapiens GN=Myh10 PE=2 SV=1 | Microtubule-associated protein 1B OS=Homo sapiens GN=Map1b PE=2 SV=1 |
| Myh14 protein (Fragment) OS=Homo sapiens GN=Myh14 PE=2 SV=1 | Microtubule-associated protein 4 OS=Homo sapiens GN=Map4 PE=1 SV=3 |
| Myh2 protein OS=Homo sapiens GN=Myh2 PE=2 SV=1 | Microtubule-associated protein OS=Homo sapiens GN=Map4 PE=1 SV=1 |
| Myh9 protein (Fragment) OS=Homo sapiens GN=Myh9 PE=2 SV=1 | Microtubule-associated protein OS=Homo sapiens GN=Map4 PE=1 SV=3 |
| Myh9 protein OS=Homo sapiens GN=Myh9 PE=2 SV=1 | MKIAA0051 protein (Fragment) OS=Homo sapiens GN=Iqgap1 PE=2 SV=1 |
| Myosin heavy chain IIB (Fragment) OS=Homo sapiens GN=Myh4 PE=2 SV=1 | MKIAA0144 protein (Fragment) OS=Homo sapiens GN=Ubap2l PE=2 SV=1 |
| Myosin heavy chain IIX (Fragment) OS=Homo sapiens GN=Myh1 PE=2 SV=1 | MKIAA0670 protein (Fragment) OS=Homo sapiens GN=Acin1 PE=2 SV=1 |
| Myosin light chain 6B OS=Homo sapiens GN=Myl6b PE=1 SV=1 | MKIAA0741 protein (Fragment) OS=Homo sapiens GN=Eif5b PE=2 SV=1 |
| Myosin light polypeptide 6 alkali smooth muscle and non-muscle protein (Fragment) OS=Homo sapiens GN=Myl6 PE=2 SV=1 | MKIAA0845 protein (Fragment) OS=Homo sapiens GN=Nefh PE=2 SV=1 |
| Myosin light polypeptide 6 OS=Homo sapiens GN=Myl6 PE=1 SV=1 | MKIAA0866 protein (Fragment) OS=Homo sapiens GN=Myh11 PE=2 SV=1 |
| Myosin light polypeptide 6 OS=Homo sapiens GN=Myl6 PE=1 SV=3 | MKIAA0936 protein (Fragment) OS=Homo sapiens GN=Ick PE=2 SV=1 |
| Myosin regulatory light chain 12B OS=Homo sapiens GN=Myl12b PE=1 SV=2 | MKIAA1172 protein (Fragment) OS=Homo sapiens GN=Scaf4 PE=2 SV=1 |
| Myosin, heavy polypeptide 1, skeletal muscle, adult OS=Homo sapiens GN=Myh1 PE=2 SV=1 | MKIAA3005 protein (Fragment) OS=Homo sapiens GN=Myh10 PE=2 SV=1 |
| Myosin, heavy polypeptide 8, skeletal muscle, perinatal OS=Homo sapiens GN=Myh8 PE=2 SV=1 | MKIAA4115 protein (Fragment) OS=Homo sapiens GN=G3bp1 PE=2 SV=1 |
| Myosin-1 OS=Homo sapiens GN=Myh1 PE=1 SV=1 | MKIAA4193 protein (Fragment) OS=Homo sapiens GN=Hnrnpm PE=2 SV=1 |
| Myosin-10 OS=Homo sapiens GN=Myh10 PE=1 SV=1 | MYB binding protein (P160) 1a, isoform CRA_b OS=Homo sapiens GN=Mybbp1a PE=1 SV=1 |
| Myosin-10 OS=Homo sapiens GN=Myh10 PE=1 SV=2 | MYB-1a OS=Homo sapiens GN=Ybx1 PE=2 SV=1 |
| Myosin-11 OS=Homo sapiens GN=Myh11 PE=1 SV=1 | MYB-1b OS=Homo sapiens GN=Ybx1 PE=2 SV=1 |
| Myosin-14 (Fragment) OS=Homo sapiens GN=Myh14 PE=1 SV=1 | Mybbp1a protein (Fragment) OS=Homo sapiens GN=Mybbp1a PE=2 SV=1 |
| Myosin-14 OS=Homo sapiens GN=Myh14 PE=1 SV=1 | Myh10 protein (Fragment) OS=Homo sapiens GN=Myh10 PE=2 SV=1 |
| Myosin-4 OS=Homo sapiens GN=Myh4 PE=2 SV=1 | Myh14 protein (Fragment) OS=Homo sapiens GN=Myh14 PE=2 SV=1 |
| Myosin-8 OS=Homo sapiens GN=Myh8 PE=2 SV=2 | Myh2 protein OS=Homo sapiens GN=Myh2 PE=2 SV=1 |
| Myosin-9 OS=Homo sapiens GN=Myh9 PE=1 SV=1 | Myh9 protein (Fragment) OS=Homo sapiens GN=Myh9 PE=2 SV=1 |
| Myosin-9 OS=Homo sapiens GN=Myh9 PE=1 SV=4 | Myh9 protein OS=Homo sapiens GN=Myh9 PE=2 SV=1 |
| Nascent polypeptide-associated complex subunit alpha OS=Homo sapiens GN=Naca PE=1 SV=1 | Myosin heavy chain IIB (Fragment) OS=Homo sapiens GN=Myh4 PE=2 SV=1 |
| Nascent polypeptide-associated complex subunit alpha, muscle-specific form OS=Homo sapiens GN=Naca PE=1 SV=2 | Myosin light chain 6B OS=Homo sapiens GN=Myl6b PE=1 SV=1 |
| Nestin OS=Homo sapiens GN=Nes PE=1 SV=1 | Myosin light polypeptide 6 OS=Homo sapiens GN=Myl6 PE=1 SV=1 |
| Nonmuscle myosin heavy chain-A (Fragment) OS=Homo sapiens GN=Myh9 PE=2 SV=1 | Myosin light polypeptide 6 OS=Homo sapiens GN=Myl6 PE=1 SV=3 |
| Nono protein OS=Homo sapiens GN=Nono PE=2 SV=1 | Myosin regulatory light chain 12B OS=Homo sapiens GN=Myl12b PE=1 SV=2 |
| Non-specific protein-tyrosine kinase (Fragment) OS=Homo sapiens GN=Lck PE=2 SV=1 | Myosin, heavy polypeptide 1, skeletal muscle, adult OS=Homo sapiens GN=Myh1 PE=2 SV=1 |
| Nuclease-sensitive element-binding protein 1 (Fragment) OS=Homo sapiens GN=Ybx1 PE=1 SV=1 | Myosin, heavy polypeptide 8, skeletal muscle, perinatal OS=Homo sapiens GN=Myh8 PE=2 SV=1 |
| Nucleolin OS=Homo sapiens GN=Ncl PE=1 SV=2 | Myosin-1 OS=Homo sapiens GN=Myh1 PE=1 SV=1 |
| NudC domain-containing protein 2 OS=Homo sapiens GN=Nudcd2 PE=1 SV=1 | Myosin-10 OS=Homo sapiens GN=Myh10 PE=1 SV=1 |
| p68 RNA helicase (Fragment) OS=Homo sapiens GN=Hlr1 PE=4 SV=1 | Myosin-10 OS=Homo sapiens GN=Myh10 PE=1 SV=2 |
| PC4 and SFRS1-interacting protein (Fragment) OS=Homo sapiens GN=Psip1 PE=1 SV=1 | Myosin-11 OS=Homo sapiens GN=Myh11 PE=1 SV=1 |
| PC4 and SFRS1-interacting protein OS=Homo sapiens GN=Psip1 PE=1 SV=1 | Myosin-14 OS=Homo sapiens GN=Myh14 PE=1 SV=1 |
| PCTAIRE-motif protein kinase 1 OS=Homo sapiens GN=Cdk16 PE=2 SV=1 | Myosin-4 OS=Homo sapiens GN=Myh4 PE=2 SV=1 |
| PCTAIRE-motif protein kinase 3 OS=Homo sapiens GN=Cdk18 PE=1 SV=1 | Myosin-8 OS=Homo sapiens GN=Myh8 PE=2 SV=2 |
| Pctk2 protein OS=Homo sapiens GN=Cdk17 PE=2 SV=1 | Myosin-9 OS=Homo sapiens GN=Myh9 PE=1 SV=1 |
| Pctk3 protein (Fragment) OS=Homo sapiens GN=Cdk18 PE=2 SV=1 | Myosin-9 OS=Homo sapiens GN=Myh9 PE=1 SV=4 |
| Peptidyl-prolyl cis-trans isomerase OS=Homo sapiens GN=Ppib PE=1 SV=1 | Nascent polypeptide-associated complex subunit alpha OS=Homo sapiens GN=Naca PE=1 SV=1 |
| Peptidyl-prolyl cis-trans isomerase OS=Homo sapiens GN=Ppib PE=2 SV=1 | Nascent polypeptide-associated complex subunit alpha, muscle-specific form OS=Homo sapiens GN=Naca PE=1 SV=2 |
| Peripherin OS=Homo sapiens GN=Prph PE=1 SV=1 | Nefl protein (Fragment) OS=Homo sapiens GN=Nefl PE=2 SV=1 |
| Peripherin OS=Homo sapiens GN=Prph PE=1 SV=2 | Neurofilament 3, medium OS=Homo sapiens GN=Nefm PE=1 SV=1 |
| Peroxiredoxin-1 (Fragment) OS=Homo sapiens GN=Prdx1 PE=1 SV=1 | Neurofilament heavy polypeptide OS=Homo sapiens GN=Nefh PE=1 SV=3 |
| Peroxiredoxin-1 (Fragment) OS=Homo sapiens GN=Prdx1 PE=1 SV=8 | Neurofilament light polypeptide OS=Homo sapiens GN=Nefl PE=1 SV=5 |
| Peroxiredoxin-1 OS=Homo sapiens GN=Prdx1 PE=1 SV=1 | Neurofilament medium polypeptide OS=Homo sapiens GN=Nefm PE=1 SV=1 |
| Pftk1 protein OS=Homo sapiens GN=Cdk14 PE=2 SV=1 | Neurofilament medium polypeptide OS=Homo sapiens GN=Nefm PE=1 SV=4 |
| Plasminogen activator inhibitor 1 RNA-binding protein (Fragment) OS=Homo sapiens GN=Serbp1 PE=1 SV=1 | Nonmuscle myosin heavy chain-A (Fragment) OS=Homo sapiens GN=Myh9 PE=2 SV=1 |
| Plasminogen activator inhibitor 1 RNA-binding protein OS=Homo sapiens GN=Serbp1 PE=1 SV=1 | Nono protein OS=Homo sapiens GN=Nono PE=2 SV=1 |
| Plasminogen activator inhibitor 1 RNA-binding protein OS=Homo sapiens GN=Serbp1 PE=1 SV=2 | Non-specific protein-tyrosine kinase (Fragment) OS=Homo sapiens GN=Lck PE=2 SV=1 |
| Plec1 protein (Fragment) OS=Homo sapiens GN=Plec PE=2 SV=1 | Nuclear receptor-binding protein OS=Homo sapiens GN=Nrbp1 PE=1 SV=1 |
| Plectin (Fragment) OS=Homo sapiens GN=Plec PE=1 SV=1 | Nuclease-sensitive element-binding protein 1 (Fragment) OS=Homo sapiens GN=Ybx1 PE=1 SV=1 |
| Plectin 10 OS=Homo sapiens GN=Plec PE=2 SV=1 | Nucleolin OS=Homo sapiens GN=Ncl PE=1 SV=2 |
| Plectin 2 OS=Homo sapiens GN=Plec PE=2 SV=1 | NudC domain-containing protein 2 OS=Homo sapiens GN=Nudcd2 PE=1 SV=1 |
| Plectin 3 OS=Homo sapiens GN=Plec PE=2 SV=1 | p68 RNA helicase (Fragment) OS=Homo sapiens GN=Hlr1 PE=4 SV=1 |
| Plectin 4 OS=Homo sapiens GN=Plec PE=2 SV=1 | PC4 and SFRS1-interacting protein (Fragment) OS=Homo sapiens GN=Psip1 PE=1 SV=1 |
| Plectin 7 OS=Homo sapiens GN=Plec PE=2 SV=1 | PC4 and SFRS1-interacting protein OS=Homo sapiens GN=Psip1 PE=1 SV=1 |
| Plectin 8 OS=Homo sapiens GN=Plec PE=2 SV=1 | Pcbp3 protein OS=Homo sapiens GN=Pcbp3 PE=2 SV=1 |
| Plectin OS=Homo sapiens GN=Plec PE=1 SV=1 | PCTAIRE-motif protein kinase 1 OS=Homo sapiens GN=Cdk16 PE=2 SV=1 |
| Plectin OS=Homo sapiens GN=Plec PE=1 SV=3 | PCTAIRE-motif protein kinase 3 OS=Homo sapiens GN=Cdk18 PE=1 SV=1 |
| Poly A binding protein, cytoplasmic 5 OS=Homo sapiens GN=Pabpc5 PE=2 SV=1 | Pctk2 protein OS=Homo sapiens GN=Cdk17 PE=2 SV=1 |
| Poly(A)-binding protein cytoplasmic 5 (Fragment) OS=Homo sapiens GN=PABPC5 PE=4 SV=1 | Pctk3 protein (Fragment) OS=Homo sapiens GN=Cdk18 PE=2 SV=1 |
| Poly(A)-binding protein, cytoplasmic 4 OS=Homo sapiens GN=Pabpc4 PE=4 SV=1 | Peptidyl-prolyl cis-trans isomerase OS=Homo sapiens GN=Ppib PE=1 SV=1 |
| Poly(rC)-binding protein 1 OS=Homo sapiens GN=Pcbp1 PE=1 SV=1 | Peptidyl-prolyl cis-trans isomerase OS=Homo sapiens GN=Ppib PE=2 SV=1 |
| Poly(U)-binding-splicing factor PUF60 OS=Homo sapiens GN=Puf60 PE=1 SV=2 | Peripherin OS=Homo sapiens GN=Prph PE=1 SV=1 |
| Polyadenylate-binding protein (Fragment) OS=Homo sapiens GN=Pabpc1 PE=2 SV=1 | Peripherin OS=Homo sapiens GN=Prph PE=1 SV=2 |
| Polyadenylate-binding protein 1 (Fragment) OS=Homo sapiens GN=Pabpc1 PE=1 SV=1 | Pftk1 protein OS=Homo sapiens GN=Cdk14 PE=2 SV=1 |
| Polyadenylate-binding protein 1 OS=Homo sapiens GN=Pabpc1 PE=1 SV=2 | Phosphatidylinositol transfer protein beta isoform OS=Homo sapiens GN=Pitpnb PE=1 SV=1 |
| Polyadenylate-binding protein OS=Homo sapiens GN=Gm10110 PE=3 SV=1 | Phosphatidylinositol transfer protein beta isoform OS=Homo sapiens GN=Pitpnb PE=1 SV=2 |
| Polyadenylate-binding protein OS=Homo sapiens GN=Pabpc1 PE=2 SV=1 | Plasminogen activator inhibitor 1 RNA-binding protein (Fragment) OS=Homo sapiens GN=Serbp1 PE=1 SV=1 |
| Polyadenylate-binding protein OS=Homo sapiens GN=Pabpc2 PE=1 SV=1 | Plasminogen activator inhibitor 1 RNA-binding protein OS=Homo sapiens GN=Serbp1 PE=1 SV=1 |
| Polyadenylate-binding protein OS=Homo sapiens GN=Pabpc4 PE=1 SV=1 | Plasminogen activator inhibitor 1 RNA-binding protein OS=Homo sapiens GN=Serbp1 PE=1 SV=2 |
| Polyadenylate-binding protein OS=Homo sapiens GN=Pabpc4 PE=2 SV=1 | Plec1 protein (Fragment) OS=Homo sapiens GN=Plec PE=2 SV=1 |
| Polyadenylate-binding protein OS=Homo sapiens GN=Pabpc6 PE=1 SV=1 | Plectin (Fragment) OS=Homo sapiens GN=Plec PE=1 SV=1 |
| Polyadenylate-binding protein OS=Homo sapiens PE=3 SV=1 | Plectin 10 OS=Homo sapiens GN=Plec PE=2 SV=1 |
| Predicted gene 10036 OS=Homo sapiens GN=Gm10036 PE=3 SV=1 | Plectin 2 OS=Homo sapiens GN=Plec PE=2 SV=1 |
| Predicted gene 10260 OS=Homo sapiens GN=Gm10260 PE=3 SV=2 | Plectin 3 OS=Homo sapiens GN=Plec PE=2 SV=1 |
| Predicted gene 14214 (Fragment) OS=Homo sapiens GN=Gm14214 PE=4 SV=1 | Plectin 4 OS=Homo sapiens GN=Plec PE=2 SV=1 |
| Predicted gene 15294 OS=Homo sapiens GN=Gm15294 PE=1 SV=1 | Plectin 7 OS=Homo sapiens GN=Plec PE=2 SV=1 |
| Predicted gene 17087 OS=Homo sapiens GN=Gm17087 PE=1 SV=1 | Plectin 8 OS=Homo sapiens GN=Plec PE=2 SV=1 |
| Predicted gene 2016 OS=Homo sapiens GN=Gm2016 PE=2 SV=1 | Plectin OS=Homo sapiens GN=Plec PE=1 SV=1 |
| Predicted gene 20425 OS=Homo sapiens GN=Gm20425 PE=4 SV=1 | Plectin OS=Homo sapiens GN=Plec PE=1 SV=3 |
| Predicted gene 2056 OS=Homo sapiens GN=Gm2056 PE=3 SV=1 | Poly [ADP-ribose] polymerase (Fragment) OS=Homo sapiens GN=Parp1 PE=2 SV=1 |
| Predicted gene 2075 OS=Homo sapiens GN=Gm2075 PE=3 SV=1 | Poly [ADP-ribose] polymerase OS=Homo sapiens GN=Parp1 PE=1 SV=1 |
| Predicted gene 2663 OS=Homo sapiens GN=Gm2663 PE=3 SV=1 | Poly [ADP-ribose] polymerase OS=Homo sapiens GN=Parp1 PE=2 SV=1 |
| Predicted gene 5039 OS=Homo sapiens GN=Gm5039 PE=3 SV=1 | Poly A binding protein, cytoplasmic 5 OS=Homo sapiens GN=Pabpc5 PE=2 SV=1 |
| Predicted gene 5093 OS=Homo sapiens GN=Gm5093 PE=1 SV=1 | Poly(A)-binding protein cytoplasmic 5 (Fragment) OS=Homo sapiens GN=PABPC5 PE=4 SV=1 |
| Predicted gene 5662 OS=Homo sapiens GN=Gm5662 PE=2 SV=1 | Poly(A)-binding protein, cytoplasmic 4 OS=Homo sapiens GN=Pabpc4 PE=4 SV=1 |
| Predicted gene 6096 OS=Homo sapiens GN=Gm6096 PE=1 SV=1 | Poly(RC) binding protein 2 OS=Homo sapiens GN=Pcbp2 PE=2 SV=1 |
| Predicted gene 6576 OS=Homo sapiens GN=Gm6576 PE=3 SV=2 | Poly(rC)-binding protein 1 OS=Homo sapiens GN=Pcbp1 PE=1 SV=1 |
| Predicted gene 6803 OS=Homo sapiens GN=Gm6803 PE=3 SV=1 | Poly(rC)-binding protein 2 OS=Homo sapiens GN=Pcbp2 PE=1 SV=1 |
| Predicted gene 8225 OS=Homo sapiens GN=Gm8225 PE=3 SV=1 | Poly(rC)-binding protein 3 OS=Homo sapiens GN=Pcbp3 PE=1 SV=1 |
| Predicted gene 8300 OS=Homo sapiens GN=Gm8332 PE=2 SV=1 | Poly(rC)-binding protein 3 OS=Homo sapiens GN=Pcbp3 PE=1 SV=3 |
| Predicted gene, 16519 OS=Homo sapiens GN=Gm16519 PE=1 SV=2 | Poly(U)-binding-splicing factor PUF60 OS=Homo sapiens GN=Puf60 PE=1 SV=2 |
| Predicted gene, 17430 OS=Homo sapiens GN=Gm17430 PE=4 SV=1 | Polyadenylate-binding protein (Fragment) OS=Homo sapiens GN=Pabpc1 PE=2 SV=1 |
| Predicted gene, 21936 OS=Homo sapiens GN=Gm21936 PE=3 SV=1 | Polyadenylate-binding protein 1 (Fragment) OS=Homo sapiens GN=Pabpc1 PE=1 SV=1 |
| Predicted pseudogene 10073 OS=Homo sapiens GN=Gm10073 PE=1 SV=1 | Polyadenylate-binding protein 1 OS=Homo sapiens GN=Pabpc1 PE=1 SV=2 |
| Predicted pseudogene 2035 OS=Homo sapiens GN=Gm2035 PE=3 SV=1 | Polyadenylate-binding protein OS=Homo sapiens GN=Gm10110 PE=3 SV=1 |
| Prelamin-A/C (Fragment) OS=Homo sapiens GN=Lmna PE=1 SV=1 | Polyadenylate-binding protein OS=Homo sapiens GN=Pabpc1 PE=2 SV=1 |
| Prelamin-A/C OS=Homo sapiens GN=Lmna PE=1 SV=2 | Polyadenylate-binding protein OS=Homo sapiens GN=Pabpc1l PE=2 SV=1 |
| Pre-mRNA-processing factor 19 OS=Homo sapiens GN=Prpf19 PE=1 SV=1 | Polyadenylate-binding protein OS=Homo sapiens GN=Pabpc2 PE=1 SV=1 |
| Pre-rRNA-processing protein TSR1 homolog OS=Homo sapiens GN=Tsr1 PE=1 SV=1 | Polyadenylate-binding protein OS=Homo sapiens GN=Pabpc4 PE=1 SV=1 |
| Probable ATP-dependent RNA helicase DDX17 OS=Homo sapiens GN=Ddx17 PE=1 SV=1 | Polyadenylate-binding protein OS=Homo sapiens GN=Pabpc4 PE=2 SV=1 |
| Probable ATP-dependent RNA helicase DDX5 (Fragment) OS=Homo sapiens GN=Ddx5 PE=1 SV=8 | Polyadenylate-binding protein OS=Homo sapiens GN=Pabpc6 PE=1 SV=1 |
| Probable ATP-dependent RNA helicase DDX5 OS=Homo sapiens GN=Ddx5 PE=1 SV=1 | Polyadenylate-binding protein OS=Homo sapiens PE=3 SV=1 |
| Probable ATP-dependent RNA helicase DDX5 OS=Homo sapiens GN=Ddx5 PE=1 SV=2 | Polypirimidine tract binding protein OS=Homo sapiens GN=Ptbp1 PE=2 SV=1 |
| Probable ATP-dependent RNA helicase DDX6 OS=Homo sapiens GN=Ddx6 PE=1 SV=1 | Polypyrimidine tract binding protein 1 OS=Homo sapiens GN=Ptbp1 PE=2 SV=1 |
| Protein 4.1G OS=Homo sapiens GN=Epb41l2 PE=2 SV=1 | Polypyrimidine tract-binding protein 1 (Fragment) OS=Homo sapiens GN=Ptbp1 PE=1 SV=1 |
| Protein arginine N-methyltransferase 3 OS=Homo sapiens GN=Prmt3 PE=1 SV=2 | Polypyrimidine tract-binding protein 1 OS=Homo sapiens GN=Ptbp1 PE=1 SV=1 |
| Protein LYRIC OS=Homo sapiens GN=Mtdh PE=1 SV=1 | Polypyrimidine tract-binding protein 1 OS=Homo sapiens GN=Ptbp1 PE=1 SV=2 |
| Protein RCC2 (Fragment) OS=Homo sapiens GN=Rcc2 PE=1 SV=1 | Predicted gene 10036 OS=Homo sapiens GN=Gm10036 PE=3 SV=1 |
| Protein RCC2 OS=Homo sapiens GN=Rcc2 PE=1 SV=1 | Predicted gene 10260 OS=Homo sapiens GN=Gm10260 PE=3 SV=2 |
| Proto-oncogene tyrosine-protein kinase LCK OS=Homo sapiens GN=Lck PE=1 SV=4 | Predicted gene 14214 (Fragment) OS=Homo sapiens GN=Gm14214 PE=4 SV=1 |
| Psip1 protein (Fragment) OS=Homo sapiens GN=Psip1 PE=2 SV=1 | Predicted gene 15294 OS=Homo sapiens GN=Gm15294 PE=1 SV=1 |
| PTB-associated splicing factor (Fragment) OS=Homo sapiens GN=Sfpq PE=2 SV=1 | Predicted gene 17087 OS=Homo sapiens GN=Gm17087 PE=1 SV=1 |
| Puf60 protein (Fragment) OS=Homo sapiens GN=Puf60 PE=2 SV=1 | Predicted gene 17190 OS=Homo sapiens GN=Gm17190 PE=1 SV=1 |
| Putative ATP-dependent RNA helicase Pl10 OS=Homo sapiens GN=D1Pas1 PE=1 SV=1 | Predicted gene 20425 OS=Homo sapiens GN=Gm20425 PE=4 SV=1 |
| Pyruvate kinase (Fragment) OS=Homo sapiens GN=Pklr PE=2 SV=1 | Predicted gene 2663 OS=Homo sapiens GN=Gm2663 PE=3 SV=1 |
| Pyruvate kinase OS=Homo sapiens GN=Pklr PE=1 SV=1 | Predicted gene 5093 OS=Homo sapiens GN=Gm5093 PE=1 SV=1 |
| Pyruvate kinase OS=Homo sapiens GN=Pklr PE=2 SV=1 | Predicted gene 6096 OS=Homo sapiens GN=Gm6096 PE=1 SV=1 |
| Pyruvate kinase PKLR OS=Homo sapiens GN=Pklr PE=1 SV=1 | Predicted gene 6576 OS=Homo sapiens GN=Gm6576 PE=3 SV=2 |
| Pyruvate kinase PKM (Fragment) OS=Homo sapiens GN=Pkm PE=1 SV=1 | Predicted gene 8225 OS=Homo sapiens GN=Gm8225 PE=3 SV=1 |
| Pyruvate kinase PKM OS=Homo sapiens GN=Pkm PE=1 SV=4 | Predicted gene, 17430 OS=Homo sapiens GN=Gm17430 PE=4 SV=1 |
| Radixin OS=Homo sapiens GN=Rdx PE=1 SV=1 | Predicted pseudogene 10073 OS=Homo sapiens GN=Gm10073 PE=1 SV=1 |
| Radixin OS=Homo sapiens GN=Rdx PE=1 SV=3 | Predicted pseudogene 9242 OS=Homo sapiens GN=Gm9242 PE=4 SV=1 |
| Ras GTPase-activating protein-binding protein 1 OS=Homo sapiens GN=G3bp1 PE=1 SV=1 | Pre-mRNA-processing factor 19 OS=Homo sapiens GN=Prpf19 PE=1 SV=1 |
| Ras GTPase-activating protein-binding protein 2 OS=Homo sapiens GN=G3bp2 PE=1 SV=2 | Pre-rRNA-processing protein TSR1 homolog OS=Homo sapiens GN=Tsr1 PE=1 SV=1 |
| Ras GTPase-activating-like protein IQGAP1 OS=Homo sapiens GN=Iqgap1 PE=1 SV=2 | Probable ATP-dependent RNA helicase DDX17 OS=Homo sapiens GN=Ddx17 PE=1 SV=1 |
| Rbm39 protein OS=Homo sapiens GN=Rbm39 PE=2 SV=1 | Probable ATP-dependent RNA helicase DDX5 (Fragment) OS=Homo sapiens GN=Ddx5 PE=1 SV=8 |
| Rdx protein (Fragment) OS=Homo sapiens GN=Rdx PE=2 SV=1 | Probable ATP-dependent RNA helicase DDX5 OS=Homo sapiens GN=Ddx5 PE=1 SV=1 |
| Receptor protein-tyrosine kinase (Fragment) OS=Homo sapiens GN=Fgfr2 PE=1 SV=1 | Probable ATP-dependent RNA helicase DDX5 OS=Homo sapiens GN=Ddx5 PE=1 SV=2 |
| Receptor protein-tyrosine kinase (Fragment) OS=Homo sapiens GN=Fgfr3 PE=2 SV=1 | Protein LYRIC OS=Homo sapiens GN=Mtdh PE=1 SV=1 |
| Ribosomal protein L14 OS=Homo sapiens GN=Rpl14-ps1 PE=2 SV=1 | Protein RCC2 (Fragment) OS=Homo sapiens GN=Rcc2 PE=1 SV=1 |
| Ribosomal protein L18 (Fragment) OS=Homo sapiens GN=Rpl18 PE=2 SV=1 | Protein RCC2 OS=Homo sapiens GN=Rcc2 PE=1 SV=1 |
| Ribosomal protein L18 OS=Homo sapiens GN=Rpl18 PE=2 SV=1 | Protein SCAF8 OS=Homo sapiens GN=Scaf8 PE=1 SV=1 |
| Ribosomal protein L19 OS=Homo sapiens GN=Rpl19 PE=1 SV=1 | Protein-serine/threonine phosphatase OS=Homo sapiens GN=Ppp1ca PE=2 SV=1 |
| Ribosomal protein L21 OS=Homo sapiens GN=Rpl21 PE=2 SV=1 | Proto-oncogene tyrosine-protein kinase LCK OS=Homo sapiens GN=Lck PE=1 SV=4 |
| Ribosomal protein L23A, pseudogene 3 OS=Homo sapiens GN=Rpl23a-ps3 PE=3 SV=1 | Psip1 protein (Fragment) OS=Homo sapiens GN=Psip1 PE=2 SV=1 |
| Ribosomal protein L3 (Fragment) OS=Homo sapiens GN=Rp13 PE=4 SV=1 | PTB-associated splicing factor (Fragment) OS=Homo sapiens GN=Sfpq PE=2 SV=1 |
| Ribosomal protein L32 OS=Homo sapiens GN=Rpl32 PE=2 SV=1 | Puf60 protein (Fragment) OS=Homo sapiens GN=Puf60 PE=2 SV=1 |
| Ribosomal protein L3-like OS=Homo sapiens GN=Rpl3l PE=1 SV=1 | Putative ATP-dependent RNA helicase Pl10 OS=Homo sapiens GN=D1Pas1 PE=1 SV=1 |
| Ribosomal protein L4 OS=Homo sapiens GN=Rpl4 PE=1 SV=1 | Putative gag-pro-pol polyprotein OS=Homo sapiens PE=4 SV=1 |
| Ribosomal protein L5 OS=Homo sapiens GN=Rpl5 PE=2 SV=1 | Putative RNA-binding protein Luc7-like 1 (Fragment) OS=Homo sapiens GN=Luc7l PE=1 SV=1 |
| Ribosomal protein L7A OS=Homo sapiens GN=Rpl7a PE=2 SV=1 | Putative RNA-binding protein Luc7-like 1 OS=Homo sapiens GN=Luc7l PE=1 SV=2 |
| Ribosomal protein L7A, pseudogene 3 OS=Homo sapiens GN=Rpl7a-ps3 PE=4 SV=1 | Putative RNA-binding protein Luc7-like 2 OS=Homo sapiens GN=Luc7l2 PE=1 SV=1 |
| Ribosomal protein L7A, pseudogene 5 OS=Homo sapiens GN=Rpl7a-ps5 PE=4 SV=1 | Pyruvate kinase (Fragment) OS=Homo sapiens GN=Pklr PE=2 SV=1 |
| Ribosomal protein L8 (Fragment) OS=Homo sapiens PE=4 SV=1 | Pyruvate kinase OS=Homo sapiens GN=Pklr PE=1 SV=1 |
| Ribosomal protein L9 OS=Homo sapiens GN=Rpl9 PE=2 SV=1 | Pyruvate kinase OS=Homo sapiens GN=Pklr PE=2 SV=1 |
| Ribosomal protein L9, pseudogene 6 OS=Homo sapiens GN=Rpl9-ps6 PE=4 SV=1 | Pyruvate kinase PKLR OS=Homo sapiens GN=Pklr PE=1 SV=1 |
| Ribosomal protein OS=Homo sapiens GN=Rpl10a PE=1 SV=1 | Pyruvate kinase PKM (Fragment) OS=Homo sapiens GN=Pkm PE=1 SV=1 |
| Ribosomal protein OS=Homo sapiens GN=Rpl10a PE=2 SV=1 | Pyruvate kinase PKM OS=Homo sapiens GN=Pkm PE=1 SV=4 |
| Ribosomal protein S14 OS=Homo sapiens GN=rps14 PE=3 SV=1 | Ras GTPase-activating protein-binding protein 1 OS=Homo sapiens GN=G3bp1 PE=1 SV=1 |
| Ribosomal protein S2 (Fragment) OS=Homo sapiens GN=Rps2 PE=2 SV=1 | Ras GTPase-activating protein-binding protein 2 OS=Homo sapiens GN=G3bp2 PE=1 SV=2 |
| Ribosomal protein S2, pseudogene 6 OS=Homo sapiens GN=Rps2-ps6 PE=3 SV=1 | Ras GTPase-activating-like protein IQGAP1 (Fragment) OS=Homo sapiens GN=Iqgap1 PE=1 SV=2 |
| Ribosomal protein S23 OS=Homo sapiens GN=Rps23 PE=2 SV=1 | Ras GTPase-activating-like protein IQGAP1 OS=Homo sapiens GN=Iqgap1 PE=1 SV=2 |
| Ribosomal protein S3 (Fragment) OS=Homo sapiens GN=Rps3 PE=2 SV=1 | Rbm39 protein OS=Homo sapiens GN=Rbm39 PE=2 SV=1 |
| Ribosomal protein S3 OS=Homo sapiens GN=Rps3 PE=1 SV=1 | Receptor protein-tyrosine kinase (Fragment) OS=Homo sapiens GN=Fgfr2 PE=1 SV=1 |
| Ribosome-binding protein 1 OS=Homo sapiens GN=Rrbp1 PE=1 SV=1 | Receptor protein-tyrosine kinase (Fragment) OS=Homo sapiens GN=Fgfr3 PE=2 SV=1 |
| Ribosome-binding protein 1 OS=Homo sapiens GN=Rrbp1 PE=1 SV=2 | Rho guanine nucleotide exchange factor 2 OS=Homo sapiens GN=Arhgef2 PE=1 SV=1 |
| RIKEN cDNA 1810009J06 gene OS=Homo sapiens GN=1810009J06Rik PE=2 SV=1 | Rho guanine nucleotide exchange factor 2 OS=Homo sapiens GN=Arhgef2 PE=1 SV=4 |
| RIKEN cDNA 2210010C04 gene OS=Homo sapiens GN=2210010C04Rik PE=1 SV=1 | Ribonuclease inhibitor (Fragment) OS=Homo sapiens GN=Rnh1 PE=1 SV=1 |
| RIKEN cDNA 9530053A07 gene OS=Homo sapiens GN=9530053A07Rik PE=1 SV=1 | Ribonuclease inhibitor OS=Homo sapiens GN=Rnh1 PE=1 SV=1 |
| RNA and export factor binding protein 2 OS=Homo sapiens GN=Alyref2 PE=2 SV=1 | Ribosomal protein L18 (Fragment) OS=Homo sapiens GN=Rpl18 PE=2 SV=1 |
| RNA and export factor binding protein 2 OS=Homo sapiens GN=Refbp2 PE=4 SV=1 | Ribosomal protein L18 OS=Homo sapiens GN=Rpl18 PE=2 SV=1 |
| RNA-binding protein 39 (Fragment) OS=Homo sapiens GN=Rbm39 PE=1 SV=1 | Ribosomal protein L19 OS=Homo sapiens GN=Rpl19 PE=1 SV=1 |
| RNA-binding protein 39 OS=Homo sapiens GN=Rbm39 PE=1 SV=1 | Ribosomal protein L21 OS=Homo sapiens GN=Rpl21 PE=2 SV=1 |
| RNA-binding protein 39 OS=Homo sapiens GN=Rbm39 PE=1 SV=2 | Ribosomal protein L23A, pseudogene 3 OS=Homo sapiens GN=Rpl23a-ps3 PE=3 SV=1 |
| RNA-binding protein EWS OS=Homo sapiens GN=Ewsr1 PE=1 SV=1 | Ribosomal protein L3 (Fragment) OS=Homo sapiens GN=Rp13 PE=4 SV=1 |
| RNA-binding protein EWS OS=Homo sapiens GN=Ewsr1 PE=1 SV=2 | Ribosomal protein L3-like OS=Homo sapiens GN=Rpl3l PE=1 SV=1 |
| RNA-binding protein FUS (Fragment) OS=Homo sapiens GN=Fus PE=1 SV=1 | Ribosomal protein L4 (Fragment) OS=Homo sapiens GN=Rpl4 PE=2 SV=1 |
| Rpl11 protein OS=Homo sapiens GN=Rpl11 PE=2 SV=1 | Ribosomal protein L4 OS=Homo sapiens GN=Rpl4 PE=1 SV=1 |
| Rpl12 protein (Fragment) OS=Homo sapiens GN=Rpl12 PE=2 SV=1 | Ribosomal protein L7A OS=Homo sapiens GN=Rpl7a PE=2 SV=1 |
| Rpl17 protein (Fragment) OS=Homo sapiens GN=Rpl17 PE=2 SV=1 | Ribosomal protein L7A, pseudogene 3 OS=Homo sapiens GN=Rpl7a-ps3 PE=4 SV=1 |
| Rpl17 protein OS=Homo sapiens GN=Rpl17 PE=2 SV=1 | Ribosomal protein L7A, pseudogene 5 OS=Homo sapiens GN=Rpl7a-ps5 PE=4 SV=1 |
| Rpl23a protein (Fragment) OS=Homo sapiens GN=Rpl23a PE=2 SV=1 | Ribosomal protein L8 (Fragment) OS=Homo sapiens PE=4 SV=1 |
| Rpl30 protein OS=Homo sapiens GN=Rpl30 PE=2 SV=1 | Ribosomal protein L9 OS=Homo sapiens GN=Rpl9 PE=2 SV=1 |
| Rpl31 protein OS=Homo sapiens GN=Rpl31 PE=2 SV=1 | Ribosomal protein L9, pseudogene 6 OS=Homo sapiens GN=Rpl9-ps6 PE=4 SV=1 |
| Rpl3l protein (Fragment) OS=Homo sapiens GN=Rpl3l PE=2 SV=1 | Ribosomal protein S14 OS=Homo sapiens GN=rps14 PE=3 SV=1 |
| Rpl7a protein (Fragment) OS=Homo sapiens GN=Rpl7a PE=2 SV=1 | Ribosomal protein S2 (Fragment) OS=Homo sapiens GN=Rps2 PE=2 SV=1 |
| Rps16 protein (Fragment) OS=Homo sapiens GN=Rps16 PE=2 SV=1 | Ribosomal protein S2, pseudogene 6 OS=Homo sapiens GN=Rps2-ps6 PE=3 SV=1 |
| Rps16 protein OS=Homo sapiens GN=Rps16 PE=2 SV=1 | Ribosomal protein S23 OS=Homo sapiens GN=Rps23 PE=2 SV=1 |
| Rps19 protein (Fragment) OS=Homo sapiens GN=Rps19 PE=2 SV=1 | Ribosomal protein S3 (Fragment) OS=Homo sapiens GN=Rps3 PE=2 SV=1 |
| Rps9 protein (Fragment) OS=Homo sapiens GN=Rps9 PE=2 SV=1 | Ribosomal protein S3 OS=Homo sapiens GN=Rps3 PE=1 SV=1 |
| Serine/arginine-rich splicing factor 1 OS=Homo sapiens GN=Srsf1 PE=1 SV=3 | Ribosome-binding protein 1 OS=Homo sapiens GN=Rrbp1 PE=1 SV=1 |
| Serine/arginine-rich splicing factor 2 OS=Homo sapiens GN=Srsf2 PE=1 SV=4 | Ribosome-binding protein 1 OS=Homo sapiens GN=Rrbp1 PE=1 SV=2 |
| Serine/arginine-rich splicing factor 3 OS=Homo sapiens GN=Srsf3 PE=1 SV=1 | RIKEN cDNA 1810009J06 gene OS=Homo sapiens GN=1810009J06Rik PE=2 SV=1 |
| Serine/arginine-rich splicing factor 4 OS=Homo sapiens GN=Srsf4 PE=2 SV=1 | RIKEN cDNA 2210010C04 gene OS=Homo sapiens GN=2210010C04Rik PE=1 SV=1 |
| Serine/arginine-rich splicing factor 5 OS=Homo sapiens GN=Srsf5 PE=1 SV=2 | RIKEN cDNA 9530053A07 gene OS=Homo sapiens GN=9530053A07Rik PE=1 SV=1 |
| Serine/arginine-rich splicing factor 6 OS=Homo sapiens GN=Srsf6 PE=1 SV=1 | RNA and export factor binding protein 2 OS=Homo sapiens GN=Alyref2 PE=2 SV=1 |
| Serine/arginine-rich splicing factor 7 OS=Homo sapiens GN=Srsf7 PE=1 SV=1 | RNA and export factor binding protein 2 OS=Homo sapiens GN=Refbp2 PE=4 SV=1 |
| Serine/arginine-rich-splicing factor 1 (Fragment) OS=Homo sapiens GN=Srsf1 PE=1 SV=1 | RNA binding motif protein, X chromosome, isoform CRA_b OS=Homo sapiens GN=Rbmx PE=1 SV=1 |
| Serine/arginine-rich-splicing factor 1 OS=Homo sapiens GN=Srsf1 PE=1 SV=1 | RNA binding motif protein, X-linked-like-1 OS=Homo sapiens GN=Rbmxl1 PE=1 SV=1 |
| Serine/arginine-rich-splicing factor 11 (Fragment) OS=Homo sapiens GN=Srsf11 PE=1 SV=1 | RNA-binding motif protein, X chromosome (Fragment) OS=Homo sapiens GN=Rbmx PE=1 SV=1 |
| Serine/arginine-rich-splicing factor 11 OS=Homo sapiens GN=Srsf11 PE=1 SV=1 | RNA-binding motif protein, X chromosome OS=Homo sapiens GN=Rbmx PE=1 SV=1 |
| Serine/arginine-rich-splicing factor 4 OS=Homo sapiens GN=Srsf4 PE=1 SV=1 | RNA-binding motif, single-stranded-interacting protein 2 OS=Homo sapiens GN=Rbms2 PE=1 SV=1 |
| Serine/threonine-protein kinase ICK OS=Homo sapiens GN=Ick PE=1 SV=1 | RNA-binding protein 14 OS=Homo sapiens GN=Rbm14 PE=1 SV=1 |
| Serine/threonine-protein kinase ICK OS=Homo sapiens GN=Ick PE=1 SV=2 | RNA-binding protein 3 OS=Homo sapiens GN=Rbm3 PE=1 SV=1 |
| Serine/threonine-protein kinase MAK (Fragment) OS=Homo sapiens GN=Mak PE=3 SV=1 | RNA-binding protein 39 (Fragment) OS=Homo sapiens GN=Rbm39 PE=1 SV=1 |
| Serine/threonine-protein kinase MAK OS=Homo sapiens GN=Mak PE=1 SV=2 | RNA-binding protein 39 OS=Homo sapiens GN=Rbm39 PE=1 SV=1 |
| Serine/threonine-protein kinase MAK OS=Homo sapiens GN=Mak PE=4 SV=1 | RNA-binding protein 39 OS=Homo sapiens GN=Rbm39 PE=1 SV=2 |
| Sfpq protein (Fragment) OS=Homo sapiens GN=Sfpq PE=2 SV=1 | RNA-binding protein EWS OS=Homo sapiens GN=Ewsr1 PE=1 SV=1 |
| Sfrs11 protein (Fragment) OS=Homo sapiens GN=Srsf11 PE=2 SV=1 | RNA-binding protein EWS OS=Homo sapiens GN=Ewsr1 PE=1 SV=2 |
| Sfrs4 protein OS=Homo sapiens GN=Srsf4 PE=2 SV=1 | RNA-binding protein FUS (Fragment) OS=Homo sapiens GN=Fus PE=1 SV=1 |
| Sfrs5 protein OS=Homo sapiens GN=Srsf5 PE=2 SV=1 | Rpl11 protein OS=Homo sapiens GN=Rpl11 PE=2 SV=1 |
| Signal recognition particle receptor subunit beta OS=Homo sapiens GN=Srprb PE=1 SV=1 | Rpl12 protein (Fragment) OS=Homo sapiens GN=Rpl12 PE=2 SV=1 |
| Skeletal muscle alpha-actin mRNA (Fragment) OS=Homo sapiens GN=Acta1 PE=2 SV=1 | Rpl17 protein (Fragment) OS=Homo sapiens GN=Rpl17 PE=2 SV=1 |
| Small nuclear ribonucleoprotein 70 (U1) OS=Homo sapiens GN=Snrnp70 PE=2 SV=1 | Rpl17 protein OS=Homo sapiens GN=Rpl17 PE=2 SV=1 |
| Small nuclear ribonucleoprotein D3 OS=Homo sapiens GN=Snrpd3 PE=2 SV=1 | Rpl23a protein (Fragment) OS=Homo sapiens GN=Rpl23a PE=2 SV=1 |
| Small nuclear ribonucleoprotein Sm D1 OS=Homo sapiens GN=Snrpd1 PE=1 SV=1 | Rpl30 protein OS=Homo sapiens GN=Rpl30 PE=2 SV=1 |
| Small nuclear ribonucleoprotein Sm D3 OS=Homo sapiens GN=Snrpd3 PE=1 SV=1 | Rpl31 protein OS=Homo sapiens GN=Rpl31 PE=2 SV=1 |
| Small nuclear ribonucleoprotein-associated protein B OS=Homo sapiens GN=Snrpb PE=1 SV=1 | Rpl3l protein (Fragment) OS=Homo sapiens GN=Rpl3l PE=2 SV=1 |
| Small nuclear ribonucleoprotein-associated protein N OS=Homo sapiens GN=Snrpn PE=1 SV=1 | Rpl7a protein (Fragment) OS=Homo sapiens GN=Rpl7a PE=2 SV=1 |
| Small nuclear ribonucleoprotein-associated protein OS=Homo sapiens GN=Snrpn PE=3 SV=1 | Rps16 protein (Fragment) OS=Homo sapiens GN=Rps16 PE=2 SV=1 |
| Smooth muscle gamma-actin OS=Homo sapiens PE=3 SV=1 | Rps16 protein OS=Homo sapiens GN=Rps16 PE=2 SV=1 |
| Snrnp70 protein (Fragment) OS=Homo sapiens GN=Snrnp70 PE=2 SV=1 | Rps19 protein (Fragment) OS=Homo sapiens GN=Rps19 PE=2 SV=1 |
| Snrnp70 protein OS=Homo sapiens GN=Snrnp70 PE=2 SV=1 | Rps9 protein (Fragment) OS=Homo sapiens GN=Rps9 PE=2 SV=1 |
| Solute carrier family 25 (Mitochondrial carrier adenine nucleotide translocator), member 31 OS=Homo sapiens GN=Slc25a31 PE=1 SV=1 | Sarcoplasmic/endoplasmic reticulum calcium ATPase 1 (Fragment) OS=Homo sapiens GN=Atp2a1 PE=1 SV=1 |
| Spermatid-specific heat shock protein 70 (Fragment) OS=Homo sapiens GN=Hsc70t PE=3 SV=1 | Sarcoplasmic/endoplasmic reticulum calcium ATPase 1 OS=Homo sapiens GN=Atp2a1 PE=1 SV=1 |
| Splicing factor 3b, subunit 3 OS=Homo sapiens GN=Sf3b3 PE=2 SV=1 | Scr3 OS=Homo sapiens GN=Rbms2 PE=2 SV=1 |
| Splicing factor U2AF 65 kDa subunit OS=Homo sapiens GN=U2af2 PE=1 SV=3 | Secreted frizzled-related protein 5 OS=Homo sapiens GN=Sfrp5 PE=2 SV=1 |
| Splicing factor, proline- and glutamine-rich OS=Homo sapiens GN=Sfpq PE=1 SV=1 | Secreted frizzled-related protein 5 OS=Homo sapiens GN=Sfrp5 PE=4 SV=1 |
| Synaptic functional regulator FMR1 OS=Homo sapiens GN=Fmr1 PE=1 SV=1 | Serine/arginine repetitive matrix protein 1 (Fragment) OS=Homo sapiens GN=Srrm1 PE=1 SV=1 |
| TAF15 RNA polymerase II, TATA box binding protein (TBP)-associated factor OS=Homo sapiens GN=Taf15 PE=1 SV=1 | Serine/arginine-rich splicing factor 1 OS=Homo sapiens GN=Srsf1 PE=1 SV=3 |
| TATA-box-binding protein-associated factor 15 (Fragment) OS=Homo sapiens GN=Taf15 PE=1 SV=1 | Serine/arginine-rich splicing factor 2 OS=Homo sapiens GN=Srsf2 PE=1 SV=4 |
| T-complex protein 1 subunit beta (Fragment) OS=Homo sapiens GN=Cct2 PE=1 SV=1 | Serine/arginine-rich splicing factor 3 OS=Homo sapiens GN=Srsf3 PE=1 SV=1 |
| T-complex protein 1 subunit beta OS=Homo sapiens GN=Cct2 PE=1 SV=1 | Serine/arginine-rich splicing factor 4 OS=Homo sapiens GN=Srsf4 PE=2 SV=1 |
| THO complex subunit 4 OS=Homo sapiens GN=Alyref PE=1 SV=3 | Serine/arginine-rich splicing factor 5 OS=Homo sapiens GN=Srsf5 PE=1 SV=2 |
| Translin-associated factor X-interacting protein 1 OS=Homo sapiens GN=Tsnaxip1 PE=1 SV=1 | Serine/arginine-rich splicing factor 6 OS=Homo sapiens GN=Srsf6 PE=1 SV=1 |
| Translin-associated factor X-interacting protein 1 OS=Homo sapiens GN=Tsnaxip1 PE=1 SV=2 | Serine/arginine-rich splicing factor 7 OS=Homo sapiens GN=Srsf7 PE=1 SV=1 |
| Trypsinogen 5 OS=Homo sapiens GN=trypsinogen PE=3 SV=1 | Serine/arginine-rich-splicing factor 1 (Fragment) OS=Homo sapiens GN=Srsf1 PE=1 SV=1 |
| Tubb2a protein (Fragment) OS=Homo sapiens GN=Tubb2a PE=2 SV=1 | Serine/arginine-rich-splicing factor 1 OS=Homo sapiens GN=Srsf1 PE=1 SV=1 |
| Tubb5 protein (Fragment) OS=Homo sapiens GN=Tubb5 PE=2 SV=1 | Serine/arginine-rich-splicing factor 11 (Fragment) OS=Homo sapiens GN=Srsf11 PE=1 SV=1 |
| Tubulin alpha chain (Fragment) OS=Homo sapiens GN=Tuba1b PE=2 SV=1 | Serine/arginine-rich-splicing factor 11 OS=Homo sapiens GN=Srsf11 PE=1 SV=1 |
| Tubulin alpha chain (Fragment) OS=Homo sapiens GN=Tuba4a PE=1 SV=1 | Serine/arginine-rich-splicing factor 4 OS=Homo sapiens GN=Srsf4 PE=1 SV=1 |
| Tubulin alpha chain OS=Homo sapiens GN=Tuba1c PE=1 SV=1 | Serine/threonine-protein kinase ICK OS=Homo sapiens GN=Ick PE=1 SV=1 |
| Tubulin alpha chain OS=Homo sapiens GN=Tuba1c PE=2 SV=1 | Serine/threonine-protein kinase ICK OS=Homo sapiens GN=Ick PE=1 SV=2 |
| Tubulin alpha-1A chain OS=Homo sapiens GN=Tuba1a PE=1 SV=1 | Serine/threonine-protein kinase MAK (Fragment) OS=Homo sapiens GN=Mak PE=3 SV=1 |
| Tubulin alpha-1B chain OS=Homo sapiens GN=Tuba1b PE=1 SV=2 | Serine/threonine-protein kinase MAK OS=Homo sapiens GN=Mak PE=1 SV=2 |
| Tubulin alpha-3 chain OS=Homo sapiens GN=Tuba3a PE=1 SV=1 | Serine/threonine-protein kinase MAK OS=Homo sapiens GN=Mak PE=4 SV=1 |
| Tubulin alpha-4A chain (Fragment) OS=Homo sapiens GN=Tuba4a PE=1 SV=1 | Serine/threonine-protein phosphatase (Fragment) OS=Homo sapiens GN=Ppp1ca PE=2 SV=1 |
| Tubulin alpha-4A chain OS=Homo sapiens GN=Tuba4a PE=1 SV=1 | Serine/threonine-protein phosphatase OS=Homo sapiens GN=mCG_126872 PE=2 SV=1 |
| Tubulin alpha-8 chain OS=Homo sapiens GN=Tuba8 PE=1 SV=1 | Serine/threonine-protein phosphatase OS=Homo sapiens GN=Ppp1cc PE=1 SV=1 |
| Tubulin beta chain (Fragment) OS=Homo sapiens GN=Tubb4b PE=2 SV=2 | Serine/threonine-protein phosphatase OS=Homo sapiens PE=2 SV=1 |
| Tubulin beta chain OS=Homo sapiens GN=Tubb2b PE=1 SV=1 | Serine/threonine-protein phosphatase PP1-alpha catalytic subunit OS=Homo sapiens GN=Ppp1ca PE=1 SV=1 |
| Tubulin beta chain OS=Homo sapiens GN=Tubb6 PE=2 SV=1 | Serine/threonine-protein phosphatase PP1-beta catalytic subunit OS=Homo sapiens GN=Ppp1cb PE=1 SV=3 |
| Tubulin beta-2A chain OS=Homo sapiens GN=Tubb2a PE=1 SV=1 | Sfpq protein (Fragment) OS=Homo sapiens GN=Sfpq PE=2 SV=1 |
| Tubulin beta-3 chain OS=Homo sapiens GN=Tubb3 PE=1 SV=1 | Sfrs11 protein (Fragment) OS=Homo sapiens GN=Srsf11 PE=2 SV=1 |
| Tubulin beta-4A chain OS=Homo sapiens GN=Tubb4a PE=1 SV=3 | Sfrs4 protein OS=Homo sapiens GN=Srsf4 PE=2 SV=1 |
| Tubulin beta-4B chain OS=Homo sapiens GN=Tubb4b PE=1 SV=1 | Sfrs5 protein OS=Homo sapiens GN=Srsf5 PE=2 SV=1 |
| Tubulin beta-5 chain (Fragment) OS=Homo sapiens GN=Tubb5 PE=1 SV=8 | Signal recognition particle receptor subunit beta OS=Homo sapiens GN=Srprb PE=1 SV=1 |
| Tubulin beta-5 chain OS=Homo sapiens GN=Tubb5 PE=1 SV=1 | Skeletal muscle alpha-actin mRNA (Fragment) OS=Homo sapiens GN=Acta1 PE=2 SV=1 |
| Tubulin beta-6 chain OS=Homo sapiens GN=Tubb6 PE=1 SV=1 | Small nuclear ribonucleoprotein 70 (U1) OS=Homo sapiens GN=Snrnp70 PE=2 SV=1 |
| Type II cytokeratin Kb39 (Fragment) OS=Homo sapiens GN=Krt77 PE=2 SV=1 | Small nuclear ribonucleoprotein D3 OS=Homo sapiens GN=Snrpd3 PE=2 SV=1 |
| Tyrosine-protein kinase Blk OS=Homo sapiens GN=Blk PE=1 SV=4 | Small nuclear ribonucleoprotein Sm D1 OS=Homo sapiens GN=Snrpd1 PE=1 SV=1 |
| Tyrosine-protein kinase Fgr OS=Homo sapiens GN=Fgr PE=1 SV=2 | Small nuclear ribonucleoprotein Sm D3 OS=Homo sapiens GN=Snrpd3 PE=1 SV=1 |
| Tyrosine-protein kinase Fyn OS=Homo sapiens GN=Fyn PE=1 SV=4 | Small nuclear ribonucleoprotein-associated protein B OS=Homo sapiens GN=Snrpb PE=1 SV=1 |
| Tyrosine-protein kinase OS=Homo sapiens GN=Blk PE=2 SV=1 | Small nuclear ribonucleoprotein-associated protein N OS=Homo sapiens GN=Snrpn PE=1 SV=1 |
| Tyrosine-protein kinase OS=Homo sapiens GN=Fgr PE=2 SV=1 | Small nuclear ribonucleoprotein-associated protein OS=Homo sapiens GN=Snrpn PE=3 SV=1 |
| Tyrosine-protein kinase OS=Homo sapiens GN=Fyn PE=1 SV=1 | Smooth muscle gamma-actin OS=Homo sapiens PE=3 SV=1 |
| Tyrosine-protein kinase OS=Homo sapiens GN=Hck PE=1 SV=1 | Snrnp70 protein (Fragment) OS=Homo sapiens GN=Snrnp70 PE=2 SV=1 |
| Tyrosine-protein kinase OS=Homo sapiens GN=Lck PE=1 SV=1 | Snrnp70 protein OS=Homo sapiens GN=Snrnp70 PE=2 SV=1 |
| Tyrosine-protein kinase OS=Homo sapiens GN=Lyn PE=2 SV=1 | Solute carrier family 25 (Mitochondrial carrier adenine nucleotide translocator), member 31 OS=Homo sapiens GN=Slc25a31 PE=1 SV=1 |
| Tyrosine-protein kinase OS=Homo sapiens GN=Yes1 PE=2 SV=1 | Spermatid-specific heat shock protein 70 (Fragment) OS=Homo sapiens GN=Hsc70t PE=3 SV=1 |
| Tyrosine-protein kinase Yes OS=Homo sapiens GN=Yes1 PE=1 SV=3 | Splicing factor 3b, subunit 3 OS=Homo sapiens GN=Sf3b3 PE=2 SV=1 |
| U1 small nuclear ribonucleoprotein 70 kDa (Fragment) OS=Homo sapiens GN=Snrnp70 PE=1 SV=1 | Splicing factor U2AF 26 kDa subunit (Fragment) OS=Homo sapiens GN=U2af1l4 PE=4 SV=1 |
| U1 small nuclear ribonucleoprotein 70 kDa OS=Homo sapiens GN=Snrnp70 PE=1 SV=1 | Splicing factor U2AF 26 kDa subunit OS=Homo sapiens GN=U2af1l4 PE=1 SV=1 |
| U1 small nuclear ribonucleoprotein A (Fragment) OS=Homo sapiens GN=Snrpa PE=1 SV=1 | Splicing factor U2AF 26 kDa subunit OS=Homo sapiens GN=U2af1l4 PE=4 SV=1 |
| U1 small nuclear ribonucleoprotein A OS=Homo sapiens GN=Snrpa PE=1 SV=1 | Splicing factor U2AF 65 kDa subunit OS=Homo sapiens GN=U2af2 PE=1 SV=3 |
| U1 small nuclear ribonucleoprotein A OS=Homo sapiens GN=Snrpa PE=1 SV=3 | Splicing factor, proline- and glutamine-rich OS=Homo sapiens GN=Sfpq PE=1 SV=1 |
| U2 snRNP auxiliary factor large subunit (Fragment) OS=Homo sapiens GN=U2af2 PE=2 SV=1 | SR-related CTD-associated factor 4 OS=Homo sapiens GN=Scaf4 PE=1 SV=1 |
| U2 snRNP auxiliary factor large subunit OS=Homo sapiens GN=U2af2 PE=1 SV=1 | Staphylococcal nuclease domain-containing protein 1 OS=Homo sapiens GN=Snd1 PE=1 SV=1 |
| U2 snRNP auxiliary factor large subunit OS=Homo sapiens GN=U2af2 PE=2 SV=1 | Synaptic functional regulator FMR1 OS=Homo sapiens GN=Fmr1 PE=1 SV=1 |
| U4/U6.U5 tri-snRNP-associated protein 2 OS=Homo sapiens GN=Usp39 PE=1 SV=2 | TAF15 RNA polymerase II, TATA box binding protein (TBP)-associated factor OS=Homo sapiens GN=Taf15 PE=1 SV=1 |
| Ubiquitin associated protein 2-like, isoform CRA_b OS=Homo sapiens GN=Ubap2l PE=1 SV=1 | TATA-box-binding protein-associated factor 15 (Fragment) OS=Homo sapiens GN=Taf15 PE=1 SV=1 |
| Ubiquitin associated protein 2-like, isoform CRA_g OS=Homo sapiens GN=Ubap2l PE=1 SV=1 | THO complex subunit 4 OS=Homo sapiens GN=Alyref PE=1 SV=3 |
| Ubiquitin-associated protein 2-like (Fragment) OS=Homo sapiens GN=Ubap2l PE=1 SV=1 | Tpm1 protein OS=Homo sapiens GN=Tpm1 PE=1 SV=1 |
| Ubiquitin-associated protein 2-like OS=Homo sapiens GN=Ubap2l PE=1 SV=1 | Tpm2 protein OS=Homo sapiens GN=Tpm2 PE=2 SV=1 |
| Vim protein (Fragment) OS=Homo sapiens GN=Vim PE=2 SV=1 | Tpm3 protein OS=Homo sapiens GN=Tpm3 PE=2 SV=1 |
| Vimentin (Fragment) OS=Homo sapiens GN=Vim PE=1 SV=2 | Translin-associated factor X-interacting protein 1 OS=Homo sapiens GN=Tsnaxip1 PE=1 SV=1 |
| Vimentin OS=Homo sapiens GN=Vim PE=1 SV=1 | Translin-associated factor X-interacting protein 1 OS=Homo sapiens GN=Tsnaxip1 PE=1 SV=2 |
| Vimentin OS=Homo sapiens GN=Vim PE=1 SV=3 | tRNA-splicing ligase RtcB homolog OS=Homo sapiens GN=Rtcb PE=1 SV=1 |
| Y box protein 1 OS=Homo sapiens GN=Ybx1 PE=1 SV=1 | tRNA-splicing ligase RtcB homolog OS=Homo sapiens GN=Rtcb PE=2 SV=1 |
| Y box protein 1 OS=Homo sapiens GN=Ybx1 PE=2 SV=1 | Tropomyosin 1, alpha OS=Homo sapiens GN=Tpm1 PE=2 SV=1 |
| Y box protein 2 OS=Homo sapiens GN=Ybx2 PE=1 SV=1 | Tropomyosin 1, alpha, isoform CRA_c OS=Homo sapiens GN=Tpm1 PE=1 SV=1 |
| Y box transcription factor (Fragment) OS=Homo sapiens GN=Ybx1 PE=2 SV=1 | Tropomyosin 1, alpha, isoform CRA_i OS=Homo sapiens GN=Tpm1 PE=1 SV=1 |
| Y-box binding protein (Fragment) OS=Homo sapiens GN=Igf2bp3 PE=2 SV=1 | Tropomyosin 1, alpha, isoform CRA_j OS=Homo sapiens GN=Tpm1 PE=1 SV=1 |
| Y-box-binding protein 2 OS=Homo sapiens GN=Ybx2 PE=1 SV=1 | Tropomyosin 1, alpha, isoform CRA_k OS=Homo sapiens GN=Tpm1 PE=1 SV=1 |
| Y-box-binding protein 3 OS=Homo sapiens GN=Ybx3 PE=1 SV=2 | Tropomyosin 1, alpha, isoform CRA_l OS=Homo sapiens GN=Tpm1 PE=2 SV=1 |
| Ybx2 protein OS=Homo sapiens GN=Ybx2 PE=2 SV=1 | Tropomyosin 3, gamma OS=Homo sapiens GN=Tpm3 PE=2 SV=1 |
|  | Tropomyosin 3, related sequence 7 OS=Homo sapiens GN=Tpm3-rs7 PE=3 SV=1 |
|  | Tropomyosin alpha-1 chain OS=Homo sapiens GN=Tpm1 PE=1 SV=1 |
|  | Tropomyosin alpha-3 chain OS=Homo sapiens GN=Tpm3 PE=1 SV=1 |
|  | Tropomyosin alpha-3 chain OS=Homo sapiens GN=Tpm3 PE=1 SV=3 |
|  | Tropomyosin beta chain (Fragment) OS=Homo sapiens GN=Tpm2 PE=1 SV=8 |
|  | Tropomyosin beta chain OS=Homo sapiens GN=Tpm2 PE=1 SV=1 |
|  | Trypsinogen 5 OS=Homo sapiens GN=trypsinogen PE=3 SV=1 |
|  | Tubb2a protein (Fragment) OS=Homo sapiens GN=Tubb2a PE=2 SV=1 |
|  | Tubb5 protein (Fragment) OS=Homo sapiens GN=Tubb5 PE=2 SV=1 |
|  | Tubulin alpha chain (Fragment) OS=Homo sapiens GN=Tuba1b PE=2 SV=1 |
|  | Tubulin alpha chain (Fragment) OS=Homo sapiens GN=Tuba4a PE=1 SV=1 |
|  | Tubulin alpha chain OS=Homo sapiens GN=Tuba1c PE=1 SV=1 |
|  | Tubulin alpha chain OS=Homo sapiens GN=Tuba1c PE=2 SV=1 |
|  | Tubulin alpha-1A chain OS=Homo sapiens GN=Tuba1a PE=1 SV=1 |
|  | Tubulin alpha-1B chain OS=Homo sapiens GN=Tuba1b PE=1 SV=2 |
|  | Tubulin alpha-3 chain OS=Homo sapiens GN=Tuba3a PE=1 SV=1 |
|  | Tubulin alpha-4A chain OS=Homo sapiens GN=Tuba4a PE=1 SV=1 |
|  | Tubulin alpha-8 chain OS=Homo sapiens GN=Tuba8 PE=1 SV=1 |
|  | Tubulin beta chain OS=Homo sapiens GN=Tubb2b PE=1 SV=1 |
|  | Tubulin beta chain OS=Homo sapiens GN=Tubb6 PE=2 SV=1 |
|  | Tubulin beta-2A chain OS=Homo sapiens GN=Tubb2a PE=1 SV=1 |
|  | Tubulin beta-3 chain OS=Homo sapiens GN=Tubb3 PE=1 SV=1 |
|  | Tubulin beta-4A chain OS=Homo sapiens GN=Tubb4a PE=1 SV=3 |
|  | Tubulin beta-4B chain OS=Homo sapiens GN=Tubb4b PE=1 SV=1 |
|  | Tubulin beta-5 chain OS=Homo sapiens GN=Tubb5 PE=1 SV=1 |
|  | Tubulin beta-6 chain OS=Homo sapiens GN=Tubb6 PE=1 SV=1 |
|  | Type II cytokeratin Kb39 (Fragment) OS=Homo sapiens GN=Krt77 PE=2 SV=1 |
|  | Tyrosine-protein kinase Blk OS=Homo sapiens GN=Blk PE=1 SV=4 |
|  | Tyrosine-protein kinase Fgr OS=Homo sapiens GN=Fgr PE=1 SV=2 |
|  | Tyrosine-protein kinase Fyn OS=Homo sapiens GN=Fyn PE=1 SV=4 |
|  | Tyrosine-protein kinase OS=Homo sapiens GN=Blk PE=2 SV=1 |
|  | Tyrosine-protein kinase OS=Homo sapiens GN=Fgr PE=2 SV=1 |
|  | Tyrosine-protein kinase OS=Homo sapiens GN=Fyn PE=1 SV=1 |
|  | Tyrosine-protein kinase OS=Homo sapiens GN=Hck PE=1 SV=1 |
|  | Tyrosine-protein kinase OS=Homo sapiens GN=Lck PE=1 SV=1 |
|  | Tyrosine-protein kinase OS=Homo sapiens GN=Lyn PE=2 SV=1 |
|  | Tyrosine-protein kinase OS=Homo sapiens GN=Yes1 PE=2 SV=1 |
|  | Tyrosine-protein kinase Yes OS=Homo sapiens GN=Yes1 PE=1 SV=3 |
|  | U1 small nuclear ribonucleoprotein 70 kDa (Fragment) OS=Homo sapiens GN=Snrnp70 PE=1 SV=1 |
|  | U1 small nuclear ribonucleoprotein 70 kDa OS=Homo sapiens GN=Snrnp70 PE=1 SV=1 |
|  | U1 small nuclear ribonucleoprotein A (Fragment) OS=Homo sapiens GN=Snrpa PE=1 SV=1 |
|  | U1 small nuclear ribonucleoprotein A OS=Homo sapiens GN=Snrpa PE=1 SV=3 |
|  | U2 snRNP auxiliary factor large subunit (Fragment) OS=Homo sapiens GN=U2af2 PE=2 SV=1 |
|  | U2 snRNP auxiliary factor large subunit OS=Homo sapiens GN=U2af2 PE=1 SV=1 |
|  | U2 snRNP auxiliary factor large subunit OS=Homo sapiens GN=U2af2 PE=2 SV=1 |
|  | U4/U6.U5 tri-snRNP-associated protein 1 OS=Homo sapiens GN=Sart1 PE=1 SV=1 |
|  | Ubiquitin associated protein 2-like, isoform CRA_b OS=Homo sapiens GN=Ubap2l PE=1 SV=1 |
|  | Ubiquitin associated protein 2-like, isoform CRA_g OS=Homo sapiens GN=Ubap2l PE=1 SV=1 |
|  | Ubiquitin-associated protein 2-like (Fragment) OS=Homo sapiens GN=Ubap2l PE=1 SV=1 |
|  | Ubiquitin-associated protein 2-like OS=Homo sapiens GN=Ubap2l PE=1 SV=1 |
|  | Vim protein (Fragment) OS=Homo sapiens GN=Vim PE=2 SV=1 |
|  | Vimentin (Fragment) OS=Homo sapiens GN=Vim PE=1 SV=2 |
|  | Vimentin OS=Homo sapiens GN=Vim PE=1 SV=1 |
|  | Vimentin OS=Homo sapiens GN=Vim PE=1 SV=3 |
|  | V-type proton ATPase 116 kDa subunit a isoform 1 OS=Homo sapiens GN=Atp6v0a1 PE=1 SV=3 |
|  | V-type proton ATPase subunit a OS=Homo sapiens GN=Atp6v0a1 PE=1 SV=1 |
|  | V-type proton ATPase subunit a OS=Homo sapiens GN=Atp6v0a1 PE=2 SV=1 |
|  | Y box protein 1 OS=Homo sapiens GN=Ybx1 PE=1 SV=1 |
|  | Y box protein 1 OS=Homo sapiens GN=Ybx1 PE=2 SV=1 |
|  | Y box protein 2 OS=Homo sapiens GN=Ybx2 PE=1 SV=1 |
|  | Y box transcription factor (Fragment) OS=Homo sapiens GN=Ybx1 PE=2 SV=1 |
|  | Y-box binding protein (Fragment) OS=Homo sapiens GN=Igf2bp3 PE=2 SV=1 |
|  | Y-box-binding protein 2 OS=Homo sapiens GN=Ybx2 PE=1 SV=1 |
|  | Y-box-binding protein 3 OS=Homo sapiens GN=Ybx3 PE=1 SV=2 |
|  | Ybx2 protein OS=Homo sapiens GN=Ybx2 PE=2 SV=1 |
|  | Zinc finger CCCH domain-containing protein 15 OS=Homo sapiens GN=Zc3h15 PE=1 SV=2 |
