# Supplementary material for: CircRNF111 Protects Against Insulin Resistance and Lipid Deposition via Regulating miR-143-3p/IGF2R Axis in Metabolic Syndrome
Source: Front Cell Dev Biol. 2021 Aug 17;9:663148. doi: 10.3389/fcell.2021.663148 (PMC8415985; doi:10.3389/fcell.2021.663148)
Supplement: Supplementary file 1 [file Data_Sheet_1.ZIP › Supplemental File Sets/Supplementary Table 8.docx]

Supplementary Table 8. The miRNAs differentially expressing between MetS and non-MetS via microarray

| **miRNA ID** | **AVG ΔC_t_ (Ct(GOI) - Ave Ct (HKG))** | | **2^-ΔC_t_** | | **Fold Difference** | **T-TEST** | **Fold Up- or Down-**  **Regulation** |
| --- | --- | --- | --- | --- | --- | --- | --- |
|  | **A组** | **B组** | **A组** | **B组** | **A组 /B组** | **p value** | **A组 /B组** |
| hsa-miR-20a-3p | 6.94 | 9.01 | 8.2E-03 | 1.9E-03 | **4.19** | **0.0086** | **4.19** |
| hsa-miR-148a-3p | 5.29 | 7.01 | 2.6E-02 | 7.7E-03 | **3.30** | 0.0918 | **3.30** |
| hsa-miR-143-3p | 5.23 | 6.95 | 2.7E-02 | 8.1E-03 | **3.28** | **0.0034** | **3.28** |
| hsa-miR-365a-3p | 4.35 | 5.92 | 4.9E-02 | 1.7E-02 | **2.98** | **0.0042** | **2.98** |
| hsa-miR-22-5p | 6.64 | 8.18 | 1.0E-02 | 3.4E-03 | **2.91** | **0.0093** | **2.91** |
| hsa-miR-15b-5p | 1.81 | 3.27 | 2.8E-01 | 1.0E-01 | **2.75** | 0.1324 | **2.75** |
| hsa-let-7i-5p | 2.11 | 3.57 | 2.3E-01 | 8.4E-02 | **2.74** | **0.0206** | **2.74** |
| hsa-miR-29a-3p | 4.13 | 5.38 | 5.7E-02 | 2.4E-02 | **2.37** | **0.0286** | **2.37** |
| hsa-miR-182-5p | 9.22 | 10.47 | 1.7E-03 | 7.1E-04 | **2.36** | 0.2921 | **2.36** |
| hsa-miR-605 | 10.14 | 11.38 | 8.9E-04 | 3.8E-04 | **2.36** | 0.2121 | **2.36** |
| hsa-miR-424-5p | 9.95 | 11.16 | 1.0E-03 | 4.4E-04 | **2.32** | 0.1187 | **2.32** |
| hsa-miR-29b-2-5p | 7.70 | 8.89 | 4.8E-03 | 2.1E-03 | **2.28** | 0.1761 | **2.28** |
| hsa-miR-99a-5p | 4.65 | 5.82 | 4.0E-02 | 1.8E-02 | **2.26** | **0.0008** | **2.26** |
| hsa-miR-21-5p | -0.30 | 0.83 | 1.2E+00 | 5.6E-01 | **2.19** | 0.2059 | **2.19** |
| hsa-let-7f-5p | 2.41 | 3.50 | 1.9E-01 | 8.9E-02 | **2.12** | 0.1161 | **2.12** |
| hsa-miR-27a-3p | 8.64 | 9.66 | 2.5E-03 | 1.2E-03 | **2.04** | 0.2520 | **2.04** |
| hsa-miR-29a-5p | 7.63 | 8.56 | 5.1E-03 | 2.7E-03 | 1.90 | 0.3493 | 1.90 |
| hsa-miR-17-5p | 5.61 | 6.53 | 2.1E-02 | 1.1E-02 | 1.90 | 0.0648 | 1.90 |
| hsa-miR-20b-5p | 9.08 | 10.00 | 1.8E-03 | 9.7E-04 | 1.90 | 0.1203 | 1.90 |
| hsa-miR-338-3p | 6.54 | 7.43 | 1.1E-02 | 5.8E-03 | 1.85 | 0.1340 | 1.85 |
| hsa-miR-145-5p | 1.84 | 2.72 | 2.8E-01 | 1.5E-01 | 1.84 | 0.3668 | 1.84 |
| hsa-miR-22-3p | -0.41 | 0.45 | 1.3E+00 | 7.3E-01 | 1.82 | **0.0274** | 1.82 |
| hsa-miR-151a-3p | 7.30 | 8.16 | 6.3E-03 | 3.5E-03 | 1.81 | 0.5050 | 1.81 |
| hsa-miR-29c-3p | 3.69 | 4.54 | 7.7E-02 | 4.3E-02 | 1.79 | **0.0087** | 1.79 |
| hsa-miR-144-3p | 1.17 | 2.01 | 4.4E-01 | 2.5E-01 | 1.78 | **0.0218** | 1.78 |
| hsa-miR-34a-5p | 6.53 | 7.35 | 1.1E-02 | 6.1E-03 | 1.77 | 0.1836 | 1.77 |
| hsa-miR-194-5p | 4.15 | 4.97 | 5.6E-02 | 3.2E-02 | 1.76 | 0.0699 | 1.76 |
| hsa-miR-2110 | 6.15 | 6.96 | 1.4E-02 | 8.0E-03 | 1.76 | **0.0494** | 1.76 |
| hsa-miR-204-5p | 5.18 | 5.97 | 2.8E-02 | 1.6E-02 | 1.73 | 0.3343 | 1.73 |
| hsa-miR-190a | 9.93 | 10.67 | 1.0E-03 | 6.1E-04 | 1.68 | 0.4327 | 1.68 |
| hsa-miR-532-5p | 5.54 | 6.23 | 2.2E-02 | 1.3E-02 | 1.62 | 0.2456 | 1.62 |
| hsa-miR-320a | 5.04 | 5.72 | 3.0E-02 | 1.9E-02 | 1.61 | 0.1748 | 1.61 |
| hsa-miR-15a-5p | 1.14 | 1.82 | 4.5E-01 | 2.8E-01 | 1.60 | 0.0612 | 1.60 |
| hsa-miR-27b-3p | 1.62 | 2.28 | 3.3E-01 | 2.1E-01 | 1.58 | 0.1159 | 1.58 |
| hsa-miR-363-3p | 3.38 | 4.02 | 9.6E-02 | 6.2E-02 | 1.56 | 0.1662 | 1.56 |
| hsa-miR-885-5p | 5.02 | 5.65 | 3.1E-02 | 2.0E-02 | 1.55 | 0.4847 | 1.55 |
| hsa-miR-185-5p | 2.78 | 3.42 | 1.5E-01 | 9.4E-02 | 1.55 | 0.3106 | 1.55 |
| hsa-miR-30d-5p | 5.70 | 6.31 | 1.9E-02 | 1.3E-02 | 1.53 | 0.1396 | 1.53 |
| hsa-miR-335-5p | 7.62 | 8.17 | 5.1E-03 | 3.5E-03 | 1.46 | 0.4977 | 1.46 |
| hsa-let-7e-5p | 3.81 | 4.34 | 7.1E-02 | 4.9E-02 | 1.44 | 0.0997 | 1.44 |
| hsa-miR-152 | 3.74 | 4.27 | 7.5E-02 | 5.2E-02 | 1.44 | 0.1591 | 1.44 |
| hsa-miR-33a-5p | 7.14 | 7.67 | 7.1E-03 | 4.9E-03 | 1.44 | 0.3397 | 1.44 |
| hsa-miR-146b-5p | 7.85 | 8.36 | 4.3E-03 | 3.0E-03 | 1.42 | 0.3587 | 1.42 |
| hsa-miR-32-5p | 2.85 | 3.35 | 1.4E-01 | 9.8E-02 | 1.42 | 0.0897 | 1.42 |
| hsa-miR-125a-5p | 2.48 | 2.98 | 1.8E-01 | 1.3E-01 | 1.42 | 0.0715 | 1.42 |
| hsa-miR-125b-5p | 2.35 | 2.84 | 2.0E-01 | 1.4E-01 | 1.41 | 0.2880 | 1.41 |
| hsa-miR-629-5p | 6.18 | 6.67 | 1.4E-02 | 9.8E-03 | 1.41 | 0.2559 | 1.41 |
| hsa-miR-215 | 2.77 | 3.25 | 1.5E-01 | 1.1E-01 | 1.39 | 0.2013 | 1.39 |
| hsa-miR-584-5p | 6.92 | 7.40 | 8.3E-03 | 5.9E-03 | 1.39 | 0.3079 | 1.39 |
| hsa-miR-30a-5p | 5.56 | 6.03 | 2.1E-02 | 1.5E-02 | 1.39 | 0.2537 | 1.39 |
| hsa-miR-324-5p | 5.14 | 5.61 | 2.8E-02 | 2.0E-02 | 1.38 | 0.1809 | 1.38 |
| hsa-miR-30e-5p | 2.38 | 2.84 | 1.9E-01 | 1.4E-01 | 1.38 | 0.2674 | 1.38 |
| hsa-miR-18b-5p | 2.51 | 2.97 | 1.8E-01 | 1.3E-01 | 1.38 | 0.2198 | 1.38 |
| hsa-miR-16-2-3p | 5.92 | 6.36 | 1.7E-02 | 1.2E-02 | 1.36 | 0.3051 | 1.36 |
| hsa-miR-19b-3p | -1.58 | -1.17 | 3.0E+00 | 2.2E+00 | 1.33 | 0.3399 | 1.33 |
| hsa-miR-500a-5p | 8.06 | 8.47 | 3.7E-03 | 2.8E-03 | 1.33 | 0.6996 | 1.33 |
| hsa-miR-132-3p | 5.14 | 5.55 | 2.8E-02 | 2.1E-02 | 1.33 | 0.4365 | 1.33 |
| hsa-miR-320b | 3.10 | 3.51 | 1.2E-01 | 8.8E-02 | 1.32 | **0.0478** | 1.32 |
| hsa-let-7c | 5.20 | 5.59 | 2.7E-02 | 2.1E-02 | 1.31 | 0.3861 | 1.31 |
| hsa-miR-101-3p | 1.98 | 2.37 | 2.5E-01 | 1.9E-01 | 1.31 | **0.0028** | 1.31 |
| hsa-let-7g-5p | 0.05 | 0.43 | 9.7E-01 | 7.4E-01 | 1.31 | 0.1686 | 1.31 |
| hsa-miR-378a-3p | 2.94 | 3.33 | 1.3E-01 | 1.0E-01 | 1.31 | 0.2985 | 1.31 |
| hsa-miR-660-5p | 1.89 | 2.27 | 2.7E-01 | 2.1E-01 | 1.30 | 0.1738 | 1.30 |
| hsa-miR-195-5p | 10.66 | 11.02 | 6.2E-04 | 4.8E-04 | 1.29 | 0.4863 | 1.29 |
| hsa-miR-99b-5p | 4.40 | 4.76 | 4.7E-02 | 3.7E-02 | 1.28 | 0.2330 | 1.28 |
| hsa-miR-28-5p | 4.19 | 4.55 | 5.5E-02 | 4.3E-02 | 1.28 | 0.4741 | 1.28 |
| hsa-miR-146a-5p | 1.46 | 1.82 | 3.6E-01 | 2.8E-01 | 1.28 | 0.2613 | 1.28 |
| hsa-miR-497-5p | 5.65 | 5.99 | 2.0E-02 | 1.6E-02 | 1.27 | 0.3965 | 1.27 |
| hsa-miR-590-5p | 4.20 | 4.52 | 5.5E-02 | 4.3E-02 | 1.25 | 0.3414 | 1.25 |
| hsa-miR-551b-3p | 6.39 | 6.71 | 1.2E-02 | 9.5E-03 | 1.25 | 0.5144 | 1.25 |
| hsa-miR-142-3p | 0.15 | 0.46 | 9.0E-01 | 7.2E-01 | 1.25 | 0.2977 | 1.25 |
| hsa-miR-142-5p | 2.79 | 3.10 | 1.4E-01 | 1.2E-01 | 1.24 | 0.3478 | 1.24 |
| hsa-miR-425-3p | 6.78 | 7.07 | 9.1E-03 | 7.5E-03 | 1.22 | 0.6347 | 1.22 |
| UniSp3 IPC | -5.60 | -5.32 | 4.9E+01 | 4.0E+01 | 1.21 | 0.4675 | 1.21 |
| hsa-miR-15b-3p | 2.96 | 3.23 | 1.3E-01 | 1.1E-01 | 1.20 | 0.3038 | 1.20 |
| hsa-miR-505-3p | 5.14 | 5.40 | 2.8E-02 | 2.4E-02 | 1.20 | 0.4480 | 1.20 |
| hsa-miR-126-3p | -1.44 | -1.19 | 2.7E+00 | 2.3E+00 | 1.19 | 0.2034 | 1.19 |
| hsa-miR-103a-3p | 1.19 | 1.43 | 4.4E-01 | 3.7E-01 | 1.19 | 0.6676 | 1.19 |
| hsa-miR-26a-5p | 0.43 | 0.67 | 7.4E-01 | 6.3E-01 | 1.18 | 0.5656 | 1.18 |
| hsa-miR-10b-5p | 4.88 | 5.12 | 3.4E-02 | 2.9E-02 | 1.18 | 0.5999 | 1.18 |
| UniSp3 IPC | -5.13 | -4.89 | 3.5E+01 | 3.0E+01 | 1.18 | 0.4748 | 1.18 |
| hsa-miR-199a-3p | 2.24 | 2.47 | 2.1E-01 | 1.8E-01 | 1.17 | 0.6150 | 1.17 |
| hsa-miR-130a-3p | 2.64 | 2.84 | 1.6E-01 | 1.4E-01 | 1.15 | 0.4138 | 1.15 |
| hsa-miR-374a-5p | 6.42 | 6.62 | 1.2E-02 | 1.0E-02 | 1.15 | 0.6987 | 1.15 |
| hsa-miR-122-5p | 2.35 | 2.54 | 2.0E-01 | 1.7E-01 | 1.15 | 0.8271 | 1.15 |
| hsa-miR-23a-3p | -0.91 | -0.72 | 1.9E+00 | 1.6E+00 | 1.14 | 0.6517 | 1.14 |
| hsa-miR-107 | 3.17 | 3.36 | 1.1E-01 | 9.8E-02 | 1.14 | 0.7506 | 1.14 |
| hsa-miR-30e-3p | 7.16 | 7.33 | 7.0E-03 | 6.2E-03 | 1.13 | 0.7665 | 1.13 |
| UniSp3 IPC | -5.35 | -5.17 | 4.1E+01 | 3.6E+01 | 1.13 | 0.5900 | 1.13 |
| hsa-miR-192-5p | 2.55 | 2.72 | 1.7E-01 | 1.5E-01 | 1.12 | 0.5848 | 1.12 |
| hsa-miR-18a-3p | 9.04 | 9.21 | 1.9E-03 | 1.7E-03 | 1.12 | 0.7704 | 1.12 |
| hsa-miR-140-3p | 1.36 | 1.50 | 3.9E-01 | 3.5E-01 | 1.10 | 0.6731 | 1.10 |
| hsa-miR-451a | -5.70 | -5.58 | 5.2E+01 | 4.8E+01 | 1.09 | 0.6216 | 1.09 |
| hsa-miR-208a | 11.25 | 11.38 | 4.1E-04 | 3.8E-04 | 1.09 | 0.7577 | 1.09 |
| hsa-miR-186-5p | 2.80 | 2.92 | 1.4E-01 | 1.3E-01 | 1.09 | 0.2967 | 1.09 |
| hsa-miR-148b-3p | 1.57 | 1.68 | 3.4E-01 | 3.1E-01 | 1.08 | 0.5965 | 1.08 |
| hsa-miR-423-5p | 0.91 | 1.01 | 5.3E-01 | 5.0E-01 | 1.07 | 0.3376 | 1.07 |
| hsa-miR-222-3p | 1.20 | 1.29 | 4.4E-01 | 4.1E-01 | 1.07 | 0.6597 | 1.07 |
| hsa-miR-140-5p | 3.93 | 4.02 | 6.5E-02 | 6.2E-02 | 1.06 | 0.7846 | 1.06 |
| UniSp3 IPC | -5.86 | -5.78 | 5.8E+01 | 5.5E+01 | 1.06 | 0.8251 | 1.06 |
| hsa-let-7a-5p | -1.38 | -1.30 | 2.6E+00 | 2.5E+00 | 1.06 | 0.7202 | 1.06 |
| hsa-miR-19a-3p | -1.15 | -1.07 | 2.2E+00 | 2.1E+00 | 1.05 | 0.7323 | 1.05 |
| hsa-miR-10a-5p | 11.15 | 11.22 | 4.4E-04 | 4.2E-04 | 1.05 | 0.8268 | 1.05 |
| hsa-miR-150-5p | 0.37 | 0.44 | 7.7E-01 | 7.4E-01 | 1.05 | 0.8576 | 1.05 |
| hsa-miR-205-5p | 9.95 | 10.02 | 1.0E-03 | 9.6E-04 | 1.04 | 0.9246 | 1.04 |
| hsa-miR-301a-3p | 2.77 | 2.83 | 1.5E-01 | 1.4E-01 | 1.04 | 0.8152 | 1.04 |
| hsa-miR-193b-3p | 4.60 | 4.65 | 4.1E-02 | 4.0E-02 | 1.03 | 0.9152 | 1.03 |
| hsa-miR-199a-5p | 2.65 | 2.69 | 1.6E-01 | 1.6E-01 | 1.03 | 0.9476 | 1.03 |
| UniSp3 IPC | -5.64 | -5.61 | 5.0E+01 | 4.9E+01 | 1.02 | 0.9383 | 1.02 |
| UniSp3 IPC | -5.32 | -5.30 | 4.0E+01 | 3.9E+01 | 1.02 | 0.9412 | 1.02 |
| hsa-miR-95 | 10.59 | 10.61 | 6.5E-04 | 6.4E-04 | 1.02 | 0.9787 | 1.02 |
| hsa-miR-29b-3p | 6.61 | 6.63 | 1.0E-02 | 1.0E-02 | 1.01 | 0.8975 | 1.01 |
| UniSp6 | 0.58 | 0.59 | 6.7E-01 | 6.6E-01 | 1.01 | 0.9782 | 1.01 |
| hsa-miR-16-5p | -5.97 | -5.97 | 6.3E+01 | 6.3E+01 | 1.00 | 0.9797 | 1.00 |
| hsa-miR-144-5p | 1.76 | 1.76 | 2.9E-01 | 3.0E-01 | 1.00 | 0.9992 | -1.00 |
| hsa-miR-18a-5p | 2.55 | 2.54 | 1.7E-01 | 1.7E-01 | 1.00 | 0.9960 | -1.00 |
| hsa-miR-106b-5p | 1.21 | 1.20 | 4.3E-01 | 4.4E-01 | 0.99 | 0.9711 | -1.01 |
| hsa-miR-361-3p | 11.36 | 11.35 | 3.8E-04 | 3.8E-04 | 0.99 | 0.9609 | -1.01 |
| hsa-miR-30b-5p | 1.04 | 1.02 | 4.8E-01 | 4.9E-01 | 0.98 | 0.9488 | -1.02 |
| hsa-miR-155-5p | 6.11 | 6.07 | 1.4E-02 | 1.5E-02 | 0.97 | 0.9052 | -1.03 |
| hsa-miR-20a-5p | -1.47 | -1.52 | 2.8E+00 | 2.9E+00 | 0.97 | 0.7670 | -1.03 |
| hsa-miR-652-3p | 0.96 | 0.91 | 5.1E-01 | 5.3E-01 | 0.96 | 0.8352 | -1.04 |
| hsa-let-7i-3p | 7.37 | 7.32 | 6.0E-03 | 6.3E-03 | 0.96 | 0.8593 | -1.04 |
| hsa-miR-92a-3p | -2.68 | -2.74 | 6.4E+00 | 6.7E+00 | 0.96 | 0.8347 | -1.04 |
| hsa-miR-24-3p | -1.07 | -1.15 | 2.1E+00 | 2.2E+00 | 0.95 | 0.7728 | -1.05 |
| hsa-miR-30c-5p | 0.45 | 0.38 | 7.3E-01 | 7.7E-01 | 0.95 | 0.7600 | -1.05 |
| hsa-miR-486-5p | -2.25 | -2.34 | 4.8E+00 | 5.1E+00 | 0.94 | 0.7922 | -1.06 |
| UniSp2 | 11.47 | 11.38 | 3.5E-04 | 3.8E-04 | 0.94 | 0.8070 | -1.07 |
| UniSp4 | 11.47 | 11.38 | 3.5E-04 | 3.8E-04 | 0.94 | 0.8070 | -1.07 |
| UniSp5 | 11.47 | 11.38 | 3.5E-04 | 3.8E-04 | 0.94 | 0.8070 | -1.07 |
| cel-miR-39-3p | 11.47 | 11.38 | 3.5E-04 | 3.8E-04 | 0.94 | 0.8070 | -1.07 |
| Blank (H2O) | 11.47 | 11.38 | 3.5E-04 | 3.8E-04 | 0.94 | 0.8070 | -1.07 |
| Blank (H2O) | 11.47 | 11.38 | 3.5E-04 | 3.8E-04 | 0.94 | 0.8070 | -1.07 |
| hsa-miR-296-5p | 6.58 | 6.48 | 1.0E-02 | 1.1E-02 | 0.94 | 0.7649 | -1.07 |
| hsa-miR-93-5p | -0.91 | -1.01 | 1.9E+00 | 2.0E+00 | 0.93 | 0.3376 | -1.07 |
| hsa-miR-223-5p | 6.76 | 6.66 | 9.2E-03 | 9.9E-03 | 0.93 | 0.8896 | -1.07 |
| hsa-miR-130b-3p | 6.24 | 6.14 | 1.3E-02 | 1.4E-02 | 0.93 | 0.7898 | -1.07 |
| hsa-miR-128 | 3.49 | 3.39 | 8.9E-02 | 9.5E-02 | 0.93 | 0.7081 | -1.07 |
| hsa-miR-25-3p | -1.26 | -1.36 | 2.4E+00 | 2.6E+00 | 0.93 | 0.4338 | -1.08 |
| hsa-miR-574-3p | 3.84 | 3.70 | 7.0E-02 | 7.7E-02 | 0.91 | 0.6382 | -1.10 |
| hsa-miR-181a-5p | 1.82 | 1.67 | 2.8E-01 | 3.1E-01 | 0.90 | 0.5724 | -1.11 |
| hsa-miR-532-3p | 4.54 | 4.39 | 4.3E-02 | 4.8E-02 | 0.90 | 0.5337 | -1.11 |
| hsa-miR-26b-5p | 1.46 | 1.30 | 3.6E-01 | 4.1E-01 | 0.90 | 0.4524 | -1.12 |
| hsa-miR-331-3p | 7.47 | 7.30 | 5.6E-03 | 6.3E-03 | 0.89 | 0.5889 | -1.12 |
| hsa-miR-139-5p | 3.05 | 2.88 | 1.2E-01 | 1.4E-01 | 0.89 | 0.2471 | -1.12 |
| hsa-miR-106a-5p | -1.51 | -1.69 | 2.9E+00 | 3.2E+00 | 0.88 | 0.2657 | -1.13 |
| hsa-miR-191-5p | 1.44 | 1.26 | 3.7E-01 | 4.2E-01 | 0.88 | 0.6116 | -1.13 |
| hsa-miR-484 | 0.69 | 0.51 | 6.2E-01 | 7.0E-01 | 0.88 | 0.2004 | -1.13 |
| hsa-miR-374b-5p | 3.47 | 3.29 | 9.0E-02 | 1.0E-01 | 0.88 | 0.6103 | -1.13 |
| hsa-miR-326 | 4.64 | 4.46 | 4.0E-02 | 4.6E-02 | 0.88 | 0.6780 | -1.14 |
| hsa-miR-421 | 8.14 | 7.95 | 3.5E-03 | 4.0E-03 | 0.88 | 0.7933 | -1.14 |
| hsa-miR-210 | 4.10 | 3.91 | 5.8E-02 | 6.7E-02 | 0.87 | 0.5082 | -1.15 |
| hsa-let-7b-5p | 1.19 | 0.98 | 4.4E-01 | 5.1E-01 | 0.87 | 0.5853 | -1.15 |
| hsa-miR-221-3p | -0.35 | -0.59 | 1.3E+00 | 1.5E+00 | 0.85 | 0.5154 | -1.18 |
| hsa-miR-425-5p | 0.60 | 0.36 | 6.6E-01 | 7.8E-01 | 0.85 | 0.1378 | -1.18 |
| hsa-let-7d-3p | 0.52 | 0.26 | 7.0E-01 | 8.3E-01 | 0.84 | 0.3033 | -1.19 |
| hsa-miR-106b-3p | 6.13 | 5.87 | 1.4E-02 | 1.7E-02 | 0.84 | 0.2387 | -1.20 |
| hsa-miR-342-3p | 2.53 | 2.27 | 1.7E-01 | 2.1E-01 | 0.84 | 0.3787 | -1.20 |
| hsa-miR-23b-3p | 0.82 | 0.54 | 5.7E-01 | 6.9E-01 | 0.82 | 0.3776 | -1.22 |
| hsa-miR-502-3p | 5.52 | 5.24 | 2.2E-02 | 2.6E-02 | 0.82 | 0.4484 | -1.22 |
| hsa-miR-92b-3p | 10.68 | 10.39 | 6.1E-04 | 7.4E-04 | 0.82 | 0.4327 | -1.22 |
| hsa-miR-197-3p | 2.98 | 2.67 | 1.3E-01 | 1.6E-01 | 0.80 | 0.4187 | -1.24 |
| hsa-let-7d-5p | 1.97 | 1.65 | 2.6E-01 | 3.2E-01 | 0.80 | 0.3561 | -1.25 |
| hsa-miR-28-3p | 3.75 | 3.41 | 7.5E-02 | 9.4E-02 | 0.79 | 0.1767 | -1.26 |
| hsa-miR-151a-5p | 0.54 | 0.15 | 6.9E-01 | 9.0E-01 | 0.76 | 0.1746 | -1.31 |
| hsa-miR-339-3p | 5.39 | 4.97 | 2.4E-02 | 3.2E-02 | 0.75 | 0.3057 | -1.34 |
| hsa-miR-133b | 6.43 | 5.98 | 1.2E-02 | 1.6E-02 | 0.73 | 0.4982 | -1.37 |
| hsa-miR-223-3p | -2.69 | -3.15 | 6.4E+00 | 8.9E+00 | 0.72 | 0.1943 | -1.38 |
| hsa-miR-324-3p | 2.66 | 2.19 | 1.6E-01 | 2.2E-01 | 0.72 | 0.0553 | -1.39 |
| hsa-miR-423-3p | 2.13 | 1.65 | 2.3E-01 | 3.2E-01 | 0.72 | 0.1178 | -1.39 |
| hsa-let-7b-3p | 5.36 | 4.81 | 2.4E-02 | 3.6E-02 | 0.69 | 0.0572 | -1.46 |
| hsa-miR-200c-3p | 7.66 | 7.11 | 4.9E-03 | 7.3E-03 | 0.68 | 0.7130 | -1.47 |
| hsa-miR-766-3p | 3.39 | 2.80 | 9.6E-02 | 1.4E-01 | 0.67 | 0.3111 | -1.50 |
| hsa-miR-328 | 4.11 | 3.42 | 5.8E-02 | 9.3E-02 | 0.62 | 0.0593 | -1.61 |
| hsa-miR-1 | 9.43 | 8.73 | 1.4E-03 | 2.4E-03 | 0.61 | 0.4474 | -1.63 |
| hsa-miR-93-3p | 4.89 | 4.17 | 3.4E-02 | 5.5E-02 | 0.61 | **0.0006** | -1.64 |
| hsa-miR-376a-3p | 10.95 | 10.16 | 5.1E-04 | 8.7E-04 | 0.58 | 0.0614 | -1.73 |
| hsa-miR-501-3p | 7.51 | 6.71 | 5.5E-03 | 9.6E-03 | 0.57 | 0.4418 | -1.75 |
| hsa-miR-339-5p | 4.64 | 3.74 | 4.0E-02 | 7.5E-02 | 0.54 | **0.0427** | -1.87 |
| hsa-miR-409-3p | 5.79 | 4.86 | 1.8E-02 | 3.5E-02 | 0.52 | 0.1761 | -1.91 |
| hsa-miR-141-3p | 8.12 | 7.16 | 3.6E-03 | 7.0E-03 | 0.52 | 0.2212 | -1.94 |
| hsa-miR-133a | 6.69 | 5.62 | 9.7E-03 | 2.0E-02 | **0.48** | 0.1396 | **-2.10** |
| hsa-miR-543 | 7.83 | 6.62 | 4.4E-03 | 1.0E-02 | **0.43** | 0.2857 | **-2.31** |
| hsa-miR-136-5p | 6.11 | 4.88 | 1.5E-02 | 3.4E-02 | **0.43** | 0.1052 | **-2.34** |
| hsa-miR-200a-3p | 10.55 | 9.19 | 6.7E-04 | 1.7E-03 | **0.39** | 0.1114 | **-2.56** |
| hsa-miR-375 | 6.29 | 4.77 | 1.3E-02 | 3.7E-02 | **0.35** | **0.0390** | **-2.86** |
| hsa-miR-154-5p | 6.84 | 5.29 | 8.7E-03 | 2.6E-02 | **0.34** | **0.0100** | **-2.93** |
| hsa-miR-127-3p | 7.05 | 5.20 | 7.5E-03 | 2.7E-02 | **0.28** | 0.0622 | **-3.60** |
| hsa-miR-495-3p | 8.26 | 6.31 | 3.3E-03 | 1.3E-02 | **0.26** | 0.1476 | **-3.84** |
| hsa-miR-485-3p | 8.15 | 6.08 | 3.5E-03 | 1.5E-02 | **0.24** | 0.0709 | **-4.20** |
| hsa-miR-346 | 10.82 | 8.31 | 5.5E-04 | 3.2E-03 | **0.18** | **0.0113** | **-5.71** |
| hsa-miR-382-5p | 7.89 | 4.82 | 4.2E-03 | 3.5E-02 | **0.12** | **0.0318** | **-8.43** |
