# Supplementary material for: CircRNF111 Protects Against Insulin Resistance and Lipid Deposition via Regulating miR-143-3p/IGF2R Axis in Metabolic Syndrome
Source: Front Cell Dev Biol. 2021 Aug 17;9:663148. doi: 10.3389/fcell.2021.663148 (PMC8415985; doi:10.3389/fcell.2021.663148)
Supplement: Supplementary file 1 [file Data_Sheet_1.ZIP › Supplemental File Sets/Supplementary Table 9.docx]

Supplementary Table 9. Overlapping of miRanda, circBank, TargetScan and microarray

| **miRanda** | **circBank** | **Targetscan** | **microarray** |
| --- | --- | --- | --- |
| hsa-miR-1 | hsa-miR-1252-5p | hsa-let-7a-3p | hsa-let-7a-5p |
| hsa-miR-101 | hsa-miR-1253 | hsa-let-7b-3p | hsa-let-7b-3p |
| hsa-miR-1297 | hsa-miR-1273h-3p | hsa-let-7c-3p | hsa-let-7b-5p |
| hsa-miR-139-5p | hsa-miR-1285-5p | hsa-let-7f-1-3p | hsa-let-7c |
| hsa-miR-143 | hsa-miR-135a-5p | hsa-let-7f-2-3p | hsa-let-7d-3p |
| hsa-miR-154 | hsa-miR-135b-5p | hsa-let-7f-2-3p | hsa-let-7d-5p |
| hsa-miR-15a | hsa-miR-143-3p | hsa-miR-101-3p.1 | hsa-let-7e-5p |
| hsa-miR-15b | hsa-miR-15b-3p | hsa-miR-101-3p.2 | hsa-let-7f-5p |
| hsa-miR-16 | hsa-miR-200c-5p | hsa-miR-103a-3p | hsa-let-7g-5p |
| hsa-miR-195 | hsa-miR-204-3p | hsa-miR-105-5p | hsa-let-7i-3p |
| hsa-miR-196a | hsa-miR-2115-3p | hsa-miR-106a-3p | hsa-let-7i-5p |
| hsa-miR-196b | hsa-miR-211-5p | hsa-miR-106a-3p | hsa-miR-1 |
| hsa-miR-19a | hsa-miR-27a-3p | hsa-miR-106a-3p | hsa-miR-101-3p |
| hsa-miR-19b | hsa-miR-27b-3p | hsa-miR-107 | hsa-miR-103a-3p |
| hsa-miR-203 | hsa-miR-298 | hsa-miR-10a-3p | hsa-miR-106a-5p |
| hsa-miR-206 | hsa-miR-302b-5p | hsa-miR-1178-5p | hsa-miR-106b-3p |
| hsa-miR-21 | hsa-miR-302d-5p | hsa-miR-1179 | hsa-miR-106b-5p |
| hsa-miR-218 | hsa-miR-3140-3p | hsa-miR-1182 | hsa-miR-107 |
| hsa-miR-23a | hsa-miR-3152-5p | hsa-miR-1183 | hsa-miR-10a-5p |
| hsa-miR-23b | hsa-miR-3173-3p | hsa-miR-1184 | hsa-miR-10b-5p |
| hsa-miR-26a | hsa-miR-324-3p | hsa-miR-1185-1-3p | hsa-miR-122-5p |
| hsa-miR-26b | hsa-miR-335-3p | hsa-miR-1185-1-3p | hsa-miR-125a-5p |
| hsa-miR-27a | hsa-miR-34b-3p | hsa-miR-1185-2-3p | hsa-miR-125b-5p |
| hsa-miR-27b | hsa-miR-361-5p | hsa-miR-1185-2-3p | hsa-miR-126-3p |
| hsa-miR-300 | hsa-miR-3916 | hsa-miR-1193 | hsa-miR-127-3p |
| hsa-miR-30a | hsa-miR-4428 | hsa-miR-1205 | hsa-miR-128 |
| hsa-miR-30b | hsa-miR-4434 | hsa-miR-1206 | hsa-miR-130a-3p |
| hsa-miR-30c | hsa-miR-4461 | hsa-miR-1206 | hsa-miR-130b-3p |
| hsa-miR-30d | hsa-miR-4495 | hsa-miR-1207-3p | hsa-miR-132-3p |
| hsa-miR-30e | hsa-miR-4659a-5p | hsa-miR-1226-3p | hsa-miR-133a |
| hsa-miR-31 | hsa-miR-4659b-5p | hsa-miR-1233-5p | hsa-miR-133b |
| hsa-miR-320a | hsa-miR-4674 | hsa-miR-1236-5p | hsa-miR-136-5p |
| hsa-miR-320b | hsa-miR-4677-5p | hsa-miR-1238-5p | hsa-miR-139-5p |
| hsa-miR-320c | hsa-miR-4698 | hsa-miR-1243 | hsa-miR-140-3p |
| hsa-miR-320d | hsa-miR-4708-3p | hsa-miR-1245a | hsa-miR-140-5p |
| hsa-miR-324-5p | hsa-miR-4743-3p | hsa-miR-1245a | hsa-miR-141-3p |
| hsa-miR-335 | hsa-miR-4753-3p | hsa-miR-124-5p | hsa-miR-142-3p |
| hsa-miR-340 | hsa-miR-4760-3p | hsa-miR-1250-3p | hsa-miR-142-5p |
| hsa-miR-381 | hsa-miR-4778-3p | hsa-miR-1250-3p | hsa-miR-143-3p |
| hsa-miR-410 | hsa-miR-4782-5p | hsa-miR-1250-3p | hsa-miR-144-3p |
| hsa-miR-421 | hsa-miR-495-3p | hsa-miR-1251-3p | hsa-miR-144-5p |
| hsa-miR-424 | hsa-miR-5011-3p | hsa-miR-1252-3p | hsa-miR-145-5p |
| hsa-miR-455-5p | hsa-miR-511-5p | hsa-miR-1255b-2-3p | hsa-miR-146a-5p |
| hsa-miR-486-5p | hsa-miR-515-5p | hsa-miR-125b-2-3p | hsa-miR-146b-5p |
| hsa-miR-488 | hsa-miR-518a-5p | hsa-miR-1261 | hsa-miR-148a-3p |
| hsa-miR-494 | hsa-miR-518c-5p | hsa-miR-1263 | hsa-miR-148b-3p |
| hsa-miR-495 | hsa-miR-519d-5p | hsa-miR-1271-3p | hsa-miR-150-5p |
| hsa-miR-496 | hsa-miR-519e-5p | hsa-miR-1271-5p | hsa-miR-151a-3p |
| hsa-miR-497 | hsa-miR-527 | hsa-miR-1275 | hsa-miR-151a-5p |
| hsa-miR-503 | hsa-miR-548aw | hsa-miR-127-5p | hsa-miR-152 |
| hsa-miR-505 | hsa-miR-5582-3p | hsa-miR-1283 | hsa-miR-154-5p |
| hsa-miR-539 | hsa-miR-580-3p | hsa-miR-128-3p | hsa-miR-155-5p |
| hsa-miR-590-3p | hsa-miR-583 | hsa-miR-1292-5p | hsa-miR-15a-5p |
| hsa-miR-590-5p | hsa-miR-606 | hsa-miR-1292-5p | hsa-miR-15b-3p |
| hsa-miR-613 | hsa-miR-6074 | hsa-miR-1297 | hsa-miR-15b-5p |
| hsa-miR-653 | hsa-miR-6505-3p | hsa-miR-1298-3p | hsa-miR-16-2-3p |
| hsa-miR-7 | hsa-miR-6515-3p | hsa-miR-1298-3p | hsa-miR-16-5p |
| hsa-miR-9 | hsa-miR-653-3p | hsa-miR-1303 | hsa-miR-17-5p |
|  | hsa-miR-6750-5p | hsa-miR-1305 | hsa-miR-181a-5p |
|  | hsa-miR-6764-3p | hsa-miR-1305 | hsa-miR-182-5p |
|  | hsa-miR-6780a-3p | hsa-miR-130a-5p | hsa-miR-185-5p |
|  | hsa-miR-6817-5p | hsa-miR-130a-5p | hsa-miR-186-5p |
|  | hsa-miR-6822-5p | hsa-miR-1321 | hsa-miR-18a-3p |
|  | hsa-miR-6834-5p | hsa-miR-1324 | hsa-miR-18a-5p |
|  | hsa-miR-6852-3p | hsa-miR-1343-3p | hsa-miR-18b-5p |
|  | hsa-miR-6859-5p | hsa-miR-134-5p | hsa-miR-190a |
|  | hsa-miR-6866-3p | hsa-miR-138-1-3p | hsa-miR-191-5p |
|  | hsa-miR-6868-3p | hsa-miR-139-5p | hsa-miR-192-5p |
|  | hsa-miR-6868-5p | hsa-miR-1-3p | hsa-miR-193b-3p |
|  | hsa-miR-6893-3p | hsa-miR-142-3p.2 | hsa-miR-194-5p |
|  | hsa-miR-7111-3p | hsa-miR-143-3p | hsa-miR-195-5p |
|  | hsa-miR-7-1-3p | hsa-miR-144-3p | hsa-miR-197-3p |
|  | hsa-miR-7160-5p | hsa-miR-144-3p | hsa-miR-199a-3p |
|  | hsa-miR-7-2-3p | hsa-miR-144-5p | hsa-miR-199a-5p |
|  | hsa-miR-744-3p | hsa-miR-145-5p | hsa-miR-19a-3p |
|  | hsa-miR-760 | hsa-miR-1468-3p | hsa-miR-19b-3p |
|  | hsa-miR-876-3p | hsa-miR-148a-5p | hsa-miR-200a-3p |
|  | hsa-miR-93-3p | hsa-miR-148a-5p | hsa-miR-200c-3p |
|  | hsa-miR-942-5p | hsa-miR-149-3p | hsa-miR-204-5p |
|  |  | hsa-miR-150-3p | hsa-miR-205-5p |
|  |  | hsa-miR-153-5p | hsa-miR-208a |
|  |  | hsa-miR-153-5p | hsa-miR-20a-3p |
|  |  | hsa-miR-153-5p | hsa-miR-20a-5p |
|  |  | hsa-miR-153-5p | hsa-miR-20b-5p |
|  |  | hsa-miR-155-5p | hsa-miR-210 |
|  |  | hsa-miR-15a-5p | hsa-miR-2110 |
|  |  | hsa-miR-15a-5p | hsa-miR-215 |
|  |  | hsa-miR-15b-3p | hsa-miR-21-5p |
|  |  | hsa-miR-15b-5p | hsa-miR-221-3p |
|  |  | hsa-miR-15b-5p | hsa-miR-222-3p |
|  |  | hsa-miR-16-1-3p | hsa-miR-223-3p |
|  |  | hsa-miR-16-2-3p | hsa-miR-223-5p |
|  |  | hsa-miR-16-2-3p | hsa-miR-22-3p |
|  |  | hsa-miR-16-5p | hsa-miR-22-5p |
|  |  | hsa-miR-16-5p | hsa-miR-23a-3p |
|  |  | hsa-miR-17-3p | hsa-miR-23b-3p |
|  |  | hsa-miR-17-3p | hsa-miR-24-3p |
|  |  | hsa-miR-181a-2-3p | hsa-miR-25-3p |
|  |  | hsa-miR-181a-3p | hsa-miR-26a-5p |
|  |  | hsa-miR-181b-2-3p | hsa-miR-26b-5p |
|  |  | hsa-miR-181b-3p | hsa-miR-27a-3p |
|  |  | hsa-miR-1825 | hsa-miR-27b-3p |
|  |  | hsa-miR-183-3p | hsa-miR-28-3p |
|  |  | hsa-miR-183-5p.2 | hsa-miR-28-5p |
|  |  | hsa-miR-186-5p | hsa-miR-296-5p |
|  |  | hsa-miR-188-5p | hsa-miR-29a-3p |
|  |  | hsa-miR-1910-5p | hsa-miR-29a-5p |
|  |  | hsa-miR-1911-3p | hsa-miR-29b-2-5p |
|  |  | hsa-miR-191-3p | hsa-miR-29b-3p |
|  |  | hsa-miR-1914-5p | hsa-miR-29c-3p |
|  |  | hsa-miR-1915-5p | hsa-miR-301a-3p |
|  |  | hsa-miR-194-5p | hsa-miR-30a-5p |
|  |  | hsa-miR-195-3p | hsa-miR-30b-5p |
|  |  | hsa-miR-195-3p | hsa-miR-30c-5p |
|  |  | hsa-miR-195-5p | hsa-miR-30d-5p |
|  |  | hsa-miR-195-5p | hsa-miR-30e-3p |
|  |  | hsa-miR-196a-5p | hsa-miR-30e-5p |
|  |  | hsa-miR-196b-5p | hsa-miR-320a |
|  |  | hsa-miR-199a-3p | hsa-miR-320b |
|  |  | hsa-miR-199a-5p | hsa-miR-324-3p |
|  |  | hsa-miR-199b-3p | hsa-miR-324-5p |
|  |  | hsa-miR-199b-5p | hsa-miR-32-5p |
|  |  | hsa-miR-19a-3p | hsa-miR-326 |
|  |  | hsa-miR-19b-3p | hsa-miR-328 |
|  |  | hsa-miR-200a-5p | hsa-miR-331-3p |
|  |  | hsa-miR-200a-5p | hsa-miR-335-5p |
|  |  | hsa-miR-200b-5p | hsa-miR-338-3p |
|  |  | hsa-miR-200b-5p | hsa-miR-339-3p |
|  |  | hsa-miR-200c-5p | hsa-miR-339-5p |
|  |  | hsa-miR-203a-3p.1 | hsa-miR-33a-5p |
|  |  | hsa-miR-203a-3p.1 | hsa-miR-342-3p |
|  |  | hsa-miR-2053 | hsa-miR-346 |
|  |  | hsa-miR-205-5p | hsa-miR-34a-5p |
|  |  | hsa-miR-206 | hsa-miR-361-3p |
|  |  | hsa-miR-208a-5p | hsa-miR-363-3p |
|  |  | hsa-miR-208b-5p | hsa-miR-365a-3p |
|  |  | hsa-miR-2113 | hsa-miR-374a-5p |
|  |  | hsa-miR-211-3p | hsa-miR-374b-5p |
|  |  | hsa-miR-2114-5p | hsa-miR-375 |
|  |  | hsa-miR-2115-3p | hsa-miR-376a-3p |
|  |  | hsa-miR-2115-5p | hsa-miR-378a-3p |
|  |  | hsa-miR-212-5p | hsa-miR-382-5p |
|  |  | hsa-miR-214-3p | hsa-miR-409-3p |
|  |  | hsa-miR-21-5p | hsa-miR-421 |
|  |  | hsa-miR-21-5p | hsa-miR-423-3p |
|  |  | hsa-miR-216a-3p | hsa-miR-423-5p |
|  |  | hsa-miR-218-2-3p | hsa-miR-424-5p |
|  |  | hsa-miR-218-5p | hsa-miR-425-3p |
|  |  | hsa-miR-222-5p | hsa-miR-425-5p |
|  |  | hsa-miR-224-5p | hsa-miR-451a |
|  |  | hsa-miR-224-5p | hsa-miR-484 |
|  |  | hsa-miR-2276-3p | hsa-miR-485-3p |
|  |  | hsa-miR-2276-3p | hsa-miR-486-5p |
|  |  | hsa-miR-2355-3p | hsa-miR-495-3p |
|  |  | hsa-miR-2355-5p | hsa-miR-497-5p |
|  |  | hsa-miR-2392 | hsa-miR-500a-5p |
|  |  | hsa-miR-2392 | hsa-miR-501-3p |
|  |  | hsa-miR-23a-3p | hsa-miR-502-3p |
|  |  | hsa-miR-23a-3p | hsa-miR-505-3p |
|  |  | hsa-miR-23b-3p | hsa-miR-532-3p |
|  |  | hsa-miR-23b-3p | hsa-miR-532-5p |
|  |  | hsa-miR-23c | hsa-miR-543 |
|  |  | hsa-miR-23c | hsa-miR-551b-3p |
|  |  | hsa-miR-24-1-5p | hsa-miR-574-3p |
|  |  | hsa-miR-24-2-5p | hsa-miR-584-5p |
|  |  | hsa-miR-26a-5p | hsa-miR-590-5p |
|  |  | hsa-miR-26b-3p | hsa-miR-605 |
|  |  | hsa-miR-26b-5p | hsa-miR-629-5p |
|  |  | hsa-miR-27a-3p | hsa-miR-652-3p |
|  |  | hsa-miR-27a-3p | hsa-miR-660-5p |
|  |  | hsa-miR-27b-3p | hsa-miR-766-3p |
|  |  | hsa-miR-27b-3p | hsa-miR-885-5p |
|  |  | hsa-miR-2909 | hsa-miR-92a-3p |
|  |  | hsa-miR-298 | hsa-miR-92b-3p |
|  |  | hsa-miR-29a-5p | hsa-miR-93-3p |
|  |  | hsa-miR-300 | hsa-miR-93-5p |
|  |  | hsa-miR-301a-5p | hsa-miR-95 |
|  |  | hsa-miR-301a-5p | hsa-miR-99a-5p |
|  |  | hsa-miR-302b-5p | hsa-miR-99b-5p |
|  |  | hsa-miR-302c-5p |  |
|  |  | hsa-miR-302d-5p |  |
|  |  | hsa-miR-3065-5p |  |
|  |  | hsa-miR-30a-5p |  |
|  |  | hsa-miR-30b-5p |  |
|  |  | hsa-miR-30c-5p |  |
|  |  | hsa-miR-30d-5p |  |
|  |  | hsa-miR-30e-5p |  |
|  |  | hsa-miR-3115 |  |
|  |  | hsa-miR-3118 |  |
|  |  | hsa-miR-3120-3p |  |
|  |  | hsa-miR-3121-3p |  |
|  |  | hsa-miR-3123 |  |
|  |  | hsa-miR-3125 |  |
|  |  | hsa-miR-3126-3p |  |
|  |  | hsa-miR-3129-3p |  |
|  |  | hsa-miR-3129-3p |  |
|  |  | hsa-miR-3129-3p |  |
|  |  | hsa-miR-3129-5p |  |
|  |  | hsa-miR-3130-3p |  |
|  |  | hsa-miR-3130-3p |  |
|  |  | hsa-miR-3136-5p |  |
|  |  | hsa-miR-3140-5p |  |
|  |  | hsa-miR-3143 |  |
|  |  | hsa-miR-3143 |  |
|  |  | hsa-miR-3145-3p |  |
|  |  | hsa-miR-3148 |  |
|  |  | hsa-miR-3150a-5p |  |
|  |  | hsa-miR-3150b-5p |  |
|  |  | hsa-miR-3152-5p |  |
|  |  | hsa-miR-3158-5p |  |
|  |  | hsa-miR-31-5p |  |
|  |  | hsa-miR-3161 |  |
|  |  | hsa-miR-3163 |  |
|  |  | hsa-miR-3163 |  |
|  |  | hsa-miR-3163 |  |
|  |  | hsa-miR-3163 |  |
|  |  | hsa-miR-3164 |  |
|  |  | hsa-miR-3164 |  |
|  |  | hsa-miR-3166 |  |
|  |  | hsa-miR-3168 |  |
|  |  | hsa-miR-3171 |  |
|  |  | hsa-miR-3171 |  |
|  |  | hsa-miR-3185 |  |
|  |  | hsa-miR-3200-3p |  |
|  |  | hsa-miR-3201 |  |
|  |  | hsa-miR-3201 |  |
|  |  | hsa-miR-3201 |  |
|  |  | hsa-miR-323a-3p |  |
|  |  | hsa-miR-323a-3p |  |
|  |  | hsa-miR-324-5p |  |
|  |  | hsa-miR-330-3p |  |
|  |  | hsa-miR-330-3p |  |
|  |  | hsa-miR-331-3p |  |
|  |  | hsa-miR-335-3p |  |
|  |  | hsa-miR-335-5p |  |
|  |  | hsa-miR-337-3p |  |
|  |  | hsa-miR-338-5p |  |
|  |  | hsa-miR-33a-3p |  |
|  |  | hsa-miR-340-5p |  |
|  |  | hsa-miR-340-5p |  |
|  |  | hsa-miR-340-5p |  |
|  |  | hsa-miR-345-5p |  |
|  |  | hsa-miR-34a-5p |  |
|  |  | hsa-miR-34c-3p |  |
|  |  | hsa-miR-34c-3p |  |
|  |  | hsa-miR-34c-5p |  |
|  |  | hsa-miR-3529-3p |  |
|  |  | hsa-miR-3529-3p |  |
|  |  | hsa-miR-3591-5p |  |
|  |  | hsa-miR-3606-3p |  |
|  |  | hsa-miR-3609 |  |
|  |  | hsa-miR-3613-3p |  |
|  |  | hsa-miR-3613-5p |  |
|  |  | hsa-miR-3614-5p |  |
|  |  | hsa-miR-361-5p |  |
|  |  | hsa-miR-3616-3p |  |
|  |  | hsa-miR-3616-5p |  |
|  |  | hsa-miR-3616-5p |  |
|  |  | hsa-miR-3618 |  |
|  |  | hsa-miR-3619-5p |  |
|  |  | hsa-miR-3646 |  |
|  |  | hsa-miR-3649 |  |
|  |  | hsa-miR-3662 |  |
|  |  | hsa-miR-3665 |  |
|  |  | hsa-miR-3668 |  |
|  |  | hsa-miR-3671 |  |
|  |  | hsa-miR-3671 |  |
|  |  | hsa-miR-3671 |  |
|  |  | hsa-miR-3671 |  |
|  |  | hsa-miR-3679-3p |  |
|  |  | hsa-miR-3681-3p |  |
|  |  | hsa-miR-3688-3p |  |
|  |  | hsa-miR-3688-5p |  |
|  |  | hsa-miR-3689a-5p |  |
|  |  | hsa-miR-3689b-5p |  |
|  |  | hsa-miR-3689e |  |
|  |  | hsa-miR-3689f |  |
|  |  | hsa-miR-369-5p |  |
|  |  | hsa-miR-370-5p |  |
|  |  | hsa-miR-374a-3p |  |
|  |  | hsa-miR-374a-3p |  |
|  |  | hsa-miR-374a-3p |  |
|  |  | hsa-miR-374b-3p |  |
|  |  | hsa-miR-374c-5p |  |
|  |  | hsa-miR-376a-2-5p |  |
|  |  | hsa-miR-376a-2-5p |  |
|  |  | hsa-miR-376a-5p |  |
|  |  | hsa-miR-379-3p |  |
|  |  | hsa-miR-380-3p |  |
|  |  | hsa-miR-380-5p |  |
|  |  | hsa-miR-380-5p |  |
|  |  | hsa-miR-381-3p |  |
|  |  | hsa-miR-382-3p |  |
|  |  | hsa-miR-383-3p |  |
|  |  | hsa-miR-3908 |  |
|  |  | hsa-miR-3909 |  |
|  |  | hsa-miR-3913-3p |  |
|  |  | hsa-miR-3914 |  |
|  |  | hsa-miR-3916 |  |
|  |  | hsa-miR-3920 |  |
|  |  | hsa-miR-3922-5p |  |
|  |  | hsa-miR-3924 |  |
|  |  | hsa-miR-3925-5p |  |
|  |  | hsa-miR-3935 |  |
|  |  | hsa-miR-3940-5p |  |
|  |  | hsa-miR-3942-5p |  |
|  |  | hsa-miR-3945 |  |
|  |  | hsa-miR-3973 |  |
|  |  | hsa-miR-3973 |  |
|  |  | hsa-miR-3973 |  |
|  |  | hsa-miR-3976 |  |
|  |  | hsa-miR-410-3p |  |
|  |  | hsa-miR-411-3p |  |
|  |  | hsa-miR-412-3p |  |
|  |  | hsa-miR-421 |  |
|  |  | hsa-miR-424-5p |  |
|  |  | hsa-miR-424-5p |  |
|  |  | hsa-miR-4255 |  |
|  |  | hsa-miR-4270 |  |
|  |  | hsa-miR-4272 |  |
|  |  | hsa-miR-4272 |  |
|  |  | hsa-miR-4274 |  |
|  |  | hsa-miR-4276 |  |
|  |  | hsa-miR-4277 |  |
|  |  | hsa-miR-4282 |  |
|  |  | hsa-miR-4282 |  |
|  |  | hsa-miR-4282 |  |
|  |  | hsa-miR-4282 |  |
|  |  | hsa-miR-4282 |  |
|  |  | hsa-miR-4299 |  |
|  |  | hsa-miR-4328 |  |
|  |  | hsa-miR-4328 |  |
|  |  | hsa-miR-4418 |  |
|  |  | hsa-miR-4419a |  |
|  |  | hsa-miR-4420 |  |
|  |  | hsa-miR-4422 |  |
|  |  | hsa-miR-4426 |  |
|  |  | hsa-miR-4426 |  |
|  |  | hsa-miR-4428 |  |
|  |  | hsa-miR-4431 |  |
|  |  | hsa-miR-4436b-5p |  |
|  |  | hsa-miR-4438 |  |
|  |  | hsa-miR-4438 |  |
|  |  | hsa-miR-4439 |  |
|  |  | hsa-miR-4441 |  |
|  |  | hsa-miR-4446-5p |  |
|  |  | hsa-miR-4446-5p |  |
|  |  | hsa-miR-4452 |  |
|  |  | hsa-miR-4456 |  |
|  |  | hsa-miR-4457 |  |
|  |  | hsa-miR-4465 |  |
|  |  | hsa-miR-4470 |  |
|  |  | hsa-miR-4471 |  |
|  |  | hsa-miR-4476 |  |
|  |  | hsa-miR-4477a |  |
|  |  | hsa-miR-4477a |  |
|  |  | hsa-miR-4477a |  |
|  |  | hsa-miR-4477a |  |
|  |  | hsa-miR-4477b |  |
|  |  | hsa-miR-4477b |  |
|  |  | hsa-miR-4495 |  |
|  |  | hsa-miR-4495 |  |
|  |  | hsa-miR-4496 |  |
|  |  | hsa-miR-449a |  |
|  |  | hsa-miR-449b-5p |  |
|  |  | hsa-miR-4501 |  |
|  |  | hsa-miR-4502 |  |
|  |  | hsa-miR-4503 |  |
|  |  | hsa-miR-4507 |  |
|  |  | hsa-miR-450b-5p |  |
|  |  | hsa-miR-4510 |  |
|  |  | hsa-miR-4517 |  |
|  |  | hsa-miR-4517 |  |
|  |  | hsa-miR-4517 |  |
|  |  | hsa-miR-4520-2-3p |  |
|  |  | hsa-miR-4520-3p |  |
|  |  | hsa-miR-4524a-5p |  |
|  |  | hsa-miR-4524a-5p |  |
|  |  | hsa-miR-4524b-3p |  |
|  |  | hsa-miR-4524b-5p |  |
|  |  | hsa-miR-4524b-5p |  |
|  |  | hsa-miR-4525 |  |
|  |  | hsa-miR-452-5p |  |
|  |  | hsa-miR-452-5p |  |
|  |  | hsa-miR-4529-3p |  |
|  |  | hsa-miR-4532 |  |
|  |  | hsa-miR-4533 |  |
|  |  | hsa-miR-4536-3p |  |
|  |  | hsa-miR-455-3p.1 |  |
|  |  | hsa-miR-455-5p |  |
|  |  | hsa-miR-4639-3p |  |
|  |  | hsa-miR-4639-3p |  |
|  |  | hsa-miR-4639-5p |  |
|  |  | hsa-miR-4645-3p |  |
|  |  | hsa-miR-4646-3p |  |
|  |  | hsa-miR-4647 |  |
|  |  | hsa-miR-4647 |  |
|  |  | hsa-miR-4652-3p |  |
|  |  | hsa-miR-4658 |  |
|  |  | hsa-miR-4659a-3p |  |
|  |  | hsa-miR-4659a-3p |  |
|  |  | hsa-miR-4659b-3p |  |
|  |  | hsa-miR-4659b-3p |  |
|  |  | hsa-miR-4662b |  |
|  |  | hsa-miR-4662b |  |
|  |  | hsa-miR-4665-5p |  |
|  |  | hsa-miR-4666a-5p |  |
|  |  | hsa-miR-4666b |  |
|  |  | hsa-miR-4667-5p |  |
|  |  | hsa-miR-4667-5p |  |
|  |  | hsa-miR-4668-3p |  |
|  |  | hsa-miR-4676-3p |  |
|  |  | hsa-miR-4676-3p |  |
|  |  | hsa-miR-4686 |  |
|  |  | hsa-miR-4686 |  |
|  |  | hsa-miR-4691-5p |  |
|  |  | hsa-miR-4691-5p |  |
|  |  | hsa-miR-4693-5p |  |
|  |  | hsa-miR-4698 |  |
|  |  | hsa-miR-4698 |  |
|  |  | hsa-miR-4699-3p |  |
|  |  | hsa-miR-4699-3p |  |
|  |  | hsa-miR-4699-3p |  |
|  |  | hsa-miR-4700-5p |  |
|  |  | hsa-miR-4700-5p |  |
|  |  | hsa-miR-4703-5p |  |
|  |  | hsa-miR-4709-5p |  |
|  |  | hsa-miR-4709-5p |  |
|  |  | hsa-miR-4711-3p |  |
|  |  | hsa-miR-4712-3p |  |
|  |  | hsa-miR-4713-5p |  |
|  |  | hsa-miR-4719 |  |
|  |  | hsa-miR-4722-3p |  |
|  |  | hsa-miR-4723-5p |  |
|  |  | hsa-miR-4724-3p |  |
|  |  | hsa-miR-4728-3p |  |
|  |  | hsa-miR-4728-5p |  |
|  |  | hsa-miR-4731-3p |  |
|  |  | hsa-miR-4732-3p |  |
|  |  | hsa-miR-4733-3p |  |
|  |  | hsa-miR-4739 |  |
|  |  | hsa-miR-4755-5p |  |
|  |  | hsa-miR-4756-5p |  |
|  |  | hsa-miR-4758-5p |  |
|  |  | hsa-miR-4760-3p |  |
|  |  | hsa-miR-4762-5p |  |
|  |  | hsa-miR-4762-5p |  |
|  |  | hsa-miR-4762-5p |  |
|  |  | hsa-miR-4763-5p |  |
|  |  | hsa-miR-4764-3p |  |
|  |  | hsa-miR-4765 |  |
|  |  | hsa-miR-4766-3p |  |
|  |  | hsa-miR-4772-3p |  |
|  |  | hsa-miR-4773 |  |
|  |  | hsa-miR-4774-3p |  |
|  |  | hsa-miR-4777-5p |  |
|  |  | hsa-miR-4778-3p |  |
|  |  | hsa-miR-4778-5p |  |
|  |  | hsa-miR-4780 |  |
|  |  | hsa-miR-4781-3p |  |
|  |  | hsa-miR-4786-3p |  |
|  |  | hsa-miR-4789-3p |  |
|  |  | hsa-miR-4789-5p |  |
|  |  | hsa-miR-4791 |  |
|  |  | hsa-miR-4793-3p |  |
|  |  | hsa-miR-4793-3p |  |
|  |  | hsa-miR-4793-5p |  |
|  |  | hsa-miR-4795-5p |  |
|  |  | hsa-miR-4795-5p |  |
|  |  | hsa-miR-4799-5p |  |
|  |  | hsa-miR-4799-5p |  |
|  |  | hsa-miR-4799-5p |  |
|  |  | hsa-miR-4801 |  |
|  |  | hsa-miR-486-3p |  |
|  |  | hsa-miR-486-5p |  |
|  |  | hsa-miR-488-3p |  |
|  |  | hsa-miR-489-3p |  |
|  |  | hsa-miR-489-3p |  |
|  |  | hsa-miR-491-3p |  |
|  |  | hsa-miR-491-3p |  |
|  |  | hsa-miR-494-3p |  |
|  |  | hsa-miR-495-3p |  |
|  |  | hsa-miR-495-3p |  |
|  |  | hsa-miR-497-5p |  |
|  |  | hsa-miR-497-5p |  |
|  |  | hsa-miR-498 |  |
|  |  | hsa-miR-499a-3p |  |
|  |  | hsa-miR-499b-3p |  |
|  |  | hsa-miR-5002-3p |  |
|  |  | hsa-miR-5002-5p |  |
|  |  | hsa-miR-5003-3p |  |
|  |  | hsa-miR-5003-3p |  |
|  |  | hsa-miR-5003-5p |  |
|  |  | hsa-miR-5006-3p |  |
|  |  | hsa-miR-5008-3p |  |
|  |  | hsa-miR-5010-5p |  |
|  |  | hsa-miR-5011-3p |  |
|  |  | hsa-miR-503-3p |  |
|  |  | hsa-miR-503-5p |  |
|  |  | hsa-miR-505-3p.1 |  |
|  |  | hsa-miR-506-5p |  |
|  |  | hsa-miR-509-3-5p |  |
|  |  | hsa-miR-5094 |  |
|  |  | hsa-miR-5094 |  |
|  |  | hsa-miR-5095 |  |
|  |  | hsa-miR-509-5p |  |
|  |  | hsa-miR-511-5p |  |
|  |  | hsa-miR-513a-3p |  |
|  |  | hsa-miR-513a-5p |  |
|  |  | hsa-miR-513b-3p |  |
|  |  | hsa-miR-513b-3p |  |
|  |  | hsa-miR-513b-3p |  |
|  |  | hsa-miR-513b-5p |  |
|  |  | hsa-miR-513c-3p |  |
|  |  | hsa-miR-514a-3p |  |
|  |  | hsa-miR-514a-5p |  |
|  |  | hsa-miR-514b-3p |  |
|  |  | hsa-miR-516a-3p |  |
|  |  | hsa-miR-516b-3p |  |
|  |  | hsa-miR-517a-3p |  |
|  |  | hsa-miR-517a-3p |  |
|  |  | hsa-miR-517b-3p |  |
|  |  | hsa-miR-517b-3p |  |
|  |  | hsa-miR-517c-3p |  |
|  |  | hsa-miR-517c-3p |  |
|  |  | hsa-miR-5187-3p |  |
|  |  | hsa-miR-518a-5p |  |
|  |  | hsa-miR-518d-5p |  |
|  |  | hsa-miR-518e-5p |  |
|  |  | hsa-miR-518f-5p |  |
|  |  | hsa-miR-5190 |  |
|  |  | hsa-miR-5190 |  |
|  |  | hsa-miR-5195-3p |  |
|  |  | hsa-miR-5197-5p |  |
|  |  | hsa-miR-519a-5p |  |
|  |  | hsa-miR-519b-5p |  |
|  |  | hsa-miR-519c-5p |  |
|  |  | hsa-miR-519d-5p |  |
|  |  | hsa-miR-520c-5p |  |
|  |  | hsa-miR-520d-5p |  |
|  |  | hsa-miR-520g-3p |  |
|  |  | hsa-miR-520g-3p |  |
|  |  | hsa-miR-520g-5p |  |
|  |  | hsa-miR-520h |  |
|  |  | hsa-miR-520h |  |
|  |  | hsa-miR-522-5p |  |
|  |  | hsa-miR-523-5p |  |
|  |  | hsa-miR-524-5p |  |
|  |  | hsa-miR-526a |  |
|  |  | hsa-miR-527 |  |
|  |  | hsa-miR-539-5p |  |
|  |  | hsa-miR-539-5p |  |
|  |  | hsa-miR-539-5p |  |
|  |  | hsa-miR-541-5p |  |
|  |  | hsa-miR-543 |  |
|  |  | hsa-miR-543 |  |
|  |  | hsa-miR-545-3p |  |
|  |  | hsa-miR-545-5p |  |
|  |  | hsa-miR-545-5p |  |
|  |  | hsa-miR-548a-3p |  |
|  |  | hsa-miR-548a-3p |  |
|  |  | hsa-miR-548a-5p |  |
|  |  | hsa-miR-548a-5p |  |
|  |  | hsa-miR-548ab |  |
|  |  | hsa-miR-548ab |  |
|  |  | hsa-miR-548ac |  |
|  |  | hsa-miR-548ad-5p |  |
|  |  | hsa-miR-548ad-5p |  |
|  |  | hsa-miR-548ae-3p |  |
|  |  | hsa-miR-548ae-3p |  |
|  |  | hsa-miR-548ae-5p |  |
|  |  | hsa-miR-548ae-5p |  |
|  |  | hsa-miR-548ag |  |
|  |  | hsa-miR-548ah-3p |  |
|  |  | hsa-miR-548ah-3p |  |
|  |  | hsa-miR-548ah-5p |  |
|  |  | hsa-miR-548ai |  |
|  |  | hsa-miR-548aj-3p |  |
|  |  | hsa-miR-548aj-3p |  |
|  |  | hsa-miR-548aj-5p |  |
|  |  | hsa-miR-548ak |  |
|  |  | hsa-miR-548ak |  |
|  |  | hsa-miR-548am-3p |  |
|  |  | hsa-miR-548am-3p |  |
|  |  | hsa-miR-548am-5p |  |
|  |  | hsa-miR-548am-5p |  |
|  |  | hsa-miR-548ao-5p |  |
|  |  | hsa-miR-548ap-5p |  |
|  |  | hsa-miR-548ap-5p |  |
|  |  | hsa-miR-548aq-3p |  |
|  |  | hsa-miR-548aq-3p |  |
|  |  | hsa-miR-548aq-5p |  |
|  |  | hsa-miR-548aq-5p |  |
|  |  | hsa-miR-548ar-3p |  |
|  |  | hsa-miR-548ar-3p |  |
|  |  | hsa-miR-548ar-5p |  |
|  |  | hsa-miR-548ar-5p |  |
|  |  | hsa-miR-548as-5p |  |
|  |  | hsa-miR-548as-5p |  |
|  |  | hsa-miR-548au-3p |  |
|  |  | hsa-miR-548au-5p |  |
|  |  | hsa-miR-548au-5p |  |
|  |  | hsa-miR-548av-3p |  |
|  |  | hsa-miR-548av-3p |  |
|  |  | hsa-miR-548av-5p |  |
|  |  | hsa-miR-548aw |  |
|  |  | hsa-miR-548ax |  |
|  |  | hsa-miR-548ay-5p |  |
|  |  | hsa-miR-548ay-5p |  |
|  |  | hsa-miR-548az-3p |  |
|  |  | hsa-miR-548az-3p |  |
|  |  | hsa-miR-548az-5p |  |
|  |  | hsa-miR-548b-5p |  |
|  |  | hsa-miR-548b-5p |  |
|  |  | hsa-miR-548ba |  |
|  |  | hsa-miR-548bb-3p |  |
|  |  | hsa-miR-548bb-5p |  |
|  |  | hsa-miR-548bb-5p |  |
|  |  | hsa-miR-548c-3p |  |
|  |  | hsa-miR-548c-3p |  |
|  |  | hsa-miR-548c-3p |  |
|  |  | hsa-miR-548c-3p |  |
|  |  | hsa-miR-548c-5p |  |
|  |  | hsa-miR-548c-5p |  |
|  |  | hsa-miR-548d-3p |  |
|  |  | hsa-miR-548d-5p |  |
|  |  | hsa-miR-548d-5p |  |
|  |  | hsa-miR-548e-3p |  |
|  |  | hsa-miR-548e-3p |  |
|  |  | hsa-miR-548f-3p |  |
|  |  | hsa-miR-548f-3p |  |
|  |  | hsa-miR-548f-5p |  |
|  |  | hsa-miR-548g-3p |  |
|  |  | hsa-miR-548g-3p |  |
|  |  | hsa-miR-548g-5p |  |
|  |  | hsa-miR-548h-3p |  |
|  |  | hsa-miR-548h-5p |  |
|  |  | hsa-miR-548h-5p |  |
|  |  | hsa-miR-548i |  |
|  |  | hsa-miR-548i |  |
|  |  | hsa-miR-548j-3p |  |
|  |  | hsa-miR-548j-3p |  |
|  |  | hsa-miR-548j-5p |  |
|  |  | hsa-miR-548j-5p |  |
|  |  | hsa-miR-548k |  |
|  |  | hsa-miR-548l |  |
|  |  | hsa-miR-548l |  |
|  |  | hsa-miR-548m |  |
|  |  | hsa-miR-548n |  |
|  |  | hsa-miR-548o-5p |  |
|  |  | hsa-miR-548o-5p |  |
|  |  | hsa-miR-548p |  |
|  |  | hsa-miR-548q |  |
|  |  | hsa-miR-548t-5p |  |
|  |  | hsa-miR-548w |  |
|  |  | hsa-miR-548w |  |
|  |  | hsa-miR-548x-3p |  |
|  |  | hsa-miR-548x-3p |  |
|  |  | hsa-miR-548x-5p |  |
|  |  | hsa-miR-548y |  |
|  |  | hsa-miR-548y |  |
|  |  | hsa-miR-548z |  |
|  |  | hsa-miR-549a |  |
|  |  | hsa-miR-550a-3-5p |  |
|  |  | hsa-miR-550a-3p |  |
|  |  | hsa-miR-550a-5p |  |
|  |  | hsa-miR-550b-2-5p |  |
|  |  | hsa-miR-551b-5p |  |
|  |  | hsa-miR-551b-5p |  |
|  |  | hsa-miR-551b-5p |  |
|  |  | hsa-miR-5579-3p |  |
|  |  | hsa-miR-5580-3p |  |
|  |  | hsa-miR-5582-3p |  |
|  |  | hsa-miR-5583-3p |  |
|  |  | hsa-miR-5583-3p |  |
|  |  | hsa-miR-5583-3p |  |
|  |  | hsa-miR-5583-5p |  |
|  |  | hsa-miR-5583-5p |  |
|  |  | hsa-miR-5583-5p |  |
|  |  | hsa-miR-5584-5p |  |
|  |  | hsa-miR-5585-5p |  |
|  |  | hsa-miR-559 |  |
|  |  | hsa-miR-559 |  |
|  |  | hsa-miR-563 |  |
|  |  | hsa-miR-563 |  |
|  |  | hsa-miR-567 |  |
|  |  | hsa-miR-5680 |  |
|  |  | hsa-miR-5680 |  |
|  |  | hsa-miR-5680 |  |
|  |  | hsa-miR-5680 |  |
|  |  | hsa-miR-5681a |  |
|  |  | hsa-miR-5681b |  |
|  |  | hsa-miR-5688 |  |
|  |  | hsa-miR-5688 |  |
|  |  | hsa-miR-5690 |  |
|  |  | hsa-miR-5691 |  |
|  |  | hsa-miR-5692a |  |
|  |  | hsa-miR-5692b |  |
|  |  | hsa-miR-5692b |  |
|  |  | hsa-miR-5692c |  |
|  |  | hsa-miR-5692c |  |
|  |  | hsa-miR-5693 |  |
|  |  | hsa-miR-5696 |  |
|  |  | hsa-miR-5697 |  |
|  |  | hsa-miR-5698 |  |
|  |  | hsa-miR-5700 |  |
|  |  | hsa-miR-570-5p |  |
|  |  | hsa-miR-573 |  |
|  |  | hsa-miR-573 |  |
|  |  | hsa-miR-577 |  |
|  |  | hsa-miR-579-3p |  |
|  |  | hsa-miR-582-5p |  |
|  |  | hsa-miR-584-5p |  |
|  |  | hsa-miR-586 |  |
|  |  | hsa-miR-586 |  |
|  |  | hsa-miR-589-3p |  |
|  |  | hsa-miR-589-3p |  |
|  |  | hsa-miR-590-3p |  |
|  |  | hsa-miR-590-3p |  |
|  |  | hsa-miR-590-5p |  |
|  |  | hsa-miR-590-5p |  |
|  |  | hsa-miR-595 |  |
|  |  | hsa-miR-599 |  |
|  |  | hsa-miR-600 |  |
|  |  | hsa-miR-601 |  |
|  |  | hsa-miR-605-5p |  |
|  |  | hsa-miR-605-5p |  |
|  |  | hsa-miR-606 |  |
|  |  | hsa-miR-607 |  |
|  |  | hsa-miR-607 |  |
|  |  | hsa-miR-607 |  |
|  |  | hsa-miR-607 |  |
|  |  | hsa-miR-6074 |  |
|  |  | hsa-miR-6074 |  |
|  |  | hsa-miR-6077 |  |
|  |  | hsa-miR-6083 |  |
|  |  | hsa-miR-6083 |  |
|  |  | hsa-miR-6124 |  |
|  |  | hsa-miR-6127 |  |
|  |  | hsa-miR-6129 |  |
|  |  | hsa-miR-613 |  |
|  |  | hsa-miR-6130 |  |
|  |  | hsa-miR-6133 |  |
|  |  | hsa-miR-6165 |  |
|  |  | hsa-miR-621 |  |
|  |  | hsa-miR-623 |  |
|  |  | hsa-miR-624-3p |  |
|  |  | hsa-miR-624-5p |  |
|  |  | hsa-miR-625-3p |  |
|  |  | hsa-miR-625-5p |  |
|  |  | hsa-miR-627-3p |  |
|  |  | hsa-miR-629-3p |  |
|  |  | hsa-miR-630 |  |
|  |  | hsa-miR-636 |  |
|  |  | hsa-miR-637 |  |
|  |  | hsa-miR-637 |  |
|  |  | hsa-miR-640 |  |
|  |  | hsa-miR-642b-5p |  |
|  |  | hsa-miR-643 |  |
|  |  | hsa-miR-646 |  |
|  |  | hsa-miR-646 |  |
|  |  | hsa-miR-648 |  |
|  |  | hsa-miR-6500-3p |  |
|  |  | hsa-miR-6501-3p |  |
|  |  | hsa-miR-6504-3p |  |
|  |  | hsa-miR-6508-5p |  |
|  |  | hsa-miR-651-3p |  |
|  |  | hsa-miR-651-3p |  |
|  |  | hsa-miR-6514-3p |  |
|  |  | hsa-miR-653-5p |  |
|  |  | hsa-miR-655-3p |  |
|  |  | hsa-miR-656-3p |  |
|  |  | hsa-miR-656-3p |  |
|  |  | hsa-miR-656-3p |  |
|  |  | hsa-miR-659-3p |  |
|  |  | hsa-miR-659-5p |  |
|  |  | hsa-miR-664b-3p |  |
|  |  | hsa-miR-6715b-3p |  |
|  |  | hsa-miR-6716-5p |  |
|  |  | hsa-miR-6727-3p |  |
|  |  | hsa-miR-6730-5p |  |
|  |  | hsa-miR-6737-3p |  |
|  |  | hsa-miR-6742-3p |  |
|  |  | hsa-miR-6749-3p |  |
|  |  | hsa-miR-6750-3p |  |
|  |  | hsa-miR-6753-5p |  |
|  |  | hsa-miR-6754-3p |  |
|  |  | hsa-miR-6754-5p |  |
|  |  | hsa-miR-6758-5p |  |
|  |  | hsa-miR-6759-3p |  |
|  |  | hsa-miR-6759-3p |  |
|  |  | hsa-miR-676-3p |  |
|  |  | hsa-miR-6764-3p |  |
|  |  | hsa-miR-6778-5p |  |
|  |  | hsa-miR-6780b-3p |  |
|  |  | hsa-miR-6782-3p |  |
|  |  | hsa-miR-6783-3p |  |
|  |  | hsa-miR-6785-5p |  |
|  |  | hsa-miR-6790-5p |  |
|  |  | hsa-miR-6792-3p |  |
|  |  | hsa-miR-6792-3p |  |
|  |  | hsa-miR-6792-5p |  |
|  |  | hsa-miR-6792-5p |  |
|  |  | hsa-miR-6794-3p |  |
|  |  | hsa-miR-6794-3p |  |
|  |  | hsa-miR-6796-3p |  |
|  |  | hsa-miR-6801-3p |  |
|  |  | hsa-miR-6805-3p |  |
|  |  | hsa-miR-6810-3p |  |
|  |  | hsa-miR-6815-5p |  |
|  |  | hsa-miR-6816-3p |  |
|  |  | hsa-miR-6820-3p |  |
|  |  | hsa-miR-6820-3p |  |
|  |  | hsa-miR-6824-3p |  |
|  |  | hsa-miR-6830-3p |  |
|  |  | hsa-miR-6830-5p |  |
|  |  | hsa-miR-6835-3p |  |
|  |  | hsa-miR-6835-3p |  |
|  |  | hsa-miR-6837-3p |  |
|  |  | hsa-miR-6837-3p |  |
|  |  | hsa-miR-6838-5p |  |
|  |  | hsa-miR-6838-5p |  |
|  |  | hsa-miR-6852-3p |  |
|  |  | hsa-miR-6852-5p |  |
|  |  | hsa-miR-6853-3p |  |
|  |  | hsa-miR-6853-3p |  |
|  |  | hsa-miR-6856-5p |  |
|  |  | hsa-miR-6858-3p |  |
|  |  | hsa-miR-6859-5p |  |
|  |  | hsa-miR-6865-5p |  |
|  |  | hsa-miR-6866-3p |  |
|  |  | hsa-miR-6867-3p |  |
|  |  | hsa-miR-6870-5p |  |
|  |  | hsa-miR-6873-5p |  |
|  |  | hsa-miR-6875-3p |  |
|  |  | hsa-miR-6875-3p |  |
|  |  | hsa-miR-6876-5p |  |
|  |  | hsa-miR-6882-5p |  |
|  |  | hsa-miR-6883-3p |  |
|  |  | hsa-miR-6883-5p |  |
|  |  | hsa-miR-6894-3p |  |
|  |  | hsa-miR-7111-5p |  |
|  |  | hsa-miR-7-1-3p |  |
|  |  | hsa-miR-7-1-3p |  |
|  |  | hsa-miR-7-1-3p |  |
|  |  | hsa-miR-7-1-3p |  |
|  |  | hsa-miR-7151-3p |  |
|  |  | hsa-miR-7151-5p |  |
|  |  | hsa-miR-7155-5p |  |
|  |  | hsa-miR-7155-5p |  |
|  |  | hsa-miR-7157-3p |  |
|  |  | hsa-miR-7159-3p |  |
|  |  | hsa-miR-7162-5p |  |
|  |  | hsa-miR-7-2-3p |  |
|  |  | hsa-miR-7-2-3p |  |
|  |  | hsa-miR-7-2-3p |  |
|  |  | hsa-miR-7-2-3p |  |
|  |  | hsa-miR-758-5p |  |
|  |  | hsa-miR-7-5p |  |
|  |  | hsa-miR-7-5p |  |
|  |  | hsa-miR-761 |  |
|  |  | hsa-miR-7843-3p |  |
|  |  | hsa-miR-7843-5p |  |
|  |  | hsa-miR-7843-5p |  |
|  |  | hsa-miR-7844-5p |  |
|  |  | hsa-miR-7844-5p |  |
|  |  | hsa-miR-7850-5p |  |
|  |  | hsa-miR-7852-3p |  |
|  |  | hsa-miR-7853-5p |  |
|  |  | hsa-miR-7977 |  |
|  |  | hsa-miR-7978 |  |
|  |  | hsa-miR-8054 |  |
|  |  | hsa-miR-8059 |  |
|  |  | hsa-miR-8063 |  |
|  |  | hsa-miR-8063 |  |
|  |  | hsa-miR-8063 |  |
|  |  | hsa-miR-8064 |  |
|  |  | hsa-miR-8065 |  |
|  |  | hsa-miR-8066 |  |
|  |  | hsa-miR-8067 |  |
|  |  | hsa-miR-8076 |  |
|  |  | hsa-miR-8076 |  |
|  |  | hsa-miR-8079 |  |
|  |  | hsa-miR-8079 |  |
|  |  | hsa-miR-8080 |  |
|  |  | hsa-miR-8081 |  |
|  |  | hsa-miR-8083 |  |
|  |  | hsa-miR-8089 |  |
|  |  | hsa-miR-8089 |  |
|  |  | hsa-miR-877-5p |  |
|  |  | hsa-miR-885-5p |  |
|  |  | hsa-miR-888-3p |  |
|  |  | hsa-miR-889-3p |  |
|  |  | hsa-miR-892a |  |
|  |  | hsa-miR-892c-3p |  |
|  |  | hsa-miR-892c-3p |  |
|  |  | hsa-miR-934 |  |
|  |  | hsa-miR-936 |  |
|  |  | hsa-miR-939-3p |  |
|  |  | hsa-miR-943 |  |
|  |  | hsa-miR-944 |  |
|  |  | hsa-miR-944 |  |
|  |  | hsa-miR-95-5p |  |
|  |  | hsa-miR-9-5p |  |
|  |  | hsa-miR-96-5p |  |
|  |  | hsa-miR-98-3p |  |
|  |  | hsa-miR-99a-3p |  |
|  |  | hsa-miR-99b-3p |  |
