# Supplementary figures and images for: CircRNF111 Protects Against Insulin Resistance and Lipid Deposition via Regulating miR-143-3p/IGF2R Axis in Metabolic Syndrome
Source: Front Cell Dev Biol. 2021 Aug 17;9:663148. doi: 10.3389/fcell.2021.663148 (PMC8415985; doi:10.3389/fcell.2021.663148)

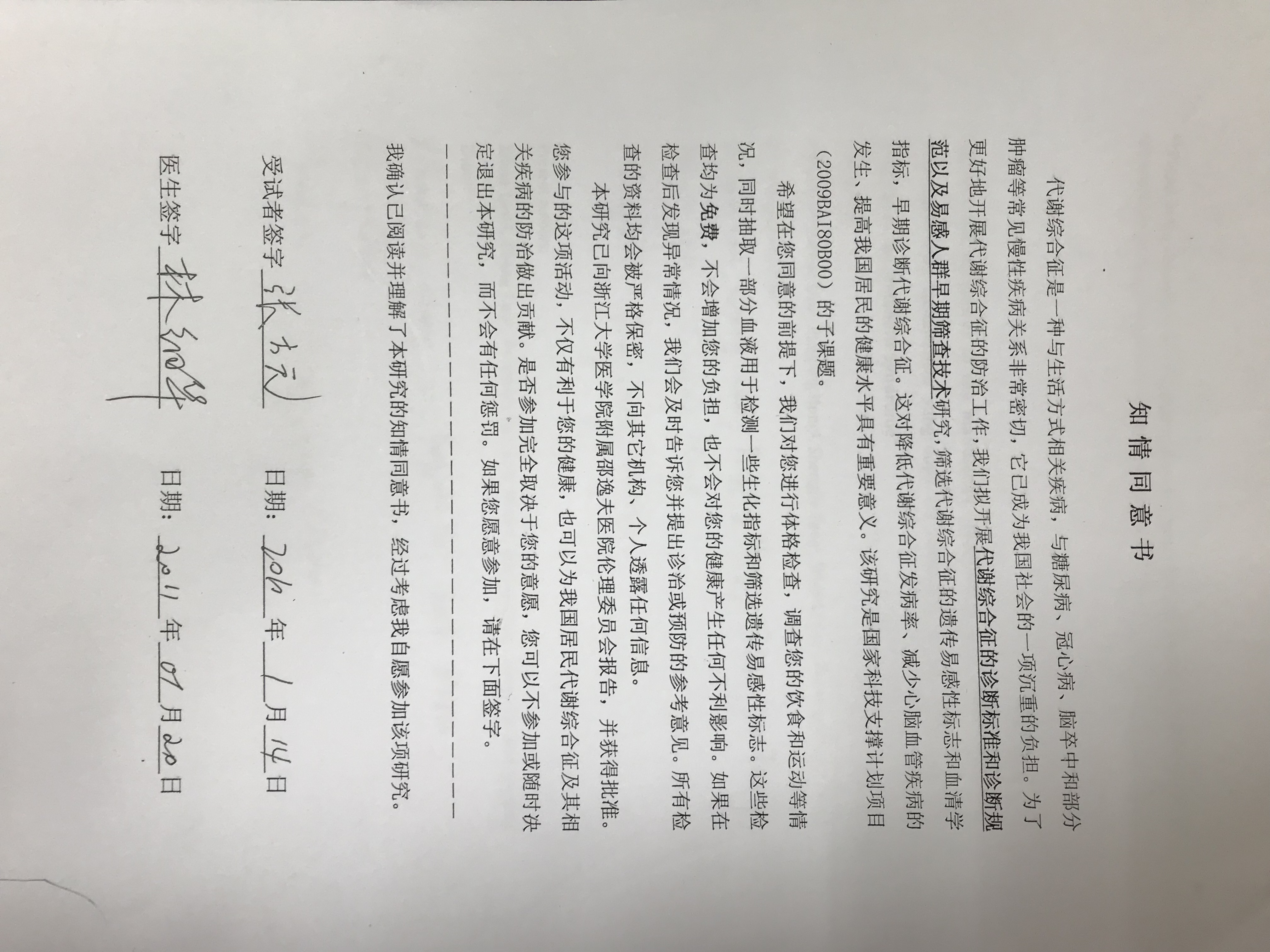

Supplement: Supplementary file 1 [file Data_Sheet_1.ZIP › Supplemental File Sets/An exemplary signed of written informed consent from patient.jpg]

Full unedited gel for Figure 2

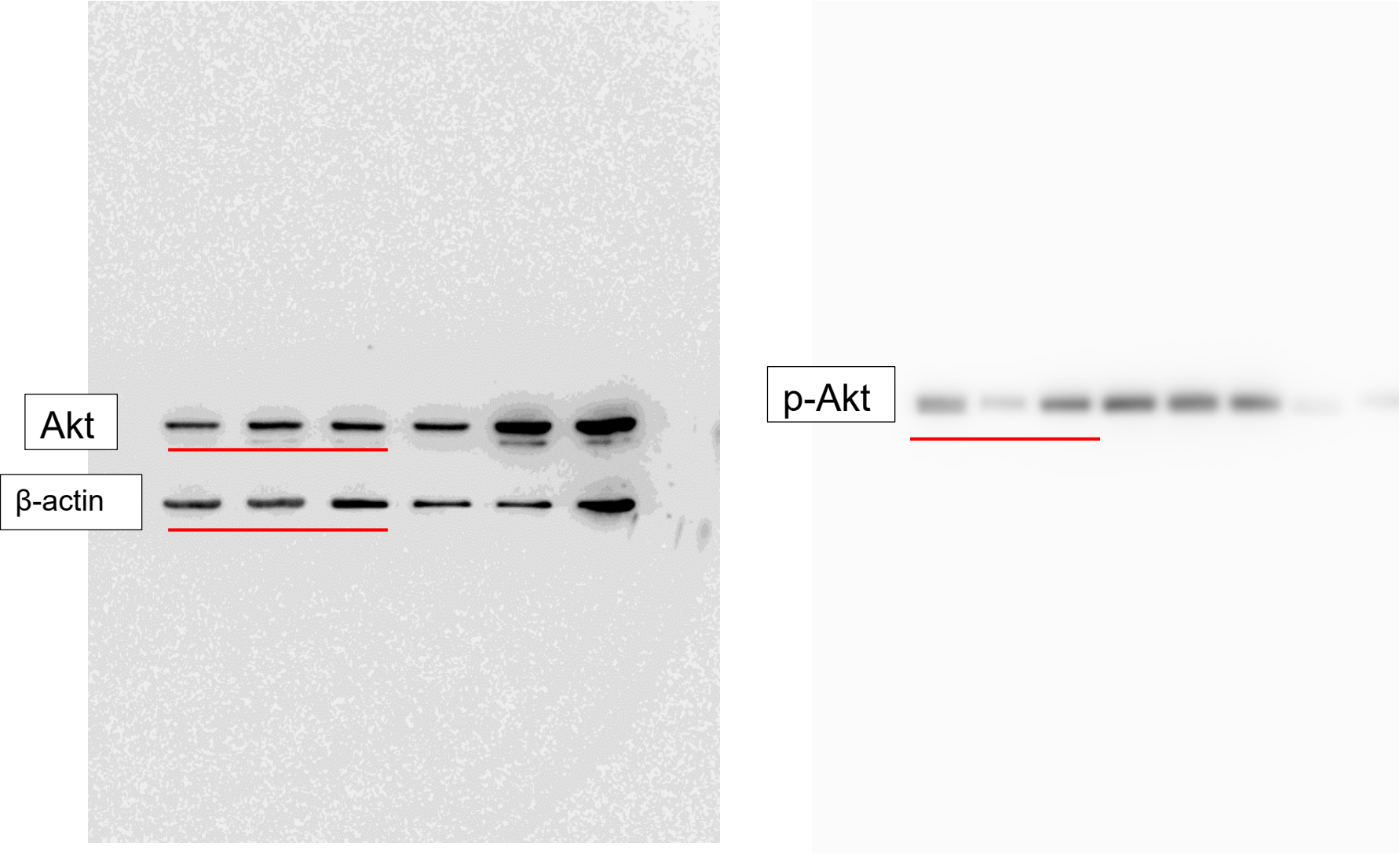

Full unedited gel for Figure 4

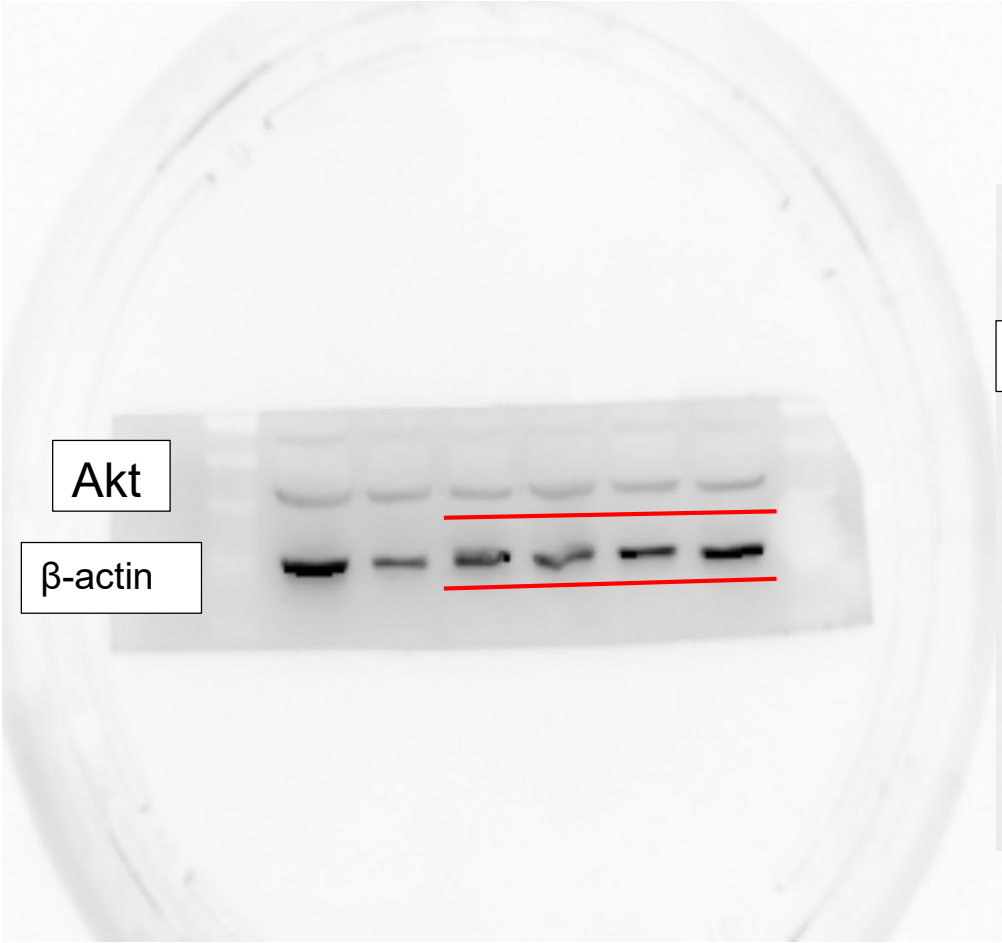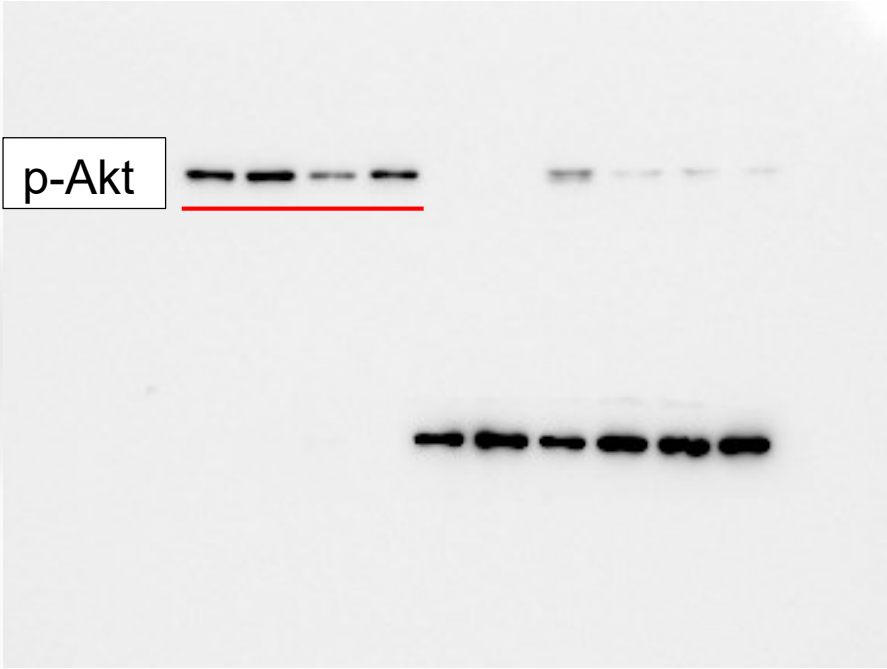

Full unedited gel for Figure 5

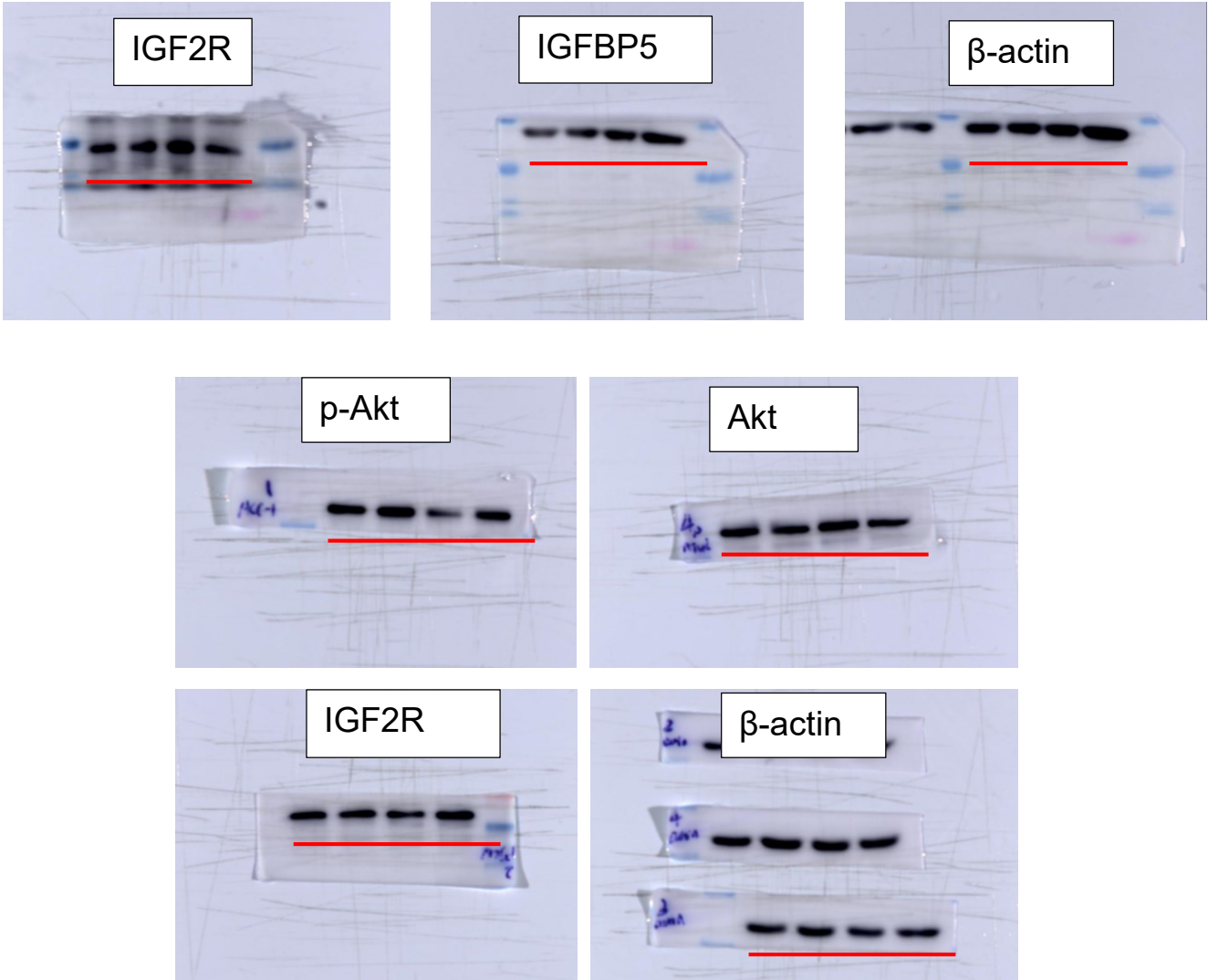

Supplement: Supplementary file 1 [file Data_Sheet_1.ZIP › Supplemental File Sets/Full uncut gels.pdf]

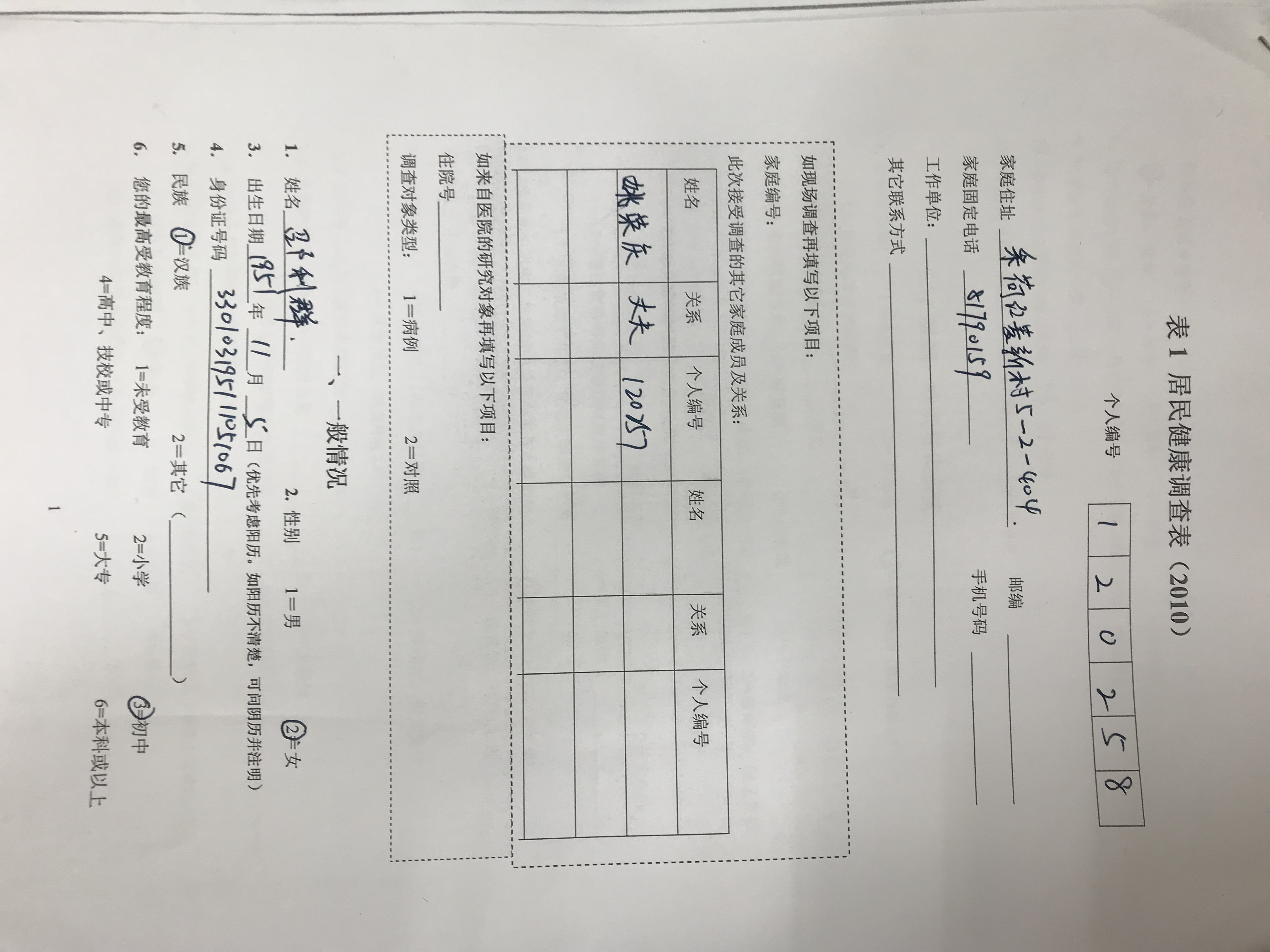

Supplement: Supplementary file 1 [file Data_Sheet_1.ZIP › Supplemental File Sets/Home page of questionnaire.jpg]

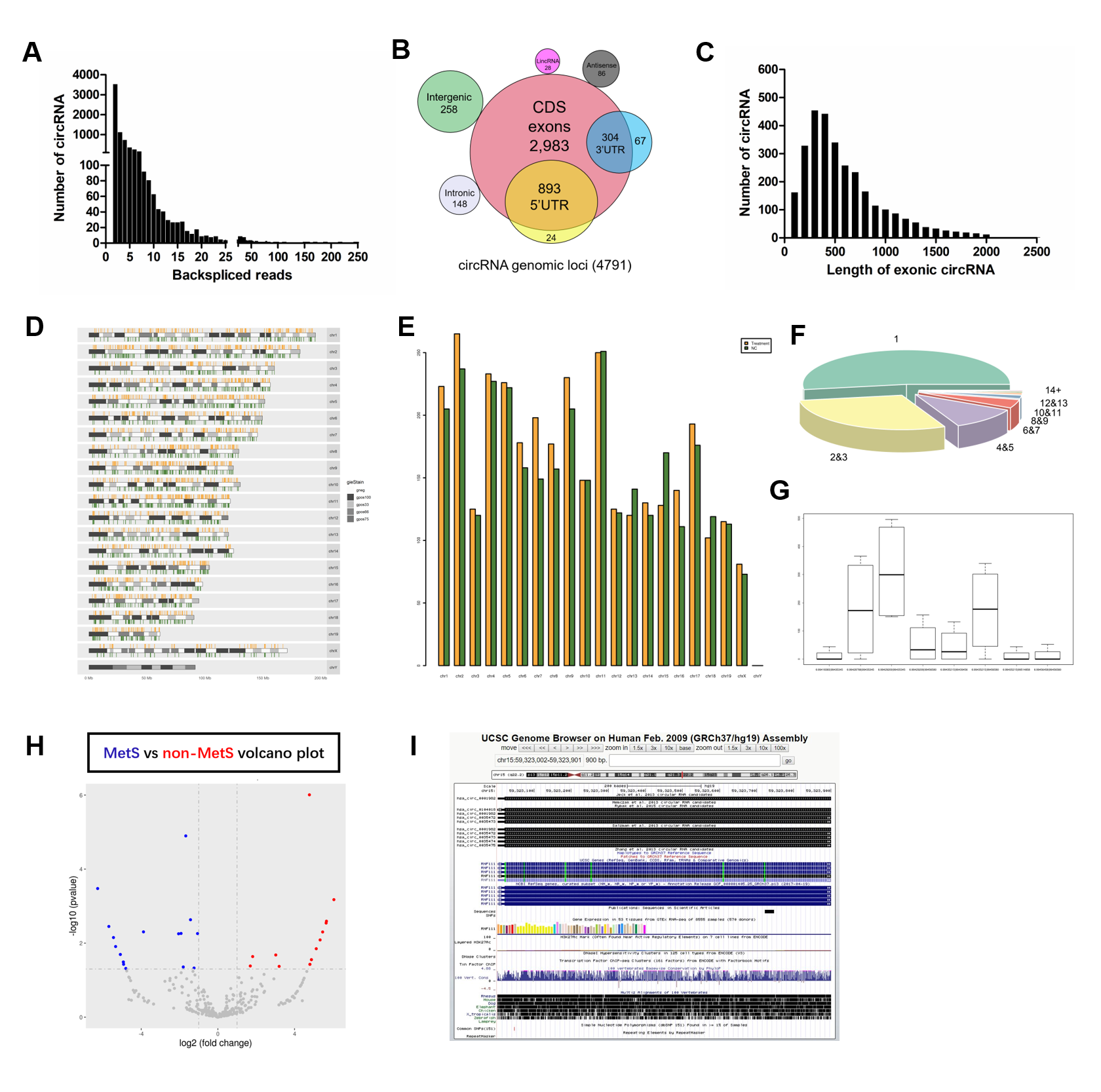

Supplement: Supplementary file 1 [file Data_Sheet_1.ZIP › Supplemental File Sets/Supplementary Figure1.tif]

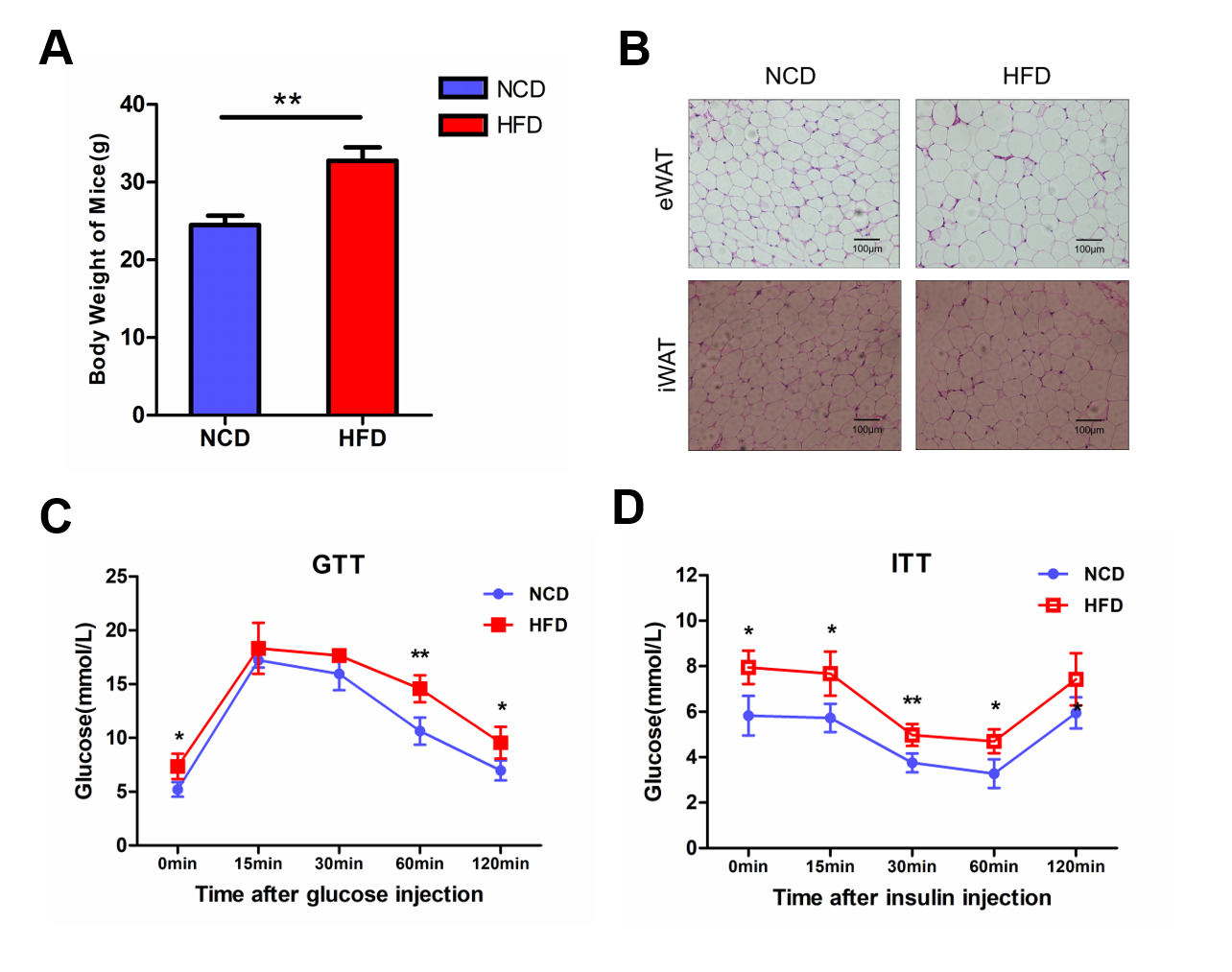

Supplement: Supplementary file 1 [file Data_Sheet_1.ZIP › Supplemental File Sets/Supplementary Figure2.tif]

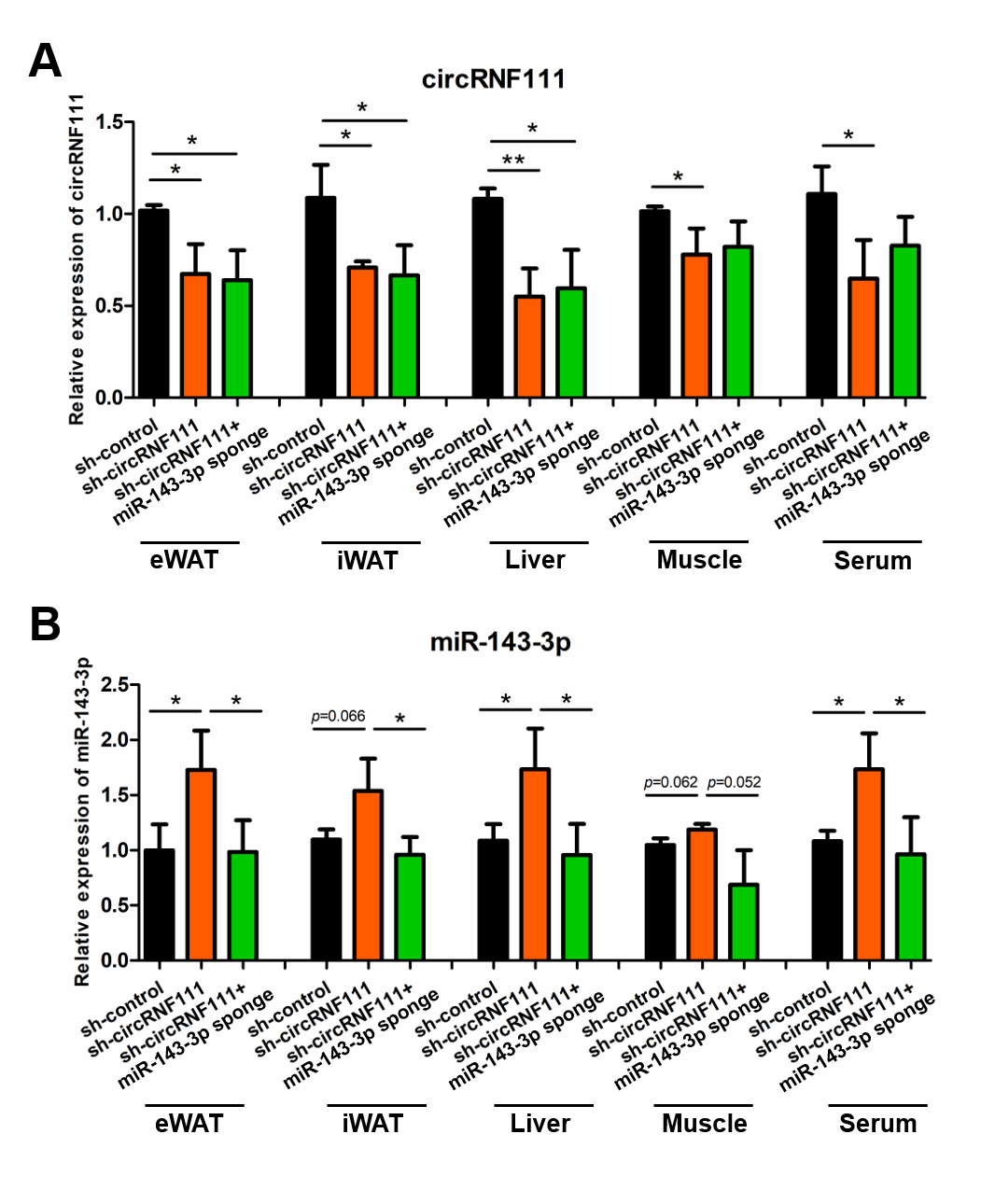

Supplement: Supplementary file 1 [file Data_Sheet_1.ZIP › Supplemental File Sets/Supplementary Figure3.tif]
